# Multi-Omic Data Improve Prediction of Personalized Tumor Suppressors and Oncogenes

**DOI:** 10.3389/fgene.2022.854190

**Published:** 2022-05-10

**Authors:** Malvika Sudhakar, Raghunathan Rengaswamy, Karthik Raman

**Affiliations:** ^1^ Centre for Integrative Biology and Systems mEdicine (IBSE), Indian Institute of Technology (IIT) Madras, Chennai, India; ^2^ Robert Bosch Center for Data Science and Artificial Intelligence (RBCDSAI), IIT Madras, Chennai, India; ^3^ Department of Biotechnology, Bhupat and Jyoti Mehta School of Biosciences, IIT Madras, Chennai, India; ^4^ Department of Chemical Engineering, IIT Madras, Chennai, India

**Keywords:** machine learning, driver genes, personalized driver genes, cancer genomics, PIVOT

## Abstract

The progression of tumorigenesis starts with a few mutational and structural driver events in the cell. Various cohort-based computational tools exist to identify driver genes but require multiple samples to identify less frequently mutated driver genes. Many studies use different methods to identify driver mutations/genes from mutations that have no impact on tumor progression; however, a small fraction of patients show no mutational events in any known driver genes. Current unsupervised methods map somatic and expression data onto a network to identify personalized driver genes based on changes in expression. Our method is the first machine learning model to classify genes as tumor suppressor gene (TSG), oncogene (OG), or neutral, thus assigning the functional impact of the gene in the patient. In this study, we develop a multi-omic approach, PIVOT (Personalized Identification of driVer OGs and TSGs), to train on experimentally or computationally validated mutational and structural driver events. Given the lack of any gold standards for the identification of personalized driver genes, we label the data using four strategies and, based on classification metrics, show gene-based labeling strategies perform best. We build different models using SNV, RNA, and multi-omic features to be used based on the data available. Our models trained on multi-omic data improved predictions compared with mutation and expression data, achieving an accuracy ≥0.99 for BRCA, LUAD, and COAD datasets. We show network and expression-based features contribute the most to PIVOT. Our predictions on BRCA, COAD, and LUAD cancer types reveal commonly altered genes such as TP53 and PIK3CA, which are predicted drivers for multiple cancer types. Along with known driver genes, our models also identify new driver genes such as PRKCA, SOX9, and PSMD4. Our multi-omic model labels both CNV and mutations with a more considerable contribution by CNV alterations. While predicting labels for genes mutated in multiple samples, we also label rare driver events occurring in as few as one sample. We also identify genes with dual roles within the same cancer type. Overall, PIVOT labels personalized driver genes as TSGs and OGs and also identified rare driver genes.

## 1 Introduction

Alterations in the genome drive the progression of cancer (Stratton et al., 2009). Mutations in certain genes, called driver genes, give cancer cells an added growth advantage (Vogelstein et al., 2013). These mutations, as well as other genomic changes, such as copy number variations (CNVs), accumulate as the tumor progresses. The genomic landscape of cancer is complex (Stratton et al., 2009; Vogelstein et al., 2013), with differences between cancer types in the number of mutations observed (Kandoth et al., 2013) or the mutation signatures (Alexandrov et al., 2013a; Alexandrov et al., 2013b). The genes mutated vary between cancer types and within subtypes of cancer. We now know that cells are heterogeneous within the same tumor, and heterogeneity confounds our understanding of the evolution of tumors (Greaves and Maley, 2012; Burrell et al., 2013). Mutational signatures vary in different cancer types (Alexandrov et al., 2013b). These specific patterns of mutations imply the need for cancer-specific driver mutation prediction tools. Both pan-cancer and tissue-specific identification of driver genes are essential for understanding cancer.

Various computational methods exist for identifying driver genes. Tools classify either mutations as driver events (Mao et al., 2013; Tokheim and Karchin, 2019; Banerjee et al., 2021), or the genes mutated as driver genes (Tokheim et al., 2016). Driver mutation prediction relies on the functional impact or neighborhood sequence. While some tools are specific to cancer (Tokheim and Karchin, 2019), other functional impact-based tools such as SIFT (Ng and Henikoff, 2003) or PolyPhen2 (Adzhubei et al., 2010) are not. Some tools are limited in their ability to predict only single-nucleotide variations, more specifically missense mutations (Tokheim and Karchin, 2019). Hence, using tools that predict driver mutations to predict personalized driver genes is limited in their scope.

Other tools exist that predict driver genes instead of mutations and use background mutation rate (BMR) or ratio-metric features for prediction. Computational methods using BMR such as MutSigCV (Lawrence et al., 2013) assume higher mutation rates in driver genes when compared with the background mutation rate. BMR-based methods are biased toward driver genes with high mutation frequency (Sudhakar et al., 2022). This shortcoming is overcome by ratio-metric features, which give importance to the functional impact of the mutation on the gene rather than the frequency of mutations (Davoli et al., 2013; Tokheim et al., 2016; Sudhakar et al., 2022). These methods are essential to identify most of the driver genes observed in a cohort but are elusive to rare driver genes.

Methods using the concept of mutual exclusivity of genes overcome the challenge by identifying a set of mutually exclusive genes in samples (Leiserson et al., 2013; Bokhari and Arodz, 2017). Somatic mutation data are used to identify sets of genes that improve coverage for the entire cohort. The mutual exclusivity approach is further improved by including biological knowledge as network information. QuaDMutNetEx (Bokhari and Arodz, 2017) uses biological interactions between proteins to find a set of mutually exclusive genes that perturb a pathway. Along with the mutual exclusivity of genes, the algorithm identifies a set of genes, which the algorithm can map onto the network to form a connected component. While this method may miss out on genes on different complementary pathways, the authors suggest iterating QuaDMutNetEx after excluding previously identified genes to help identify other essential driver genes. Network-based approaches may help identify low mutation frequency genes in a cohort by including biological interactions between proteins. While cohort-based methods help in understanding the biological mechanism of the disease, they are not very useful in a clinical setup. Additionally, a large number of samples are required for cohort-based methods to produce reliable results. They also cannot be used to find very rare driver genes.

While a large number of genomic events in the cancer genome are single-nucleotide variations (SNVs), other genomic rearrangements such as CNVs, gene fusions, and epigenomic changes are also known to occur. While the above-mentioned methods help identify many genes, many samples remain with no mutations in known driver genes (ICGC/TCGA Pan-Cancer Analysis of Whole Genomes Consortium, 2020). This implies that rare driver genes are missed out by cohort-based methods. BMR or ratio-metric methods also do not capture the effects of these methods. Another approach to identifying driver genes mutated at very low frequency in samples is to identify *personalized* driver genes, i.e., driver genes for individual samples rather than a cohort. Cohort-based studies rely on a large sample size to identify patterns consistent across samples, while identification of personalized driver genes is especially relevant for sub-types of cancers where large cohort studies are not possible or show very few mutations.

Identification of personalized driver genes helps identify actionable targets in patients without known driver mutations. The methods for the identification of personalized driver genes are based on unsupervised algorithms because we lack the ground truth. Instead, the methods use a network-based algorithm to identify perturbed pathways. The graph, along with somatic alterations and differential gene expression profile of the patient, is used to predict driver genes. DawnRank (Hou and Ma, 2014) and SCS (Guo et al., 2018) use a directed graph with loops for autoregulation. The directed graph is a collapsed network built using multiple protein–protein interaction (PPI) networks. DawnRank uses a modification of the Page-Rank algorithm to rank genes with downstream perturbed genes, while SCS uses Random Walker with Restart algorithm (RWR). Prodigy (Dinstag and Shamir, 2019) uses network as well as pathway data to identify genes, which deregulate a large number of pathways. The method uses a prize-collecting Steiner tree algorithm to find genes with SNV mutations. All methods identify rare driver genes compared with existing network-based methods for driver gene identification.

Network-based personalized driver gene tools integrate somatic mutation and gene expression data and identify genes using an unsupervised method. A subset of mutations, SNV, are used in PRODIGY though the method can be extended to other mutation types. Furthermore, the functional impact of mutations is ignored when mutation data are converted into presence/absence calls. Data ingested are limited to network, mutation, and expression data, though methods such as DawnRank also analyze CNV data. With many high-throughput multi-omic data available, the prediction of driver genes can be improved by including multi-omic data. Moreover, the expression data are included as differentially expressed genes (DEGs), calculated based on the cohort and not an individual sample. These methods rank the driver genes but do not classify genes as TSG or OG.

In this study, we define a machine learning (ML) classification problem to identify personalized driver genes and address the challenges. We define strategies for labeling genes as driver or neutral and identify the best model that classifies them. We employ features based on mutation, expression, CNV, and miRNA expression data and understand their contribution to classification. We finally build mutation, RNA, and multi-omic models to identify personalized diver genes and assign functional classes. We classify genes of three TCGA cancer cohorts as TSG or OG for individual samples and identify new driver genes.

## 2 Results

**FIGURE 1:**
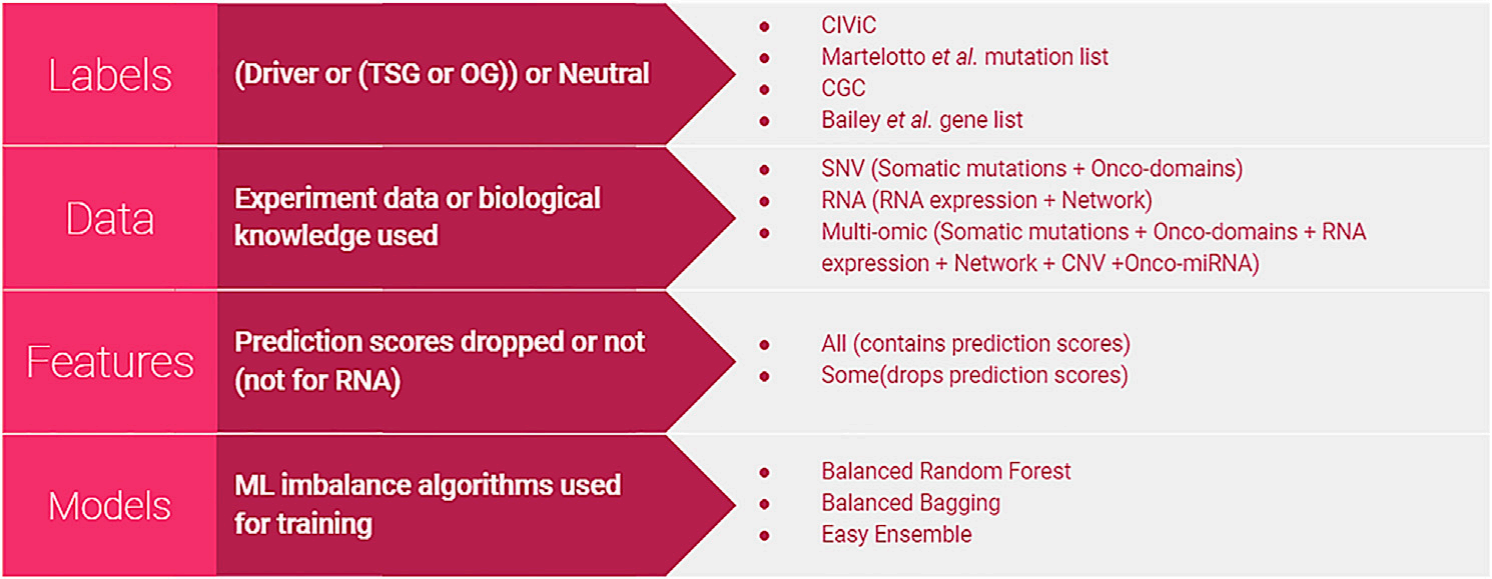
Different types of classifiers. Four different labeling strategies were used to label the altered genes. Models were built using SNV, RNA, or multi-omic data. The number of features used was varied for SNV and multi-omic data to either include or drop features based on prediction scores. For building classifiers, we used algorithms specific for imbalanced data as the number of neutral genes is far higher than TSGs or OGs. Classifiers are built using all combinations of labels, data, features, and models.

Our method, PIVOT (Personalized Identification of driVer OGs and TSGs), is the first ML-based supervised approach for identifying personalized driver genes to the best of our knowledge. Unlike previous methods that distinguish between driver and non-driver genes, PIVOT can also identify whether it functions as a TSG or OG in the specific cancer type. We build separate models using features extracted from different modalities of -omics data: SNV, gene expression, and multi-omics. The SNV model is trained on mutation data not limited to single-nucleotide variation but all mutation types. The multi-omic data integrates SNV, gene expression, CNV, miRNA data, and also network information. Figure 1 shows the different combinations of labeling strategies, data, feature sets, and models used. We further identify common cancer domains mutated frequently and find that only a subset of domains contribute to the models. We show that integrating network information with gene expression data improves the overall predictive power of the classification models. Last, we observe that our multi-omic model generates better predictions than the models based on SNV and gene expression data alone and can be used to predict novel TSGs and OGs in individual samples.

### 2.1 Gene-Based Labeling Helps Learn Better Models

One of the significant challenges in formulating a supervised classification problem for driver gene identification is the lack of a labeled gold standard dataset. We define four labeling strategies to assign labels and build models using the SNV data, two at the mutation level and two at the gene level. We used the CIViC (Griffith et al., 2017) database and a list of mutations published by Martelotto et al. (2014), to label genes containing known driver mutations as driver or TSG and OG. The models developed show high accuracy for BRCA. The precision and recall of driver classes are ≥0.95 for CIViC and ≥0.86 for Martelotto et al. list (Table 1). Although these models perform well, we use a highly curated list of less than 10 genes to train the model. While we could successfully label all the mutations from the BRCA dataset, both methods fail to label the COAD, LGG, and LUAD datasets. Notably, the number of samples in BRCA is twice the number of samples compared with other datasets (Supplementary Table S1). We conclude that although mutation labeling-based approaches successfully predict personalized driver genes, they are often limited in their ability to label mutations from all cancer types.

**TABLE 1:** Classification metrics for best SNV models.

Cancer type	Labeling strategy	Feature set	Accuracy	F1 score			Precision			Recall		
				Neutral	Driver		Neutral	Driver		Neutral	Driver	
					OG	TSG		OG	TSG		OG	TSG
BRCA	CIViC	All	0.99	1.00	0.98		1.00	0.95		0.99	1.00	
		Small	0.96	0.98	0.62		1.00	0.45		0.95	0.99	
	Martellotto *et al.*	All	0.86	0.91	0.86	0.96	0.97	0.95	0.98	0.86	0.78	0.95
		Small	0.83	0.90	0.49	0.97	1.00	0.36	1.00	0.82	0.81	0.95
	CGC	All	0.69	0.77	0.65	0.52	0.80	0.73	0.45	0.75	0.58	0.61
		Small	0.55	0.67	0.34	0.36	0.68	0.38	0.33	0.65	0.31	0.41
	Bailey *et al.*	All	0.89	0.94	0.84	0.69	0.96	0.78	0.66	0.92	0.90	0.72
		Small	0.75	0.83	0.43	0.61	0.95	0.30	0.50	0.74	0.77	0.79
COAD	CGC	All	0.61	0.73	0.44	0.35	0.72	0.55	0.32	0.73	0.36	0.39
		Small	0.67	0.78	0.30	0.38	0.72	0.95	0.41	0.85	0.18	0.36
	Bailey *et al.*	All	0.84	0.90	0.78	0.52	0.95	1.00	0.38	0.86	0.64	0.81
		Small	0.80	0.88	0.43	0.53	0.97	0.33	0.39	0.81	0.61	0.86
LGG	CGC	All	0.63	0.68	0.76	0.26	0.60	0.97	0.28	0.79	0.62	0.25
		Small	0.68	0.74	0.74	0.45	0.65	1.00	0.50	0.86	0.58	0.40
	Bailey *et al.*	All	0.87	0.87	0.93	0.67	0.86	1.00	0.57	0.89	0.86	0.81
		Small	0.86	0.88	0.90	0.74	0.87	0.98	0.68	0.89	0.84	0.81
LUAD	CGC	All	0.74	0.85	0.23	0.31	0.78	0.46	0.47	0.93	0.15	0.23
		Small	0.73	0.84	0.22	0.21	0.78	0.30	0.39	0.92	0.18	0.14
	Bailey *et al*.	All	0.88	0.94	0.71	0.34	0.97	1.00	0.23	0.90	0.55	0.61
		Small	0.82	0.90	0.69	0.23	0.98	0.88	0.14	0.83	0.57	0.64

Cancer Gene Census (CGC) (Sondka et al., 2018) is the gold standard database for known driver genes labeled as either TSG or OG. Similarly, the list of cancer-type specific genes published by Bailey et al. (2018) consists of predicted TSG and OG lists, which has been manually curated. Datasets labeled using CGC genes show accuracy ≤0.73 , where the neutral class mainly contributes to the score (Table 1). Given the heavy class imbalance, accuracy is not the best metric to judge a model. While the accuracy of the models is low compared with other labeling strategies, the F1-score (harmonic mean of precision and recall) is found to be ≤0.52 for TSGs and ≤0.76 for OGs. In some models, the F1 score of the TSG and OG is ≤0.20 . The list of genes in CGC is not cancer-type specific. Using CGC as the source to label cancer-type specific personalized driver genes results in models that perform well on the training set but not on the test dataset (Supplementary Table S1).

The best classification performances for identifying personalized driver genes were obtained using the labels derived from the list of TSGs and OGs published by Bailey et al. The method consistently performs well across all four cancer types with the best model accuracy of 0.89 (Table 1), though classification of TSGs is poorer than OGs. Across all feature sets used to build the best model, the accuracy is ≥0.80 except for the best model for BRCA data using a subset of the SNV features. The F1 score of TSGs is lower at 0.69 when compared with Martelotto et al. labels at 0.96. It is to be noted that the metrics cannot be compared directly between labeling strategies as the number of data-points differ. The cancer-specific genes obtained using Bailey et al. strategy resulted in a larger training dataset consisting of TSGs and OGs and consistently label all four cancer types, unlike mutation-based labeling approach (Supplementary Table S1). We conclude that the increase in the size of the training dataset using TSGs and OGs from Bailey et al., and the specificity of the genes used for training contribute to building better models for identifying personalized driver genes.

### 2.2 Mutation Data is Helpful for Identifying Personalized Genes

We used PIVOT on four TCGA datasets [Breast Cancer: BRCA (The Cancer Genome Atlas Research Network, 2012b), Colorectal Adenocarcinoma: COAD (The Cancer Genome Atlas Research Network, 2012a), Lower Grade Glioma: LGG (The Cancer Genome Atlas Research Network, 2015), and Lung Adenocarcinoma: LUAD (The Cancer Genome Atlas Research Network, 2014)] to predict genes as neutral or drivers. Depending on the labeling strategy, driver genes were further classified as TSG or OG. For BRCA, we observed the best accuracy for the model trained on CIViC labels using all SNV features (Supplementary Table S1). The F1 score for the best model was ≥0.98 for driver and neutral class. All ML algorithms, balanced bagging, balanced random forest, and Easy ensemble gave comparable results. Based on the F1 scores of TSG and OG for the best model in each labeling strategy, we built better models labeled using genes from Martelotto et al., followed by those from Bailey et al. and CGC. Different ML algorithms perform better based on the labeling strategy or the number of features used for prediction, though balanced bagging consistently performed best or close to best.

No data was labeled using CIViC or Martelotto et al. driver genes for the other three datasets (Supplementary Table S1). Models built on data labeled using TSGs and OGs published by Bailey et al. consistently performed better than models built on data labeled using CGC (Table 1). Furthermore, irrespective of the ML algorithm used for Bailey et al. labels, the recall on TSG is always higher than the precision. Predicting OGs using SNV is more straightforward than predicting TSG, as evident from the higher F1 score for OGs compared with the TSGs. The SNV features are primarily based on the scores given by mutation prediction tools that predict the damaging nature of the missense mutation. Since the training dataset mainly consists of missense mutations, it is intuitive that predicting OGs is easier. In general, models learnt on data labeled using Bailey et al. perform well using all the SNV features.

### 2.3 Mutation-Based Categorical Features are not Sufficient to Predict Driver Genes

SNV features are based on the functional impact of the mutation, domains mutated, and the prediction scores by various driver or mutation impact predicting tools. These tools cannot score all mutations and are hence dropped while training. We train our models using two feature sets, one that uses all features and the second that uses a subset of these features. The advantage of a smaller feature set is an increased number of training data. Since features with a large number of missing data are dropped, the number of data points dropped because of missing data reduces. The statistics for labeled data used for training and testing is available in Supplementary Table S1. Most SNV features consist of prediction scores and a corresponding categorical feature defining the impact of the mutation. For example, feature *SIFT*_*score*_ consists of a prediction score between 0 and 1, while the corresponding feature *SIFT*_*pred*_ is a categorical feature with “D” defining damaging and “N” for neutral mutations. We cannot impute missing values for *SIFT*_*score*_ and *SIFT*_*pred*_, as the given mutation type might be out of the scope of the tool. Hence in the smaller feature subset, we drop *SIFT*_*pred*_ feature and encode *SIFT*_*pred*_ as an ordinal feature with the lowest value for missing data (explained in *Methods*).

**FIGURE 2:**
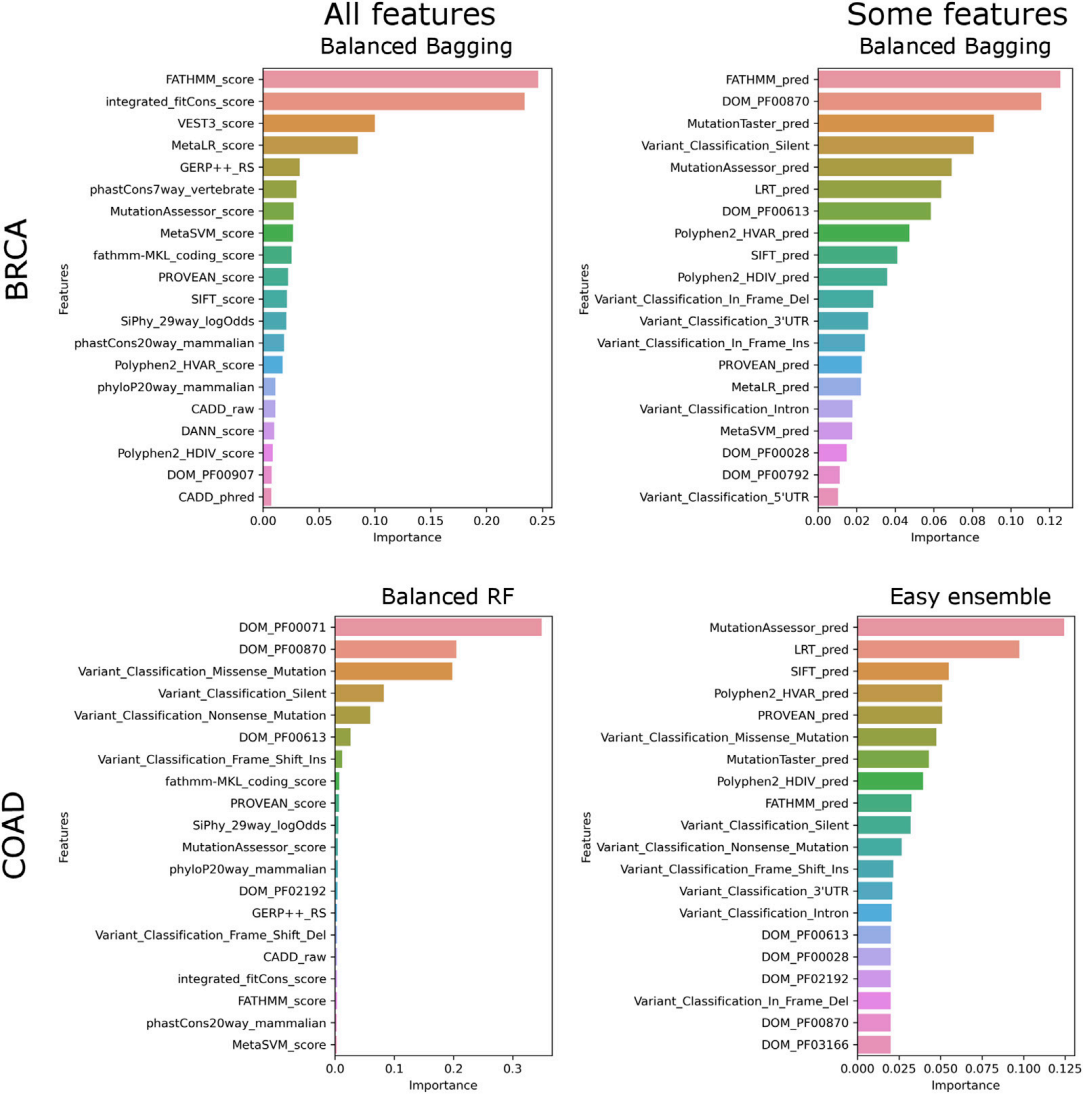
Top features contributing to models. The top 20 features and their contribution to the best model are plotted for “all” and “some” feature sets.

We ran the SNV models with two feature sets, “all” and “small,” where “small” consists of categorical features. It is to be noted that the number of features in the two feature sets vary between the datasets because of the difference in domains associated with a cancer type. In BRCA, irrespective of the labeling strategy, the models consistently perform better on “all” features (Table 1). Since only a subset of features is used, lower scores show that non-categorical features are essential for classification (Table 1). For other datasets labeled using Bailey et al. genes, we find that the “small” feature set performs equally well as compared to the “all” feature set (Table 1). Feature importance ranking shows that, unlike BRCA, for other datasets, categorical features rank high for both “all” and “small” feature sets, explaining the slight difference in F1 scores (Figure 2). While score-based features rank in the top 20 of all datasets, these features contribute more to BRCA (Supplementary Table S2) elucidating that the tools are better at predicting the functional effects of mutations in BRCA.

### 2.4 Cancer Domains Used for Predicting Driver Genes

We include onco-domain information into the SNV features. For each cancer type, we define features to capture the presence or absence of mutations in cancer-type-specific known onco-domains. Not all domains contribute equally to the classifier’s performance, with a majority having no contribution. We identify the top contributing domain in each cancer type. The subset of domains identified during SNV classification intersects with domain features contributing to multi-omic models (Table 2). BRCA identifies three domains: p53 DNA-binding domain, T-box, and cadherin-binding domain. T-box is a DNA-binding domain used in transcriptional activation/repression roles. TP53 is a known TSG pan-cancer, while cadherins are trans-membrane proteins used for adhesion. Similarly, we identify the top domains for all four cancer types. We find that the p53 DNA-binding domain is the only domain feature identified for all cancer types by SNV and multi-omic models. The individual contribution of domains to SNV and multi-omic models is listed in Supplementary Table S3.

**TABLE 2:** Domains contributing to models for the cancer type.

Cancer type	Domain
BRCA	PF00870: P53 DNA-binding domain
	PF00907: T-box
	PF00028: Cadherin domain
COAD	PF00071: Ras family
	PF00870: P53 DNA-binding domain
	PF03166: MH2 domain
	PF00001: 7 transmembrane receptor (rhodopsin family)
	PF00028: Cadherin domain
	PF00613: Phosphoinositide 3-kinase family, accessory domain (PIK domain)
	PF00520: Ion transport protein
LGG	PF00180: Isocitrate/isopropylmalate dehydrogenase
	PF00870: P53 DNA-binding domain
	PF00505: HMG (high mobility group) box
	PF00757: Furin-like cysteine-rich region
	PF07710: P53 tetramerization motif
	PF10409: C2 domain of PTEN tumor-suppressor protein
LUAD	PF00071: Ras family
	PF00431: CUB domain
	PF00084: Sushi repeat (SCR repeat)
	PF00870: P53 DNA-binding domain
	PF08441: Integrin alpha
	PF00057: Low-density lipoprotein receptor domain class A
	PF00028: Cadherin domain
	PF00001: 7 transmembrane receptor (rhodopsin family)
	PF02210: Laminin G domain
	PF10565: N-methyl D-aspartate receptor 2B3 C-terminus
	PF01094: Receptor family ligand binding region
	PF00041: Fibronectin type III domain
	PF07710: P53 tetramerization motif
	PF00754: F5/8 type C domain
	PF07679: Immunoglobulin I-set domain
	PF01007: Inward rectifier potassium channel transmembrane domain

### 2.5 Expression and Network-Based Features Improve Prediction Accuracy

**FIGURE 3:**
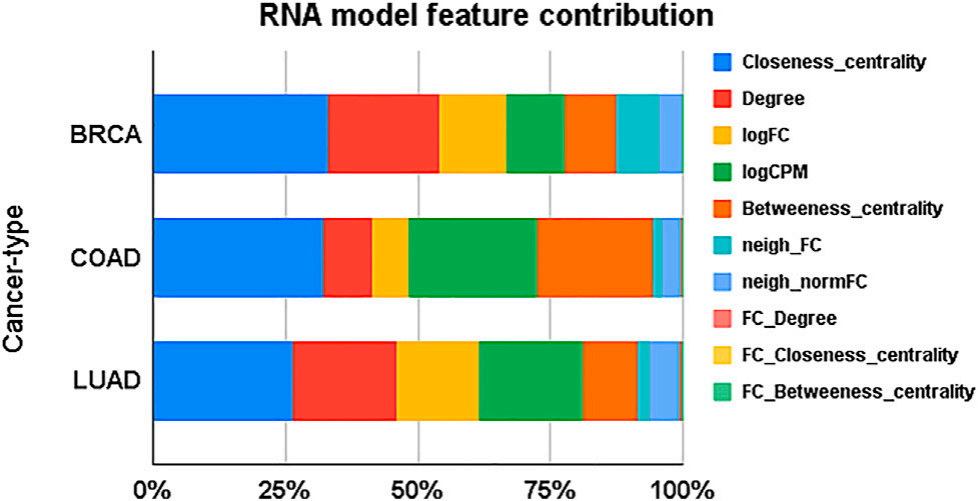
Expression and network-based features contributing to models. For each cancer type, the average contribution of a feature across all models is plotted.

While mutation data helps classify genes into TSGs and OGs, there is scope for improvement. We used expression data and PPI networks for generating features and predicting personalized driver genes. We built models for BRCA, COAD, and LUAD cancer types using all feasible labeling strategies. In contrast to SNV models, we find ≥96% accuracy across the best models for all three cancer types (Supplementary Table S4). The F1 score of OG and TSG is ≥94% across all models, irrespective of the labels (Table 3). An analysis of the features revealed network properties of genes such as closeness centrality and degree are the significant contributors to the identification of TSGs and OGs (Figure 3). While the RNA expression-based features logFC and logCPM contribute, they are not sufficient for classification. Network and RNA expression features improve the classification accuracy above SNV features.

**TABLE 3:** Classification metrics for best RNA models.

Cancer-type	Labeling strategy	Accuracy	F1 score			Precision			Recall		
			Neutral	Driver		Neutral	Driver		Neutral	Driver	
				OG	TSG		OG	TSG		OG	TSG
BRCA	CIViC	1.00	1.00	0.96		1.00	0.92		1.00	1.00	
	Martellotto *et al.*	0.96	0.98	0.94	0.96	1.00	0.91	1.00	0.96	0.97	0.92
	CGC	0.99	0.99	0.98	0.98	0.99	0.99	0.98	0.99	0.98	0.99
	Bailey *et al.*	0.99	1.00	1.00	0.98	1.00	1.00	0.97	0.99	1.00	1.00
COAD	CGC	0.98	0.99	0.98	0.97	0.99	0.96	0.98	0.99	0.99	0.97
	Bailey *et al.*	0.99	0.99	0.99	0.97	1.00	0.98	0.94	0.99	1.00	1.00
LUAD	CGC	0.99	0.99	0.98	0.98	1.00	0.97	0.98	0.99	0.98	0.98
	Bailey *et al.*	0.99	1.00	0.97	0.94	1.00	0.96	0.89	0.99	0.98	1.00

### 2.6 Multi-Omic Data Generate the Best Classification Performances

**FIGURE 4:**
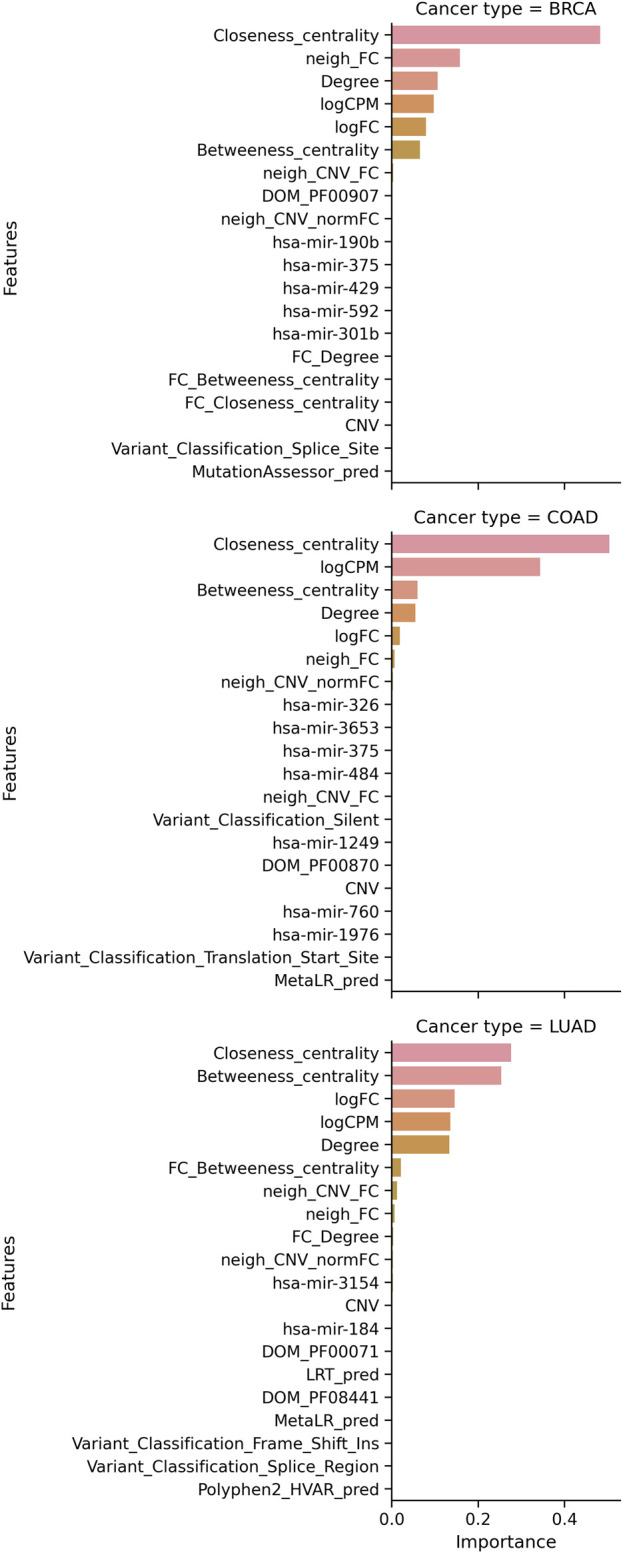
Multi-omic features contributing to the best model for cancer type. For each cancer type, the contribution of the top 20 features is plotted.

The SNV and RNA models are sufficient to predict the driver genes, but including both improves the prediction. We used features extracted from SNV, RNA features, CNV, and miRNA data to build the multi-omic models. The best prediction model uses Balanced Bagging across all cancer types using Bailey et al. genes for labeling (Supplementary Table S5). Unlike the SNV models, the smaller feature subset performs better across all cancer types for Bailey et al. labels (Table 4). The model accuracy is 1.0 for BRCA, with an F1 score of 0.99 for OG and 0.97 for TSG. For COAD, we observe an accuracy of 0.99 with an F1 score of 0.97 and 0.94 for OG and TSG, respectively. Similarly, for the LUAD cancer type, we achieve an accuracy of 0.99 and an F1 score of 0.91 and 0.90 for OG and TSG, respectively. Randomizing the training labels shows a substantial drop in the classification metrics from the F1 score >0.90 to ≤0.11 for TSG and OG, showing that our models do not overfit. Detailed classification metrics for all multi-omic models and randomized labels are available in Supplementary Table S5. Feature importance shows network and RNA expression are top-ranking features (Figure 4) among other multi-omic features (Supplementary Table S2). We generated two random networks: 1) by node label randomization and 2) degree maintaining randomization. Both randomized networks lead to decreases in the F1 score suggesting that the network includes biological knowledge important for identifying personalized driver genes better. Along with CNV features, we also find some miRNA features contributing to the overall classification performance (Supplementary Table S9).

**TABLE 4:** Classification metrics for best multi-omic models.

Cancer-type	Labeling strategy	Feature set	Accuracy	F1 score			Precision			Recall		
				Neutral	Driver		Neutral	Driver		Neutral	Driver	
					OG	TSG		OG	TSG		OG	TSG
BRCA	CIViC	All	1.00	1	0.99		1	0.99		1.00	1.00	
		Small	1.00	1	0.97		1.00	0.93		1.00	1.00	
	Martellotto *et al.*	All	0.96	0.98	0.97	0.87	1.00	1.00	0.80	0.96	0.95	0.96
		Small	0.96	0.98	0.83	0.95	1.00	0.73	0.95	0.96	0.95	0.96
	CGC	All	0.89	0.92	0.89	0.80	0.92	0.92	0.80	0.93	0.87	0.81
		Small	0.89	0.92	0.89	0.80	0.92	0.92	0.80	0.93	0.87	0.81
	Bailey *et al.*	All	0.96	0.97	0.95	0.86	1.00	0.91	0.76	0.95	1.00	0.98
		Small	1.00	1.00	0.99	0.97	1.00	0.97	0.95	1.00	1.00	0.99
COAD	CGC	All	0.85	0.91	0.73	0.74	0.91	0.69	0.76	0.91	0.76	0.72
		Small	0.94	0.95	0.86	0.92	0.94	0.86	0.96	0.97	0.87	0.88
	Bailey *et al.*	All	0.97	0.98	0.90	0.90	1.00	0.82	0.83	0.97	1.00	0.98
		Small	0.99	0.99	0.97	0.94	1.00	0.94	0.89	0.99	1.00	1.00
LUAD	CGC	All	0.91	0.94	0.81	0.78	0.96	0.78	0.73	0.93	0.84	0.84
		Small	0.95	0.97	0.89	0.93	0.97	0.85	0.94	0.96	0.92	0.92
	Bailey *et al.*	All	0.98	0.99	0.89	0.81	1.00	0.80	0.68	0.98	1.00	0.99
		Small	0.99	0.99	0.91	0.90	1.00	0.85	0.82	0.99	0.99	1.00

### 2.7 Rare Driver Genes Predicted Across Cancer Types

We used the multi-omic model to predict TSGs and OGs for BRCA, COAD, and LUAD cancer types. Most samples predicted at least one TSG or OG. Out of 984 samples, we generated features for 972 samples and identified driver genes in 963. Similarly, only three samples in COAD predicted no driver genes and the number was larger at 62 for LUAD. Surprisingly, the number of unique genes predicted in each cancer type was large. In BRCA a total of 1,342 unique genes were identified, followed by 1,155 and 1,152 in LUAD and COAD, respectively. The distribution of genes identified across samples was similar in all cancer types with most samples with <100 genes identified as drivers (Supplementary Figures S43, S49, S56). A large number of genes identified, consist of mutation as well as CNV alterations with CNVs contributing with as high as 100 genes altered in one sample (Supplementary Figures S44, S50, S57). In contrast, most samples had an average of 10 mutations identified as driver genes (Supplementary Figures S45, S51, S58) as previously reported in the literature (Vogelstein et al., 2013).

**FIGURE 5:**
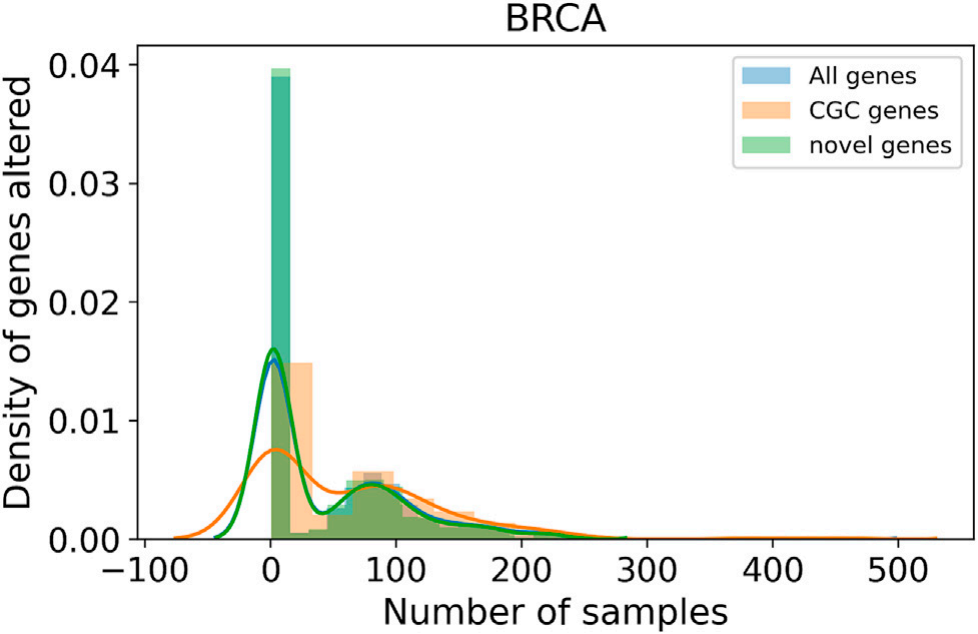
Distribution of samples for genes predicted as a driver. The *x*-axis represents the number of BRCA samples a gene is identified as driver. The *y*-axis shows the density of genes identified in *n* samples. The plot shows a bi-modal distribution with genes mutated in many samples as well as <20 samples.

Genes identified across a large number of samples are well-known driver genes. The top 10 genes in each cancer type are listed in Table 5. Comparison with the list of genes in CGC showed an overlap of 228, 169, and 188 genes for BRCA, COAD, and LUAD, respectively. The list of top genes also consists of genes, such as *PRKCA* (Kelemen et al., 2009; Lin et al., 2017; Pham et al., 2017; Beetch et al., 2021), *SOX9* (Lü et al., 2008; Carrasco-Garcia et al., 2016; Lizarraga et al., 2019), and *NFKBIA* (Furukawa et al., 2013), that are not present in CGC but found in the literature for their role in respective cancer types. We also identify a large number of rare driver genes identified in as few as one sample in BRCA (Supplementary Table S6), COAD (Supplementary Table S7), and LUAD (Supplementary Table S8). In BRCA (Figure 5) and LUAD (Supplementary Figure S62), we observe a bimodal distribution with a large number of genes identified in a large number of samples along with genes that are mutated in less than 10 samples. Genes predicted in COAD cancer type on the other hand show consensus in a few samples (Supplementary Figure S55). The distribution is similar for the known driver gene listed in CGC and predicted by the model, indicating that genes identified in a few samples are not false positives. We find genes, such as *TK1* in LUAD (Xu et al., 2012; Malvi et al., 2019) and *ELAVL1* in BRCA (Chou et al., 2015; Liu et al., 2019), predicted in only one sample but known to have a role in respective cancer types. Patients with high expression of *TK1* showed higher stromal invasion grade and poor survival in lung adenocarcinoma. A knockdown of the gene with shRNAs led to reduced growth and metastasis in cell lines and mice models. Silencing *ELAVL1* directly or indirectly inhibited the growth of breast cancer *in vitro* and *in vivo* by interacting with other proteins such as *β*-catenin, *PKD*1, *PKD2*, and *PKD3*. Moreover, treatment with Quercetin in triple-negative breast cancer inhibited cytoplasmc *ELAVL1*, which directly affects adhesion and migration of cells (Umar et al., 2021). The list of rare predicted genes provides potential genes to target and study to understand their role in the progression of tumors.

**TABLE 5:** Top genes identified for all cancer types. The genes are listed along with the number of samples they were labeled TSGs or OGs. Their presence or absence in CGC is also given.

BRCA			COAD			LUAD		
Gene	Samples	CGC	Gene	Samples	CGC	Gene	Samples	CGC
PIK3CA	453	Yes	APC	408	Yes	TP53	268	Yes
TP53	383	Yes	TP53	212	Yes	KRAS	186	Yes
PRKCA	239	No	KRAS	153	Yes	STK11	135	Yes
MYC	235	Yes	PIK3CA	122	Yes	CDKN2A	133	Yes
GATA3	230	Yes	ARID1A	87	Yes	EGFR	112	Yes
PSMC5	229	No	FBXW7	86	Yes	KEAP1	111	Yes
IKBKB	228	Yes	SOX9	81	No	NF1	105	Yes
PSMD12	228	No	PRKDC	73	No	PSMD4	105	No
MAP2K6	226	No	SMAD4	72	Yes	NFKBIA	103	No
RPS6KB1	225	No	MTOR	60	Yes	MYC	99	Yes

### 2.8 Genes with Dual Roles in Different Samples Identified

We observed that the number of TSGs in a given cancer type is always greater than OGs (Supplementary Figures S47, S48, S53, S54, S60, S61). In LUAD, 1,030 TSGs and 195 OGs were predicted, while the number was 991 and 798 in BRCA and 938 and 266 in COAD. It is interesting to note that some genes are labeled differently in different samples, with as many as 447 genes in BRCA, 70 in LUAD, and 52 in COAD. Genes labeled as both TSG and OG suggest genes behaving differently in not only cancer types but within subtypes of cancer. *JAK1* was one such gene identified as both TSG and OG in BRCA and COAD cancer types. *JAK1* is a signal transducer and activates the JAK/STAT pathway. It has been shown to be consistently active leading to cell survival in colon cancer (An et al., 2014) and lower survival rates (Tang et al., 2018). Similarly, *JAK1* is activated by PRLR signaling in a subset of breast cancers (Neilson et al., 2007), while underexpression of *JAK1* is needed for the invasion of immune response (Albacker et al., 2017; Chen et al., 2019). The role of *JAK1* is highly dependent on the cell conditions and can vary across different cancer subtypes (Yeh et al., 2007). *GNA11* is another gene that was classified as both a TSG and an OG in all three cancer types. The role of *GNA11* as an oncogene and the occurrence of mutations in tumor samples are well studied. But, unlike an oncogene downregulation of *GNA11* was observed in human breast cancers. Genes identified with multiple labels might be used to understand diverging roles of a gene in cancer.

### 2.9 Comparison of Personalized Driver Gene Tools

**FIGURE 6:**
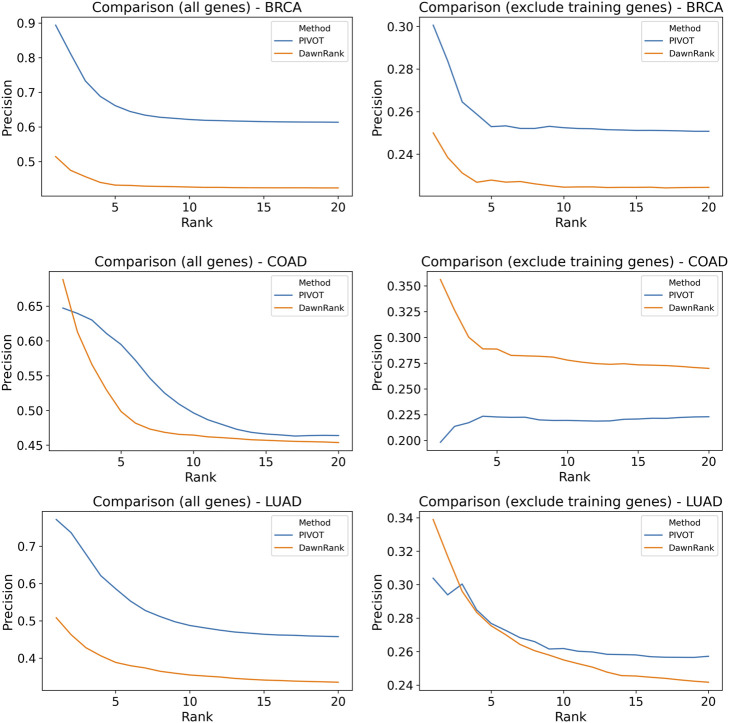
Comparison of precision of personalized driver genes. Average precision of DawnRank and PRODIGY for varying ranks are plotted for BRCA, COAD, and LUAD. The plots of the left include predictions on all genes for both methods. The plots on the right drop genes used for training PRODIGY for both methods before ranking. The precision is calculated for CGC gene list.

We compare PIVOT with DawnRank, another personalized driver gene prediction tool. We compare the predictions only for mutated genes and find consensus for 1,315, 1,628, and 1,281 sample-gene pair combinations for BRCA, COAD, and LUAD, respectively. We rank all the genes for each sample and calculate the precision of the predictions for each sample. The average precision across samples is plotted in Figure 6 for the top 20 ranked genes. We find that, for the entire data set, PIVOT predicts better than DawnRank across all cancer types. For COAD, overall PIVOT performs better though for higher ranks the average precision for DawnRank is greater than PIVOT. PIVOT, unlike DawnRank, is trained on known genes, and to avoid any bias, we ignore the genes trained on. After excluding the training genes, we find that for BRCA, PIVOT performs better. For COAD, DawnRank has higher precision than PIVOT when the training genes are excluded. The lower precision on excluding training genes means PIVOT fails to identify some CGC genes. The precision for DawnRank is higher for the first couple of ranks but PIVOT performs equally well or better. It is to be noted that the precision is calculated using CGC genes and the analysis of labeling strategies showed that the CGC gene list is not specific to cancer types. Given the lack of any gold standard data for personalized genes, comparison with CGC genes is commonly used to evaluate a tool. The comparison shows that PIVOT identifies genes trained on, as well as other possible driver genes not previously trained on.

## 3 Discussion

Identifying driver genes is still a challenging problem with new tools being developed to identify driver genes. Tumors are highly heterogeneous, even within the same cancer type. While some genes such as *TP53* (Vogelstein et al., 2000; Petitjean et al., 2007) are mutated in a significant fraction of tumors, a small fraction of patients with no mutations in known cancer driver genes exist (ICGC/TCGA Pan-Cancer Analysis of Whole Genomes Consortium, 2020). The identification of less frequently mutated genes in a new set of samples will require multiple samples with mutations in these genes. Personalized driver tools can predict driver genes in a single new sample once trained on a large number of samples. The treatment strategy is geared toward personalized medicine based on the presence of prognostic markers to optimize for patient’s recovery (Verma, 2012). Targeted therapy is given based on the genomic alterations in known driver genes (Amado et al., 2008; Ross et al., 2009; Cho et al., 2012). Identification of personalized driver genes is the first step to push the field of personalized medicine further. Personalized genes will not only help identify potential targets but identify genes mutated in only a small subset of samples.

We developed a machine learning model that employs multi-omic data to identify personalized driver genes. To the best of our knowledge, this study is the first supervised ML approach for identifying personalized driver genes. Our models label the genes based on their functionality as TSGs and OGs, another first in the field of personalized driver genes. We employ the SNV, RNA, CNV, miRNA, network, and Pfam (Mistry et al., 2021) domains to build the models. Not all data from multiple omics will be available at all times. Hence, we build the SNV and RNA models when only SNV or RNA data are available. Many driver genes are also hub genes, and integrating STRING network (Szklarczyk et al., 2019) information greatly improves the prediction as observed by our RNA and multi-omic models. All three node-based network features rank consistently high. While driver genes are known to have a high degree, the distribution of the degree of predicted genes shows genes with a low degree are also predicted and the model is not biased toward genes with a high degree (Supplementary Figures S46, S52, S59). Analysis of feature contribution of SNV features shows that the most contributing feature for BRCA data is the FATHMM score, and when the score is dropped, the FATHHM prediction category is important. For other cancer types, the contribution of tools varies. The feature contribution of prediction scores can be used to identify tools that best predict the functional effect of the mutations in different cancer types.

Supervised classification models require labels to train the models. The field of personalized driver gene prediction is largely unexplored, and no gold standards are available to validate the results. Formulation of the supervised problem requires a label. The mutation-based labeling methods consist of highly curated mutations and genes, which build models with high accuracy for only one cancer type (BRCA). While models built on labeled mutations score high on classification metrics, their ability to predict genes not observed may vary. Furthermore, ML algorithms predict consistently only when trained on large datasets that mimic all driver genes. We increased the training data by employing gene-based labeling methods and dropping SNV features with large missing data. Our final multi-omic models built on fewer features and many cancer-type specific driver genes perform the best.

The lack of gold standard further makes comparison difficult among different methods. Most driver gene lists are either pan-cancer or cancer-type specific. Comparison for personalized driver genes requires a list of driver genes specific for a patient. In the absence of any ground truth most methods use CGC gene list to compare predictions. While building models we show the performance is poorest on CGC genes as the list of genes is not specific to cancer type. There is a need for establishing gold-standard for personalized driver genes to further the field.

Compared with previous methods that employ only mutation and expression data, we include known biological knowledge regarding domains and onco-miRNA expression. Furthermore, the previous network-based unsupervised methods consider the presence or absence of mutations in the gene. The functional impact of the mutation is lost in the compression. We use mutation type and prediction scores by multiple tools, and we observe functional impact-based features ranking high in the absence of network or RNA data. Furthermore, the methods use expression data to identify DEGs to map onto the network. The method to identify DEGs is based on cohort, making it difficult to use in a clinical setting. We identify DEGs for an individual sample based on pre-computed values of biological variation for the cancer type. Any new sample can be processed to feature and predict TSG and OG.

Our method has its limitation commonly observed with ML-based models. ML algorithms assume the training data covers all possible driver genes and the features capture all the required information to predict the genes. Given the lack of experimental data on personalized driver genes, we assume known driver mutations and all mutations in the curated driver gene list are drivers in the observed tumor. Furthermore, it is to be noted that PIVOT may also produce false positives along with true personalized driver genes. Our tool generates reasonable candidate driver genes in a personalized fashion, which help shortlist genes for experimental studies to understand the progression of cancer. PIVOT uses logCPM values from expression data, which is biased to genes with longer length. Calculating transcripts per million (TPM) might be a better feature as it is normalized for gene length. Including new features that improve the prediction while not increasing computation cost are potential directions in this field. We use network data from STRING (Szklarczyk et al., 2019), and other networks such as Reactome can be used instead of or in combination. Currently, we use miRNA expression of known cancer miRNAs. We can generate gene-specific features to capture miRNA and mRNA interaction. Furthermore, we can include scores from other personalized driver prediction tools as features to develop an ensemble model to improve predictions. The research space for the identification of personalized driver genes is mostly under-explored.

## 4 Conclusion

In this study, we make three significant contributions. First, we define the identification of personalized drivers as a supervised problem by defining and studying various labeling strategies. We conclude that the best labeling strategy is using gene-based labeling that is cancer-specific. Second, we build multiple models on mutation, RNA, and multi-omic features to identify the contribution of individual omic-based features. We show network-based and expression features contribute the most to models, with the multi-omic models performing the best. In case of missing omic data, mutation or RNA models can be used for predicting driver genes. Lastly, our models can label genes as TSG and OG in a tumor. The functional labeling of genes is helpful for the identification of potential treatment strategies. Our method, PIVOT, is capable of predicting TSG and OG for individual samples, with multiple models available for predicting based on the availability of data.

## 5 Methods

**FIGURE 7:**
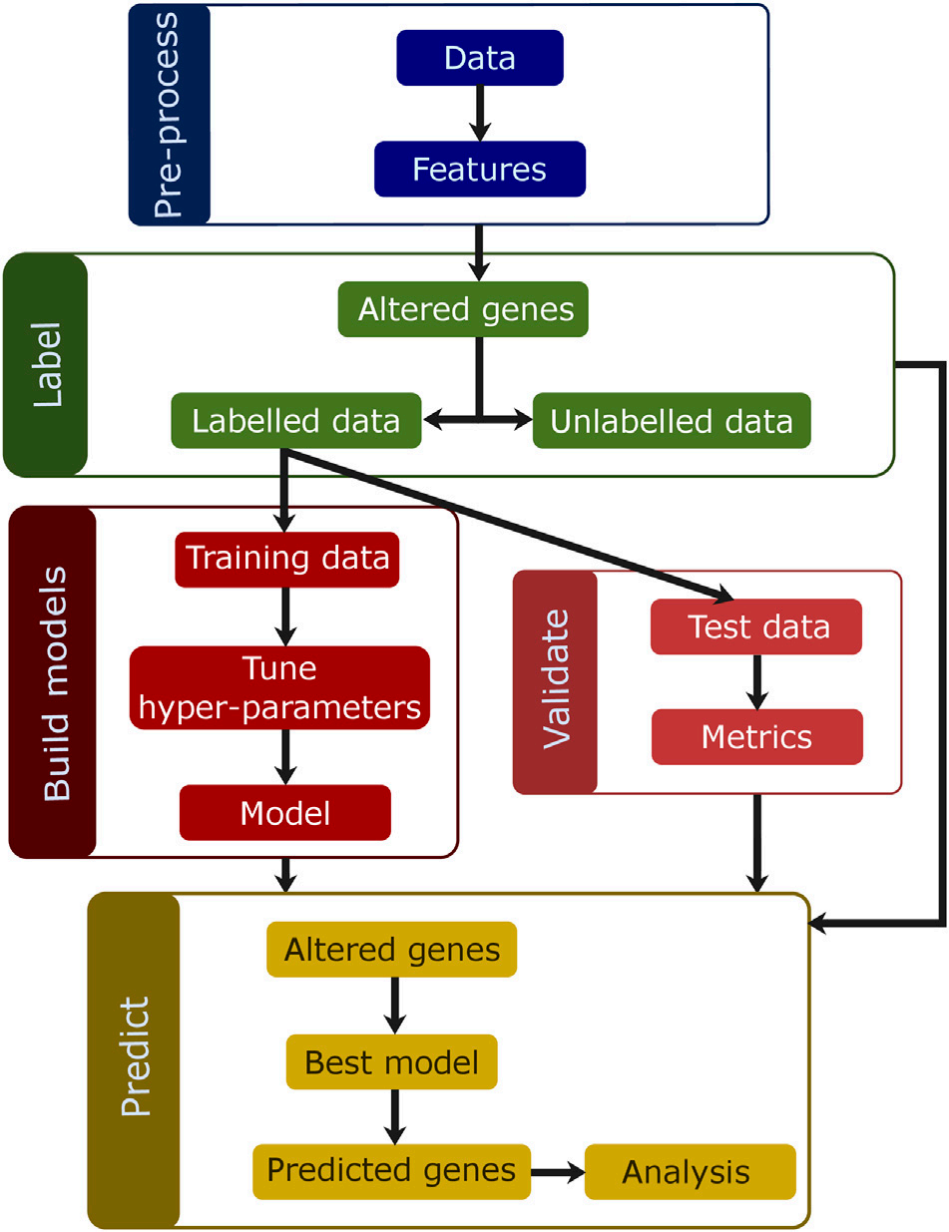
Overview of the methods. The data are preprocessed to generate features to be used for classification. The data are then labeled using one of the four labeling strategies, and samples are split into train and test. The training data are used to tune and build models. The metrics are calculated for the predictions made on test data and used to select the best model. The best model is then used to predict TSGs and OGs on the altered genes for all samples.

The models for the identification of personalized TSGs and OGs are built using different modalities of data, feature sets, labeling strategies and imbalance algorithms. The data are labeled using four different strategies. The data are split into training and test and models are tuned using cross-validation on the training data. The best models are selected using metrics on the test data. The overview of model building is shown in Figure 7.

### 5.1 Data

The TCGA data were downloaded from GDC for the four cancer types, BRCA, COAD, LGG, and LUAD. For each cancer type the mutation (downloaded BRCA on October 4, 2021, rest on November 17, 2021), expression (downloaded BRCA on October 4, 2021, rest on December 3, 2021), CNV, and miRNA data were downloaded (downloaded BRCA on October 4, 2021, rest on December 7, 2021). The mutation file was downloaded as a maf file generated using Mutect2. The data were annotated using ANNOVAR (Wang et al., 2010) and processed to include domain-based features. The expression data from RNA-sequencing experiments alone were downloaded for BRCA, COAD, and LUAD as raw HT-seq counts. The data were processed using edgeR (Robinson et al., 2009; McCarthy et al., 2012; Chen et al., 2016) to obtain DEGs for each patient. For BRCA, COAD, and LUAD we downloaded CNV data as a GISTIC gene-level copy number score. For labeling genes, driver genes lists are downloaded from the CIViC database, Cancer Gene Census (CGC), Martelotto et al. and Bailey et al. The list of neutral genes is also published by Bailey et al. included in the GitHub PIVOT folder under folder data, subfolder driver. A list of onco-domains, miRNA, associated with cancer type is also included in the GitHub PIVOT folder data. The STRING v11.5 (Szklarczyk et al., 2019) database was downloaded for the protein–protein interaction (PPI) network. Only edges with experimental or database scores above 700 were retained. The network was processed to generate degree, closeness centrality, betweenness centrality, and all neighbors for a gene. The data are stored as pickle files and accessible via the GitHub PIVOT repository.

### 5.2 Labeling Genes

**FIGURE 8:**
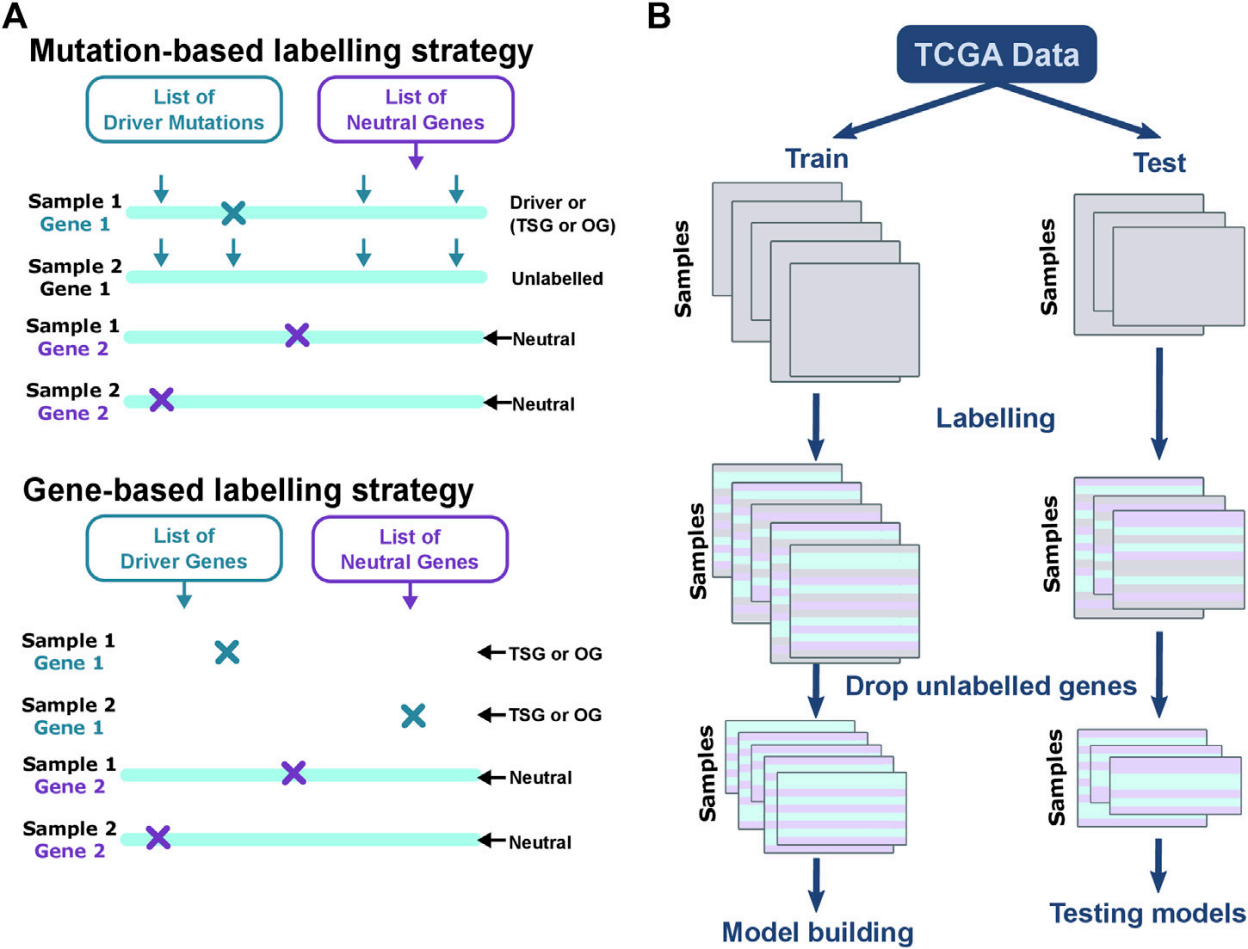
Labeling data for training and testing. The figure explains the differences between labeling strategies and the steps for training and test split. **(A)** For mutation-based strategies, the gene and location of the mutation are both considered. The arrows indicate the locations of driver mutations in a gene. The mutation in Gene 1 coincides with known driver mutation and the gene is labeled as “Driver” in Sample 1. Mutation in Gene 1 of Sample 2 is not a known driver mutation. Since the mutation locations differ between the first and second samples shown here, Gene 1 in Sample 2 is unlabeled. For gene-based methods, irrespective of the location of mutation, Gene 1 is labeled “Driver” in Samples 1 and 2. All lists of neutral genes if altered in a sample are labeled as neutral. Gene-based methods can be used to label mutation and CNV alterations. Altered genes, if present in the list of driver mutations or driver genes, are labeled as “Driver” if the labeling strategy is CIViC or “TSG” or “OG” for the rest. **(B)** The samples are split into training and test in the ratio 70:30. The number of mutations may vary in samples, and is captured by the varying shapes of the data boxes. Each row represents a sample—gene pair and the columns represent the features. The genes for each sample are labeled, and used for building models and evaluation.

Driver genes are defined as a gene that contains driver mutations or is expressed aberrantly such that it confers a selective growth advantage (Vogelstein et al., 2013). We label all mutations and CNV changes based on the previously published driver and neutral gene lists. We use four driver gene lists to label the data, where two are based on mutations, and two are based on genes (Figure 8A). The mutation-based strategies include CIViC database, and Martelotto et al. After considering threshold of >15 for the evidence scores, CIViC data contain 342 unique mutations which span 155 genes. For each sample and individual gene, the mutation location and nucleotide alteration were searched in the CIViC database and if found the gene was labeled “Driver” for the sample. Similarly, the mutation location and nucleotide alteration were used to label genes using Martelotto et al. as “TSG” or “OG.” The Martelotto et al. gene list comprised 543 unique mutations in six oncogenes, and 2,947 alterations in three tumor suppressors. The gene-based labeling strategies include CGC and Bailey et al. CGC comprises 723 pan-cancer driver genes, out of which 182 and 106 genes were labeled as “TSG” and “OG,” respectively. Genes with multiple roles in cancer were excluded. Any alteration in gene, including mutations and CNVs, in the given sample was labeled as “TSG” or “OG” if present in the CGC list. Unlike the previous methods for labeling, where the list of genes remains the same irrespective of the cancer type, the Bailey et al. gene list varies for cancer types. Similar to the CGC labeling strategies, the Bailey et al. gene list is used to label mutations and CNV alterations. The total numbers of genes used for labeling are 20, 29, 24, and 20 for COAD, BRCA, LGG, and LUAD, respectively. Genes tagged as “tsg” or “possible tsg” are labeled “TSG,” and genes tagged as “oncogene” or “possible oncogene” are labeled “OG.” The lists of TSGs and OGs vary for cancer types as listed: COAD—12 TSGs and 8 OGs, BRCA—19 TSGs and 9 OGs, LGG—12 TSG and 12 OG, and LUAD—13 TSG 6 OG. We used the list of 488 genes published by Bailey et al. to label neutral genes, which remain constant across labeling strategies and cancer types. For each sample, all genes altered (mutation/CNV) are labeled as “TSG,” “OG,” or “Neutral,” based on the labeling strategy. Unlike cohort-based methods where labels are predicted for genes for the entire cohort, our method predicts labels for a patient–gene pair making the method personalized. It is to be noted that while the number of genes are few, since the training data consider each sample–gene as a single data point and not a gene; the size of the training dataset is larger than the number of genes. Genes not labeled as neutral or driver or TSG or OG are unlabeled, and not included during training or testing. The unlabeled data are used for predicting new and rare driver genes.

### 5.3 Feature Generation

#### 5.3.1 Mutational Features

For SNV features, the mutation data were annotated using ANNOVAR (downloaded on March 25th, 2018) (Wang et al., 2010) to include prediction scores for various tools. The mutation type was one-hot encoded. All other categorical predicted features were converted into ordinal categories, where missing data were given value 0. The list of domains (Hashemi et al., 2017) was processed to retrieve Pfam ids. All onco-domains for the given cancer type were used as features, and the domain feature was assigned value one if the mutation is associated with the domain. The mutation and list of domains associated are given in the mutation maf file. The number of features vary in each cancer type and are dependent on the number of cancer domains identified.

#### 5.3.2 Expression Features

For RNA features, the data were processed for each sample to generate a list of DEGs using edgeR (v3.32.1). Differential genes are usually reported for a cohort. It is advisable to have more than one sample in each condition. In a clinical setting, multiple samples may not be available. We generate DEGs for each sample. Not all samples contain paired adjacent normal for a cancer type. First, we calculate the common biological coefficient of variation (bcv) for all tumor samples against normal—this is the coefficient of variation with which the (unknown) true abundance of the gene varies between replicate RNA samples (McCarthy et al., 2012). It represents the CV that would remain between biological replicates if sequencing depth could be increased indefinitely. Estimating BCV is required to avoid false discoveries. We then group all normal samples and run DEG against all tumor samples individually. The previously calculated bcv is specified to the fit function when only one tumor sample is used to calculate the DEGs. The common bcv calculated for a cancer type can be used for the identification of DEGs for future samples and saved as individual files. The logFC and logCPM values are used as features. The logFC refers to the log fold change, which is the ratio of the difference in gene expressed in the tumor sample in comparison with the normal samples. The logCPM value quantifies the expression of gene as log counts per million. Other features include node properties degree, closeness centrality, and betweenness centrality. Another three features are produced, multiplying the logFC to the node properties. The differential expression of neighbors is captured by features *neigh_FC* and *neigh_normFC*. For a given gene, *neigh_FC* is calculated as the sum of logFC of all neighbors of the gene, with fold change >2 or <−2 . *neigh_normFC* is normalized for the number of genes differentially expressed in the neighborhood.

#### 5.3.3 Multi-Omic Features

Multi-omic features concatenate the SNV, RNA features, CNV, and miRNA data. The effect of CNV on neighborhood is calculated by *neigh_CNV_FC* and *neigh_CNV_normFC* similar to RNA features. All genes with copy number variation ≠ 0, the sum of logFC values >2 or <−2 of all neighboring genes defines *neigh_CNV_FC*. The neighborhood is defined by all genes that can be accessed with ≤n hops. We consider *n* = 1 for this analysis. The list of miRNA associated with cancer type was downloaded from OncomiR *Cancer* Database [OCMD; Sarver et al. (2018)] and OncomiR (Wong et al., 2018) databases and the intersection of both databases was used for the analysis. Expression of the known oncogenic miRNAs was used as features for predicting.

### 5.4 Classification

The data is labeled using four different gene lists. CIViC and Martelotto et al. list the gene as well as the mutation location. Only mutations with the exact location and base change are used labeled. CIViC labels genes as *driver*, while all other methods label genes as TSG or OG. CGC and Bailey et al. label all mutations in a gene with the same label. Furthermore, we labeled genes with copy number variations in driver genes based on labels assigned by gene-based labeling strategies for multi-omic analysis. Classification of CIViC labels was a binary classification problem (classes: Driver, Neutral), while the rest were multi-class classification problems (classes: TSG, OG, Neutral). We used 70:30 that split the samples into train and test data (Figure 8B). All the mutations and/or CNV alterations were labeled using one of the four lists of driver genes described earlier. It is to be noted the numbers of data points in train and test split vary for the different labeling strategies (Supplementary Tables S1, S4, S5). The data is highly imbalanced, with a large number of neutral genes and very few TSG and OG. We used sampling algorithms for imbalanced data from *imblearn* package. Models were built using balanced random forest, balanced bagging, and easy ensemble. Five-fold cross-validation was conducted to tune hyperparameters using grid search. We also shuffle the labels of the training set to test for over-fitting. The precision-recall (PR) curve and the receiver operator curve (ROC) were plotted for all classes and all models including randomized labels (Supplementary Figures S1A–S42C). Furthermore, the top 20 features contributing to the model are plotted. The accuracy, F1 score, precision, and recall for the training and test set were calculated. All the models, plots, and output metrics are available in the GitHub folder and as supplementary data.

### 5.5 Feature, Domain, and miRNA Analysis

For each cancer type, the consensus feature contribution was calculated as the average feature contribution for all models built on the dataset. The top domains for SNV and multi-omic datasets were identified based on all domains with feature contribution >0 . Similarly, the top miRNA features with consensus feature contribution >0 for the cancer type were listed.

### 5.6 Comparison with Other Tools

The predictions of multi-omic models were compared with DawnRank, another personalized driver tool. The mutation and mRNA expression data were run using DawnRank. The complete network published with the tool was used for the analysis. For comparable results, we only consider the labels predicted by PRODIGY for mutated genes and not genes altered by CNV. For each sample, the precision was calculated for increasing ranks. Precision is calculated as the true positives divided by all predictions. True positives are genes predicted by tool and also present in the CGC list of driver genes. We calculate and plot the average rank for samples in a cancer type for varying ranks.

## Data Availability

Publicly available datasets were analyzed in this study. This data can be found here: https://github.com/RamanLab/PIVOT. The pre-print is available from bioRxiv.
