# Supplementary material for: Multi-Omic Data Improve Prediction of Personalized Tumor Suppressors and Oncogenes
Source: Front Genet. 2022 May 10;13:854190. doi: 10.3389/fgene.2022.854190 (PMC9127508; doi:10.3389/fgene.2022.854190)
Supplement: Supplementary file 5 [file DataSheet1.PDF]

Supplementary Material

List Of Supplementary Figures

- Figure S1A. Top 20 features for BRCA using SNV data with Bailey et al. labels built on “all” features using Balanced bagging.
- Figure S1B. Precision-recall curve for BRCA using SNV data with Bailey et al. labels built on “all” features using Balanced bagging.
- Figure S1C Receiver operating characteristic for BRCA using SNV data with Bailey et al. labels built on “all” features using Balanced bagging.
- Figure S2A. Top 20 features for BRCA using SNV data with Bailey et al. labels built on “some” features using Balanced bagging.
- Figure S2B. Precision-recall curve for BRCA using SNV data with Bailey et al. labels built on “some” features using Balanced bagging.
- Figure S2C Receiver operating characteristic for BRCA using SNV data with Bailey et al. labels built on “some” features using Balanced bagging.
- Figure S3A. Top 20 features for BRCA using SNV data with CGC labels built on “all” features using Balanced bagging.
- Figure S3B. Precision-recall curve for BRCA using SNV data with CGC labels built on “all” features using Balanced bagging.
- Figure S3C. Receiver operating characteristic for BRCA using SNV data with CGC labels built on “all” features using Balanced bagging.
- Figure S4A. Top 20 features for BRCA using SNV data with CGC labels built on “some” features using Balanced Random Forest.
- Figure S4B. Precision-recall curve for BRCA using SNV data with CGC labels built on “some” features using Balanced Random Forest.
- Figure S4C. Receiver operating characteristic for BRCA using SNV data with CGC labels built on “some” features using Balanced Random Forest.
- Figure S5A. Top 20 features for BRCA using SNV data with CIViC labels built on “all” features using Easy Ensemble.
- Figure S5B. Precision-recall curve for BRCA using SNV data with CIViC labels built on “all” features using Easy Ensemble.
- Figure S5C. Receiver operating characteristic for BRCA using SNV data with CIViC labels built on “all” features using Easy Ensemble.
- Figure S6A. Top 20 features for BRCA using SNV data with CIViC labels built on “some” features using Balanced Bagging.
- Figure S6B. Precision-recall curve for BRCA using SNV data with CIViC labels built on “some” features using Balanced Bagging.
- Figure S6C. Receiver operating characteristic for BRCA using SNV data with CIViC labels built on “some” features using Balanced Bagging.
- Figure S7A. Top 20 features for BRCA using SNV data with Marellotto et al. labels built on “all” features using Easy Ensemble.

- Figure S7B. Precision-recall curve for BRCA using SNV data with Marellotto et al. labels built on “all” features using Easy Ensemble.
- Figure S7C. Receiver operating characteristic for BRCA using SNV data with Marellotto et al. labels built on “all” features using Easy Ensemble.
- Figure S8A. Top 20 features for BRCA using SNV data with Marellotto et al. labels built on “some” features using Balanced Bagging.
- Figure S8B. Precision-recall curve for BRCA using SNV data with Marellotto et al. labels built on “some” features using Balanced Bagging.
- Figure S8C. Receiver operating characteristic for BRCA using SNV data with Marellotto et al. labels built on “some” features using Balanced Bagging.
- Figure S9A. Top 20 features for COAD using SNV data with Bailey et al. labels built on “all” features using Balanced Random Forest.
- Figure S9B. Precision-recall curve for COAD using SNV data with Bailey et al. labels built on “all” features using Balanced Random Forest.
- Figure S9C Receiver operating characteristic for COAD using SNV data with Bailey et al. labels built on “all” features using Balanced Random Forest.
- Figure S10A. Top 20 features for COAD using SNV data with Bailey et al. labels built on “some” features using Easy Ensemble.
- Figure S10B. Precision-recall curve for COAD using SNV data with Bailey et al. labels built on “some” features using Easy Ensemble.
- Figure S10C Receiver operating characteristic for COAD using SNV data with Bailey et al. labels built on “some” features using Easy Ensemble.
- Figure S11A. Top 20 features for COAD using SNV data with CGC labels built on “all” features using Balanced Bagging.
- Figure S11B. Precision-recall curve for COAD using SNV data with CGC labels built on “all” features using Balanced Bagging.
- Figure S11C Receiver operating characteristic for COAD using SNV data with CGC labels built on “all” features using Balanced Bagging.
- Figure S12A. Top 20 features for COAD using SNV data with CGC labels built on “some” features using Balanced Random Forest.
- Figure S12B. Precision-recall curve for COAD using SNV data with CGC labels built on “some” features using Balanced Random Forest.
- Figure S12C Receiver operating characteristic for COAD using SNV data with CGC labels built on “some” features using Balanced Random Forest.
- Figure S13A. Top 20 features for LGG using SNV data with Bailey et al. labels built on “all” features using Balanced Random Forest.
- Figure S13B. Precision-recall curve for LGG using SNV data with Bailey et al. labels built on “all” features using Balanced Random Forest.
- Figure S13C Receiver operating characteristic for LGG using SNV data with Bailey et al. labels built on “all” features using Balanced Random Forest.
- Figure S14A. Top 20 features for LGG using SNV data with Bailey et al. labels built on “some” features using Easy Ensemble.
- Figure S14B. Precision-recall curve for LGG using SNV data with Bailey et al. labels

built on “some” features using Easy Ensemble.
- Figure S14C Receiver operating characteristic for LGG using SNV data with Bailey et al. labels built on “some” features using Easy Ensemble.
- Figure S15A. Top 20 features for LGG using SNV data with CGC labels built on “all” features using Balanced Bagging.
- Figure S15B. Precision-recall curve for LGG using SNV data with CGC labels built on “all” features using Balanced Bagging.
- Figure S15C Receiver operating characteristic for LGG using SNV data with CGC labels built on “all” features using Balanced Bagging.
- Figure S16A. Top 20 features for LGG using SNV data with CGC labels built on “some” features using Balanced Random Forest.
- Figure S16B. Precision-recall curve for LGG using SNV data with CGC labels built on “some” features using Balanced Random Forest.
- Figure S16C Receiver operating characteristic for LGG using SNV data with CGC labels built on “some” features using Balanced Random Forest.
- Figure S17A. Top 20 features for LUAD using SNV data with Bailey et al. labels built on “all” features using Balanced Random Forest.
- Figure S17B. Precision-recall curve for LUAD using SNV data with Bailey et al. labels built on “all” features using Balanced Random Forest.
- Figure S17C Receiver operating characteristic for LUAD using SNV data with Bailey et al. labels built on “all” features using Balanced Random Forest.
- Figure S18A. Top 20 features for LUAD using SNV data with Bailey et al. labels built on “some” features using Balanced Random Forest.
- Figure S18B. Precision-recall curve for LUAD using SNV data with Bailey et al. labels built on “some” features using Balanced Random Forest.
- Figure S18C Receiver operating characteristic for LUAD using SNV data with Bailey et al. labels built on “some” features using Balanced Random Forest.
- Figure S19A. Top 20 features for BRCA using RNA data with Bailey et al. labels built using Balanced bagging.
- Figure S19B. Precision-recall curve for BRCA using RNA data with Bailey et al. labels built using Balanced bagging.
- Figure S19C Receiver operating characteristic for BRCA using RNA data with Bailey et al. labels built using Balanced bagging.
- Figure S20A. Top 20 features for BRCA using RNA data with CGC labels built using Balanced bagging.
- Figure S20B. Precision-recall curve for BRCA using RNA data with CGC labels built using Balanced bagging.
- Figure S20C Receiver operating characteristic for BRCA using RNA data with CGC labels built using Balanced bagging.
- Figure S21A. Top 20 features for BRCA using RNA data with CIViC labels built using Balanced bagging.
- Figure S21B. Precision-recall curve for BRCA using RNA data with CIViC labels built using Balanced bagging.

- Figure S21C Receiver operating characteristic for BRCA using RNA data with CIViC labels built using Balanced bagging.
- Figure S22A. Top 20 features for BRCA using RNA data with Marellotto et al. labels built using Easy Ensemble.
- Figure S22B. Precision-recall curve for BRCA using RNA data with Marellotto et al. labels built using Easy Ensemble.
- Figure S22C Receiver operating characteristic for BRCA using RNA data with Marellotto et al. labels built using Easy Ensemble.
- Figure S23A. Top 20 features for COAD using RNA data with Bailey et al. labels built using Balanced bagging.
- Figure S23B. Precision-recall curve for COAD using RNA data with Bailey et al. labels built using Balanced bagging.
- Figure S23C Receiver operating characteristic for COAD using RNA data with Bailey et al. labels built using Balanced bagging.
- Figure S24A. Top 20 features for COAD using RNA data with CGC labels built using Balanced bagging.
- Figure S24B. Precision-recall curve for COAD using RNA data with CGC labels built using Balanced bagging.
- Figure S24C Receiver operating characteristic for COAD using RNA data with CGC labels built using Balanced bagging.
- Figure S25A. Top 20 features for LUAD using RNA data with Bailey et al. labels built using Balanced bagging.
- Figure S25B. Precision-recall curve for LUAD using RNA data with Bailey et al. labels built using Balanced bagging.
- Figure S25C Receiver operating characteristic for LUAD using RNA data with Bailey et al. labels built using Balanced bagging.
- Figure S26A. Top 20 features for LUAD using RNA data with CGC labels built using Balanced bagging.
- Figure S26B. Precision-recall curve for LUAD using RNA data with CGC labels built using Balanced bagging.
- Figure S26C Receiver operating characteristic for LUAD using RNA data with CGC labels built using Balanced bagging.
- Figure S27A. Top 20 features for BRCA using multi-omic data with Bailey et al. labels built on “all” features using Balanced bagging.
- Figure S27B. Precision-recall curve for BRCA using multi-omic data with Bailey et al. labels built on “all” features using Balanced bagging.
- Figure S27C Receiver operating characteristic for BRCA using multi-omic data with Bailey et al. labels built on “all” features using Balanced bagging.
- Figure S28A. Top 20 features for BRCA using multi-omic data with Bailey et al. labels built on “some” features using Balanced bagging.
- Figure S28B. Precision-recall curve for BRCA using multi-omic data with Bailey et al. labels built on “some” features using Balanced bagging.
- Figure S28C Receiver operating characteristic for BRCA using multi-omic data with

Bailey et al. labels built on “some” features using Balanced bagging.

- Figure S29A. Top 20 features for BRCA using multi-omic data with CGC labels built on “all” features using Balanced bagging.
- Figure S29B. Precision-recall curve for BRCA using multi-omic data with CGC labels built on “all” features using Balanced bagging.
- Figure S29C. Receiver operating characteristic for BRCA using multi-omic data with CGC labels built on “all” features using Balanced Bagging.
- Figure S30A. Top 20 features for BRCA using multi-omic data with CGC labels built on “some” features using Balanced Bagging.
- Figure S30B. Precision-recall curve for BRCA using multi-omic data with CGC labels built on “some” features using Balanced Bagging.
- Figure S30C. Receiver operating characteristic for BRCA using multi-omic data with CGC labels built on “some” features using Balanced Bagging.
- Figure S31A. Top 20 features for BRCA using multi-omic data with CIViC labels built on “all” features using Balanced Bagging.
- Figure S31B. Precision-recall curve for BRCA using multi-omic data with CIViC labels built on “all” features using Balanced Bagging.
- Figure S31C. Receiver operating characteristic for BRCA using multi-omic data with CIViC labels built on “all” features using Balanced Bagging.
- Figure S32A. Top 20 features for BRCA using multi-omic data with CIViC labels built on “some” features using Balanced Bagging.
- Figure S32B. Precision-recall curve for BRCA using multi-omic data with CIViC labels built on “some” features using Balanced Bagging.
- Figure S32C. Receiver operating characteristic for BRCA using multi-omic data with CIViC labels built on “some” features using Balanced Bagging.
- Figure S33A. Top 20 features for BRCA using multi-omic data with Marellotto et al. labels built on “all” features using Balanced Bagging.
- Figure S33B. Precision-recall curve for BRCA using multi-omic data with Marellotto et al. labels built on “all” features using Balanced Bagging.
- Figure S33C. Receiver operating characteristic for BRCA using multi-omic data with Marellotto et al. labels built on “all” features using Balanced Bagging.
- Figure S34A. Top 20 features for BRCA using multi-omic data with Marellotto et al. labels built on “some” features using Balanced Bagging.
- Figure S34B. Precision-recall curve for BRCA using multi-omic data with Marellotto et al. labels built on “some” features using Balanced Bagging.
- Figure S34C. Receiver operating characteristic for BRCA using multi-omic data with Marellotto et al. labels built on “some” features using Balanced Bagging.
- Figure S35A. Top 20 features for COAD using multi-omic data with Bailey et al. labels built on “all” features using Balanced bagging.
- Figure S35B. Precision-recall curve for COAD using multi-omic data with Bailey et al. labels built on “all” features using Balanced bagging.
- Figure S35C Receiver operating characteristic for COAD using multi-omic data with Bailey et al. labels built on “all” features using Balanced bagging.

- Figure S36A. Top 20 features for COAD using multi-omic data with Bailey et al. labels built on “some” features using Balanced bagging.
- Figure S36B. Precision-recall curve for COAD using multi-omic data with Bailey et al. labels built on “some” features using Balanced bagging.
- Figure S36C Receiver operating characteristic for COAD using multi-omic data with Bailey et al. labels built on “some” features using Balanced bagging.
- Figure S37A. Top 20 features for COAD using multi-omic data with CGC labels built on “all” features using Balanced bagging.
- Figure S37B. Precision-recall curve for COAD using multi-omic data with CGC labels built on “all” features using Balanced bagging.
- Figure S37C. Receiver operating characteristic for COAD using multi-omic data with CGC labels built on “all” features using Balanced Bagging.
- Figure S38A. Top 20 features for COAD using multi-omic data with CGC labels built on “some” features using Balanced Bagging.
- Figure S38B. Precision-recall curve for COAD using multi-omic data with CGC labels built on “some” features using Balanced Bagging.
- Figure S38C. Receiver operating characteristic for COAD using multi-omic data with CGC labels built on “some” features using Balanced Bagging.
- Figure S39A. Top 20 features for LUAD using multi-omic data with Bailey et al. labels built on “all” features using Balanced bagging.
- Figure S39B. Precision-recall curve for LUAD using multi-omic data with Bailey et al. labels built on “all” features using Balanced bagging.
- Figure S39C Receiver operating characteristic for LUAD using multi-omic data with Bailey et al. labels built on “all” features using Balanced bagging.
- Figure S40A. Top 20 features for LUAD using multi-omic data with Bailey et al. labels built on “some” features using Balanced bagging.
- Figure S40B. Precision-recall curve for LUAD using multi-omic data with Bailey et al. labels built on “some” features using Balanced bagging.
- Figure S40C Receiver operating characteristic for LUAD using multi-omic data with Bailey et al. labels built on “some” features using Balanced bagging.
- Figure S41A. Top 20 features for LUAD using multi-omic data with CGC labels built on “all” features using Balanced bagging.
- Figure S41B. Precision-recall curve for LUAD using multi-omic data with CGC labels built on “all” features using Balanced bagging.
- Figure S41C. Receiver operating characteristic for LUAD using multi-omic data with CGC labels built on “all” features using Balanced Bagging.
- Figure S42A. Top 20 features for LUAD using multi-omic data with CGC labels built on “some” features using Balanced Bagging.
- Figure S42B. Precision-recall curve for LUAD using multi-omic data with CGC labels built on “some” features using Balanced Bagging.
- Figure S42C. Receiver operating characteristic for LUAD using multi-omic data with CGC labels built on “some” features using Balanced Bagging.
- Figure S43. Distribution of genes predicted for samples in BRCA.

- Figure S44. Distribution of mutated genes predicted for samples in BRCA.
- Figure S45. Distribution of CNV altered genes predicted for samples in BRCA.
- Figure S46. Distribution of degree of genes predicted for samples in BRCA.
- Figure S47. Distribution of predicted oncogenes for samples in BRCA.
- Figure S48. Distribution of predicted tumour suppressor genes in BRCA.
- Figure S49. Distribution of genes predicted for samples in COAD.
- Figure S50. Distribution of mutated genes predicted for samples in COAD.
- Figure S51. Distribution of CNV altered genes predicted for samples in COAD.
- Figure S52. Distribution of degree of genes predicted for samples in COAD.
- Figure S53. Distribution of predicted oncogenes for samples in COAD.
- Figure S54. Distribution of predicted tumour suppressor genes in COAD.
- Figure S55. Distribution of samples for genes predicted as driver in COAD.
- Figure S56. Distribution of genes predicted for samples in LUAD.
- Figure S57. Distribution of mutated genes predicted for samples in LUAD.
- Figure S58. Distribution of CNV altered genes predicted for samples in LUAD.
- Figure S59. Distribution of degree of genes predicted for samples in LUAD.
- Figure S60. Distribution of predicted oncogenes for samples in LUAD.
- Figure S61. Distribution of predicted tumour suppressor genes in LUAD.
- Figure S62. Distribution of samples for genes predicted as driver in LUAD.

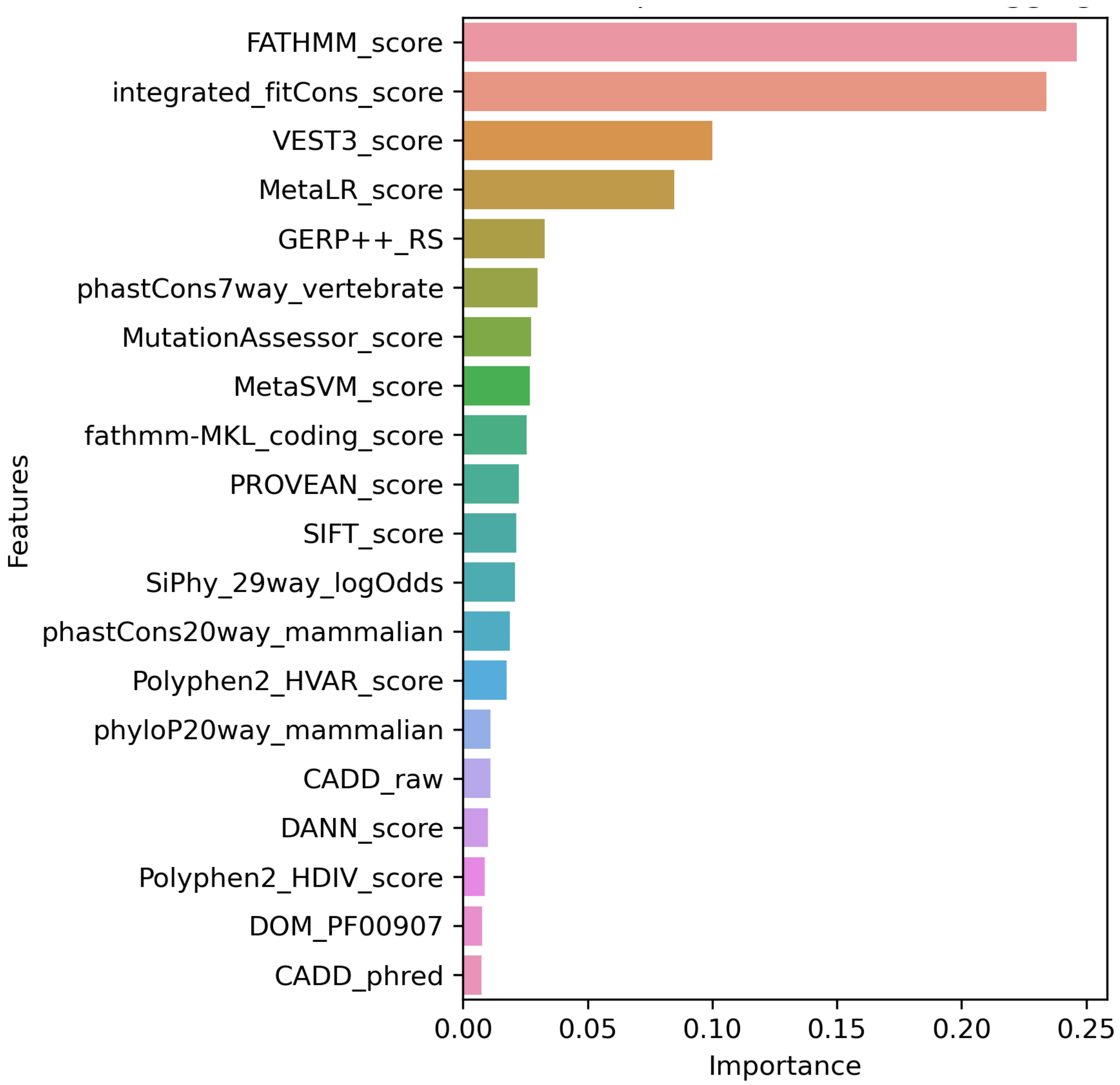

Figure S1A. Top 20 features for BRCA using SNV data with Bailey *et al.* labels built on "all" features using Balanced bagging.

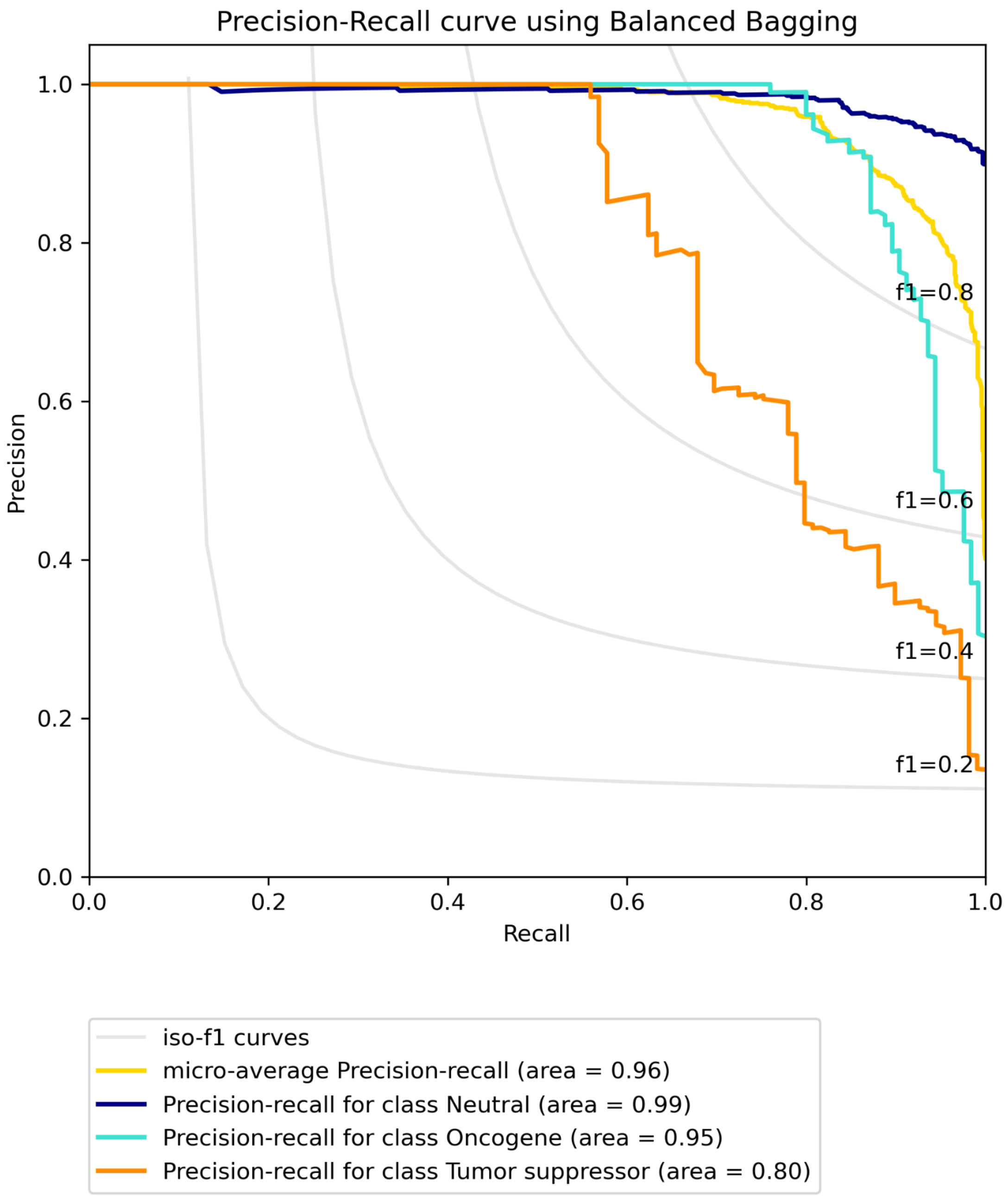

Figure S1B. Precision-recall curve for BRCA using SNV data with Bailey *et al.* labels built on "all" features using Balanced bagging.

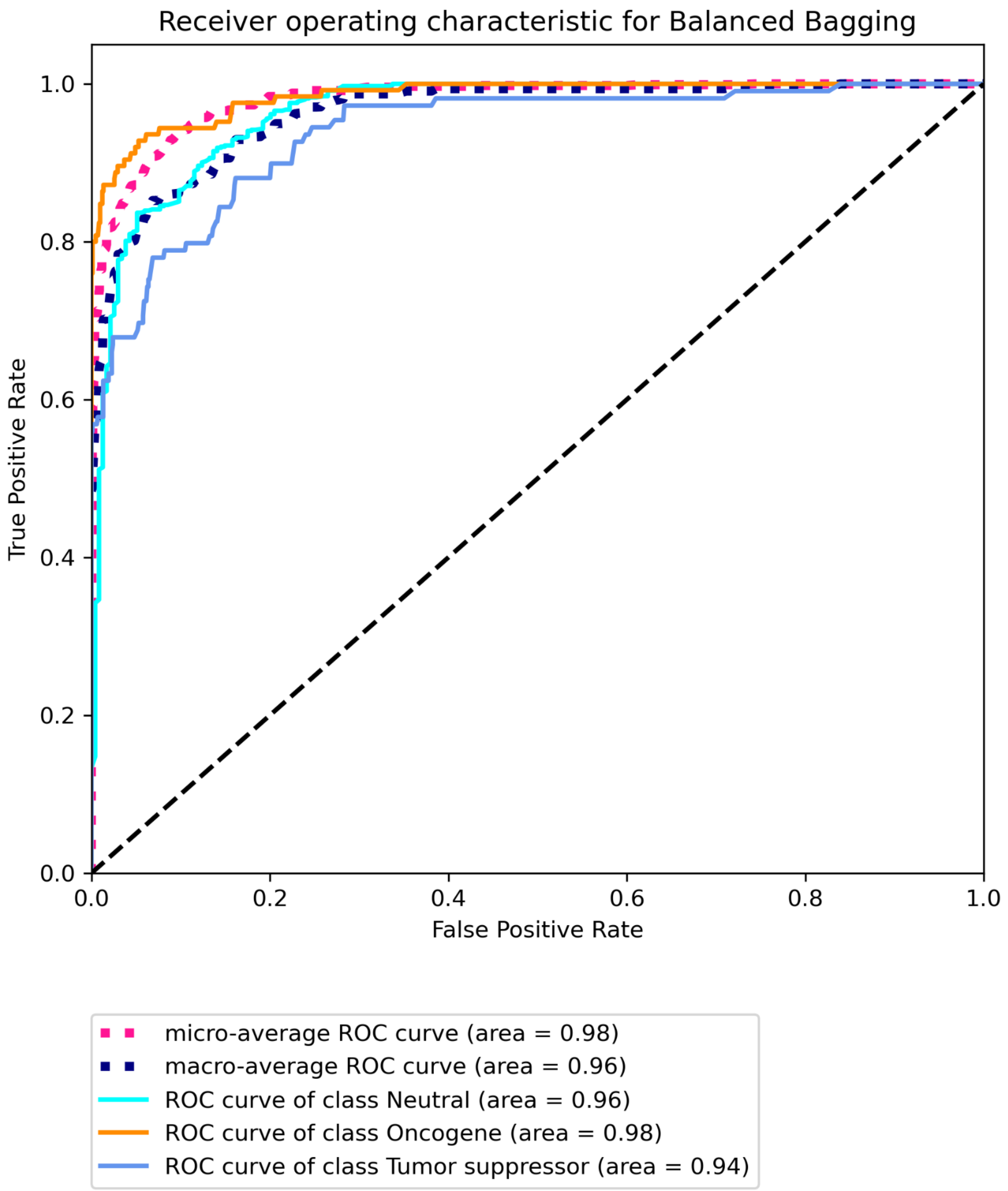

Figure S1C Receiver operating characteristic for BRCA using SNV data with Bailey *et al.* labels built on "all" features using Balanced bagging.

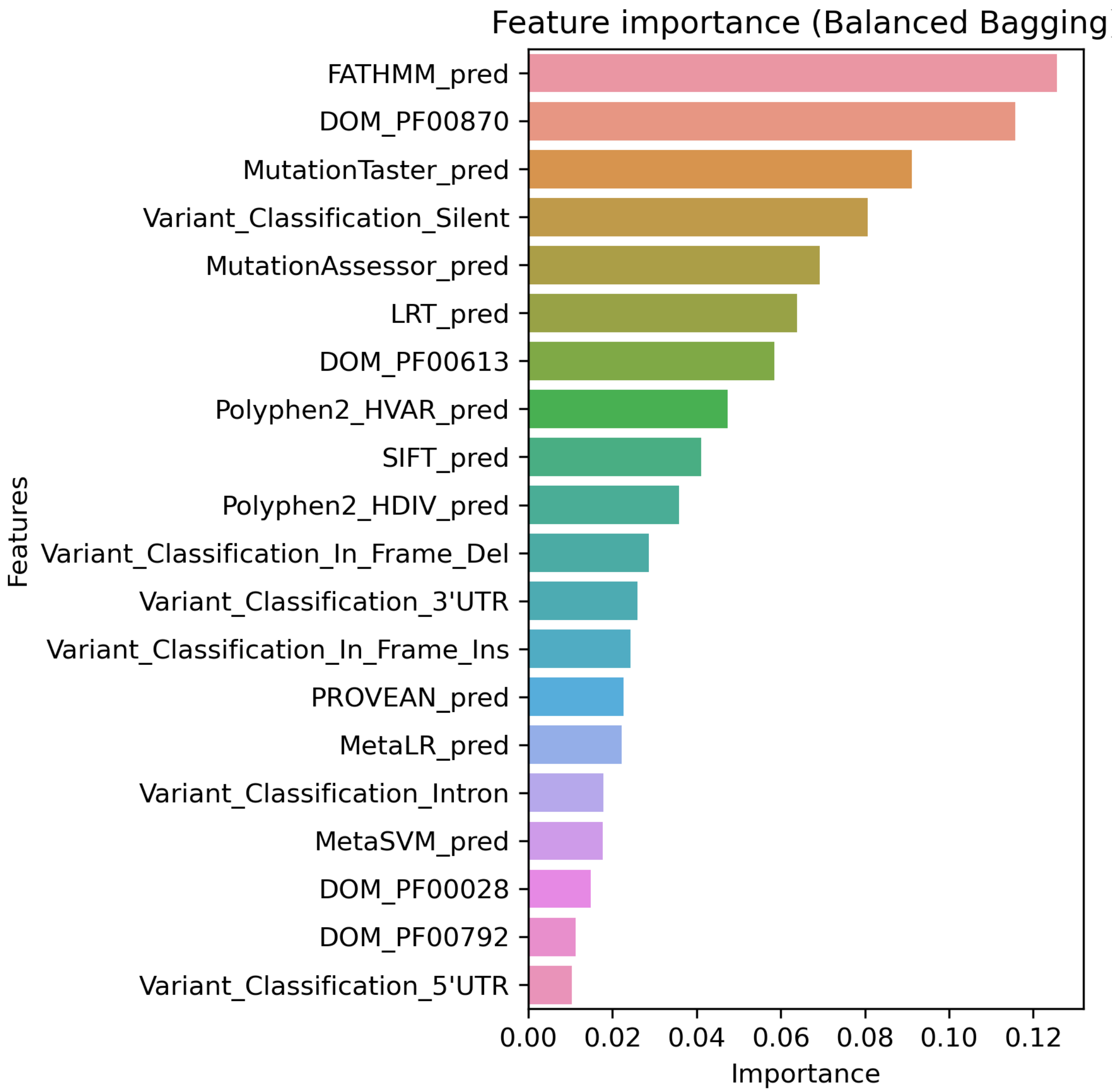

Figure S2A. Top 20 features for BRCA using SNV data with Bailey *et al.* labels built on “some” features using Balanced bagging.

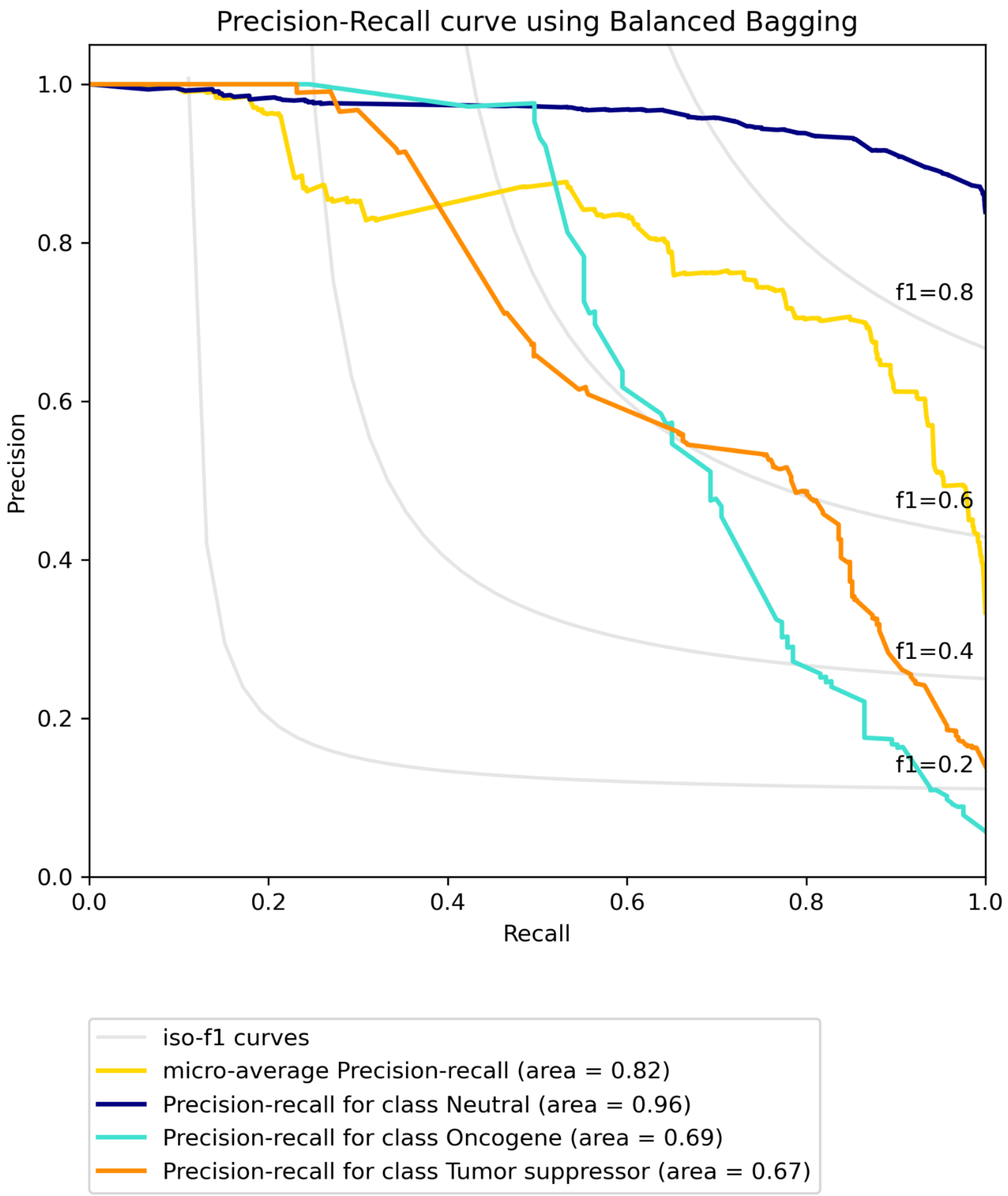

Figure S2B. Precision-recall curve for BRCA using SNV data with Bailey *et al.* labels built on "some" features using Balanced bagging.

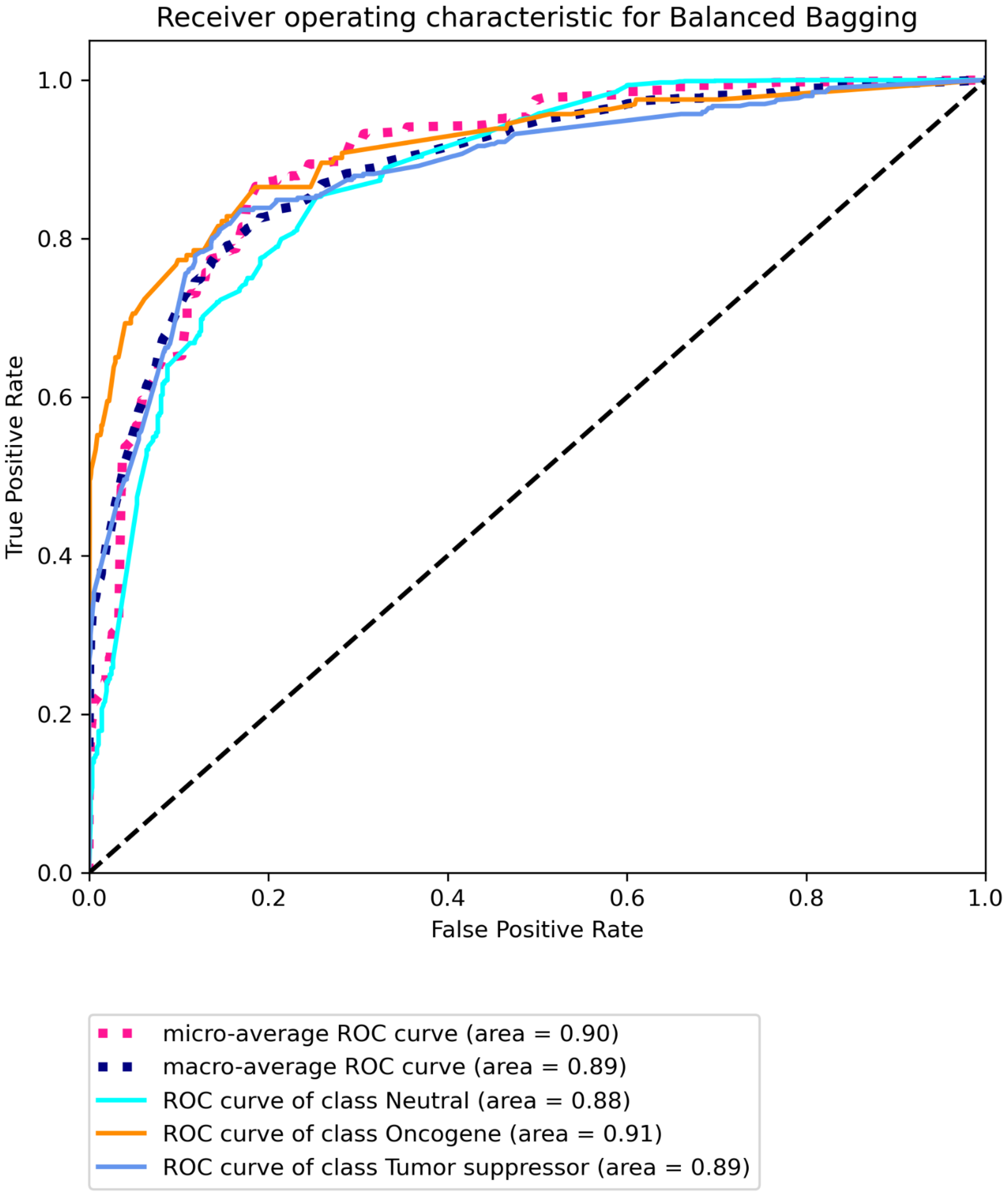

Figure S2C Receiver operating characteristic for BRCA using SNV data with Bailey *et al.* labels built on "some" features using Balanced bagging.

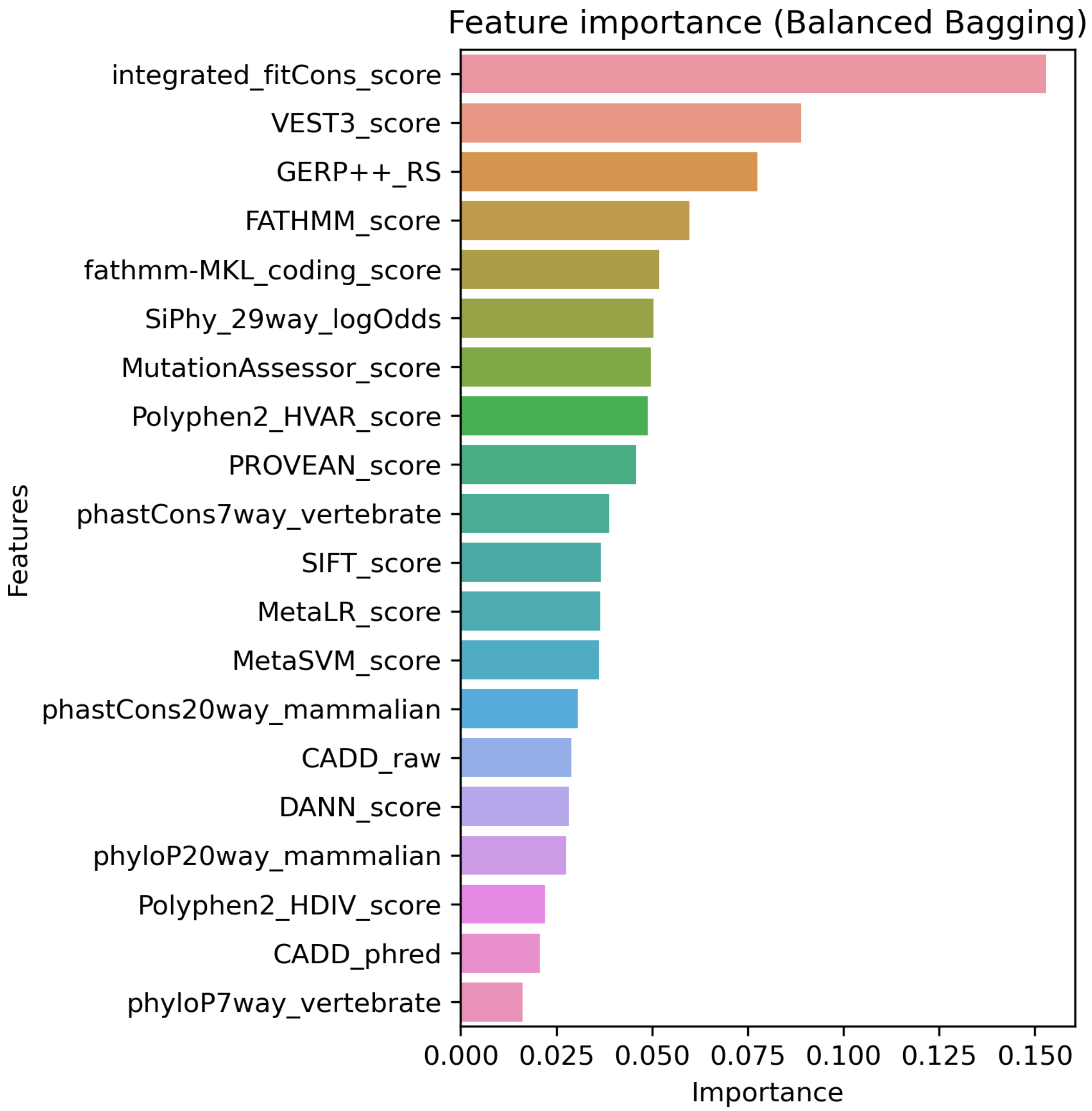

Figure S3A. Top 20 features for BRCA using SNV data with CGC labels built on "all" features using Balanced bagging.

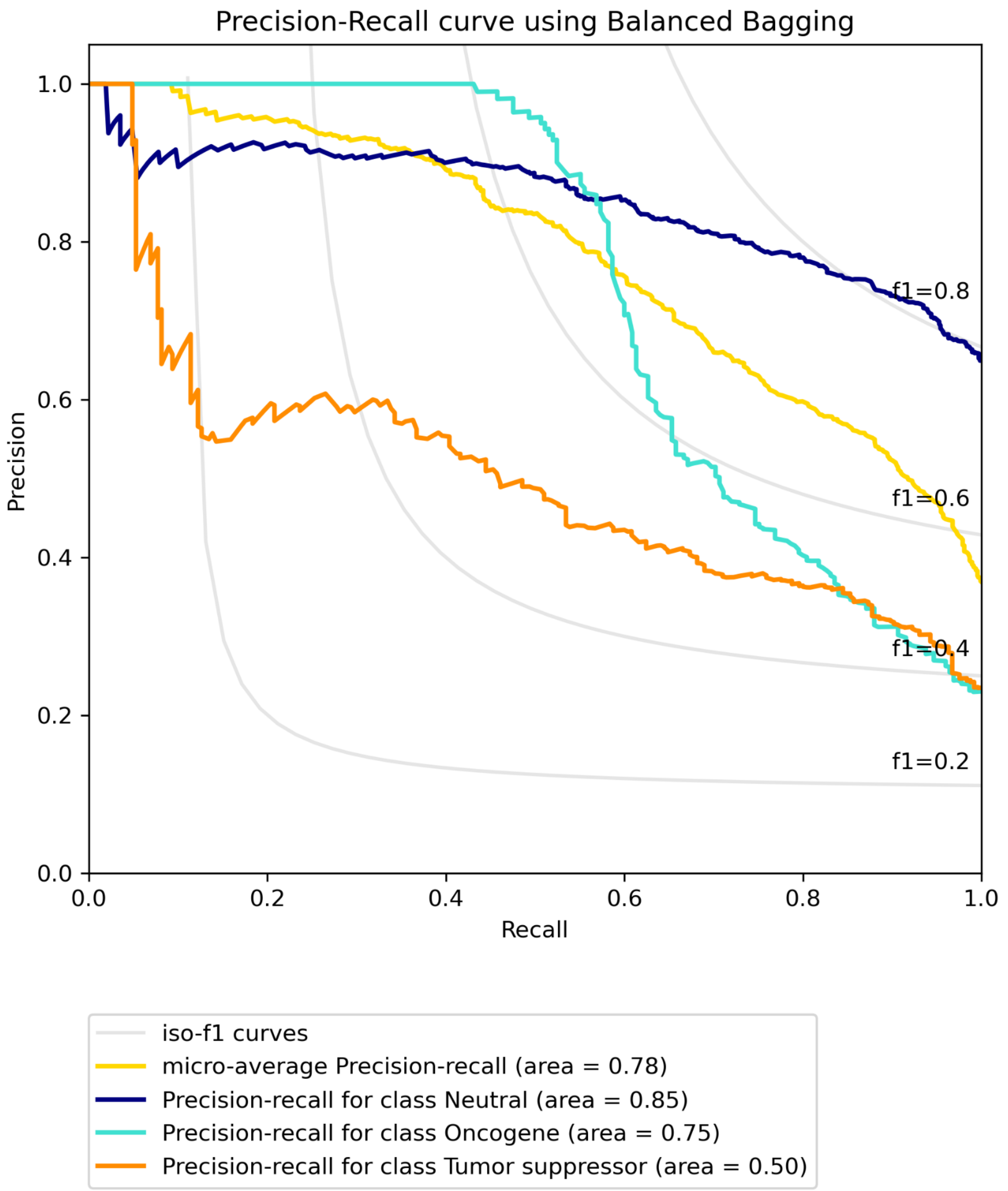

Figure S3B. Precision-recall curve for BRCA using SNV data with CGC labels built on “all” features using Balanced bagging.

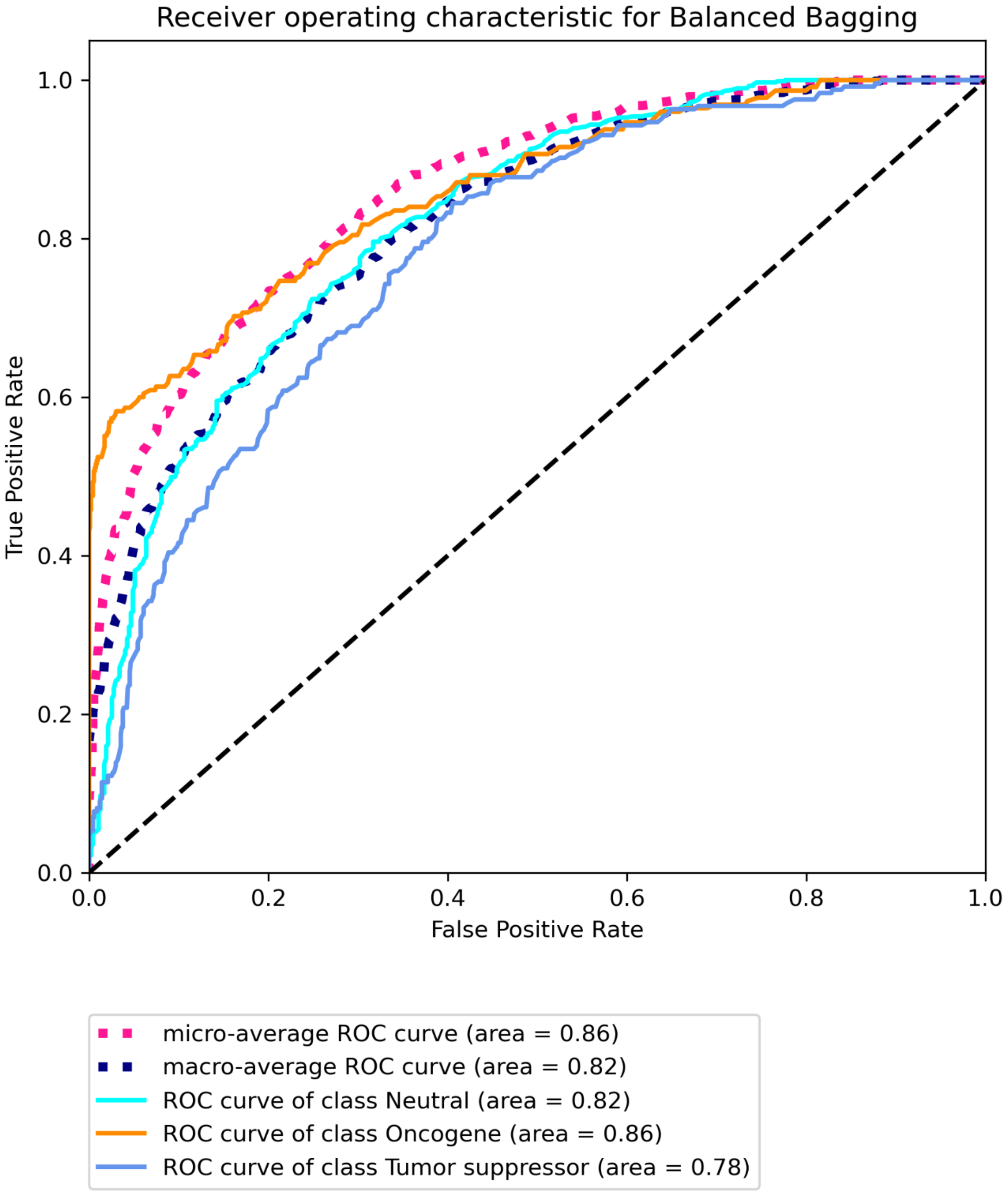

Figure S3C. Receiver operating characteristic for BRCA using SNV data with CGC labels built on “all” features using Balanced bagging.

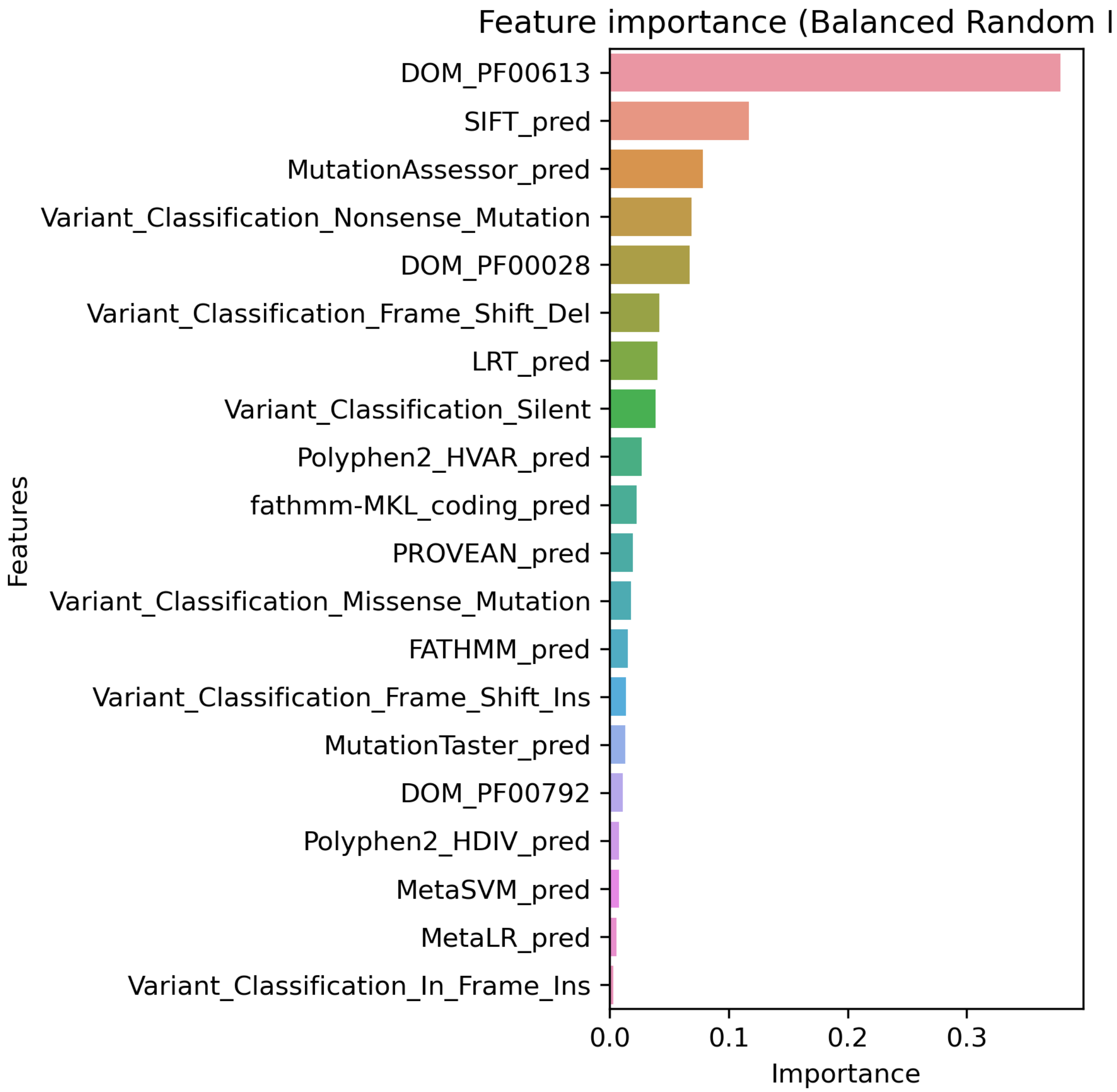

Figure S4A. Top 20 features for BRCA using SNV data with CGC labels built on "some" features using Balanced Random Forest.

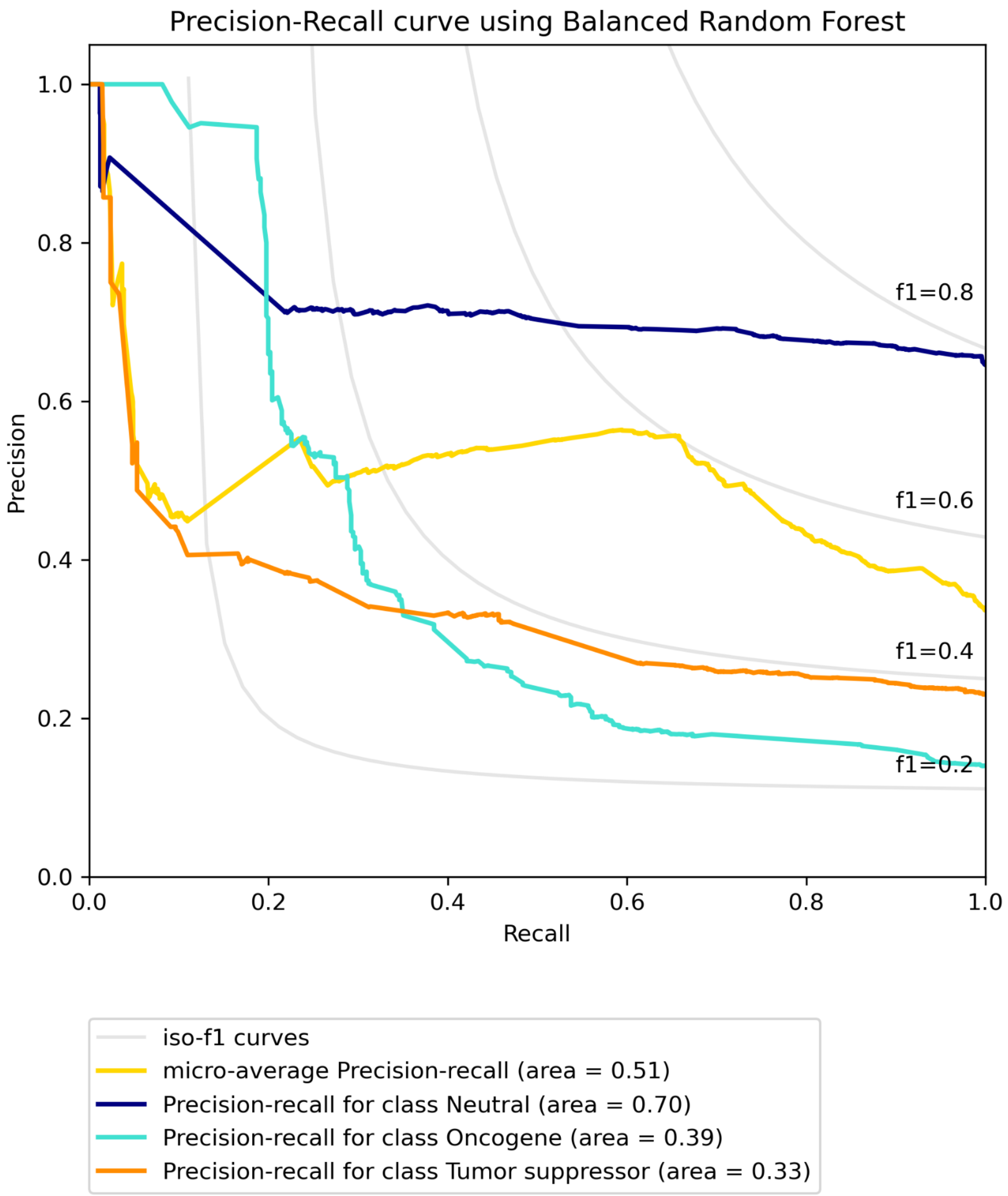

Figure S4B. Precision-recall curve for BRCA using SNV data with CGC labels built on “some” features using Balanced Random Forest.

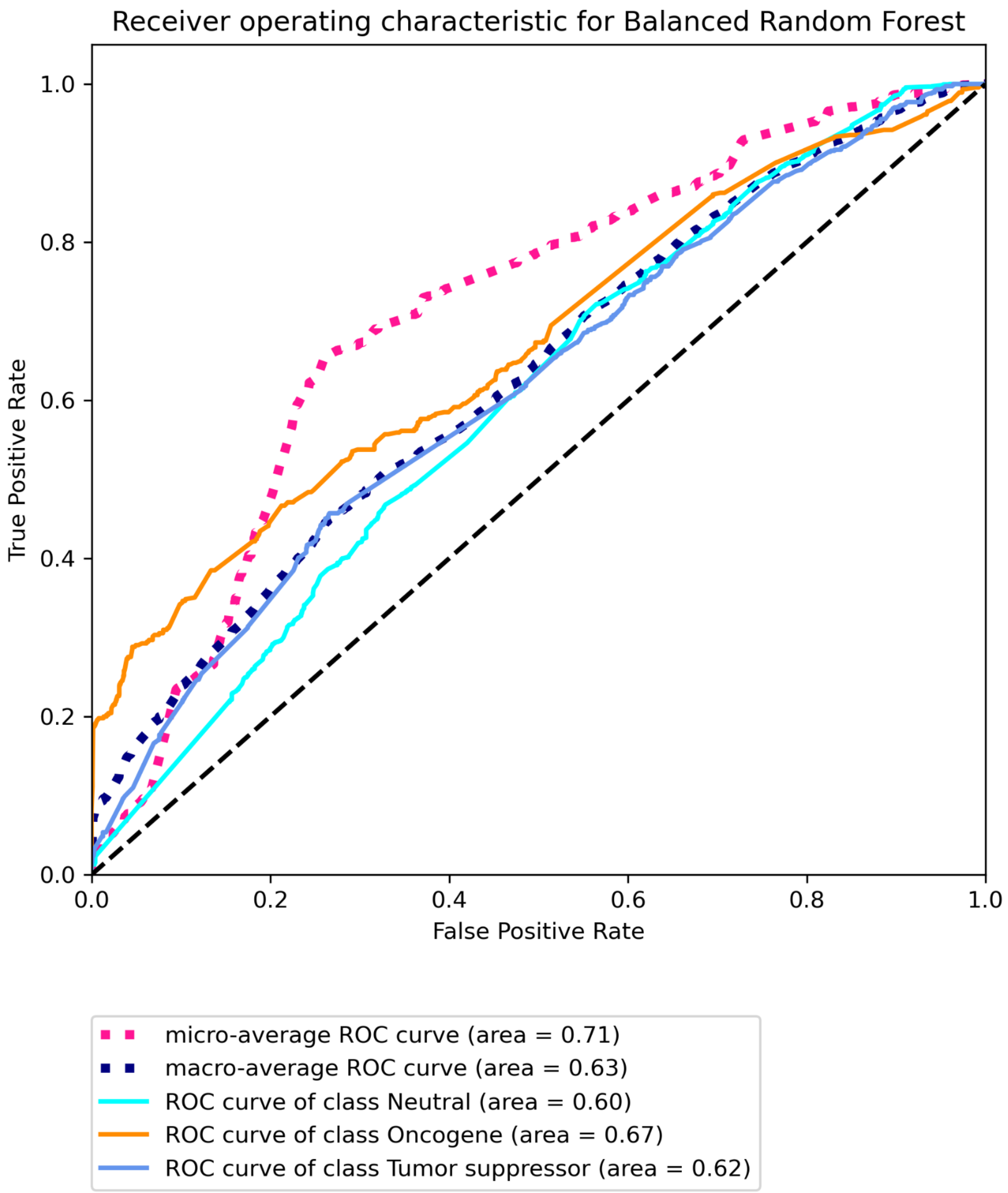

Figure S4C. Receiver operating characteristic for BRCA using SNV data with CGC labels built on "some" features using Balanced Random Forest.

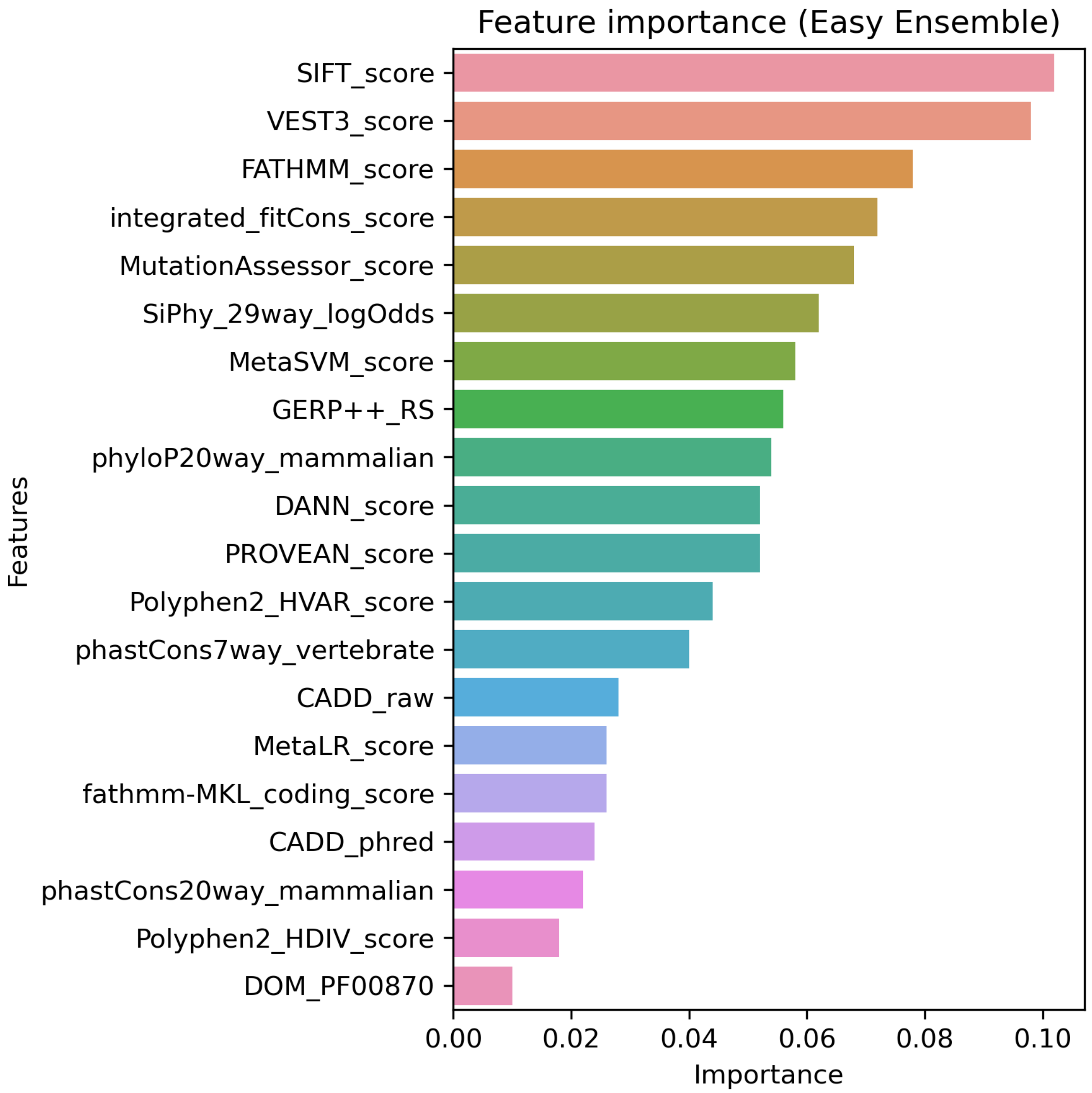

Figure S5A. Top 20 features for BRCA using SNV data with CIViC labels built on "all" features using Easy Ensemble.

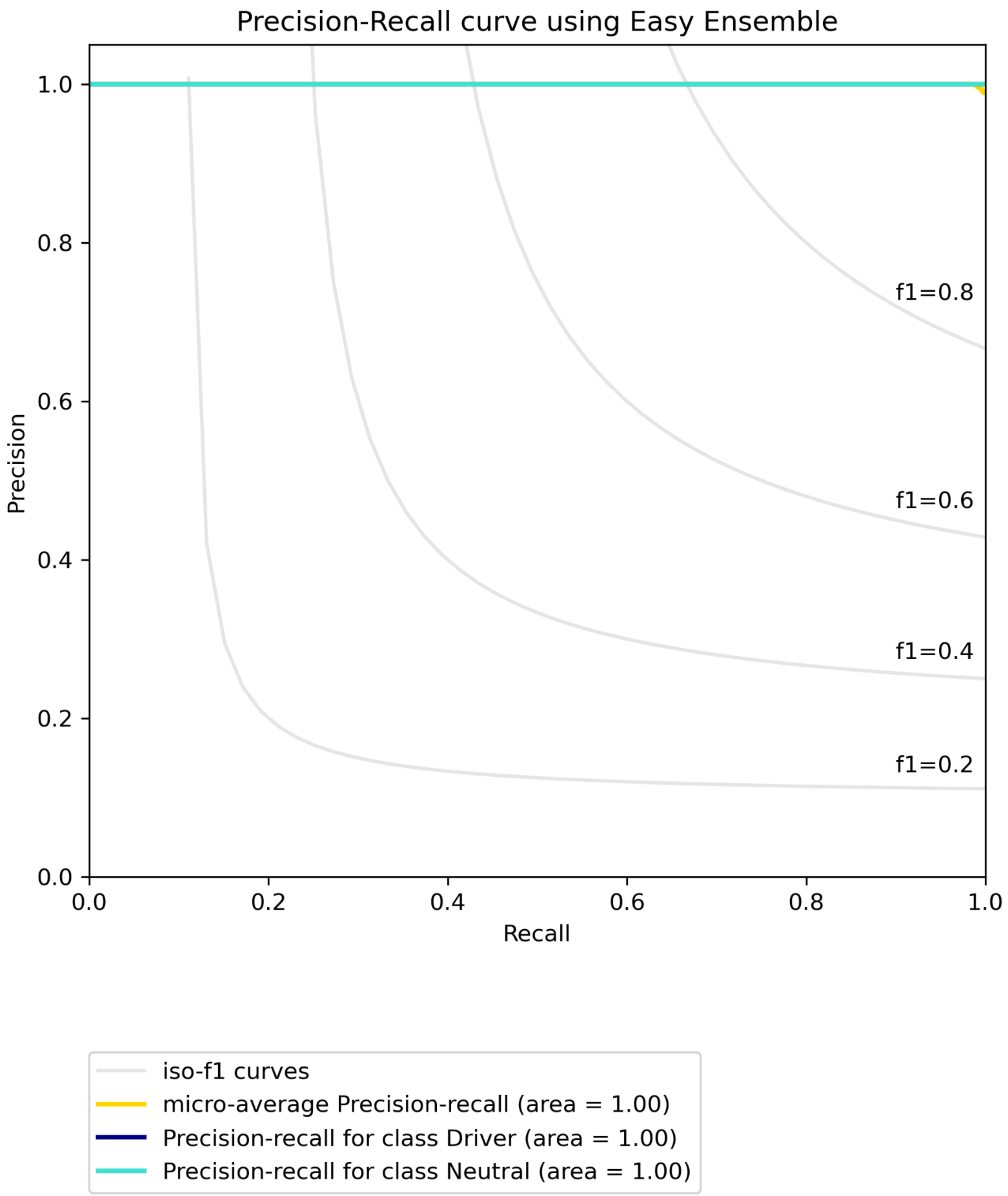

Figure S5B. Precision-recall curve for BRCA using SNV data with CIViC labels built on "all" features using Easy Ensemble.

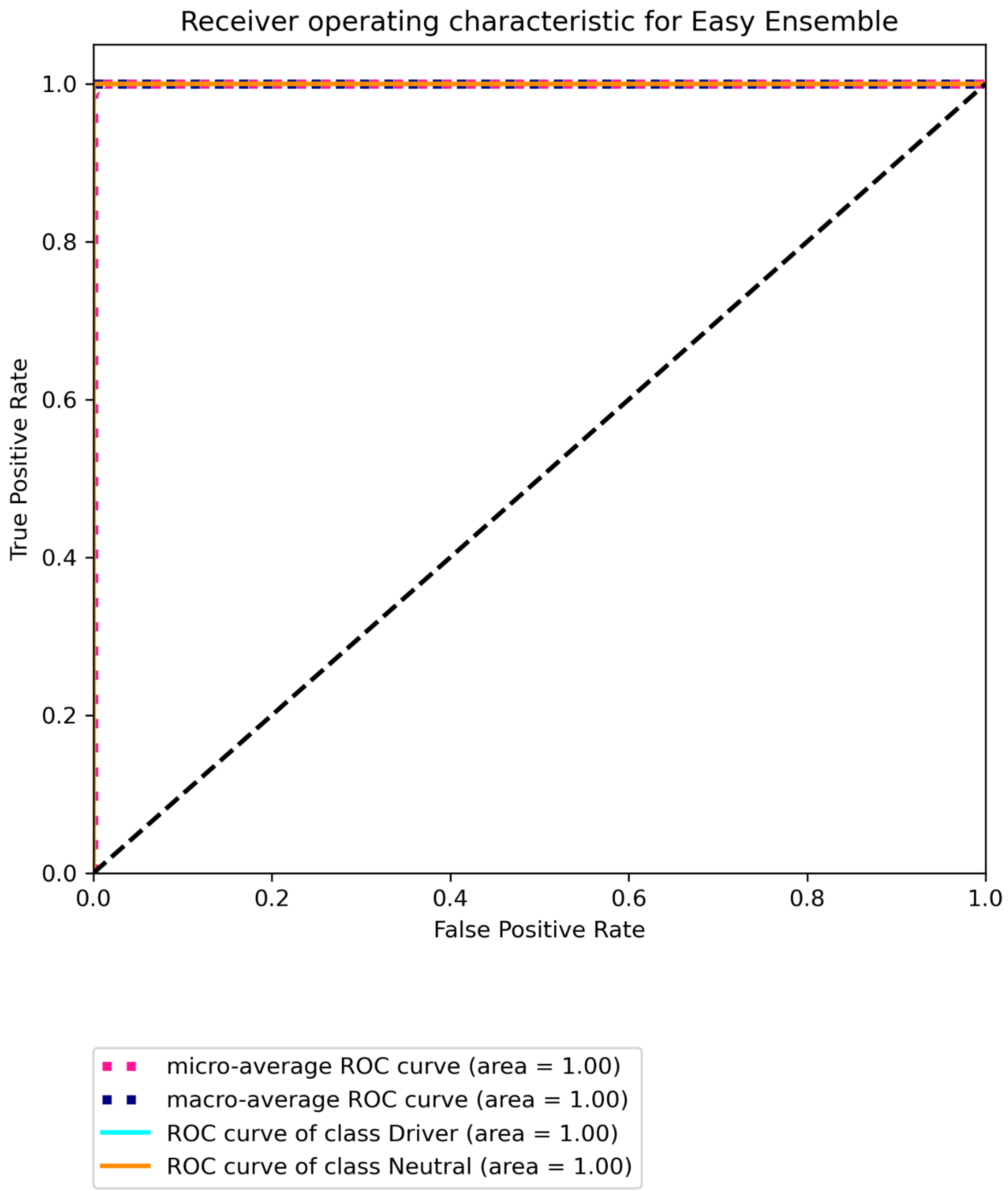

Figure S5C. Receiver operating characteristic for BRCA using SNV data with CIViC labels built on "all" features using Easy Ensemble.

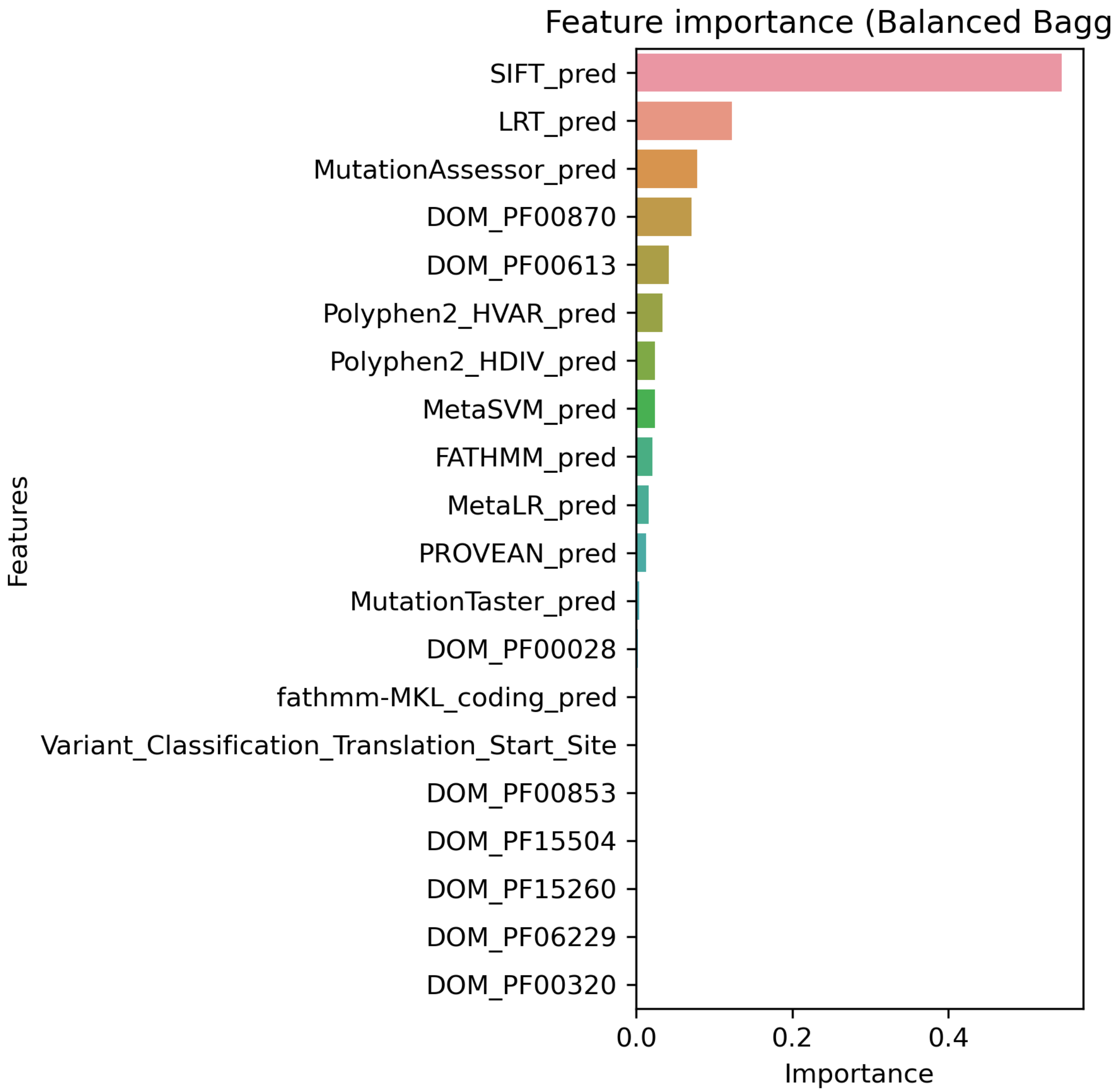

Figure S6A. Top 20 features for BRCA using SNV data with CIViC labels built on "some" features using Balanced Bagging.

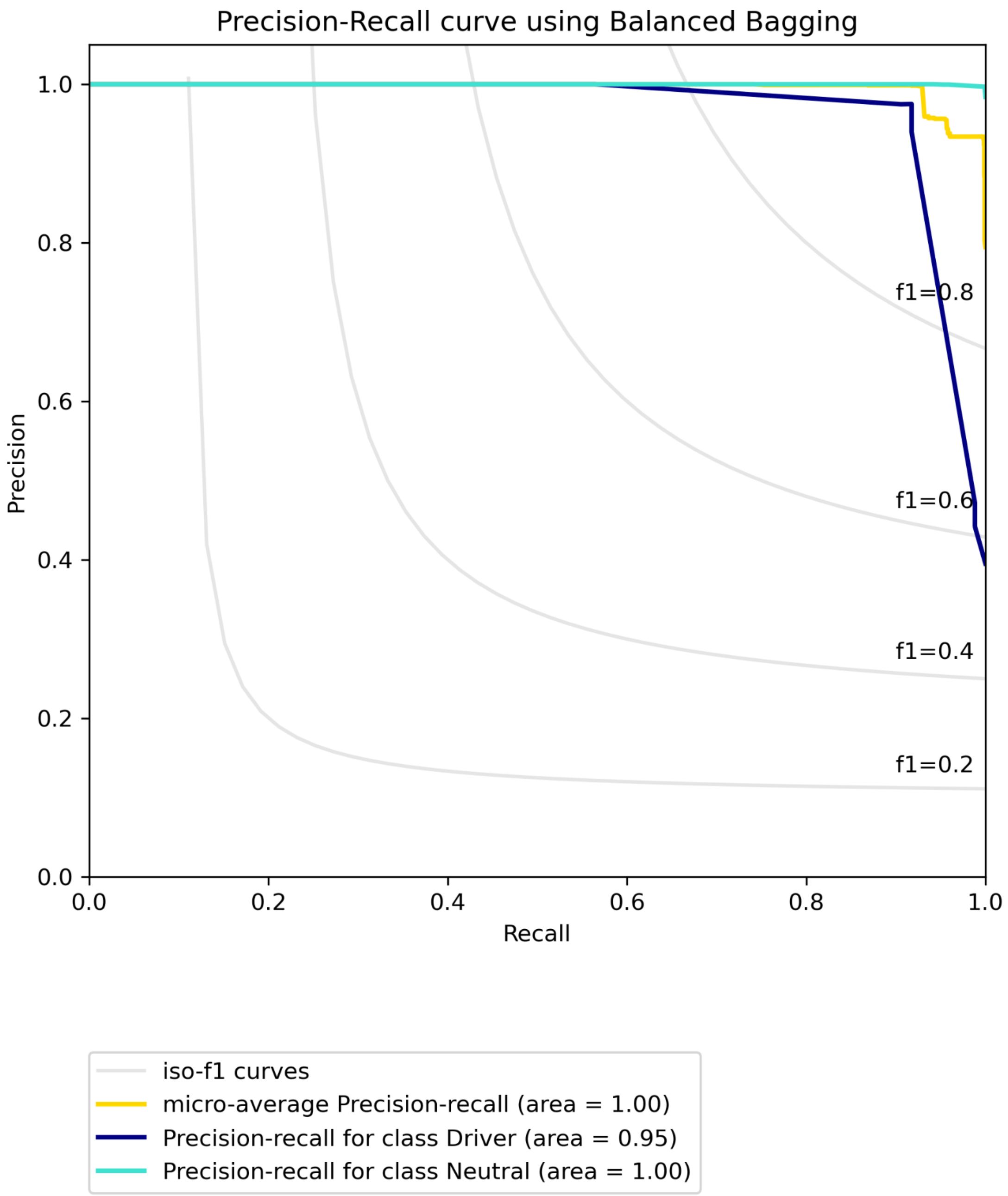

Figure S6B. Precision-recall curve for BRCA using SNV data with CIViC labels built on "some" features using Balanced Bagging.

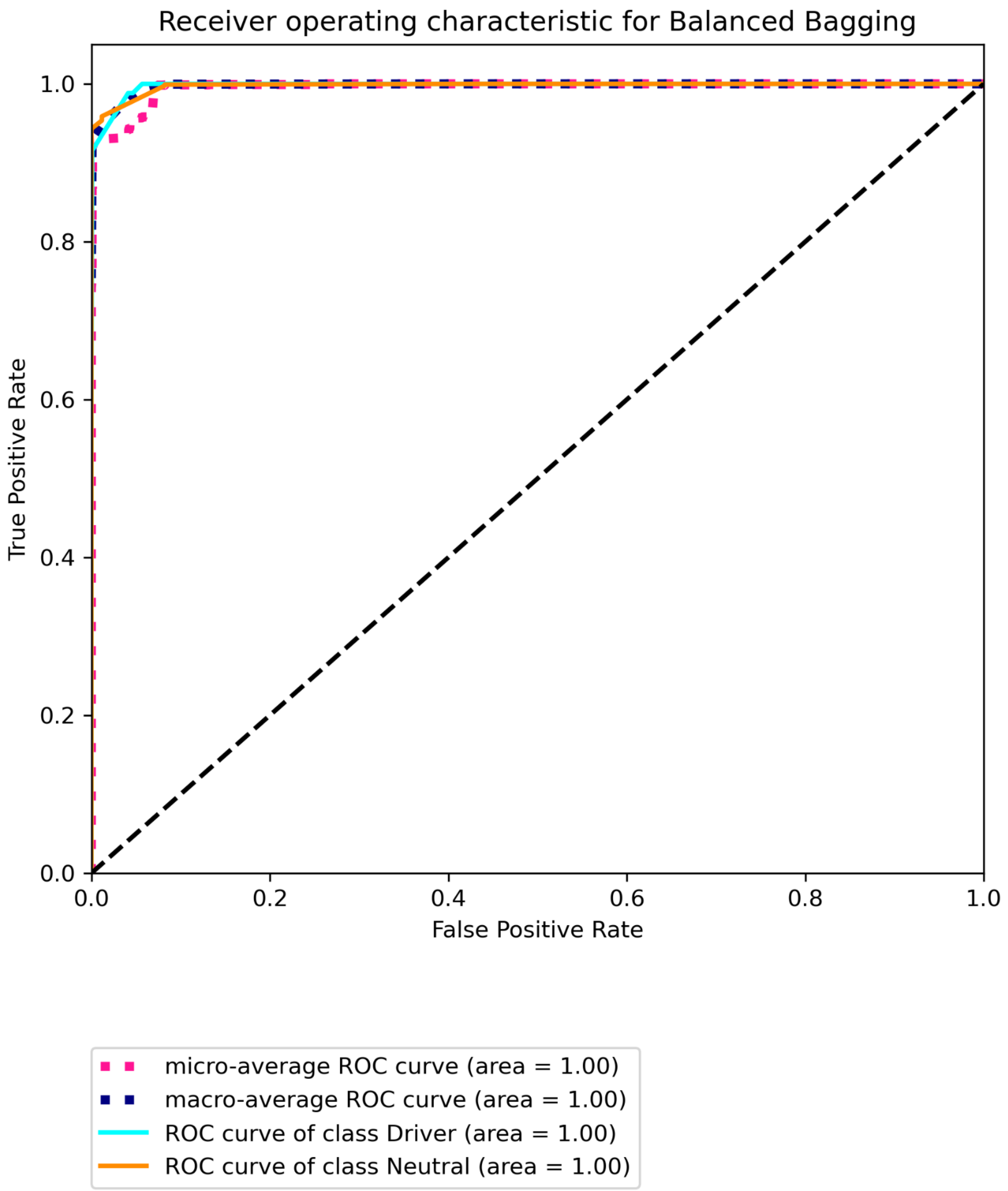

Figure S6C. Receiver operating characteristic for BRCA using SNV data with CIViC labels built on “some” features using Balanced Bagging.

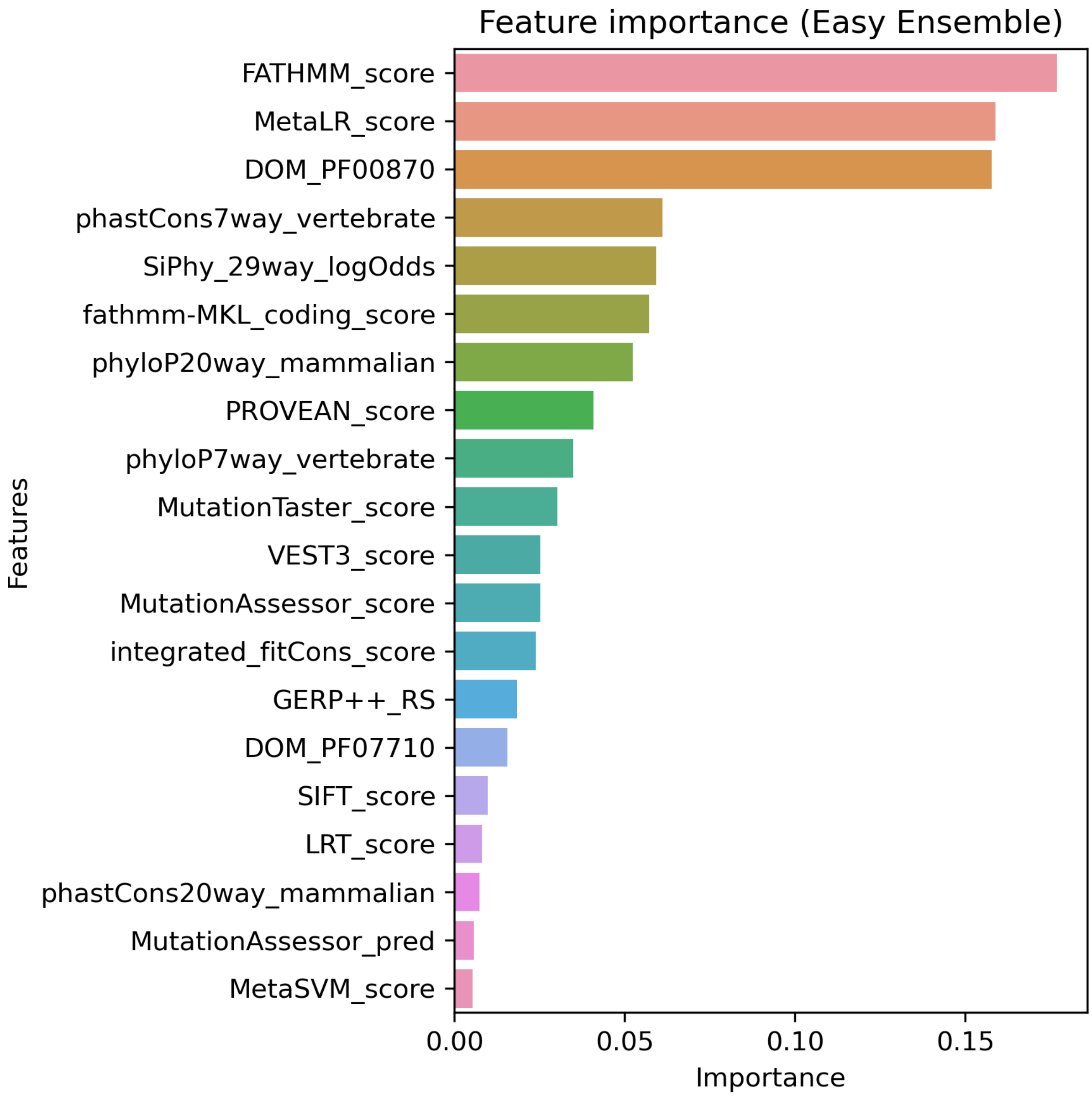

Figure S7A. Top 20 features for BRCA using SNV data with Marellotto *et al.* labels built on "all" features using Easy Ensemble.

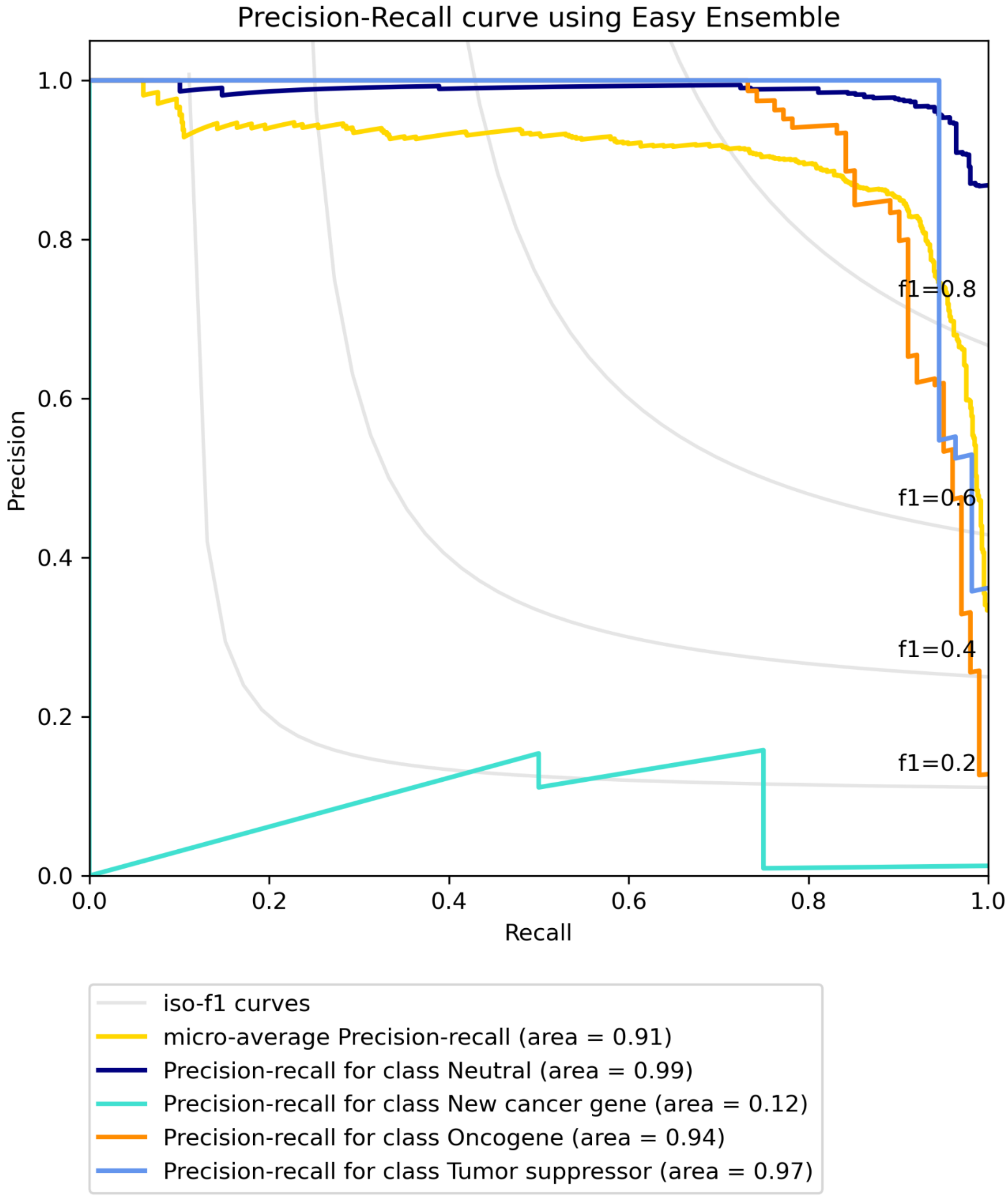

Figure S7B. Precision-recall curve for BRCA using SNV data with Marellotto *et al.* labels built on "all" features using Easy Ensemble.

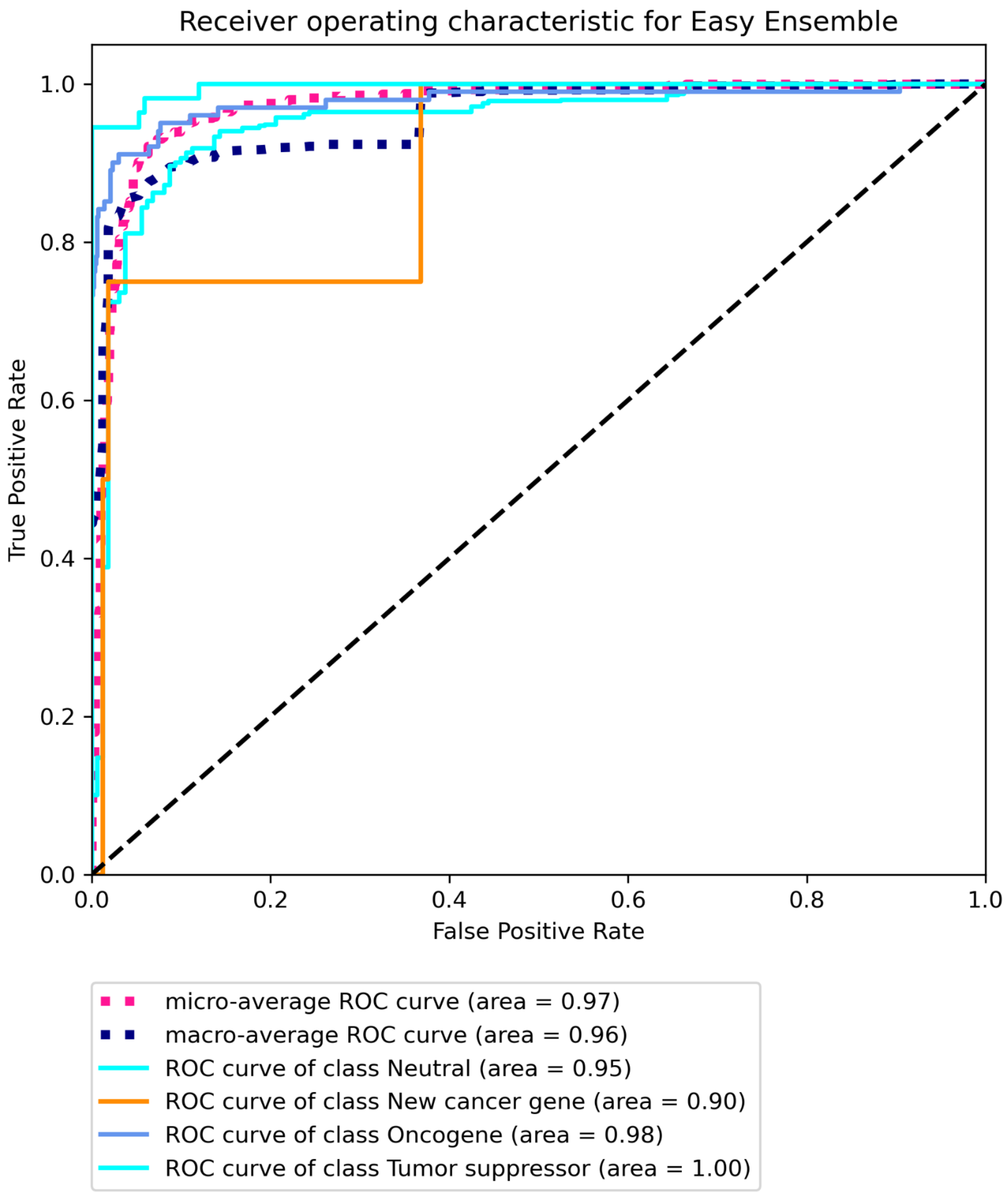

Figure S7C. Receiver operating characteristic for BRCA using SNV data with Marellotto *et al.* labels built on "all" features using Easy Ensemble.

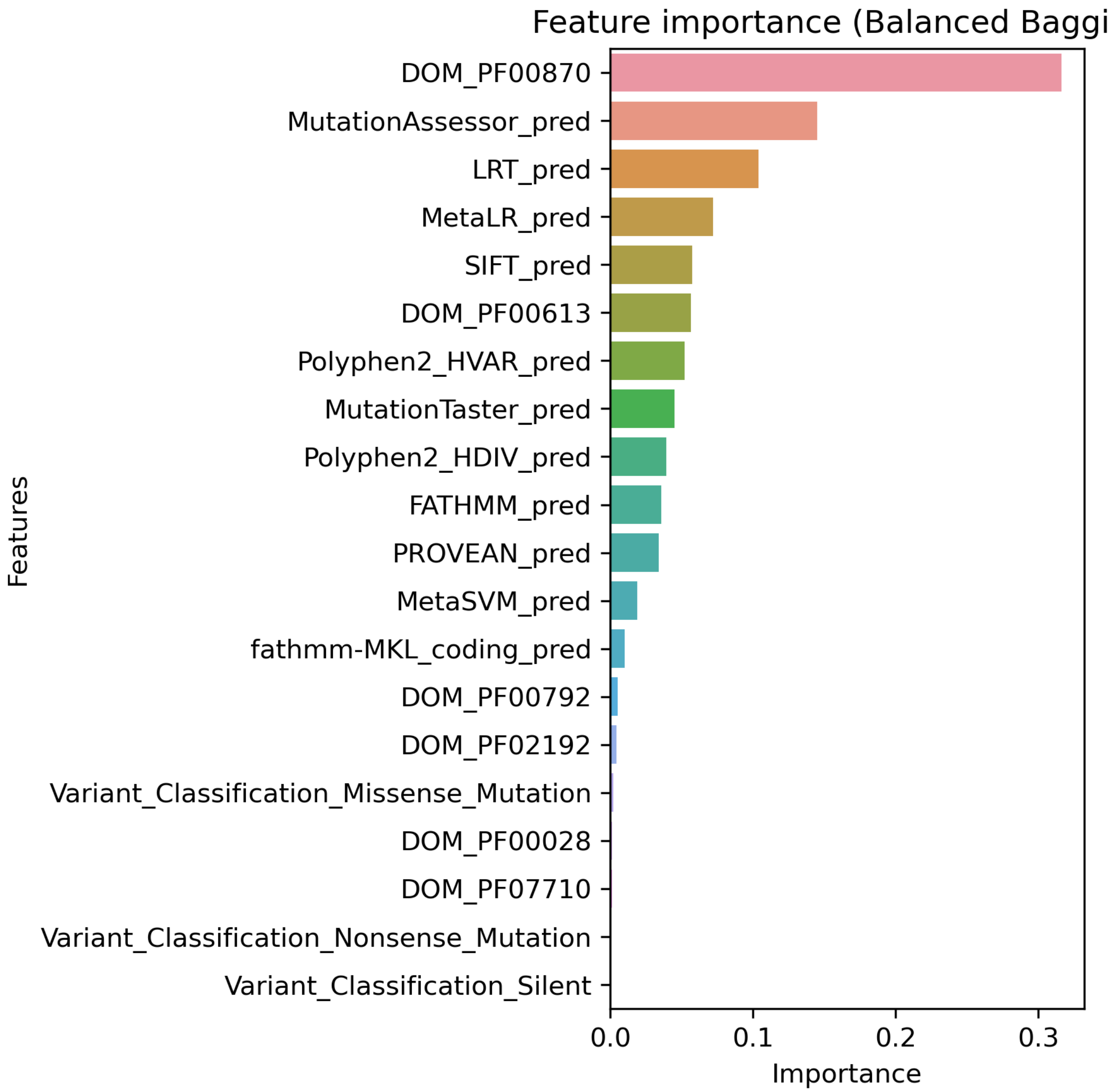

Figure S8A. Top 20 features for BRCA using SNV data with Marellotto *et al.* labels built on "some" features using Balanced Bagging.

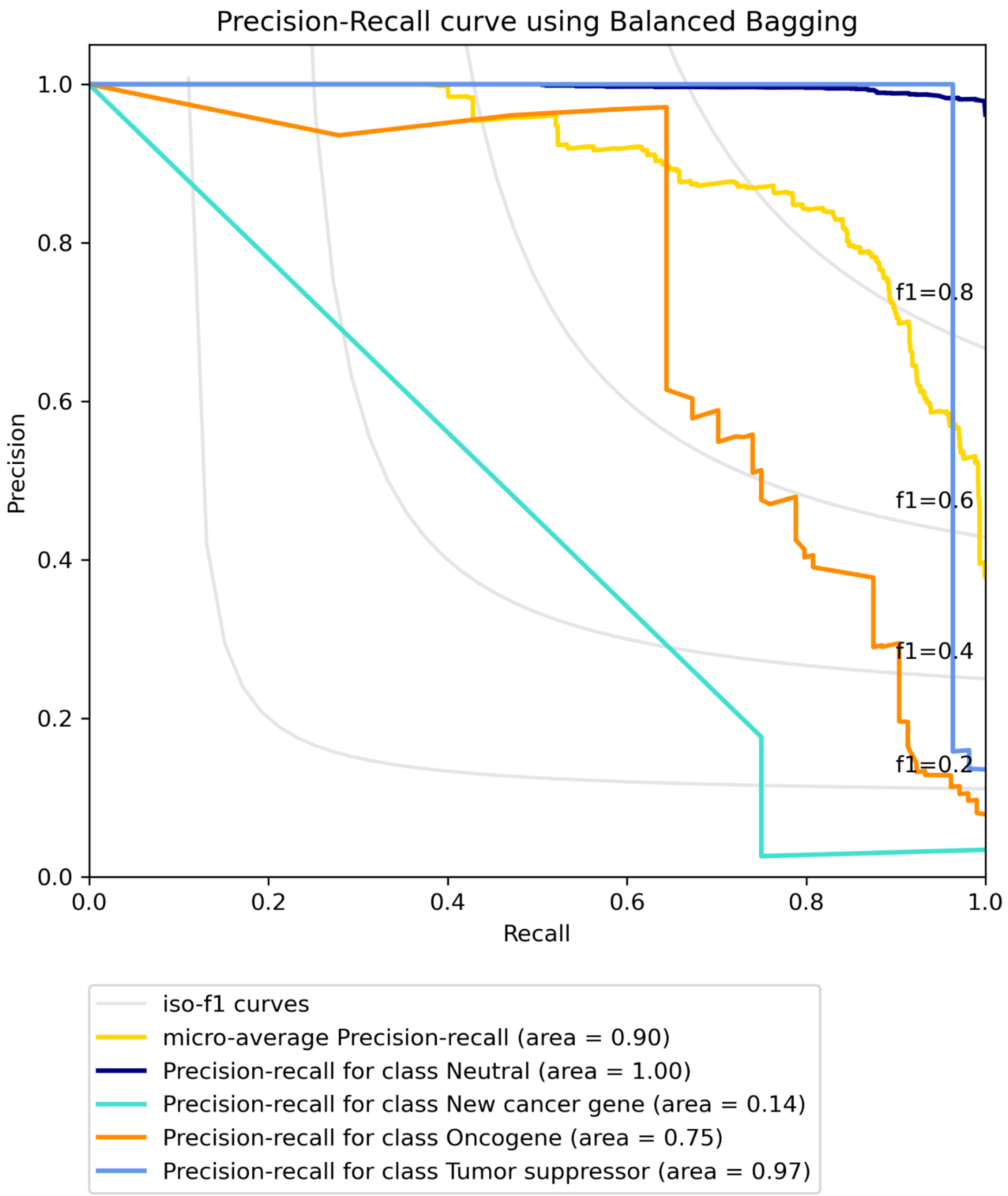

Figure S8B. Precision-recall curve for BRCA using SNV data with Marellotto *et al.* labels built on “some” features using Balanced Bagging.

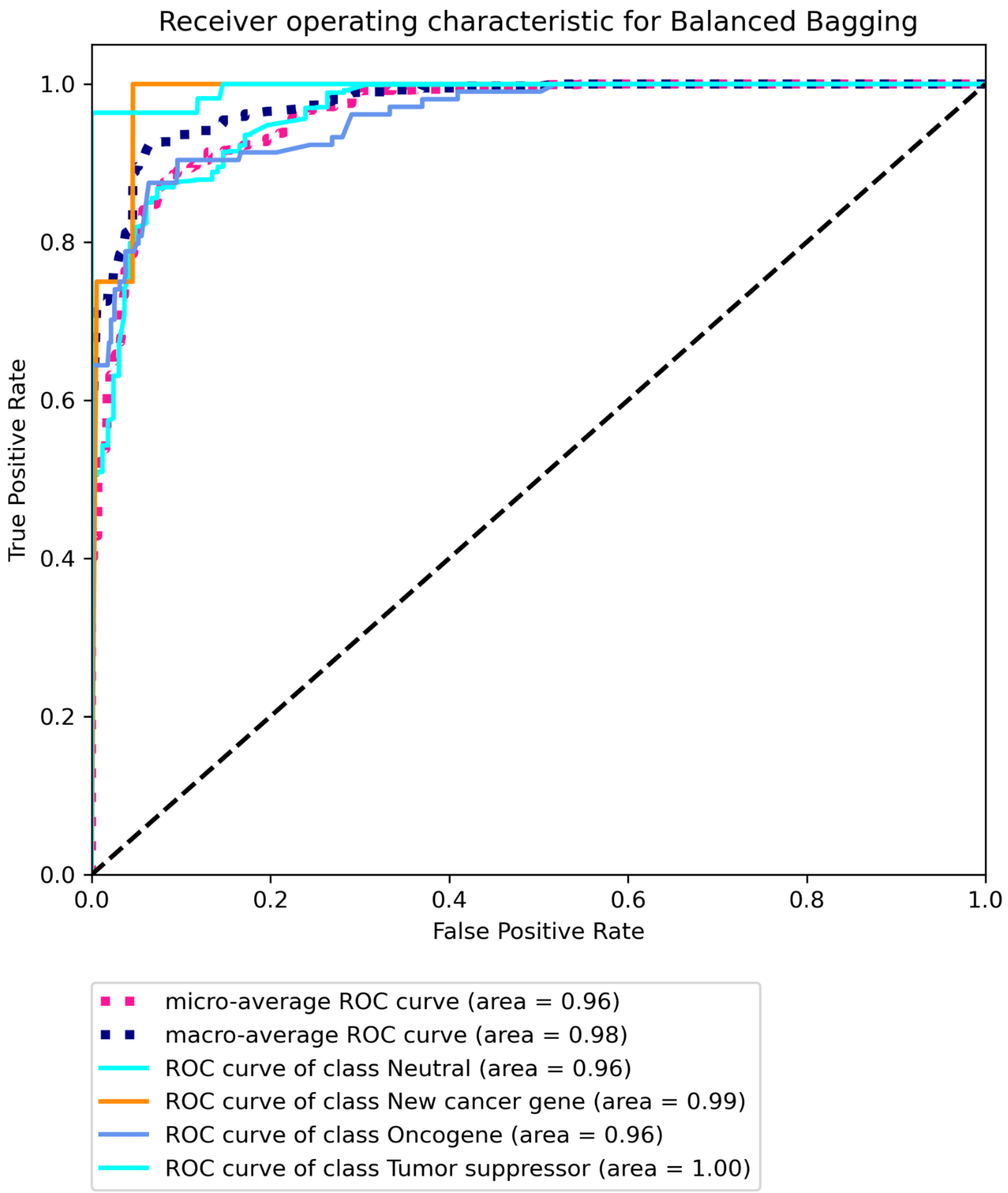

Figure S8C. Receiver operating characteristic for BRCA using SNV data with Marellotto *et al.* labels built on "some" features using Balanced Bagging.

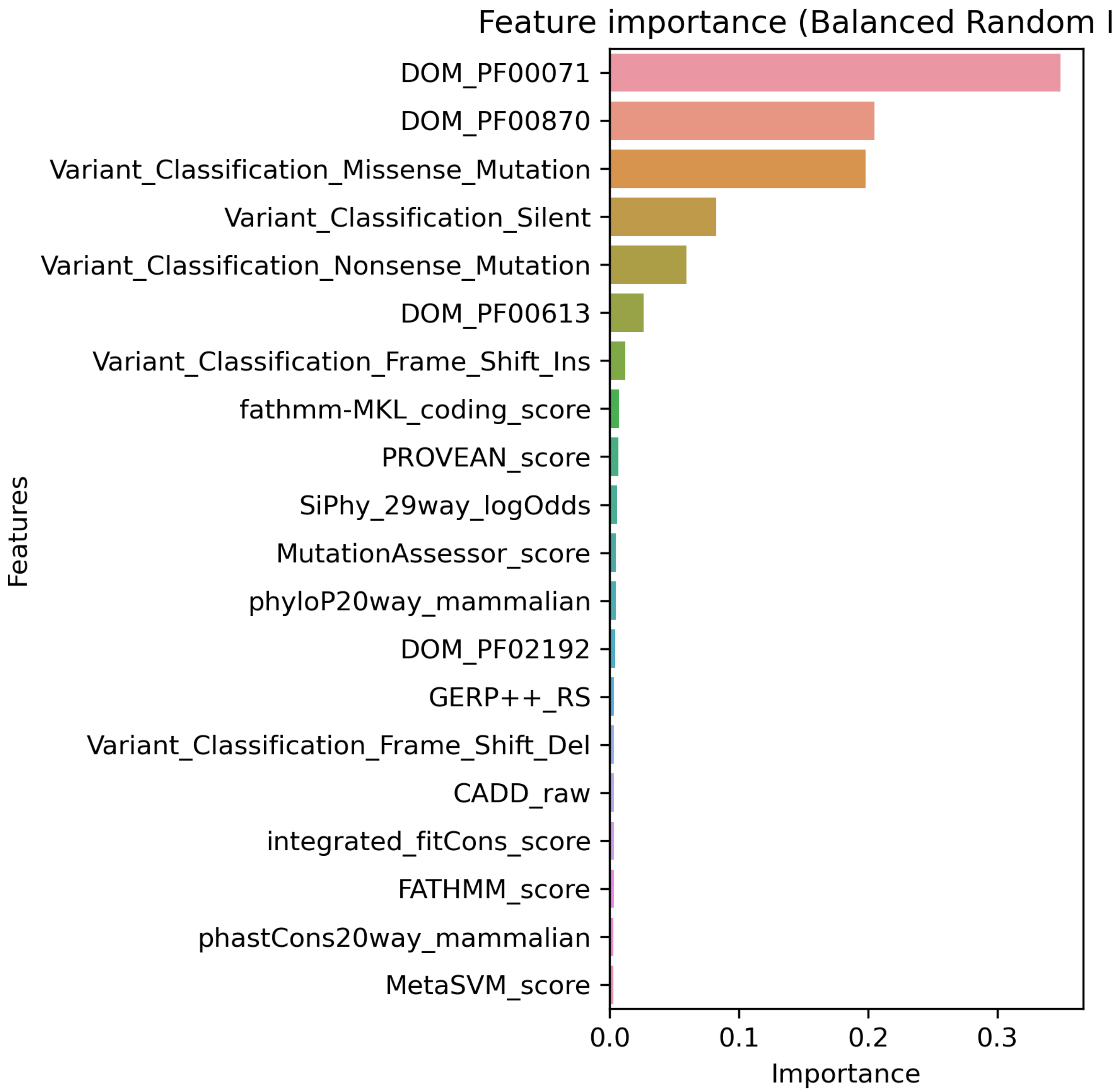

Figure S9A. Top 20 features for COAD using SNV data with Bailey *et al.* labels built on "all" features using Balanced Random Forest.

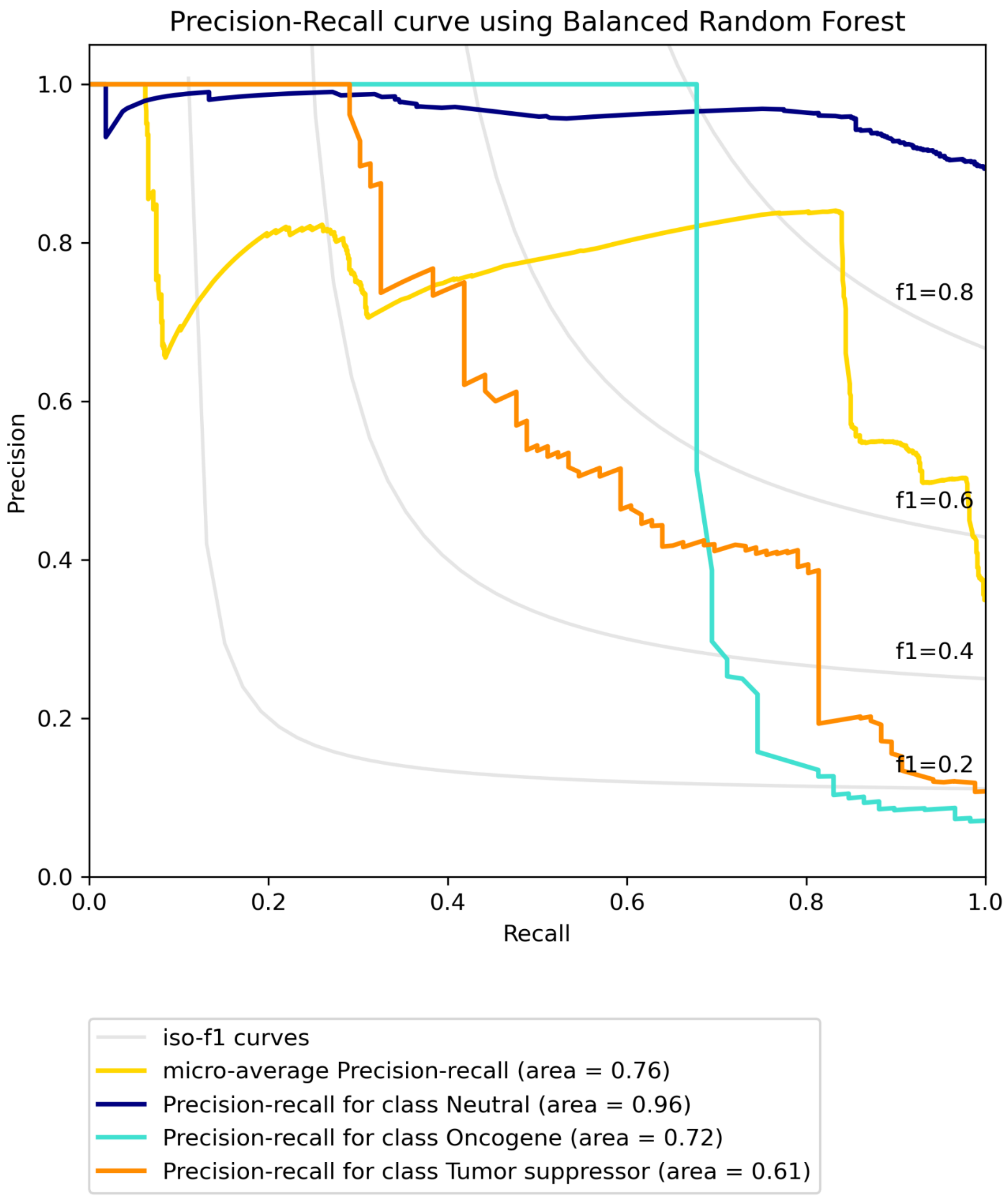

Figure S9B. Precision-recall curve for COAD using SNV data with Bailey *et al.* labels built on "all" features using Balanced Random Forest.

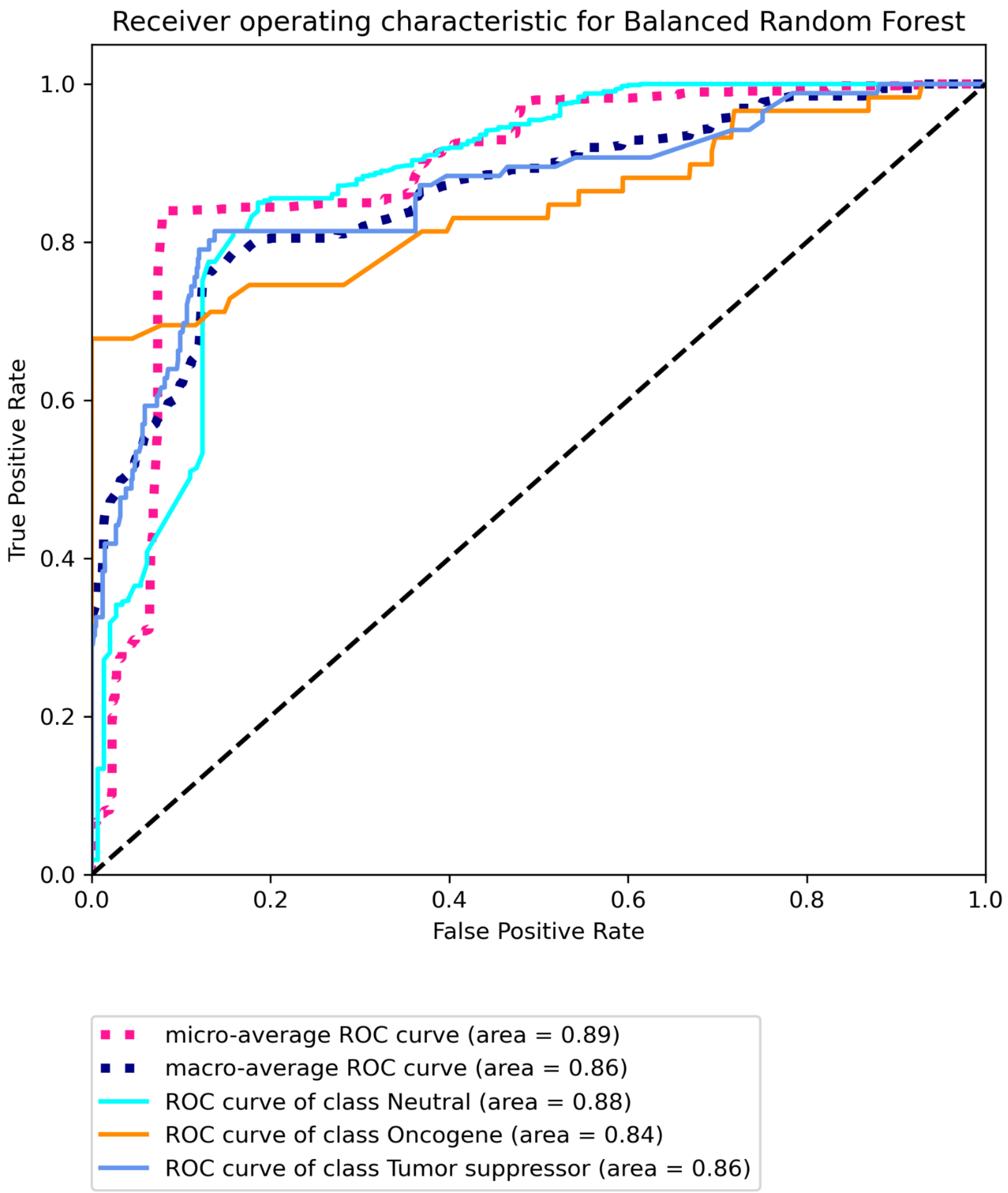

Figure S9C Receiver operating characteristic for COAD using SNV data with Bailey *et al.* labels built on “all” features using Balanced Random Forest.

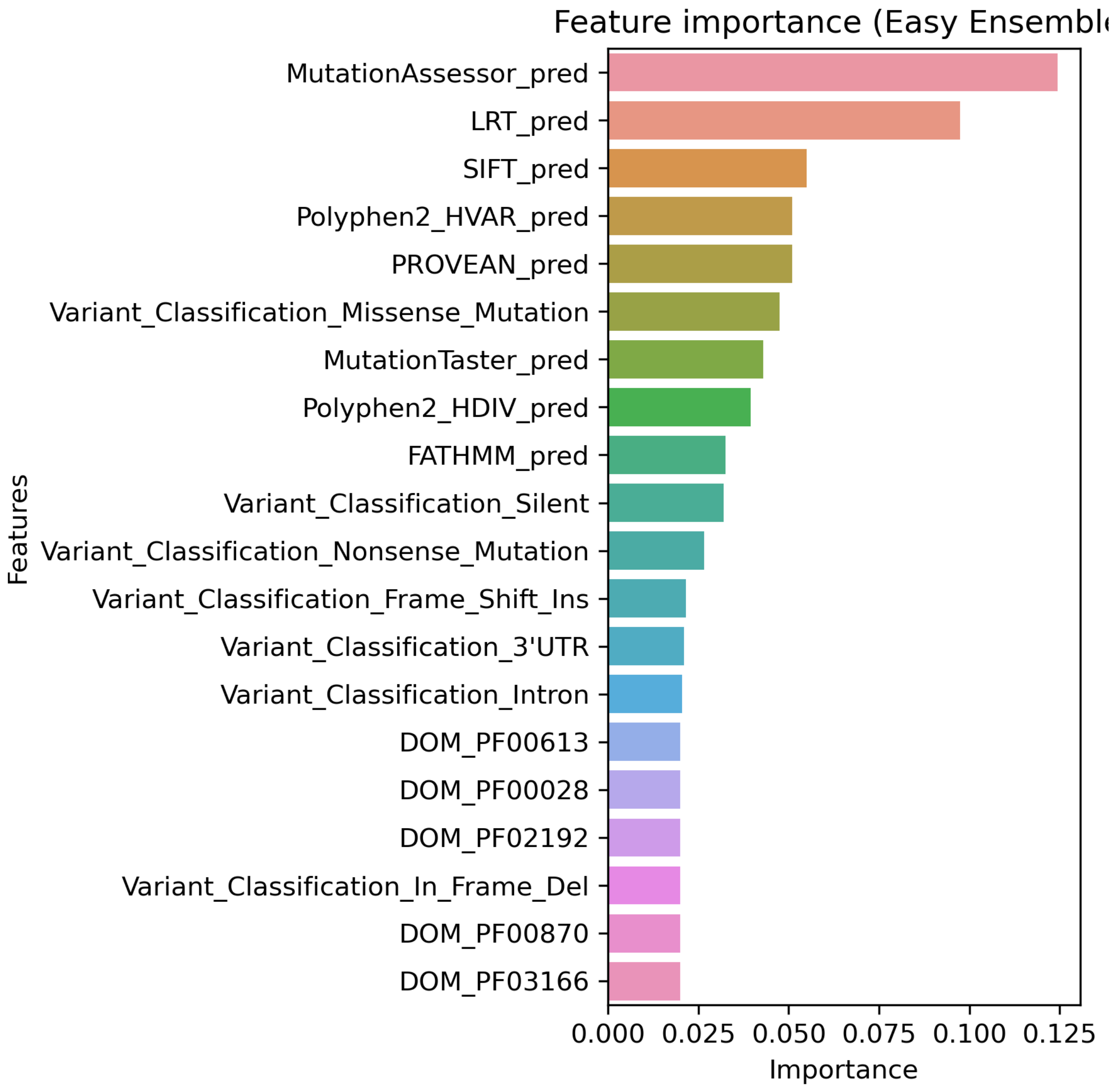

Figure S10A. Top 20 features for COAD using SNV data with Bailey *et al.* labels built on "some" features using Easy Ensemble.

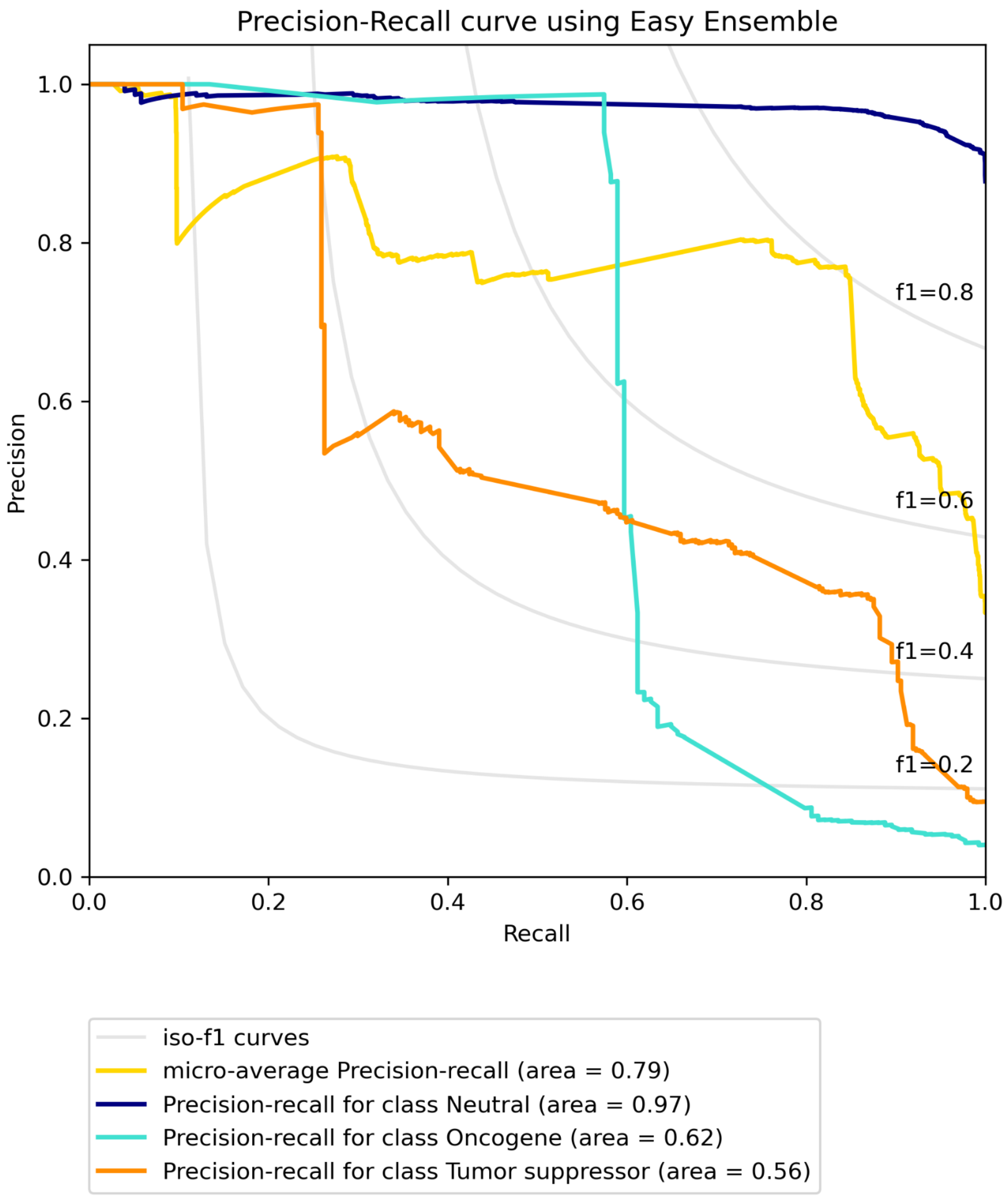

Figure S10B. Precision-recall curve for COAD using SNV data with Bailey *et al.* labels built on "some" features using Easy Ensemble.

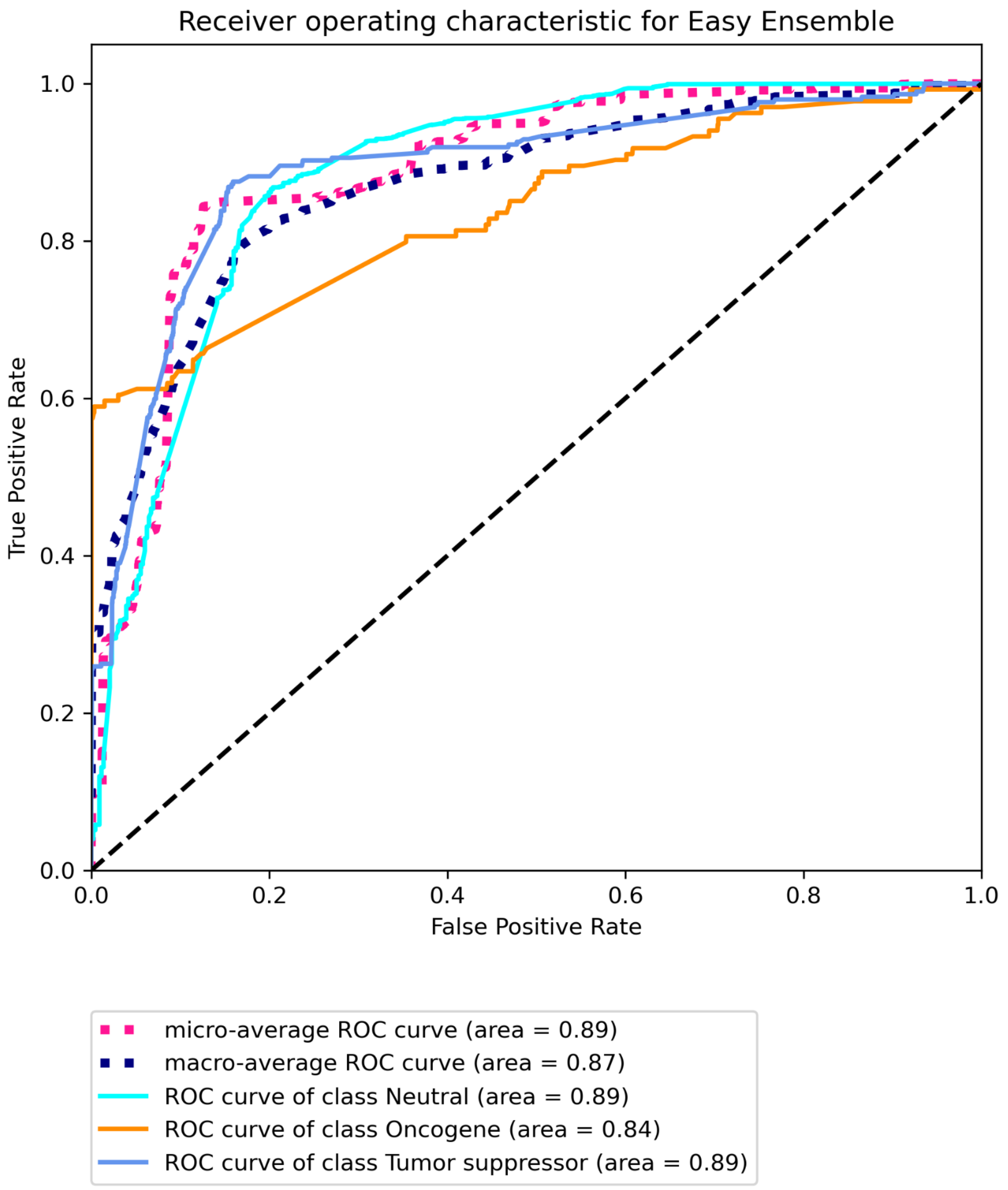

Figure S10C Receiver operating characteristic for COAD using SNV data with Bailey *et al.* labels built on "some" features using Easy Ensemble.

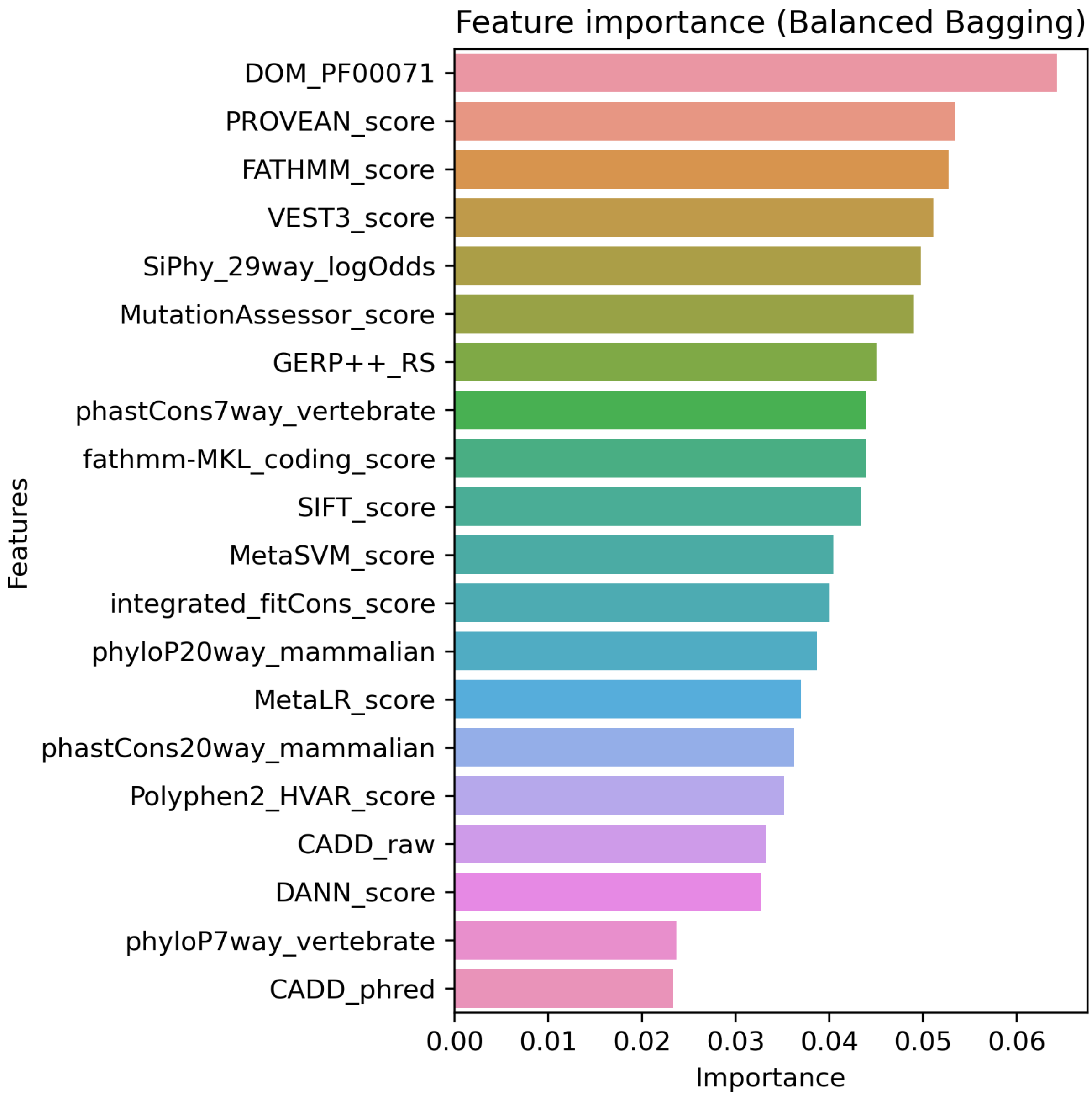

Figure S11A. Top 20 features for COAD using SNV data with CGC labels built on “all” features using Balanced Bagging.

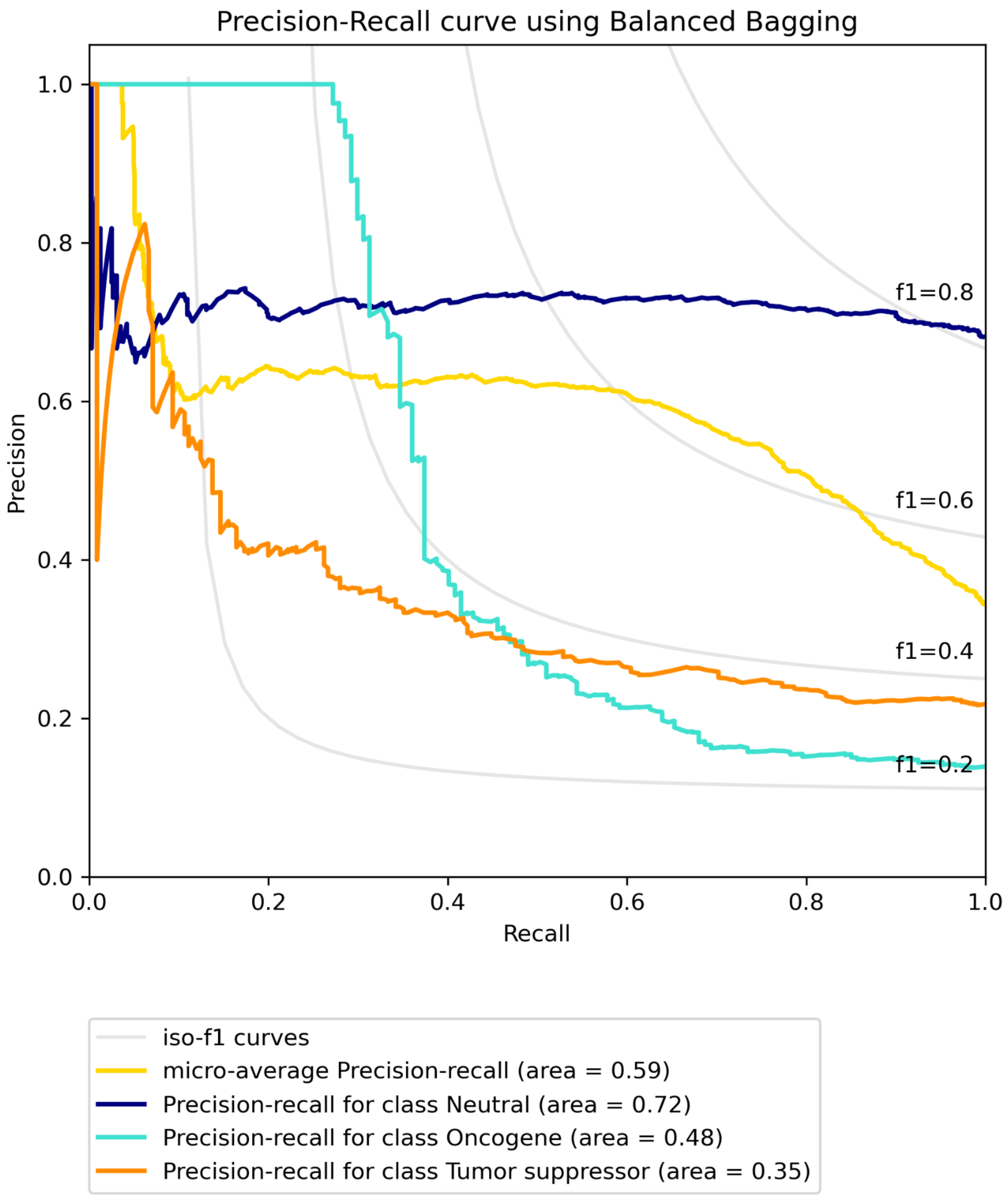

Figure S11B. Precision-recall curve for COAD using SNV data with CGC labels built on "all" features using Balanced Bagging.

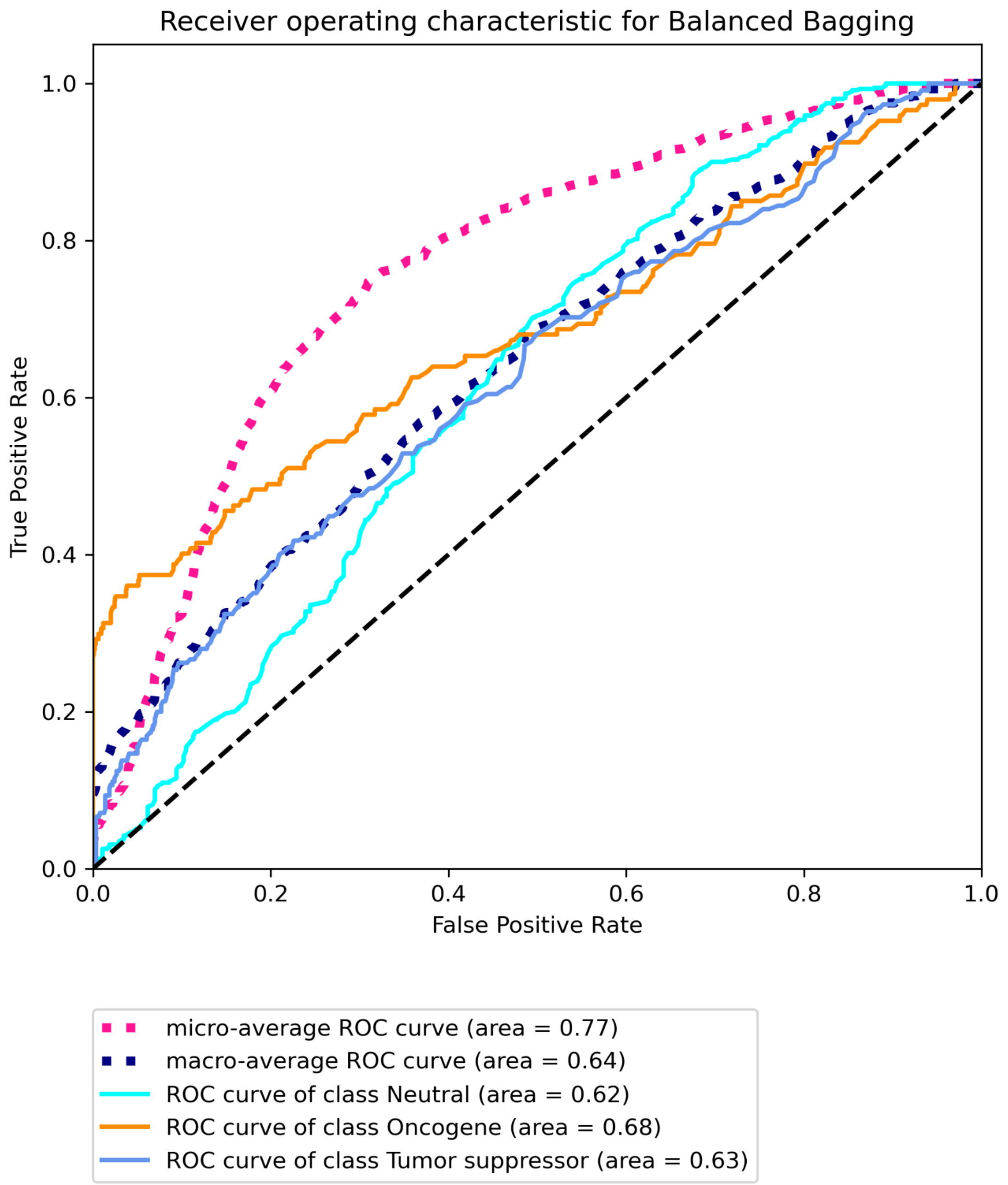

Figure S11C Receiver operating characteristic for COAD using SNV data with CGC labels built on "all" features using Balanced Bagging.

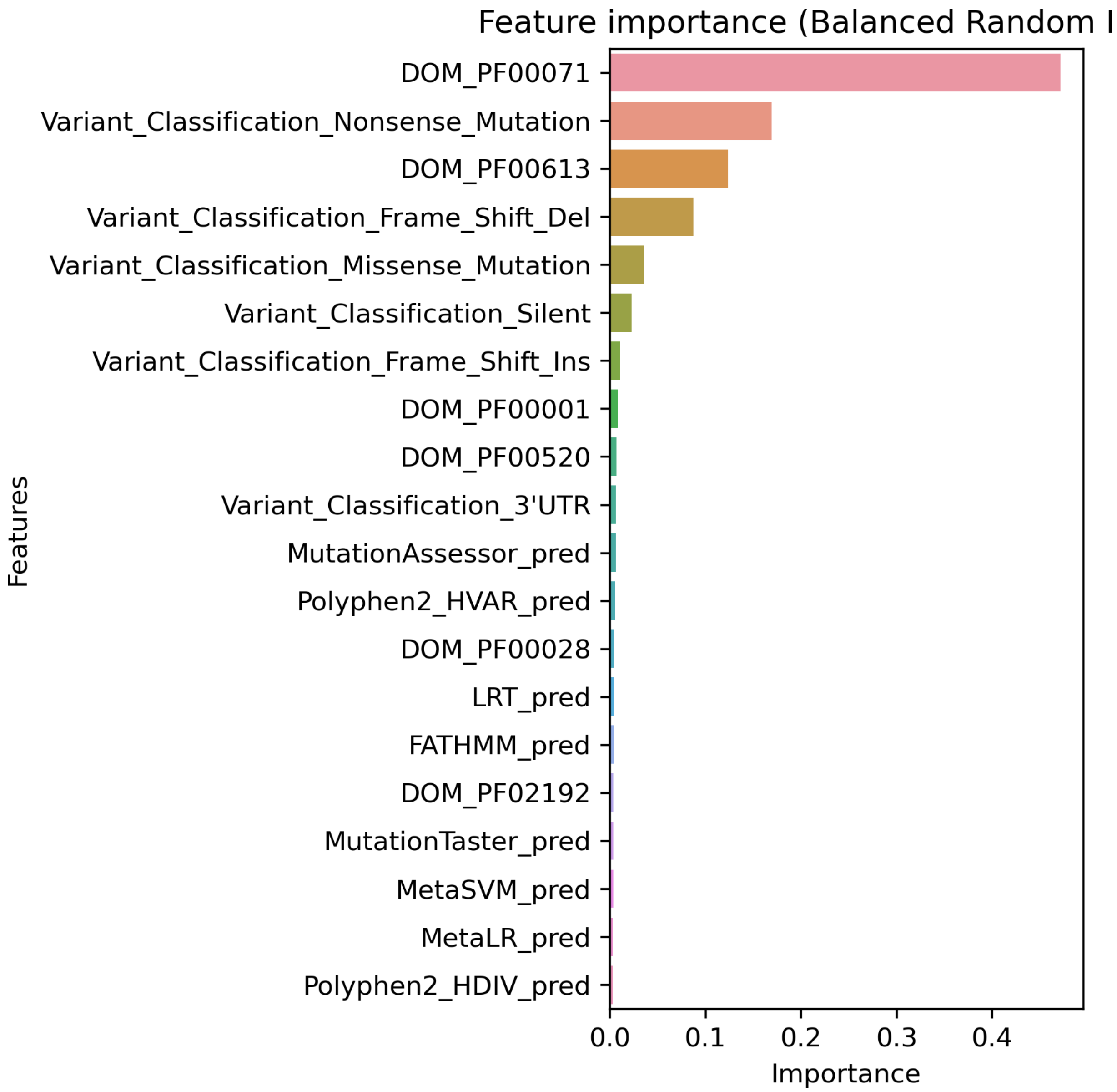

Figure S12A. Top 20 features for COAD using SNV data with CGC labels built on "some" features using Balanced Random Forest.

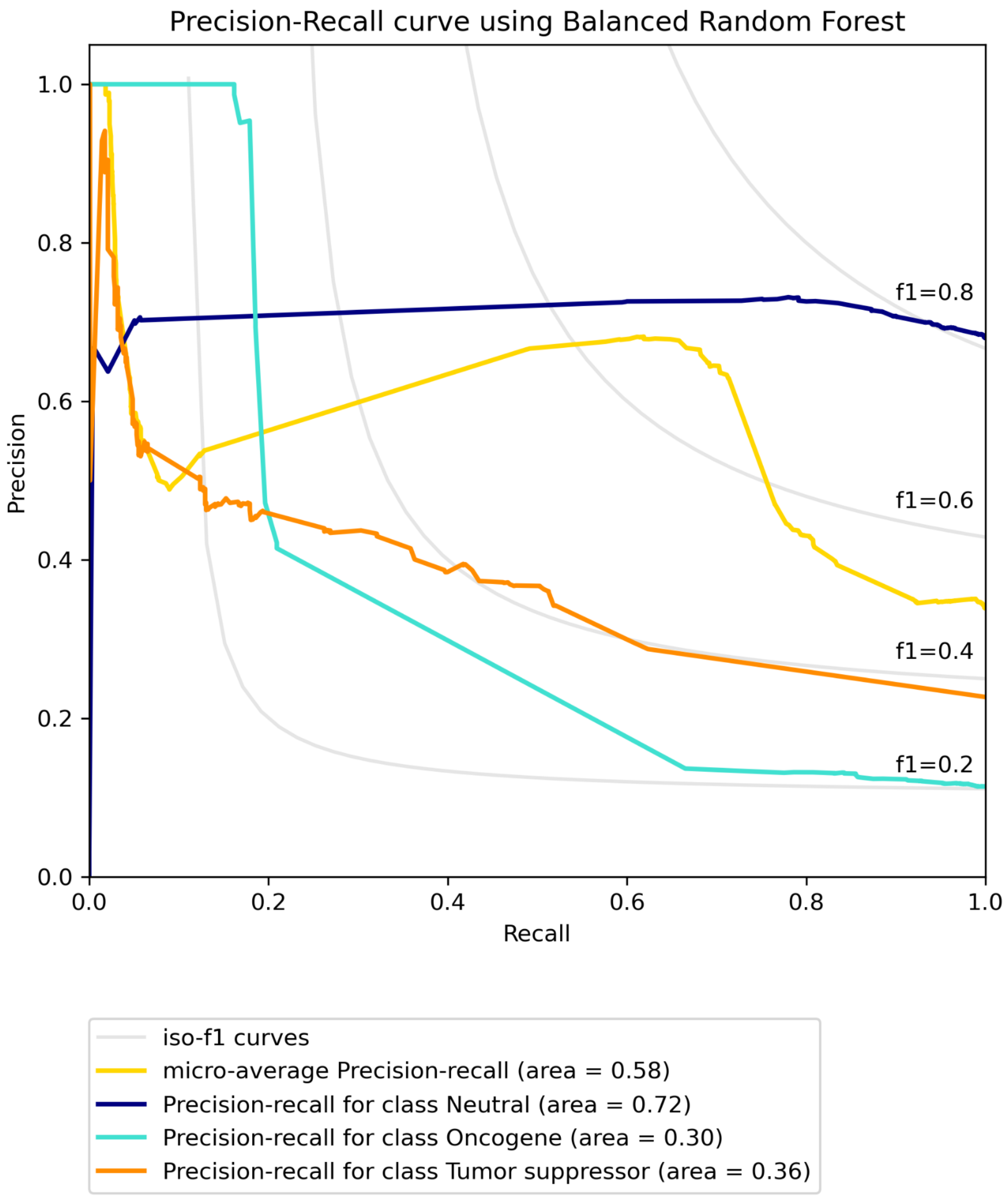

Figure S12B. Precision-recall curve for COAD using SNV data with CGC labels built on "some" features using Balanced Random Forest.

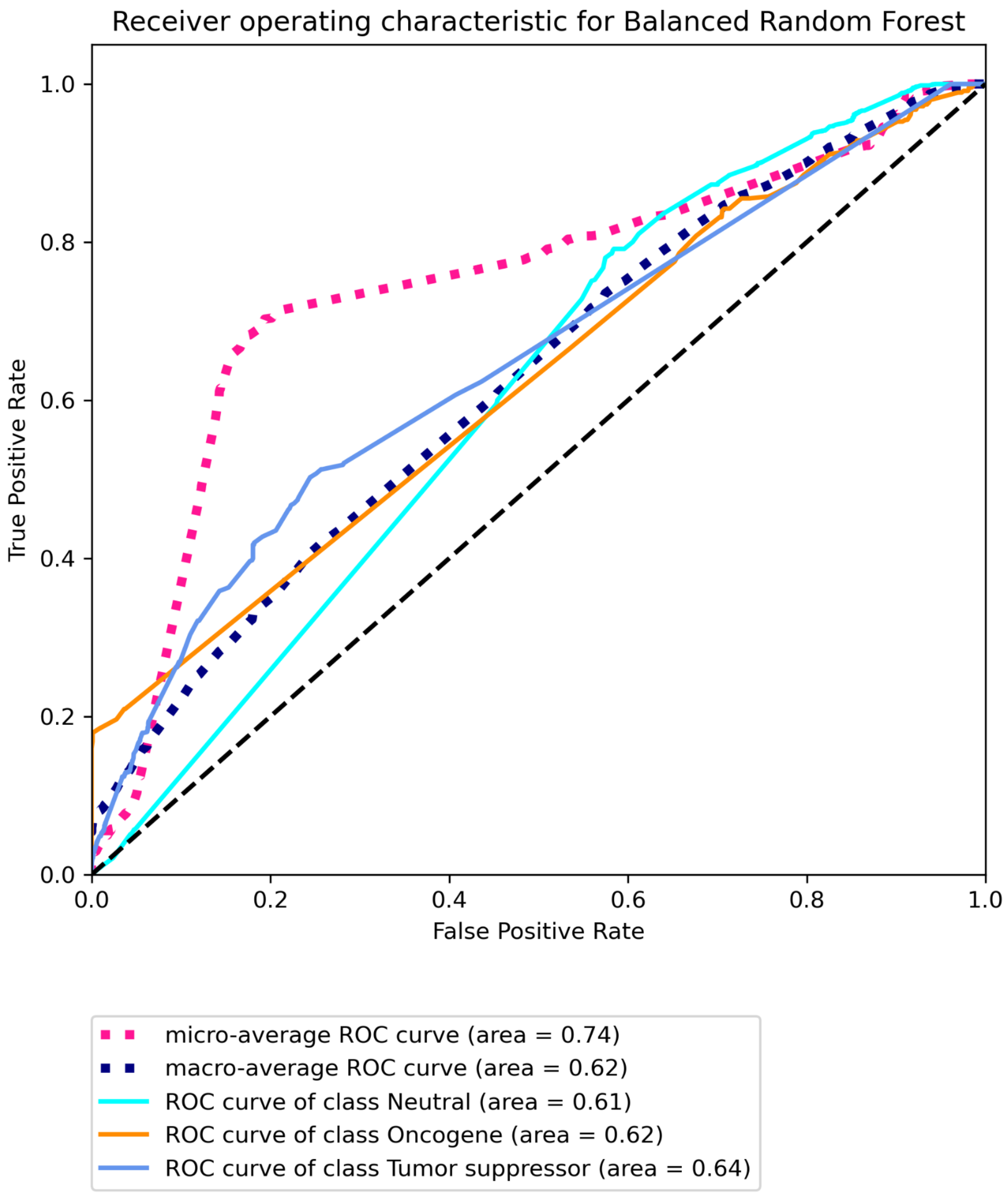

Figure S12C Receiver operating characteristic for COAD using SNV data with CGC labels built on "some" features using Balanced Random Forest.

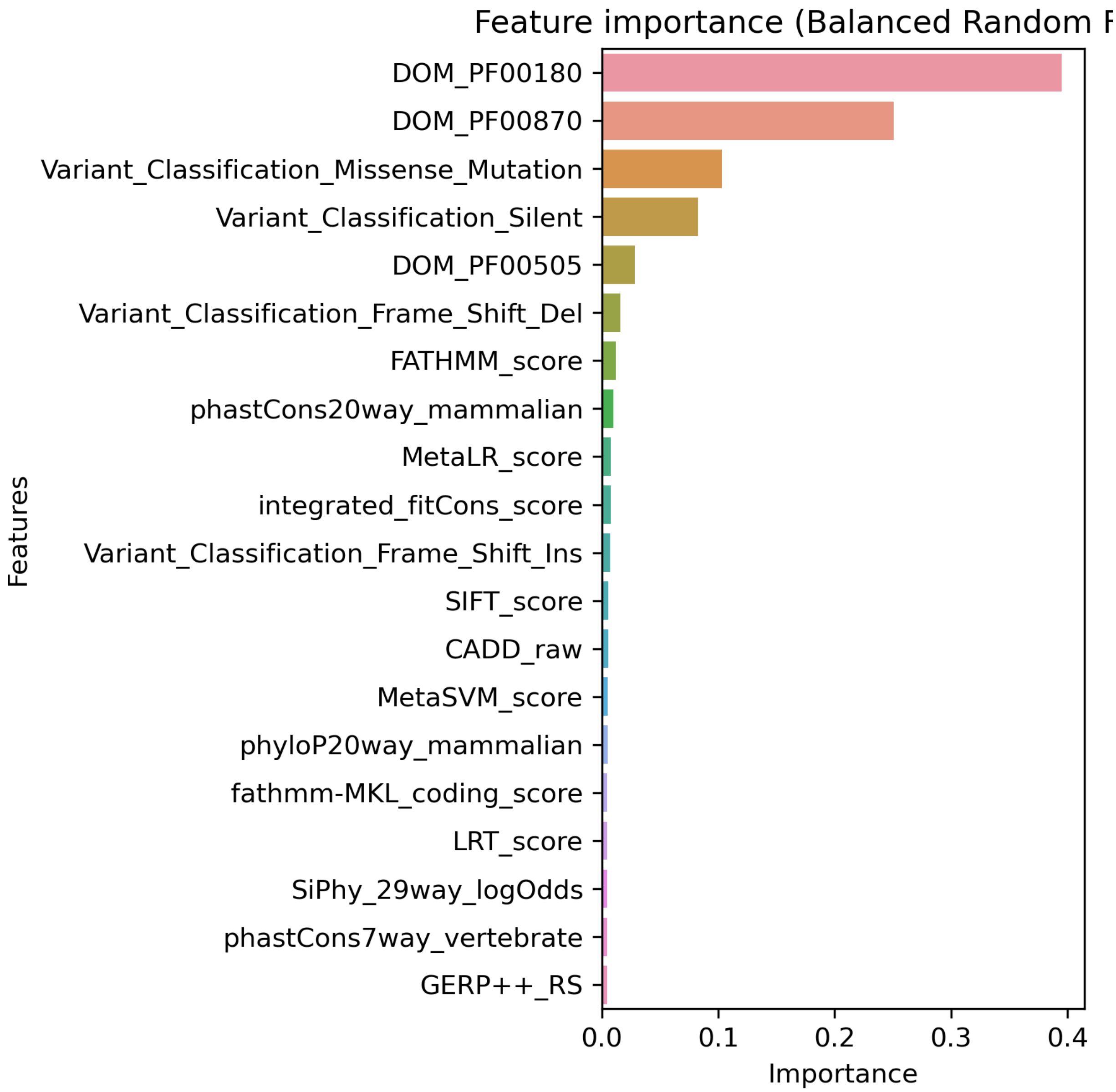

Figure S13A. Top 20 features for LGG using SNV data with Bailey *et al.* labels built on "all" features using Balanced Random Forest.

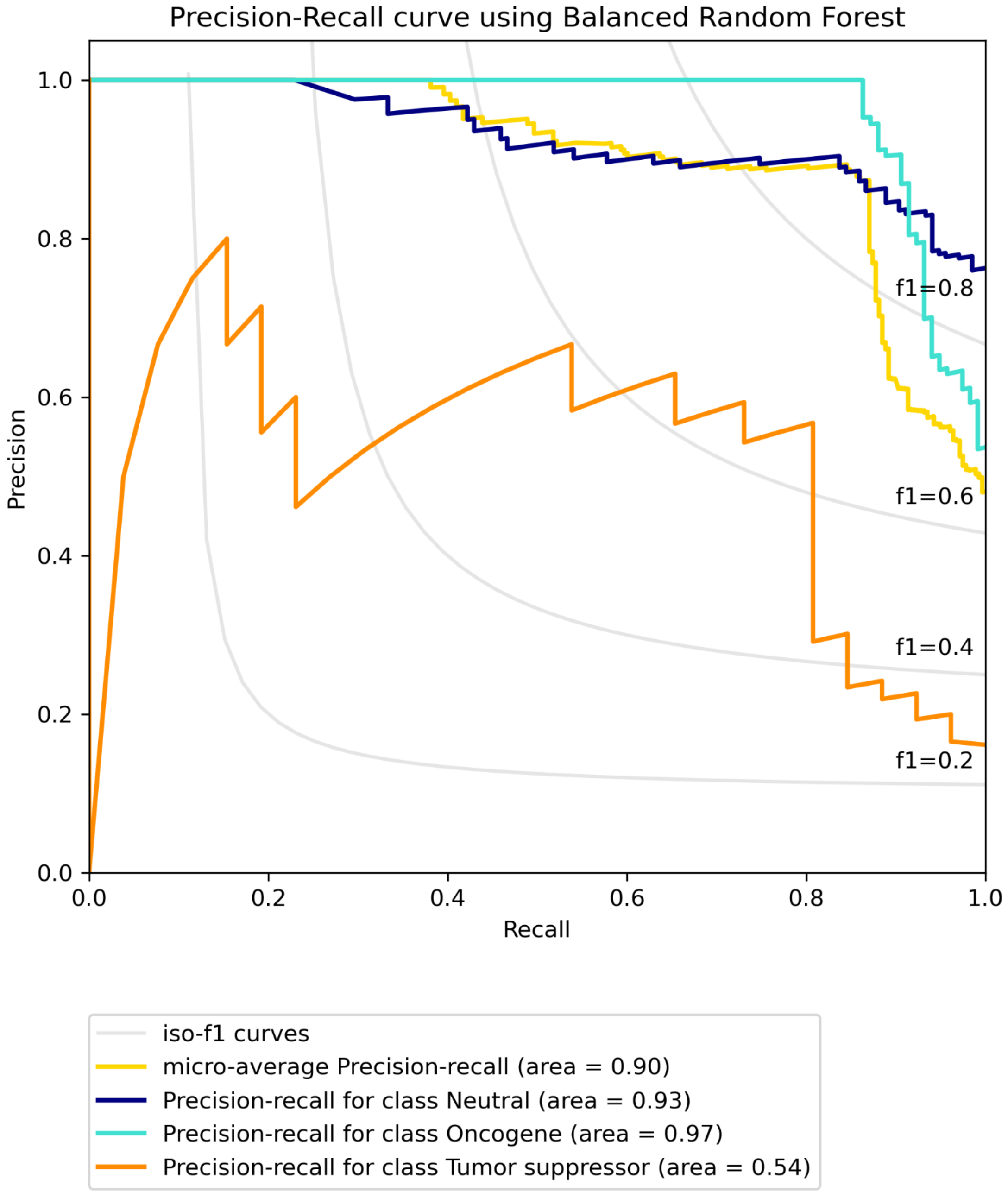

Figure S13B. Precision-recall curve for LGG using SNV data with Bailey *et al.* labels built on "all" features using Balanced Random Forest.

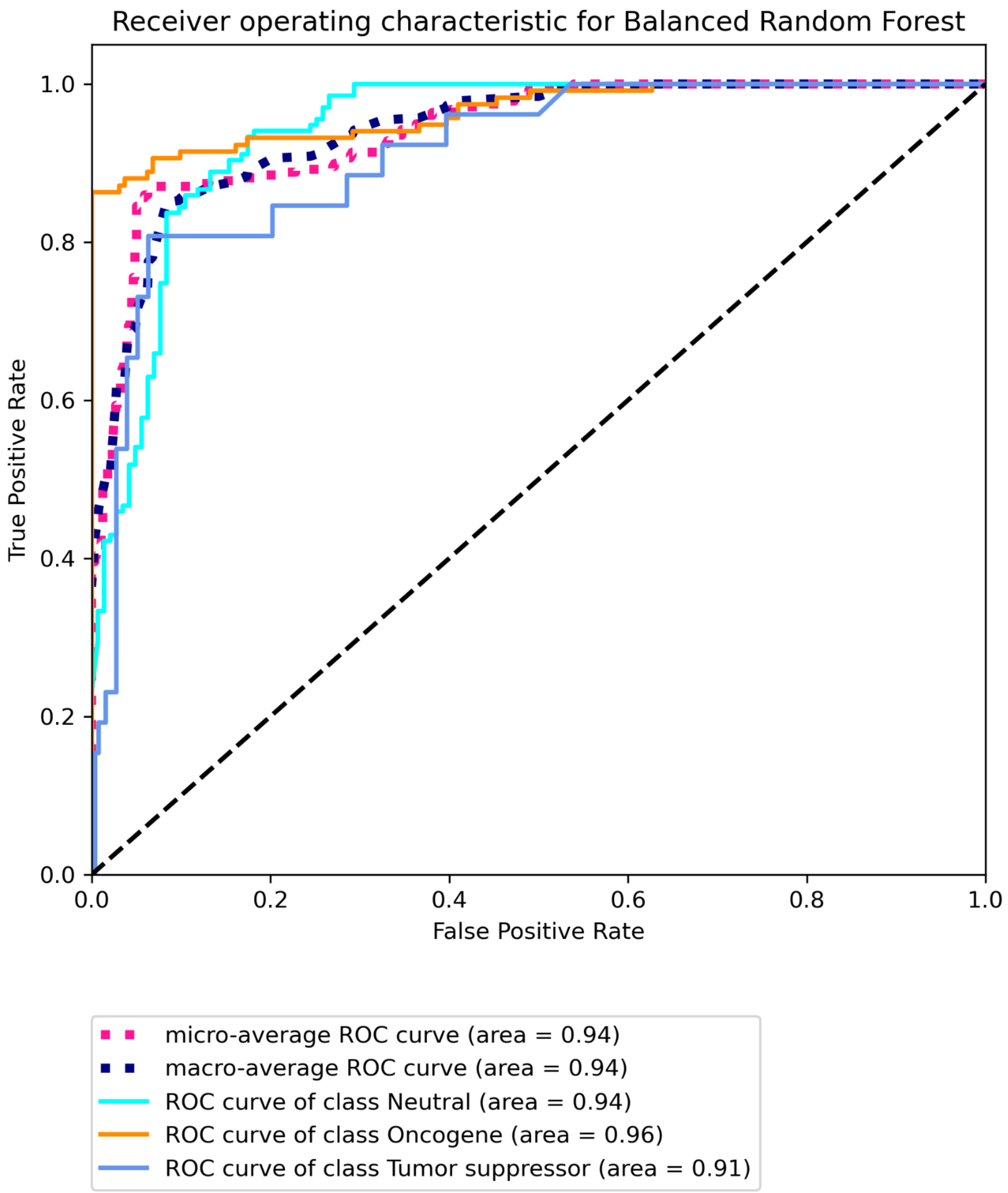

Figure S13C Receiver operating characteristic for LGG using SNV data with Bailey *et al.* labels built on "all" features using Balanced Random Forest.

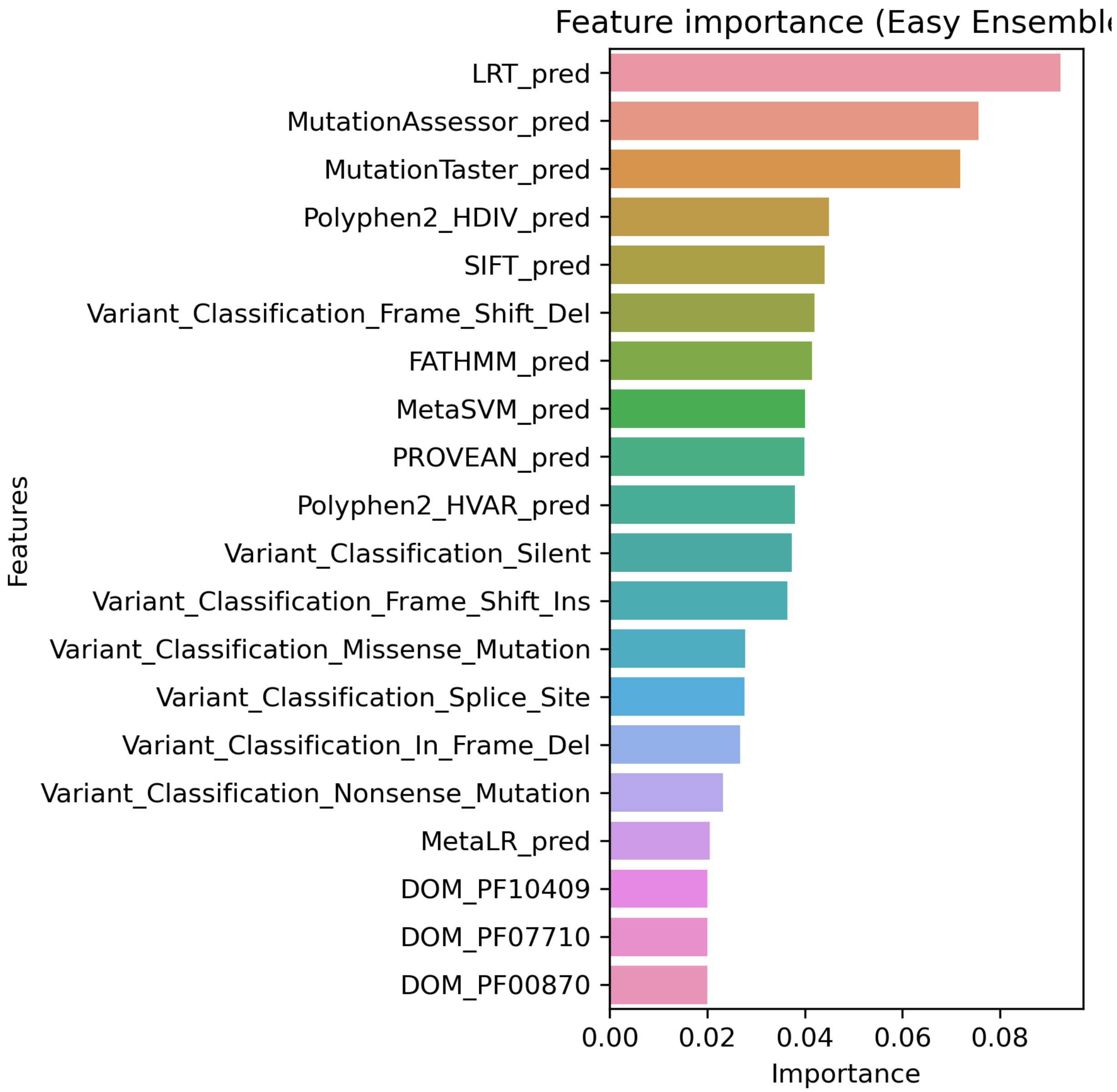

Figure S14A. Top 20 features for LGG using SNV data with Bailey *et al.* labels built on "some" features using Easy Ensemble.

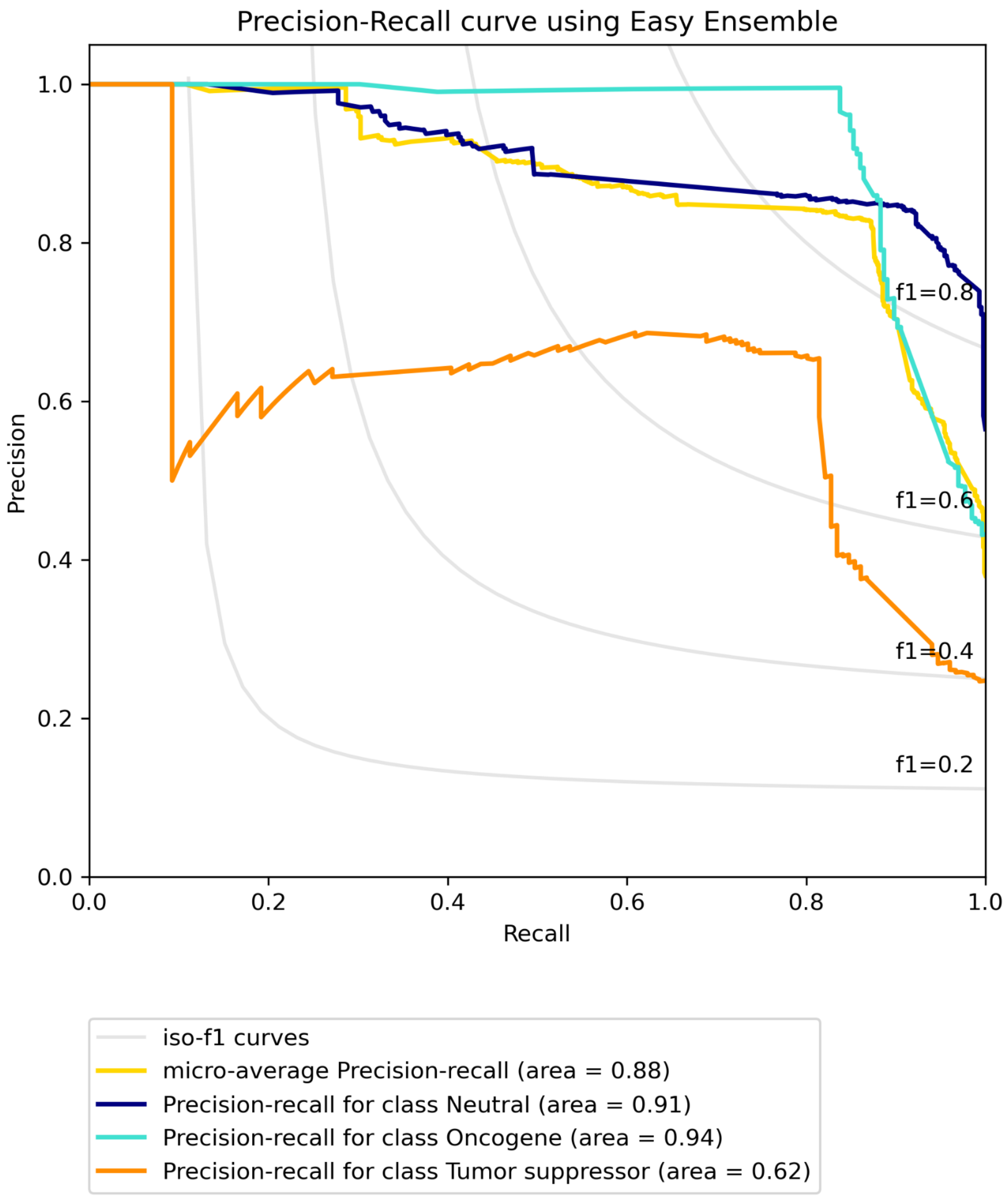

Figure S14B. Precision-recall curve for LGG using SNV data with Bailey *et al.* labels built on "some" features using Easy Ensemble.

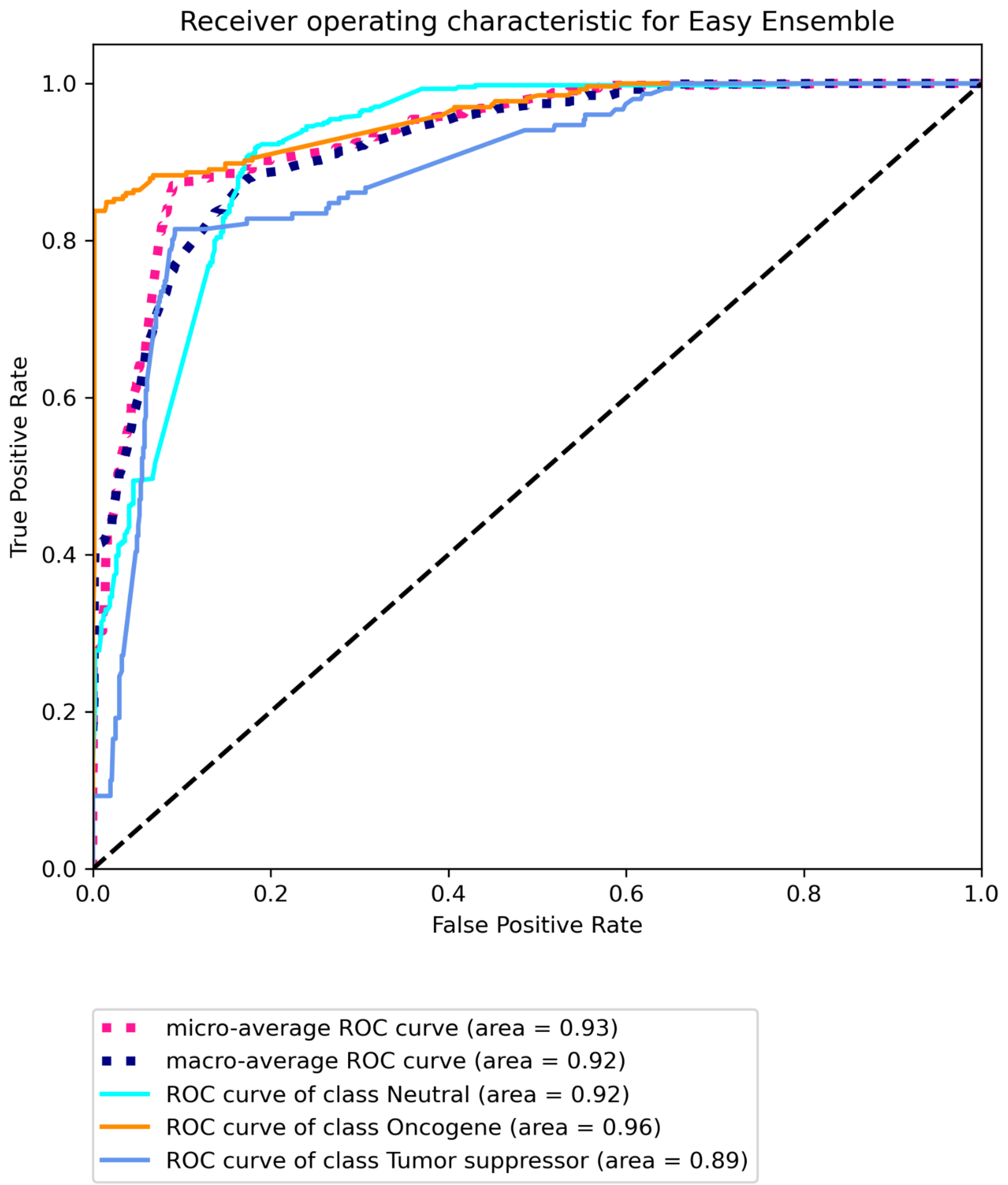

Figure S14C Receiver operating characteristic for LGG using SNV data with Bailey *et al.* labels built on "some" features using Easy Ensemble.

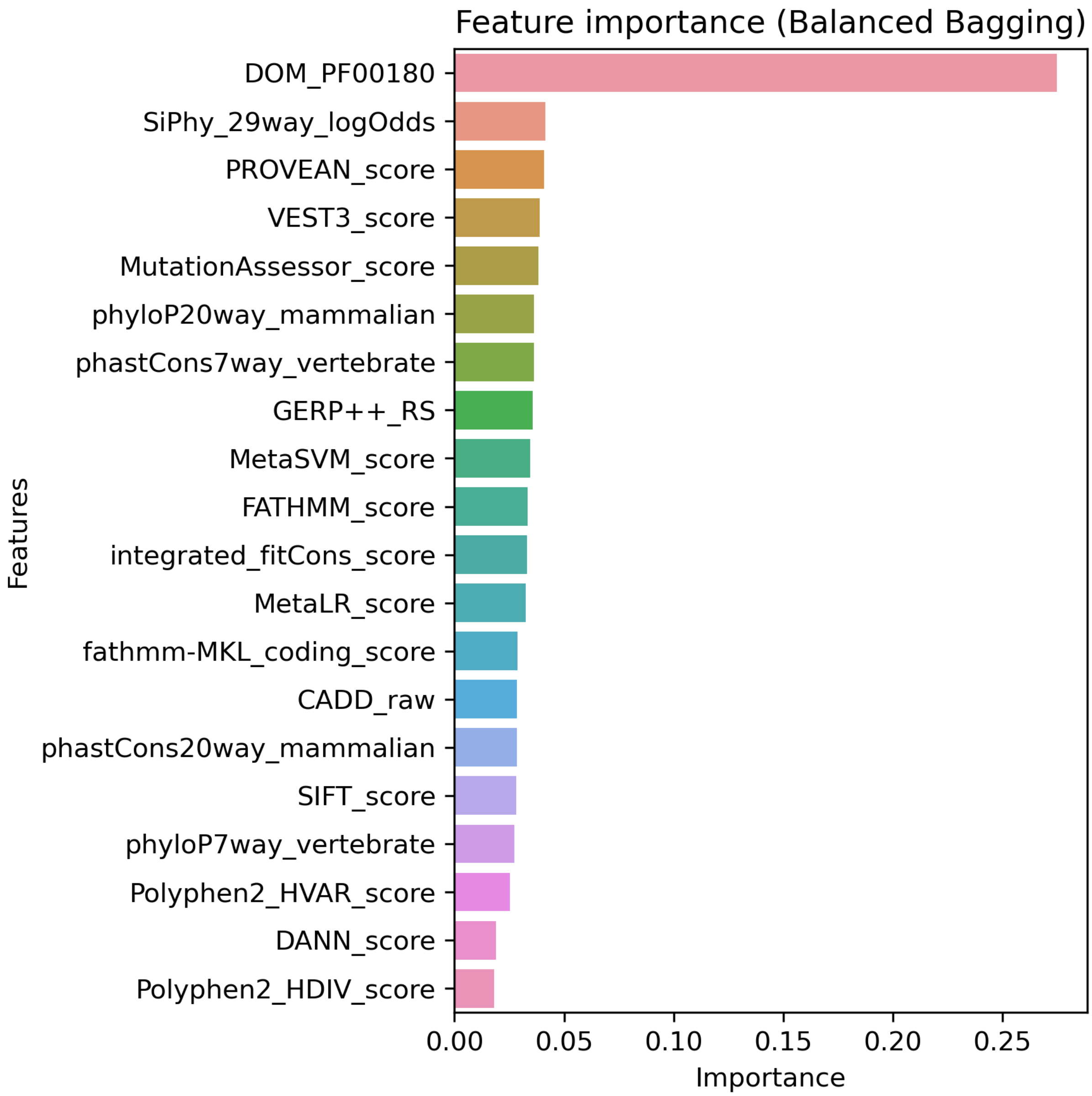

Figure S15A. Top 20 features for LGG using SNV data with CGC labels built on “all” features using Balanced Bagging.

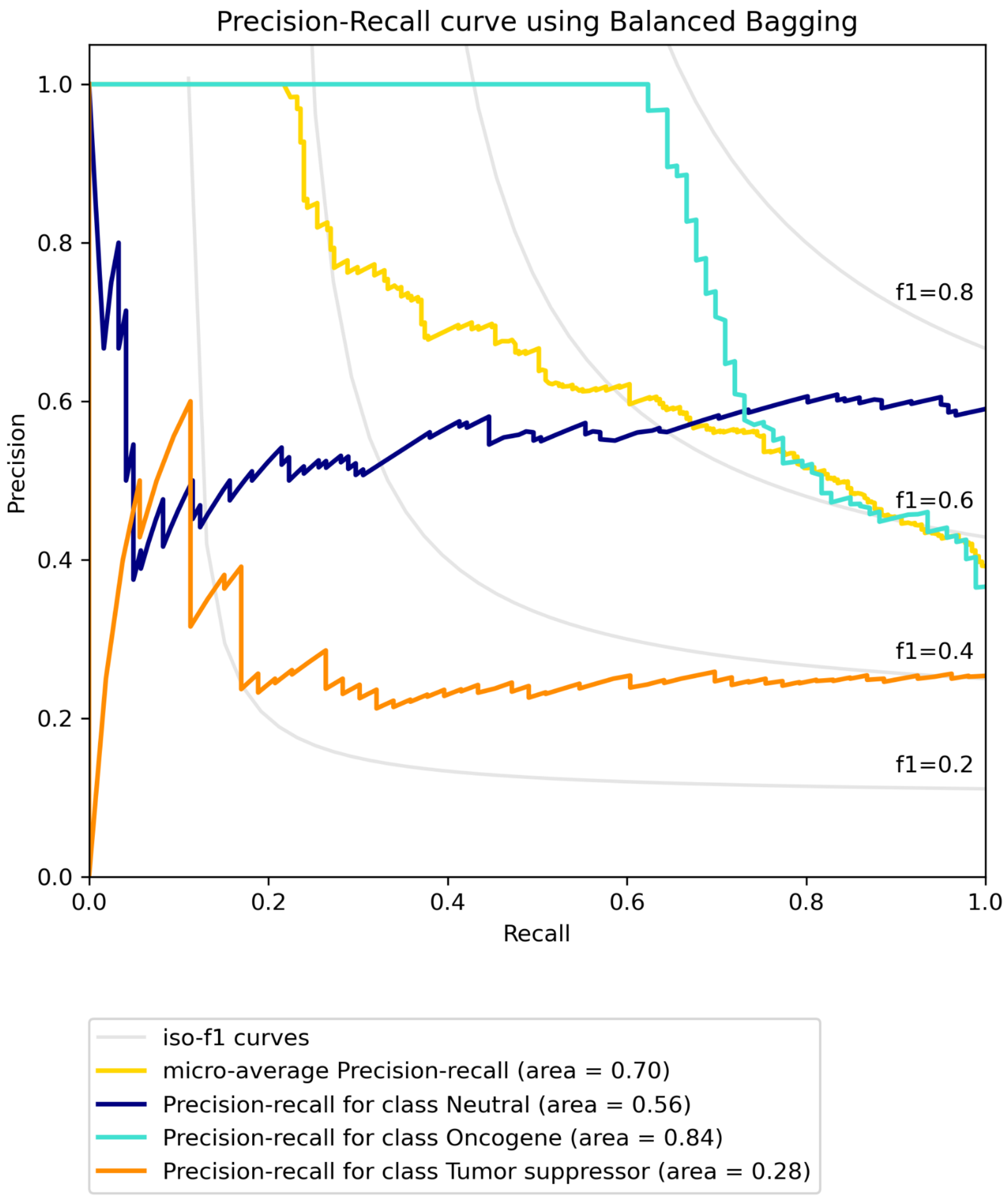

Figure S15B. Precision-recall curve for LGG using SNV data with CGC labels built on "all" features using Balanced Bagging.

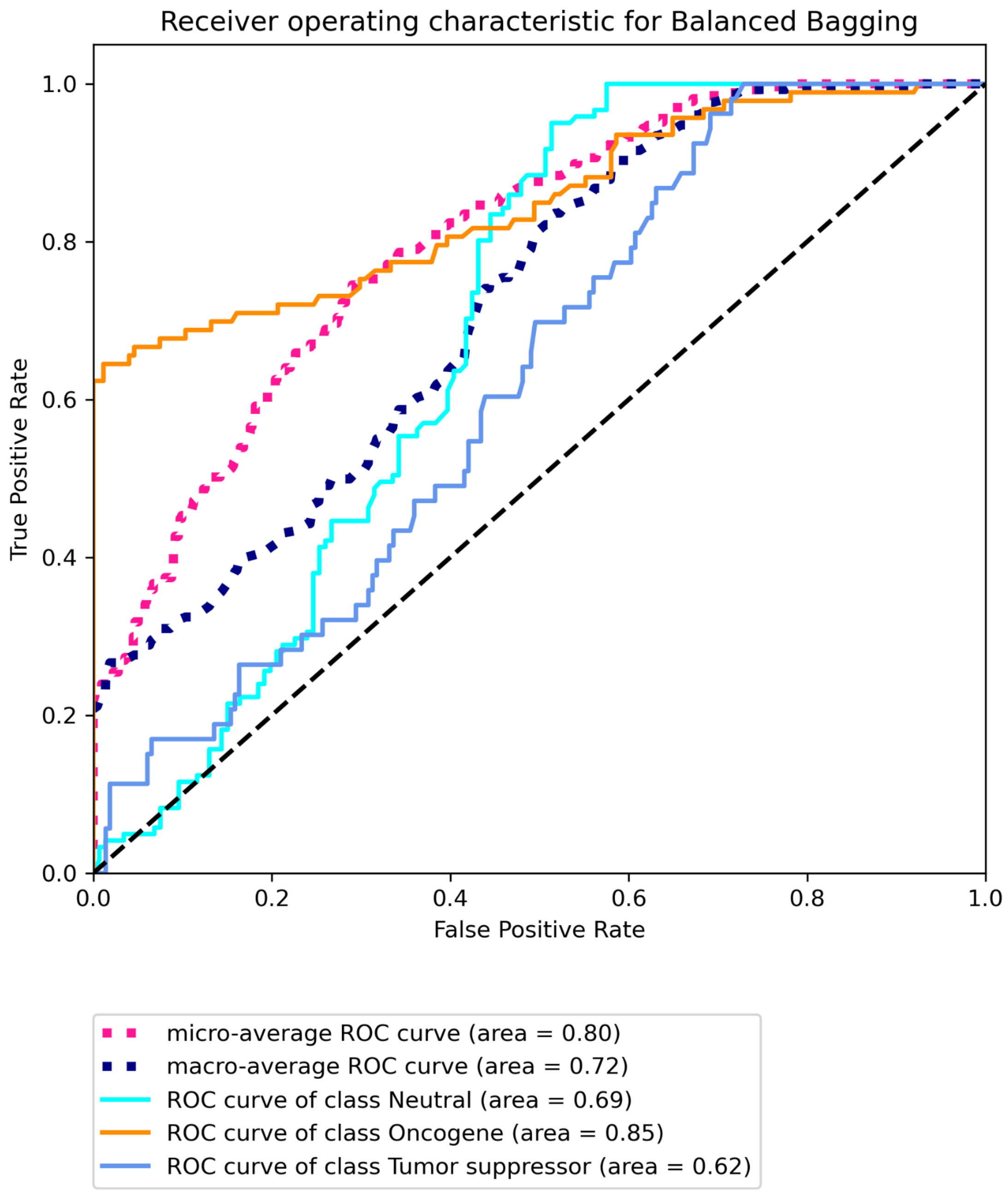

Figure S15C Receiver operating characteristic for LGG using SNV data with CGC labels built on “all” features using Balanced Bagging.

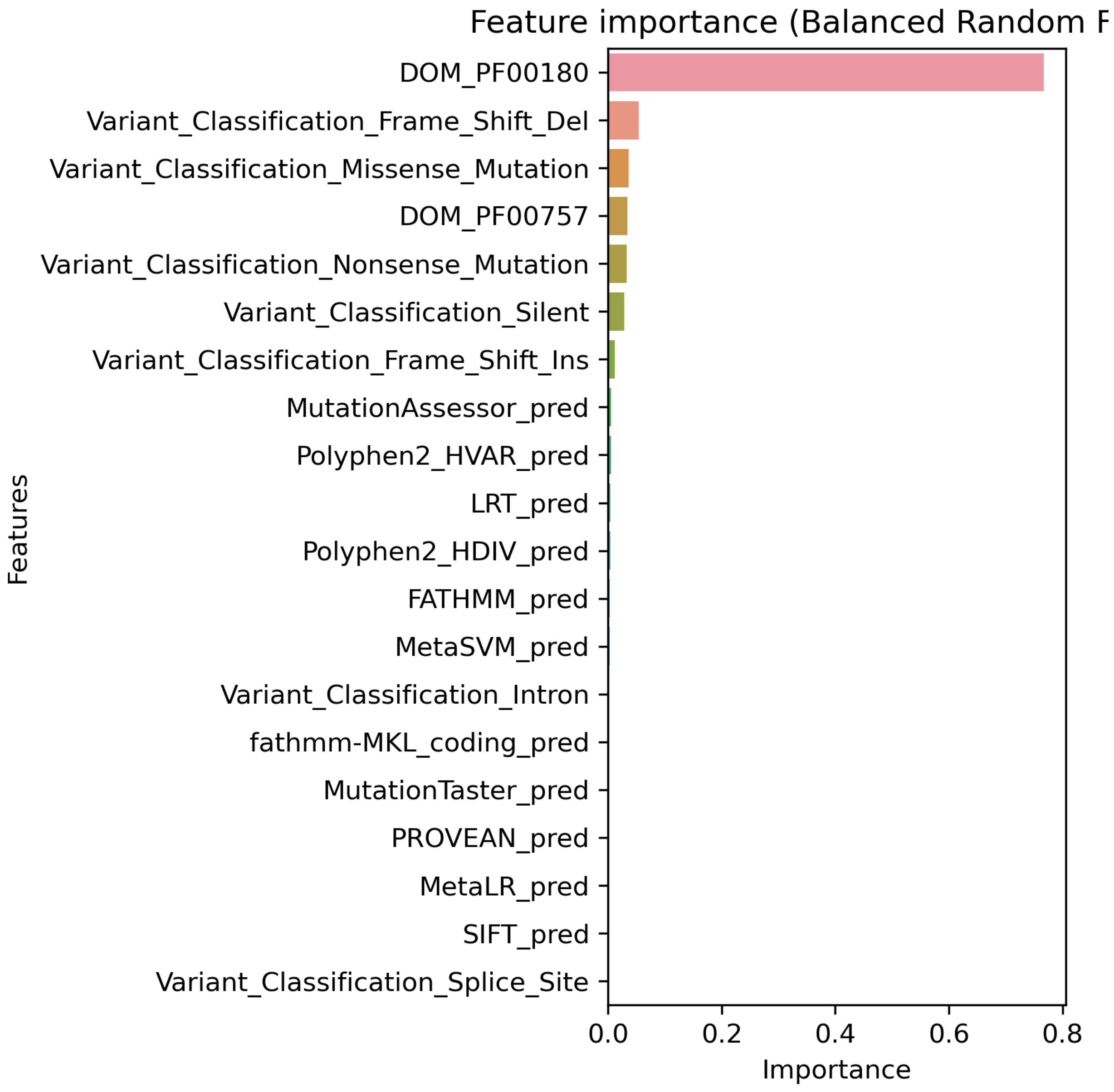

Figure S16A. Top 20 features for LGG using SNV data with CGC labels built on "some" features using Balanced Random Forest.

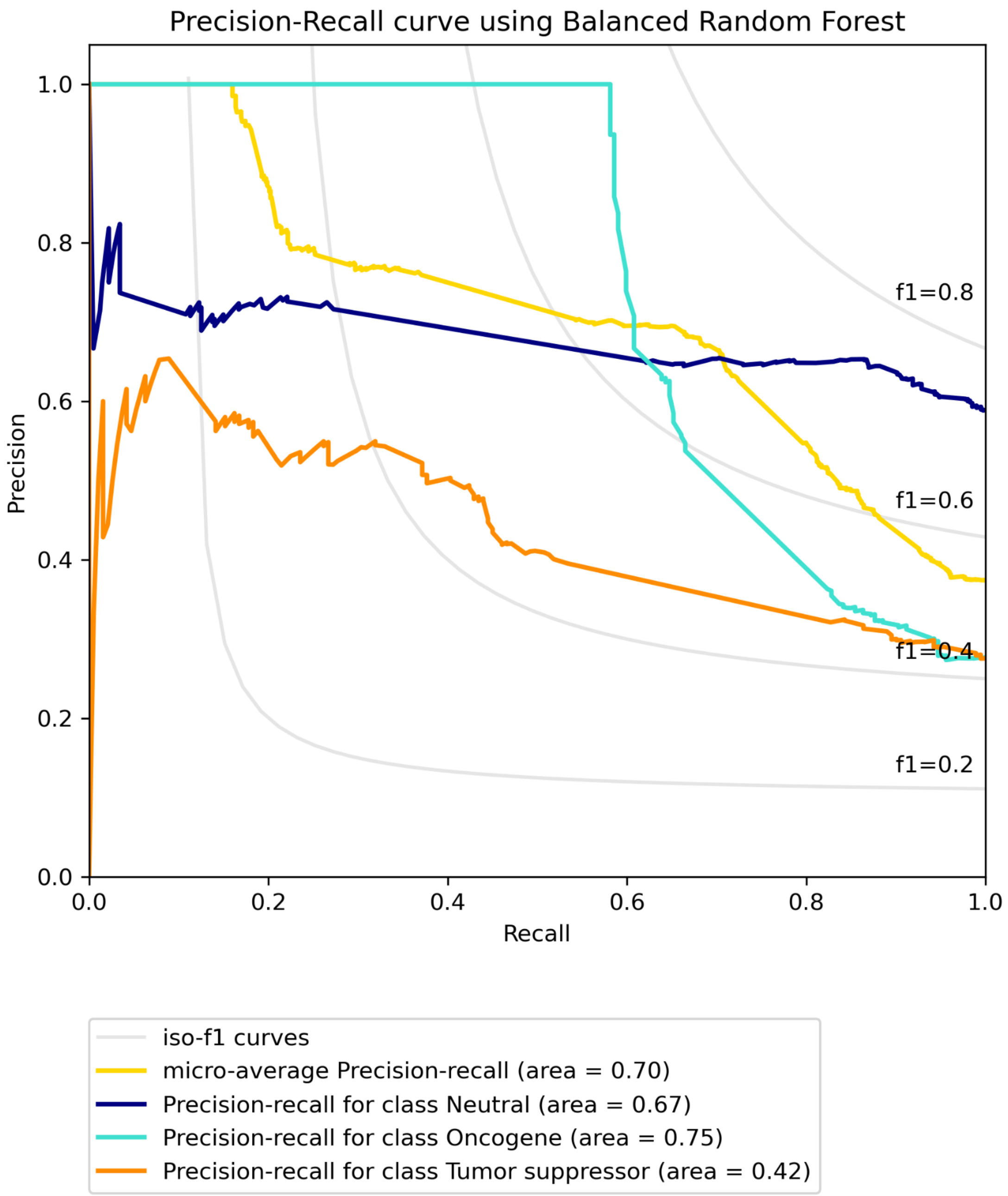

Figure S16B. Precision-recall curve for LGG using SNV data with CGC labels built on “some” features using Balanced Random Forest.

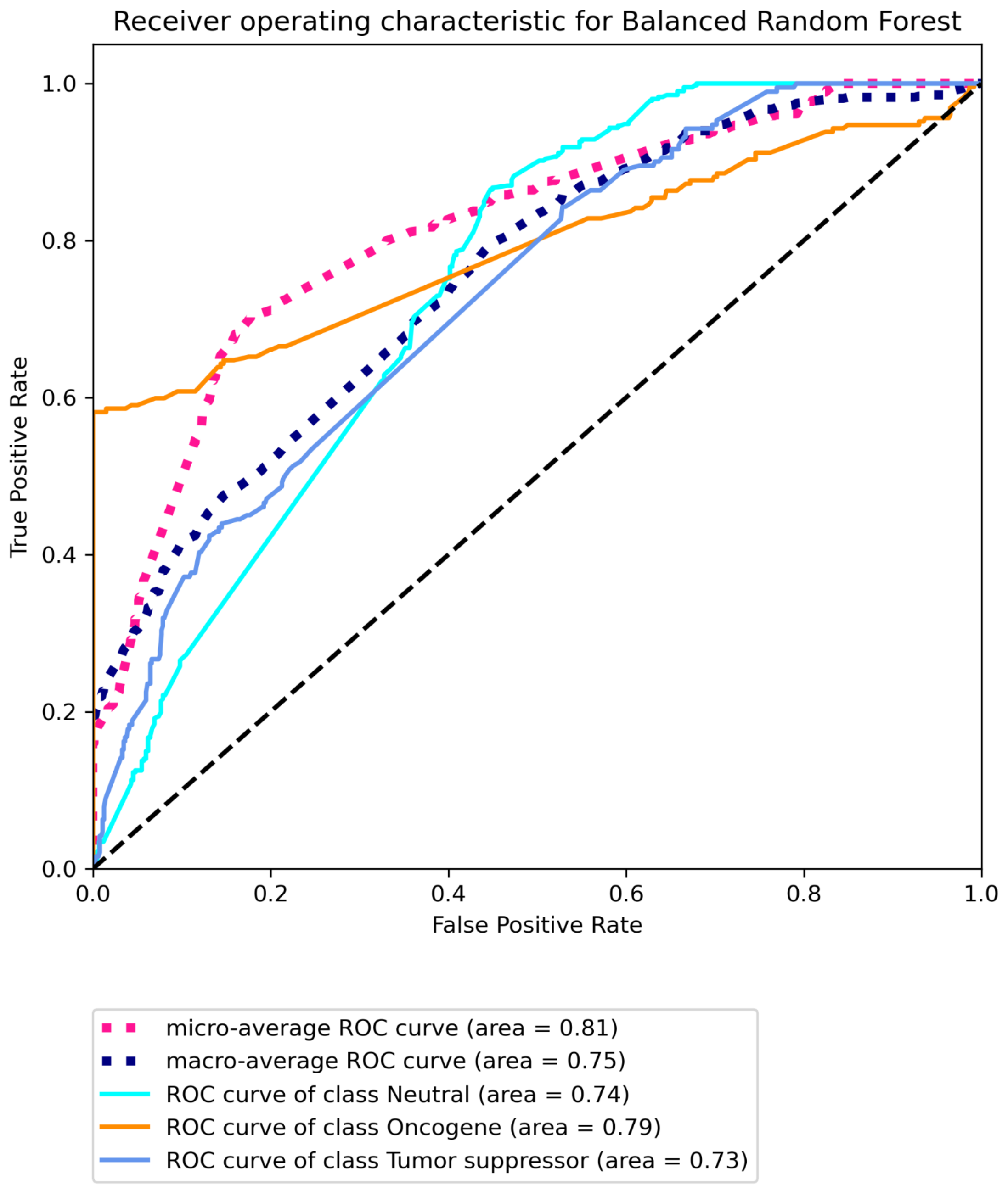

Figure S16C Receiver operating characteristic for LGG using SNV data with CGC labels built on "some" features using Balanced Random Forest.

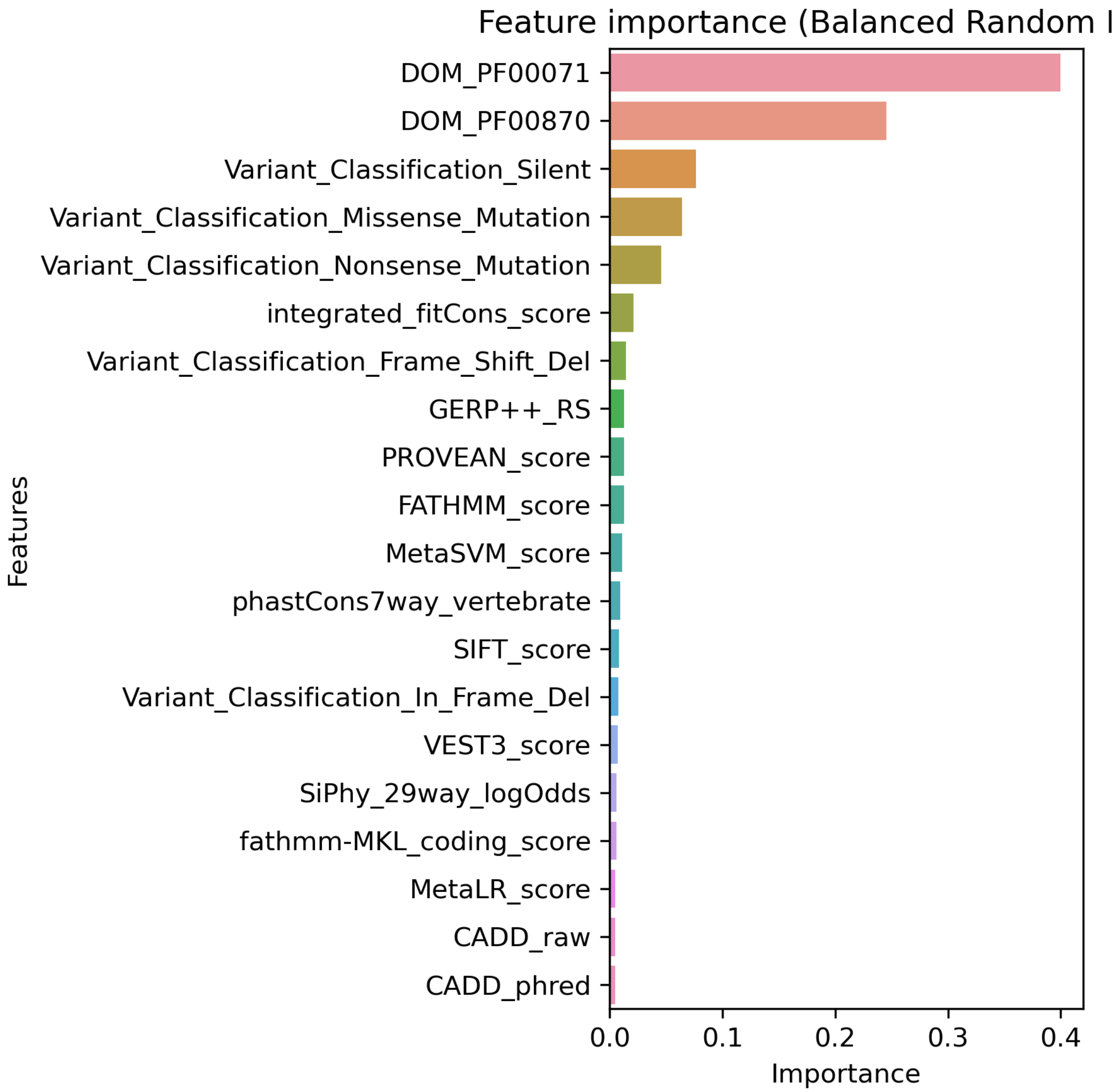

Figure S17A. Top 20 features for LUAD using SNV data with Bailey *et al.* labels built on "all" features using Balanced Random Forest.

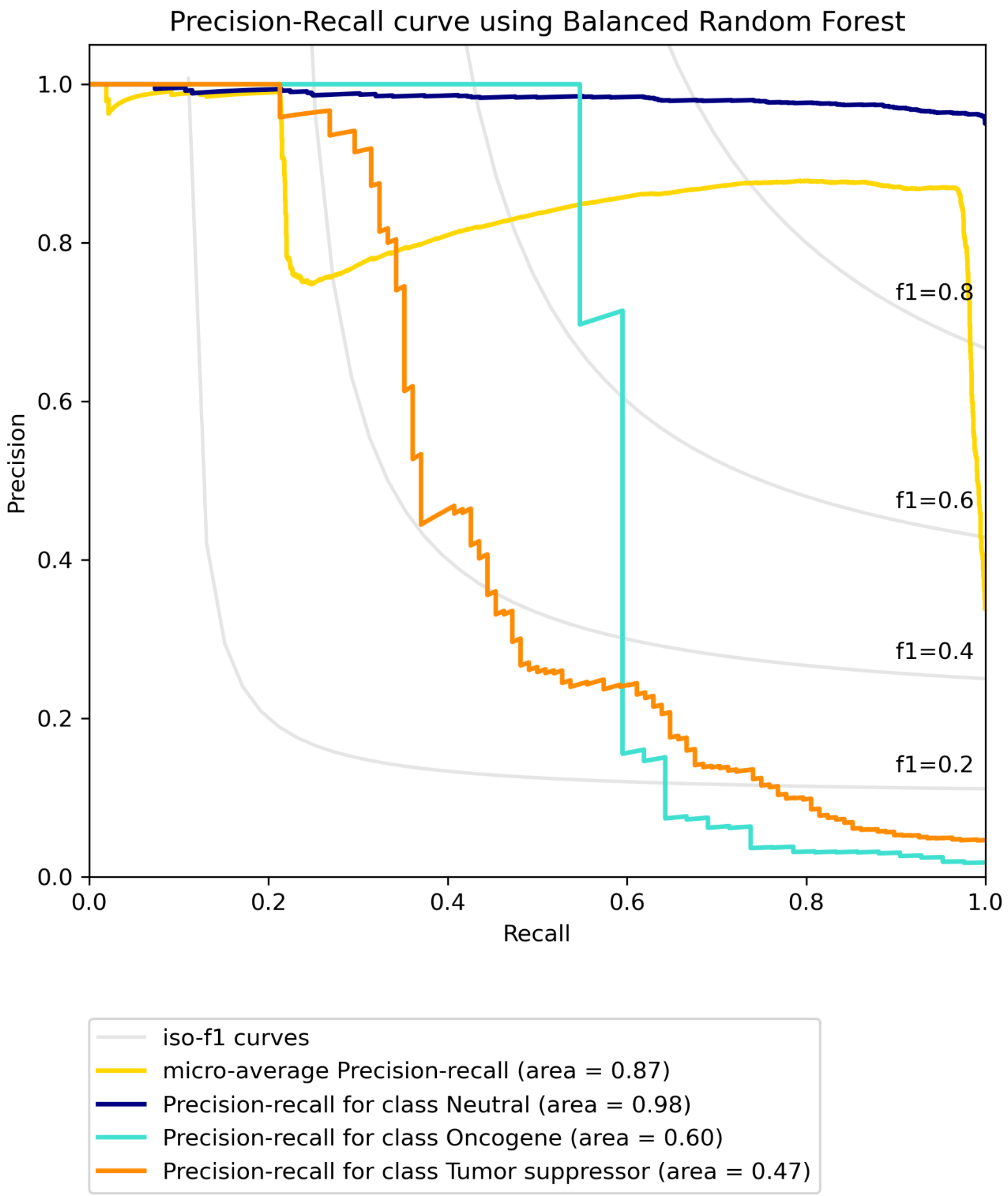

Figure S17B. Precision-recall curve for LUAD using SNV data with Bailey *et al.* labels built on "all" features using Balanced Random Forest.

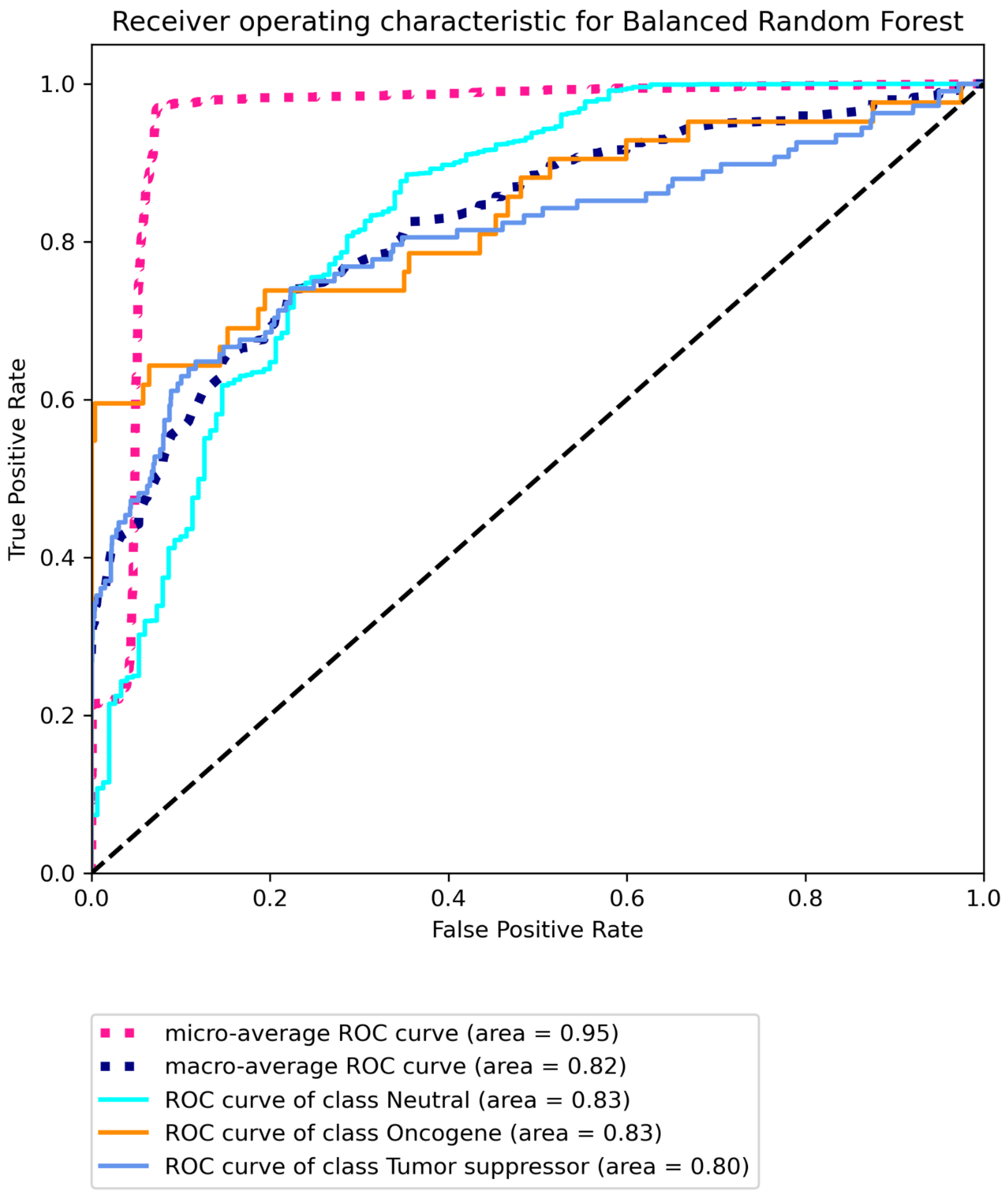

Figure S17C Receiver operating characteristic for LUAD using SNV data with Bailey *et al.* labels built on "all" features using Balanced Random Forest.

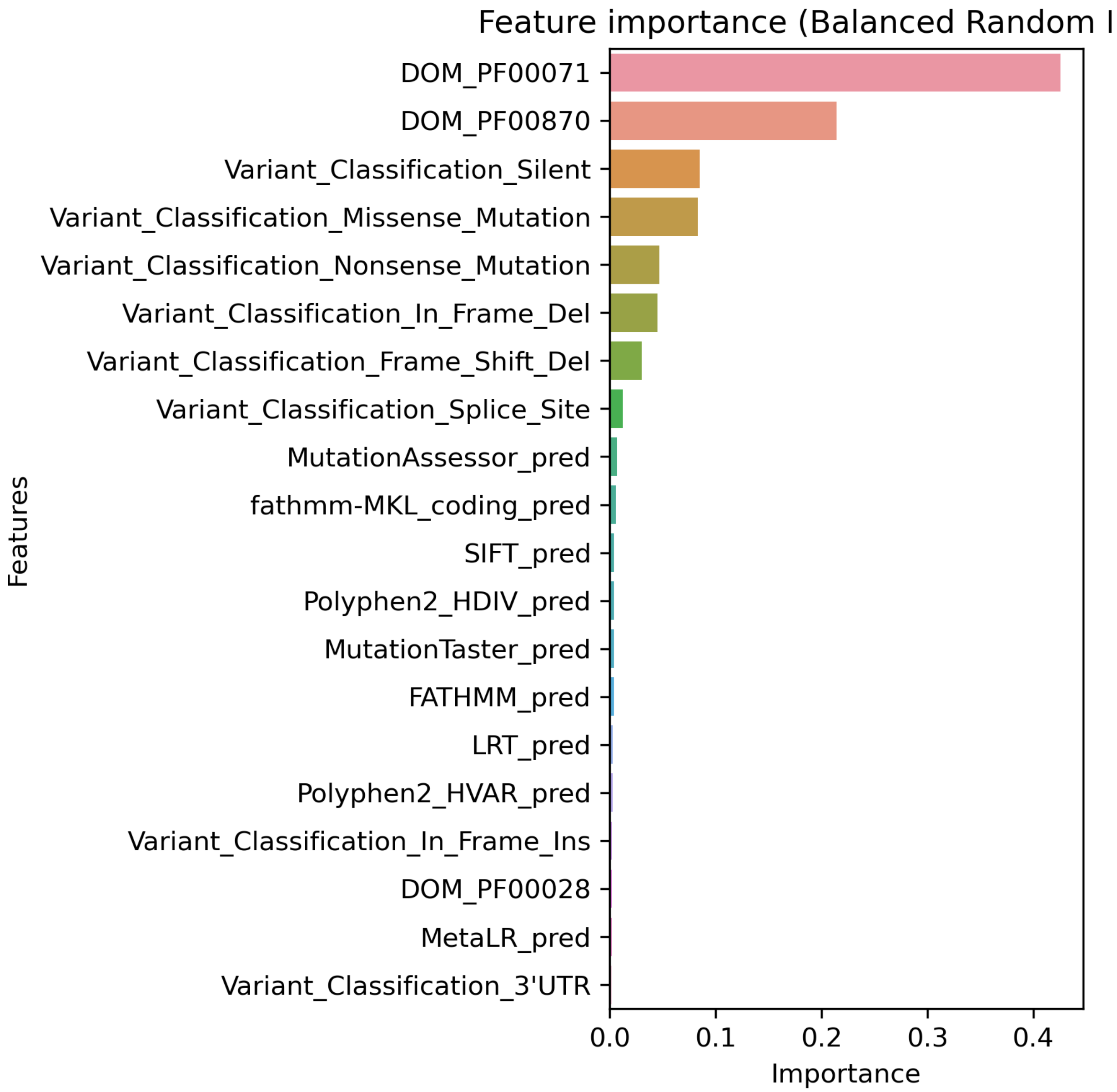

Figure S18A. Top 20 features for LUAD using SNV data with Bailey *et al.* labels built on "some" features using Balanced Random Forest.

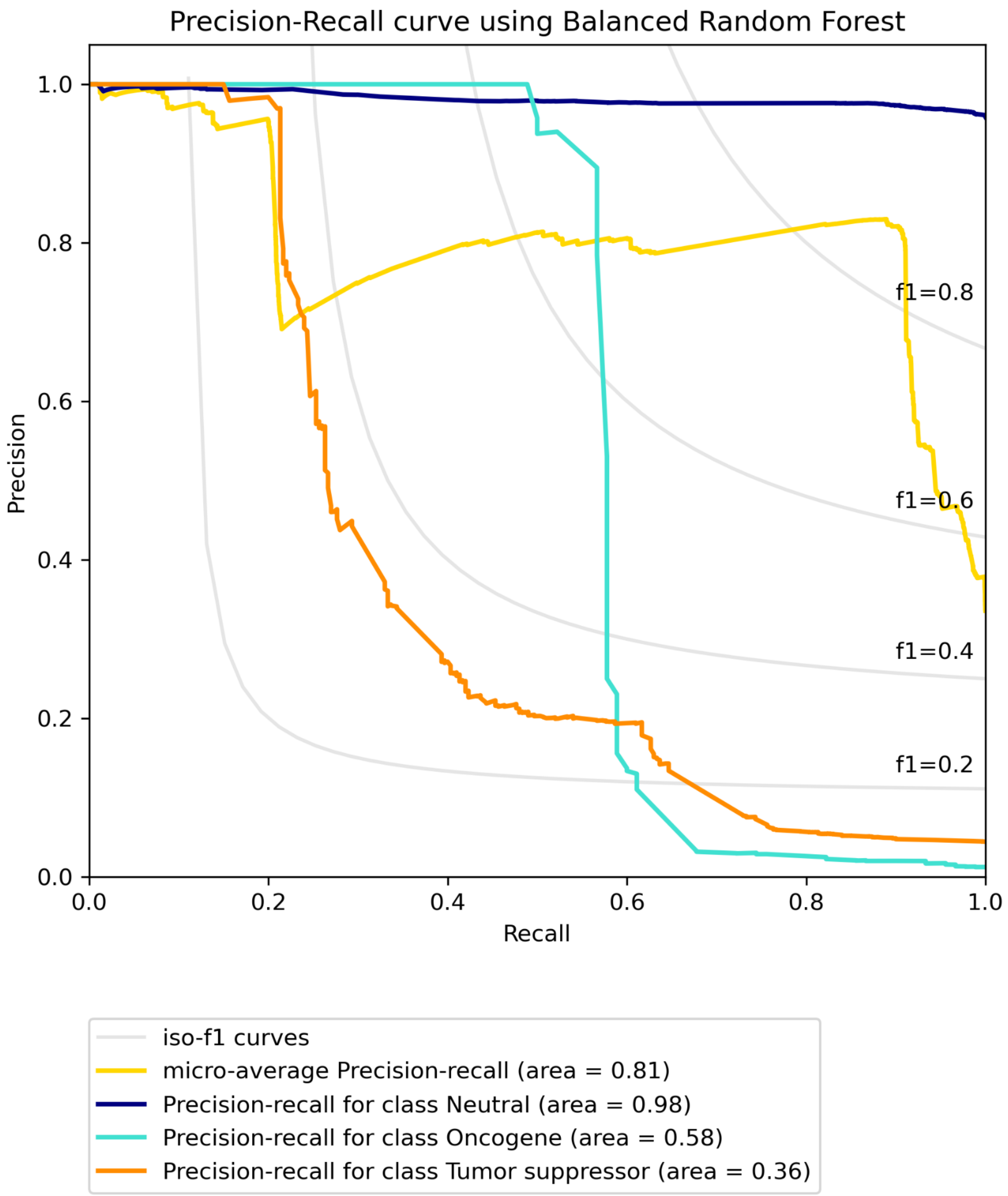

Figure S18B. Precision-recall curve for LUAD using SNV data with Bailey *et al.* labels built on "some" features using Balanced Random Forest.

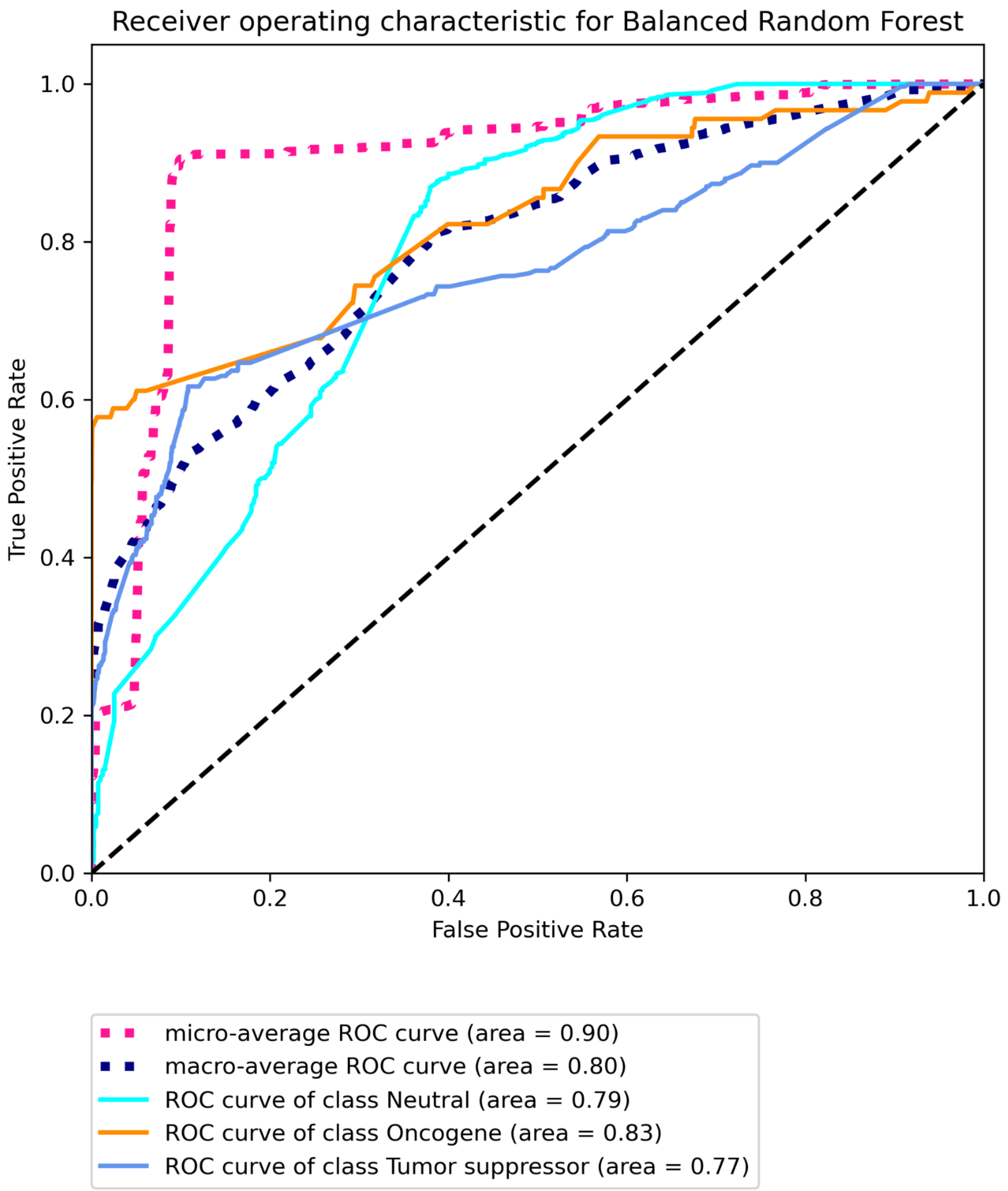

Figure S18C Receiver operating characteristic for LUAD using SNV data with Bailey *et al.* labels built on "some" features using Balanced Random Forest.

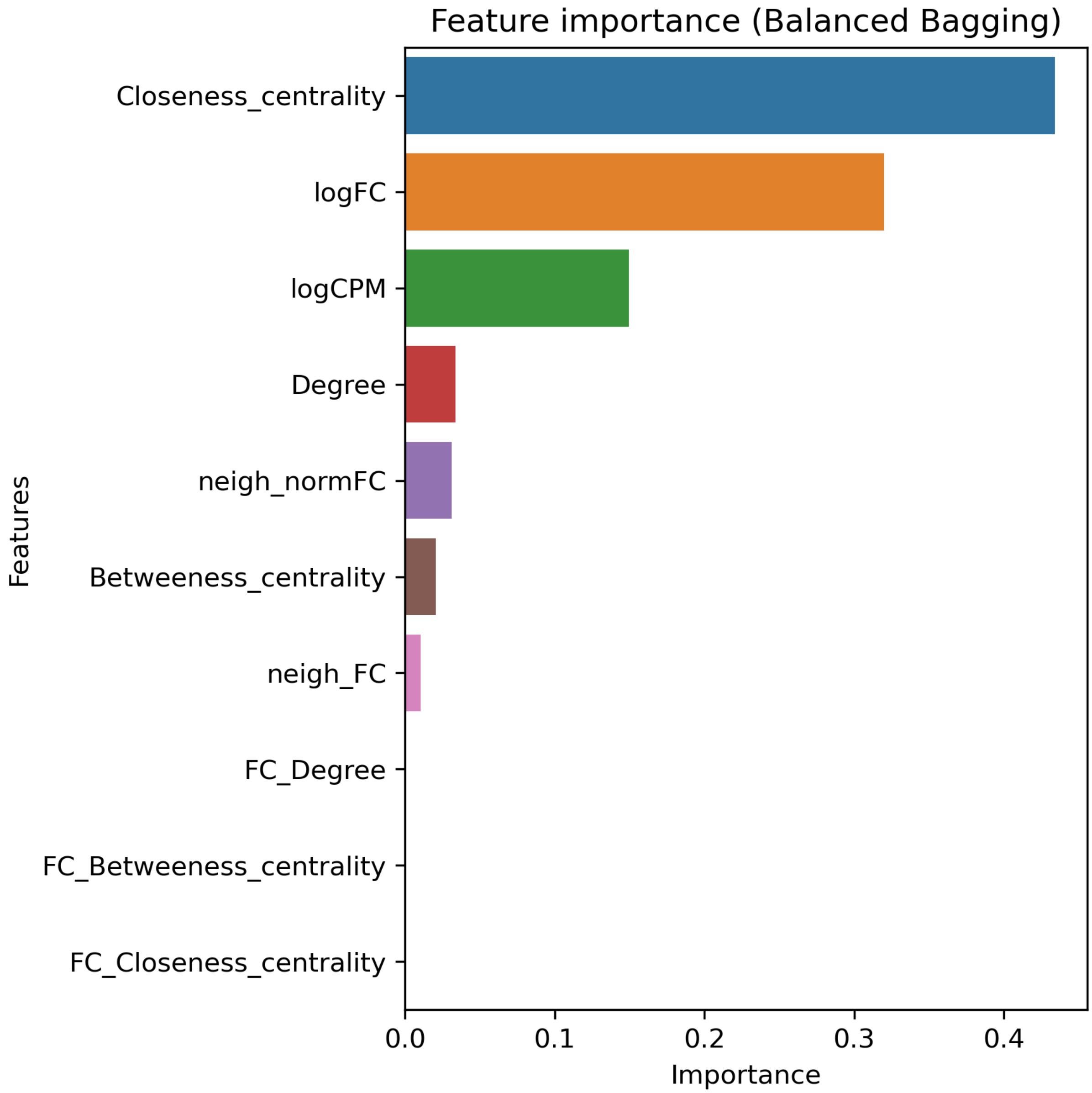

Figure S19A. Top 20 features for BRCA using RNA data with Bailey *et al.* labels built using Balanced bagging.

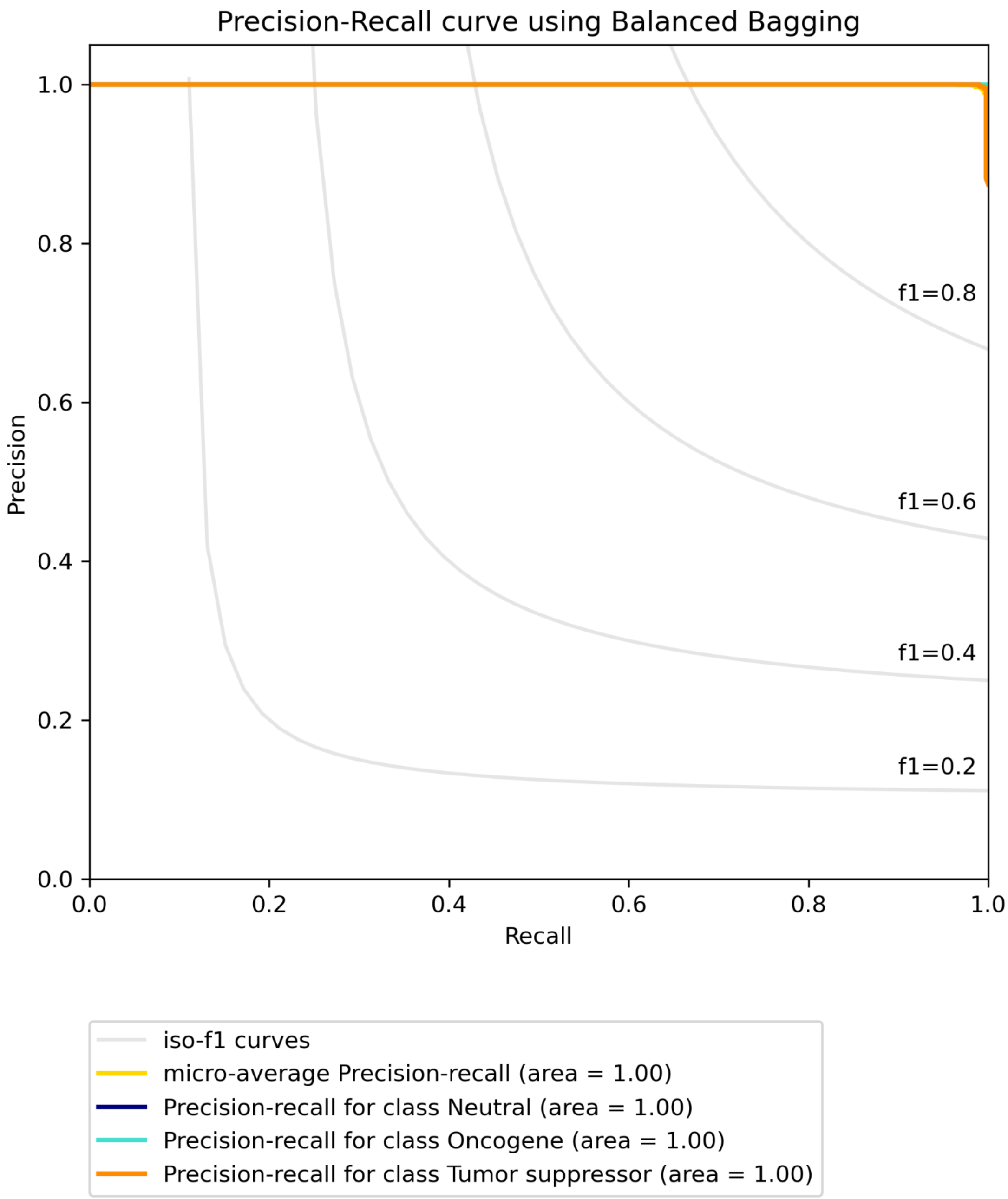

Figure S19B. Precision-recall curve for BRCA using RNA data with Bailey *et al.* labels built using Balanced bagging.

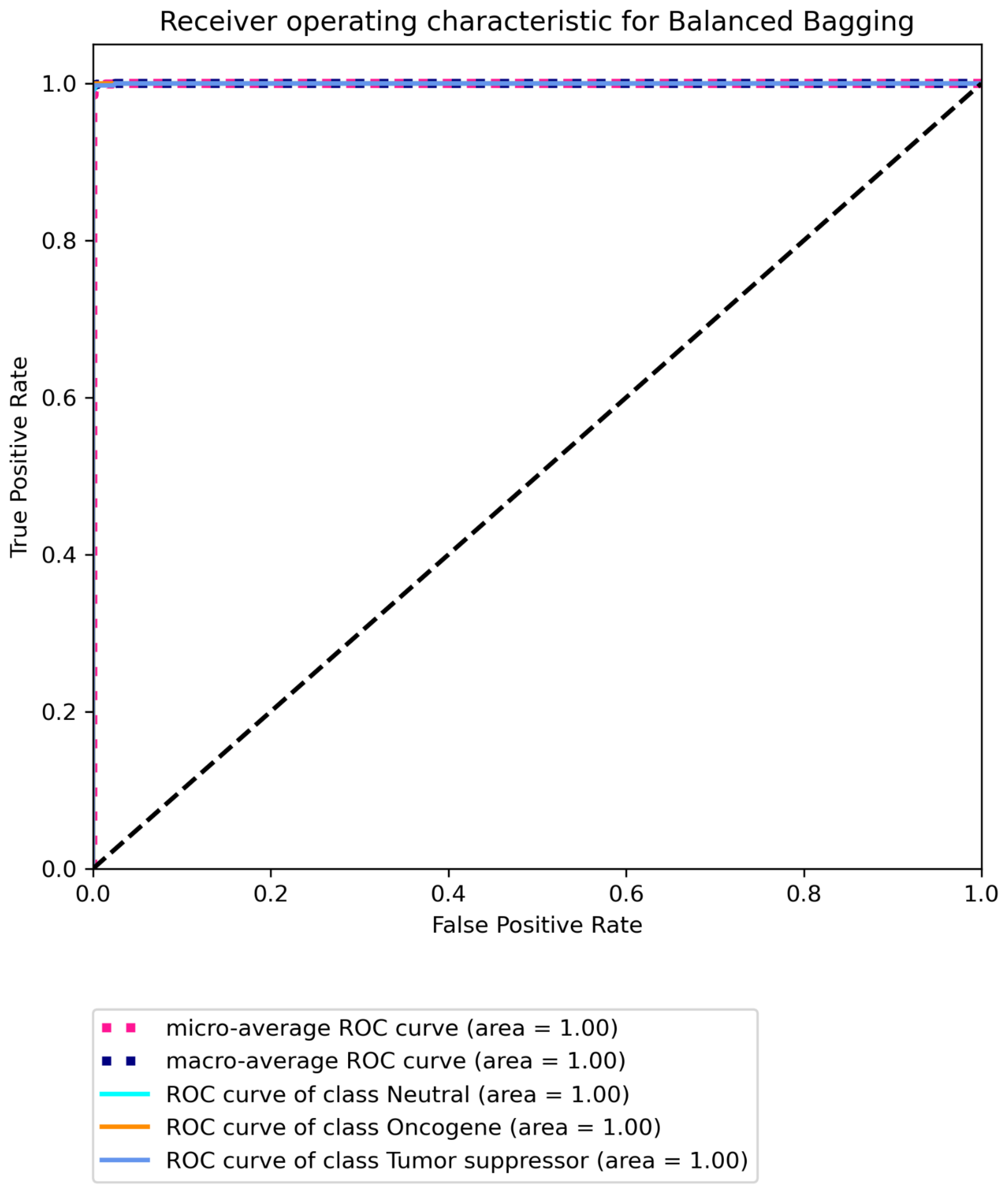

Figure S19C Receiver operating characteristic for BRCA using RNA data with Bailey *et al.* labels built using Balanced bagging.

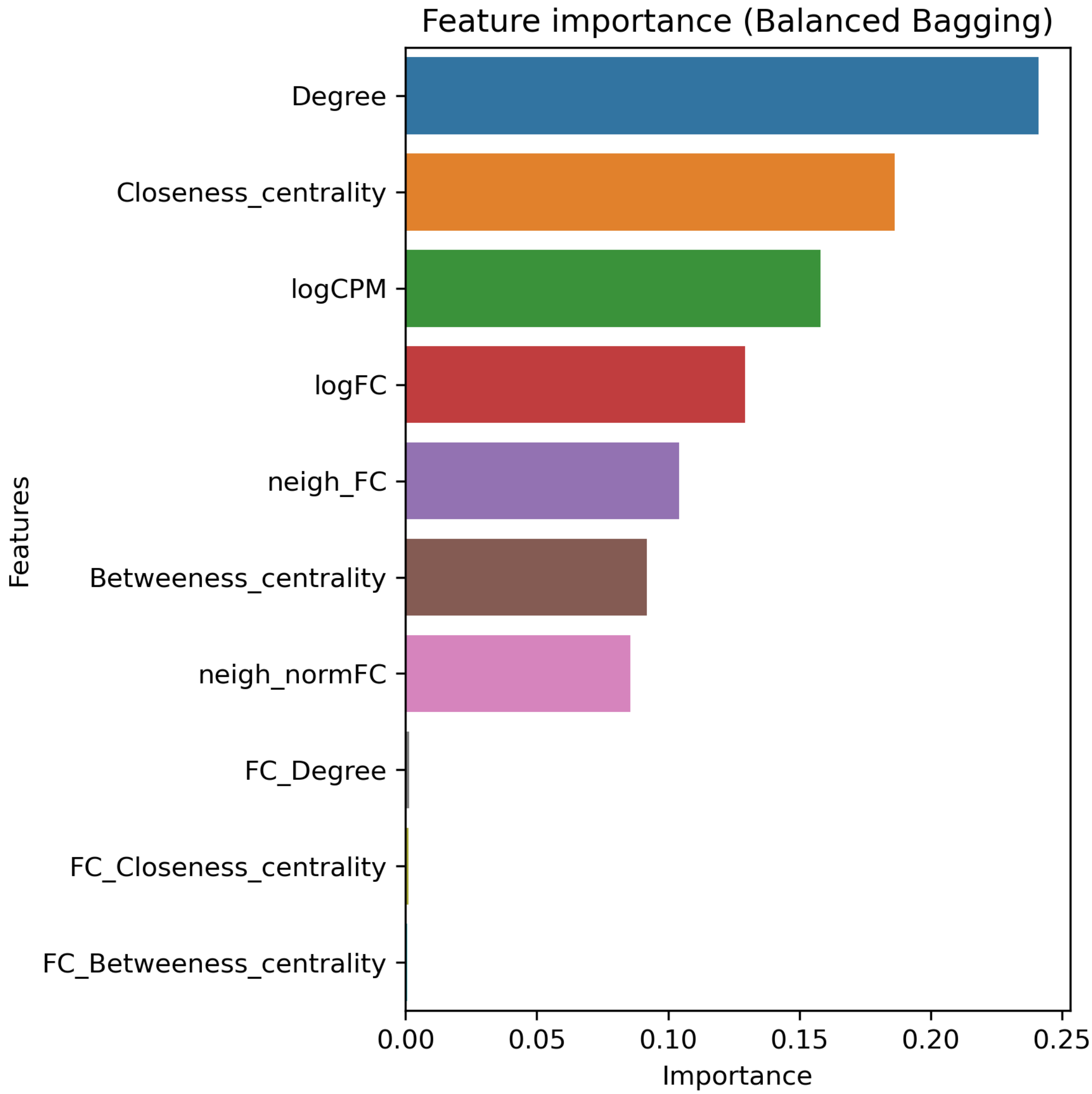

Figure S20A. Top 20 features for BRCA using RNA data with CGC labels built using Balanced bagging.

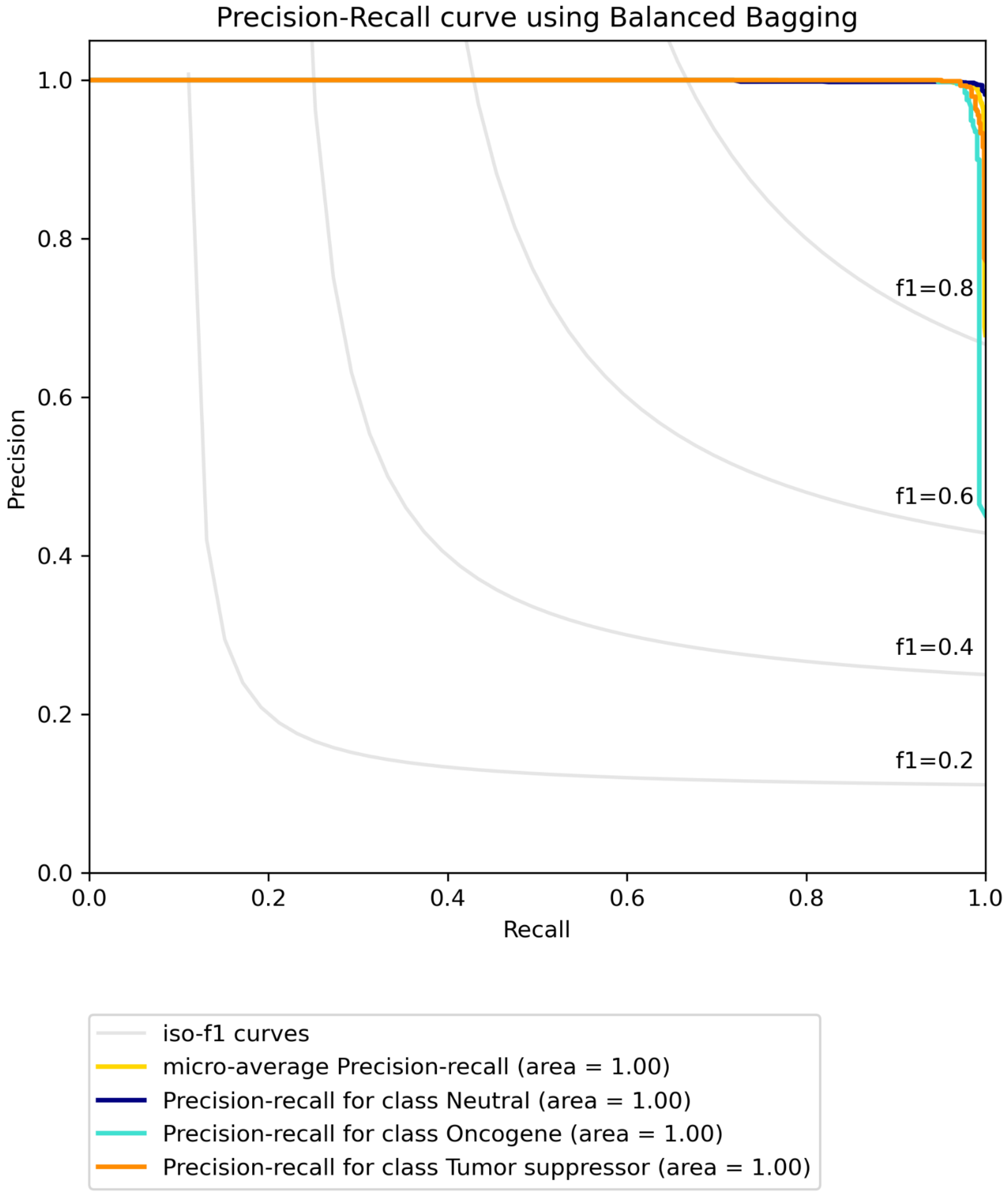

Figure S20B. Precision-recall curve for BRCA using RNA data with CGC labels built using Balanced bagging.

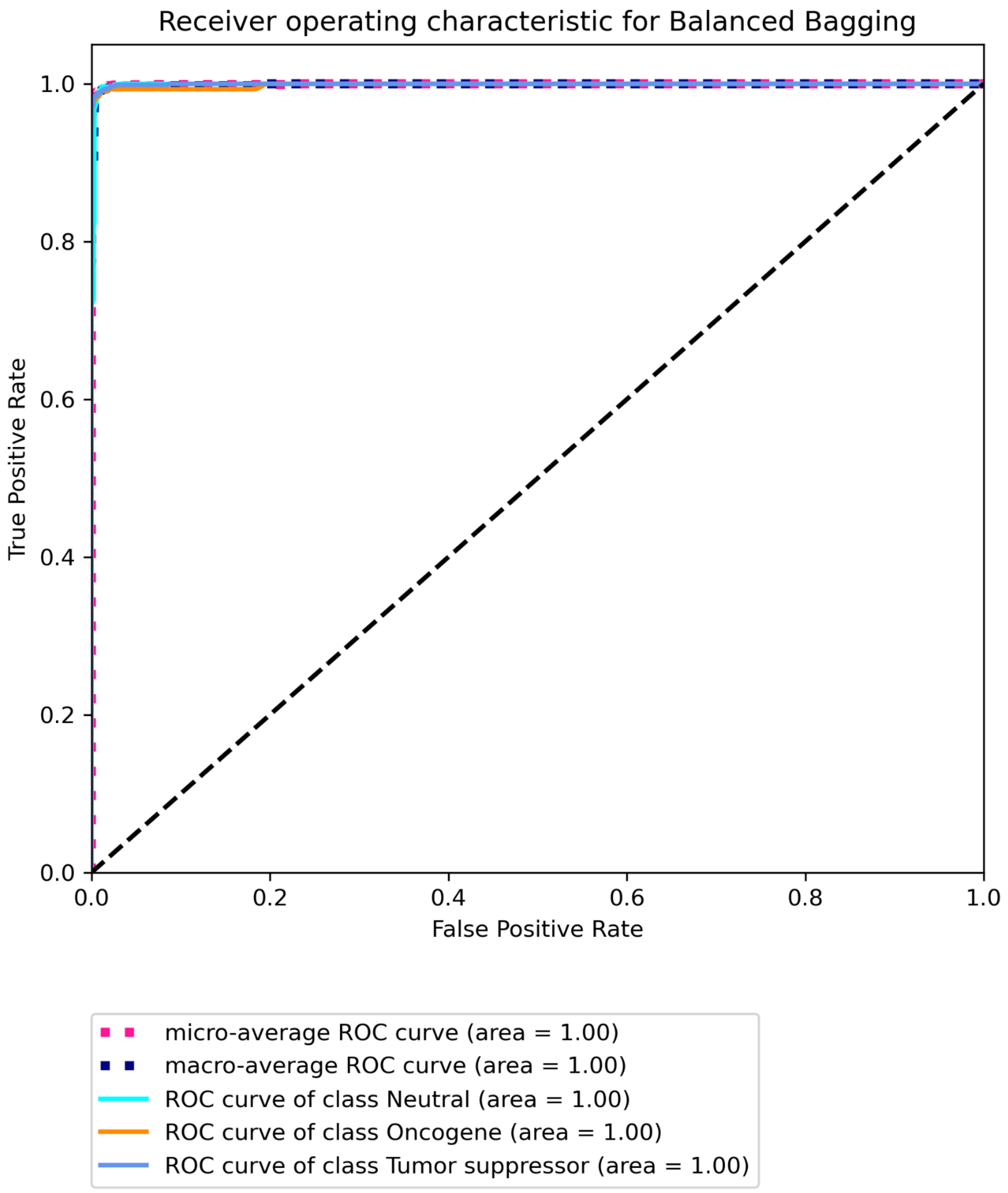

Figure S20C Receiver operating characteristic for BRCA using RNA data with CGC labels built using Balanced bagging.

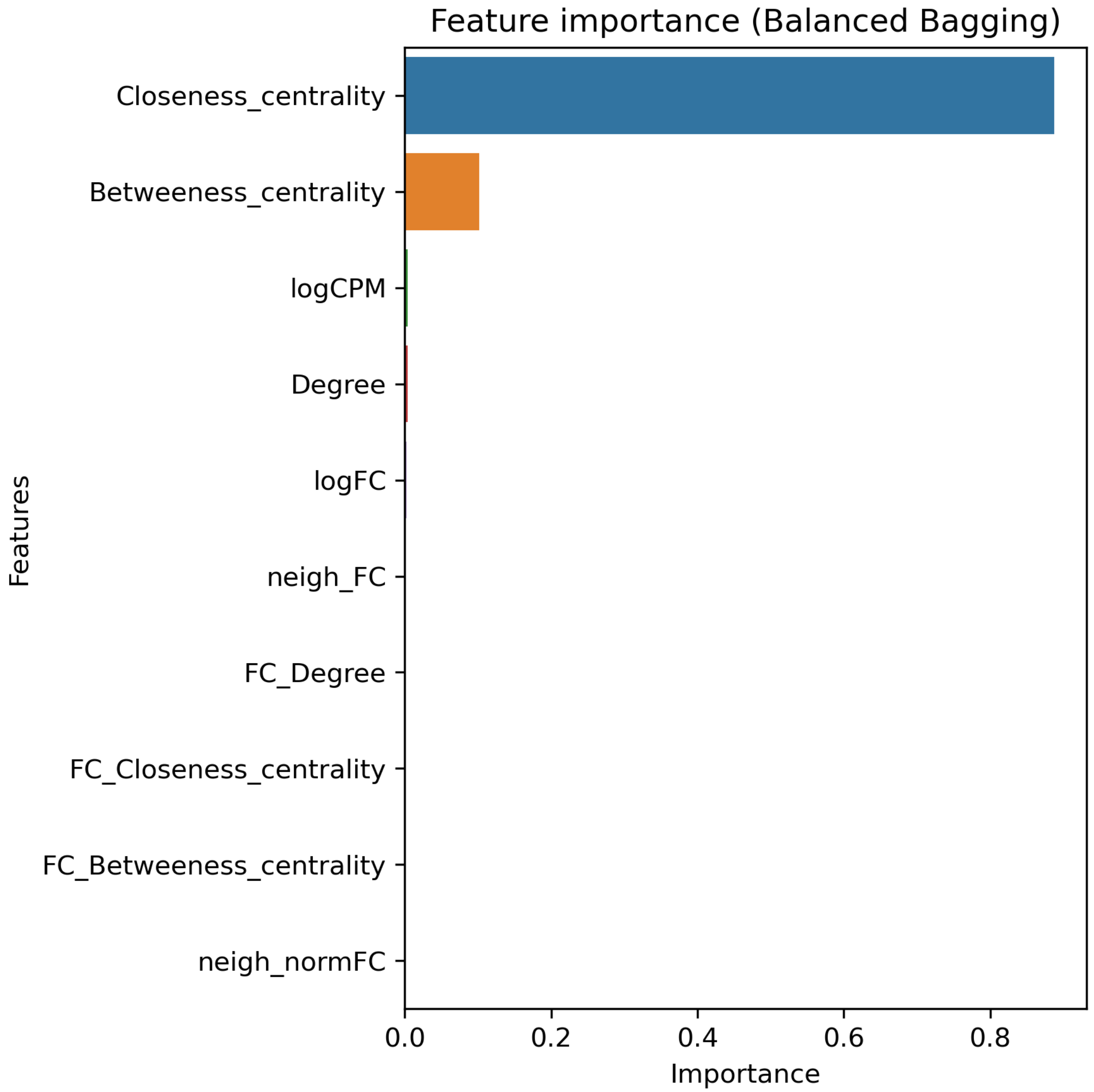

Figure S21A. Top 20 features for BRCA using RNA data with CIViC labels built using Balanced bagging.

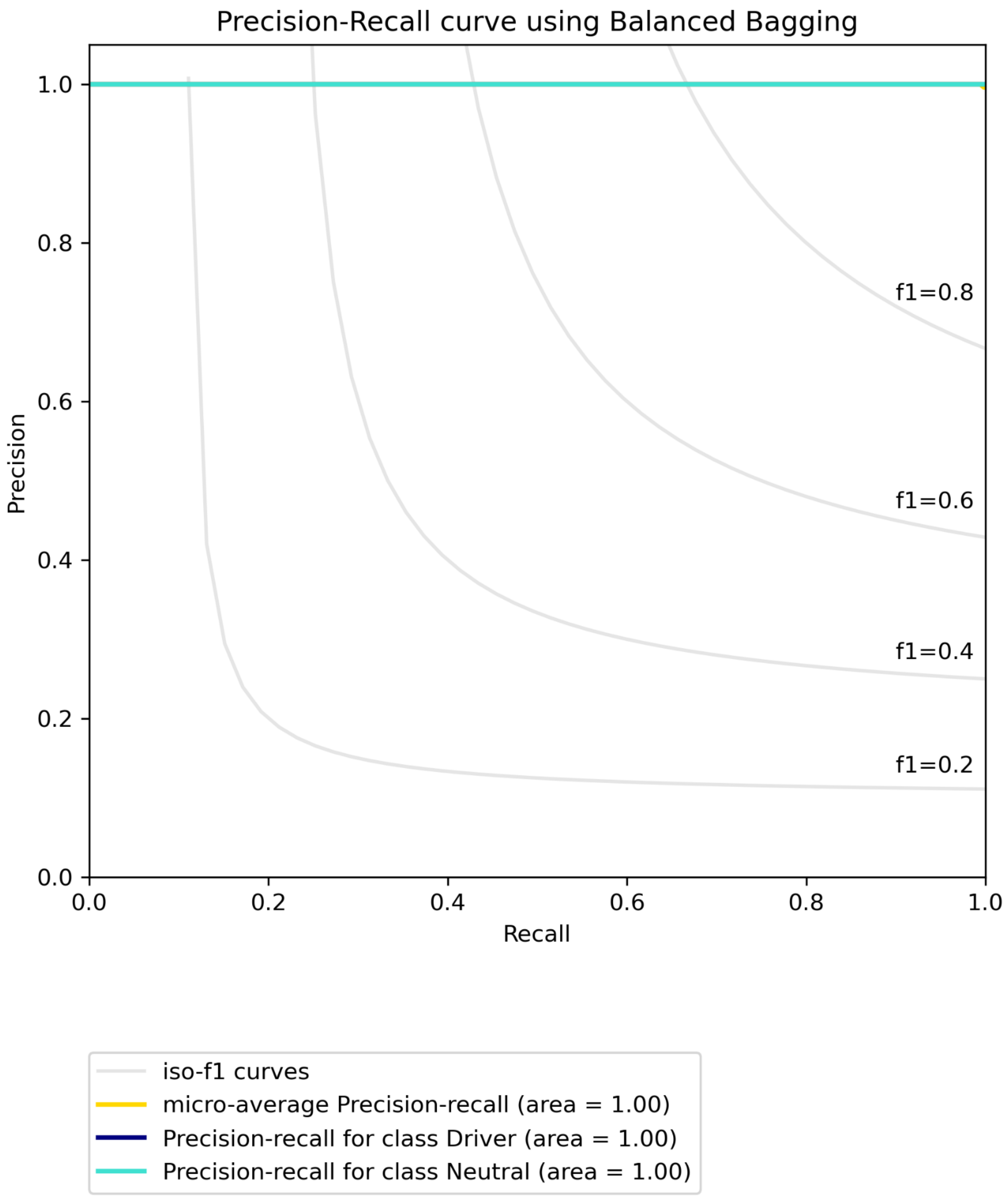

Figure S21B. Precision-recall curve for BRCA using RNA data with CIViC labels built using Balanced bagging.

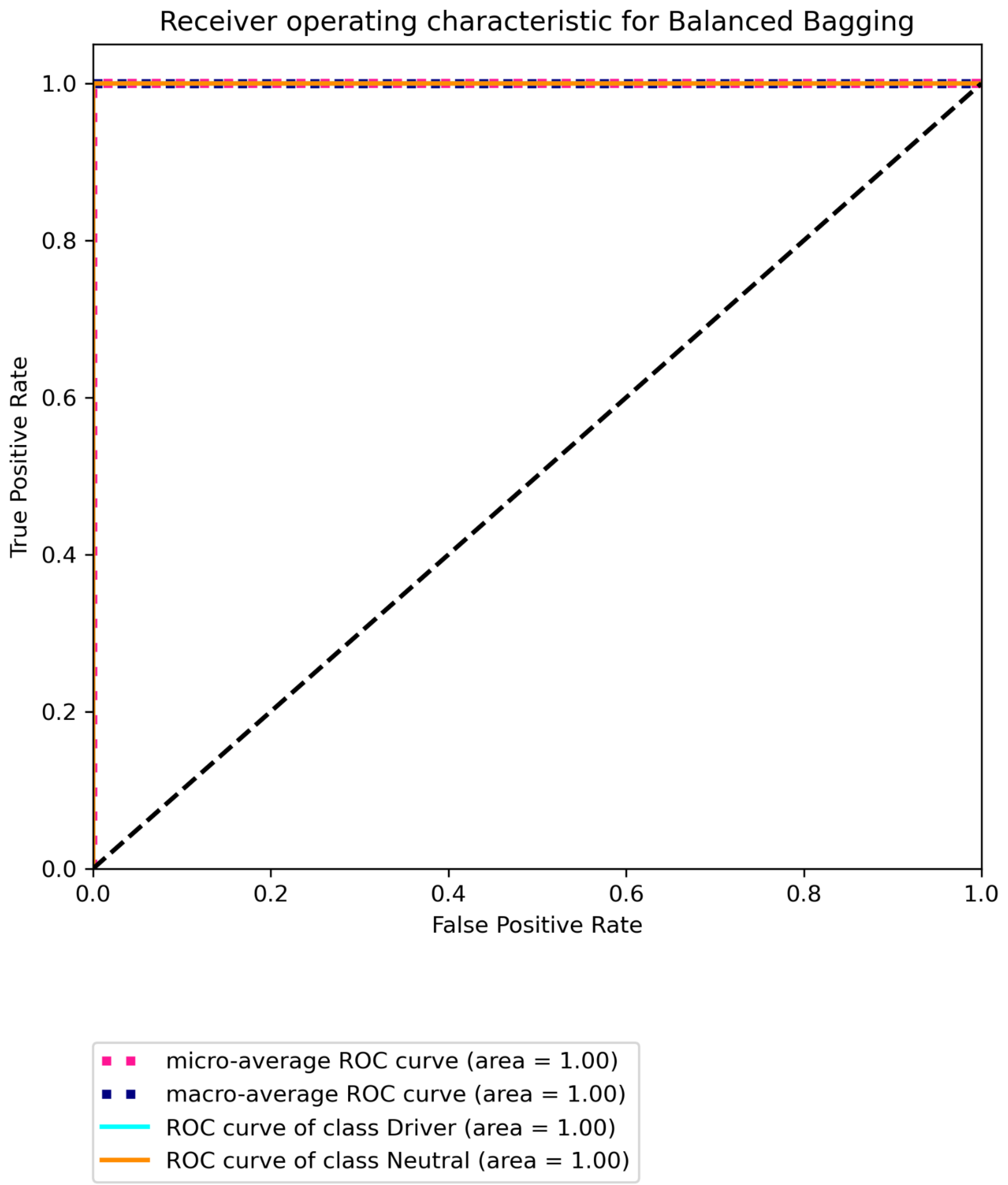

Figure S21C Receiver operating characteristic for BRCA using RNA data with CIViC labels built using Balanced bagging.

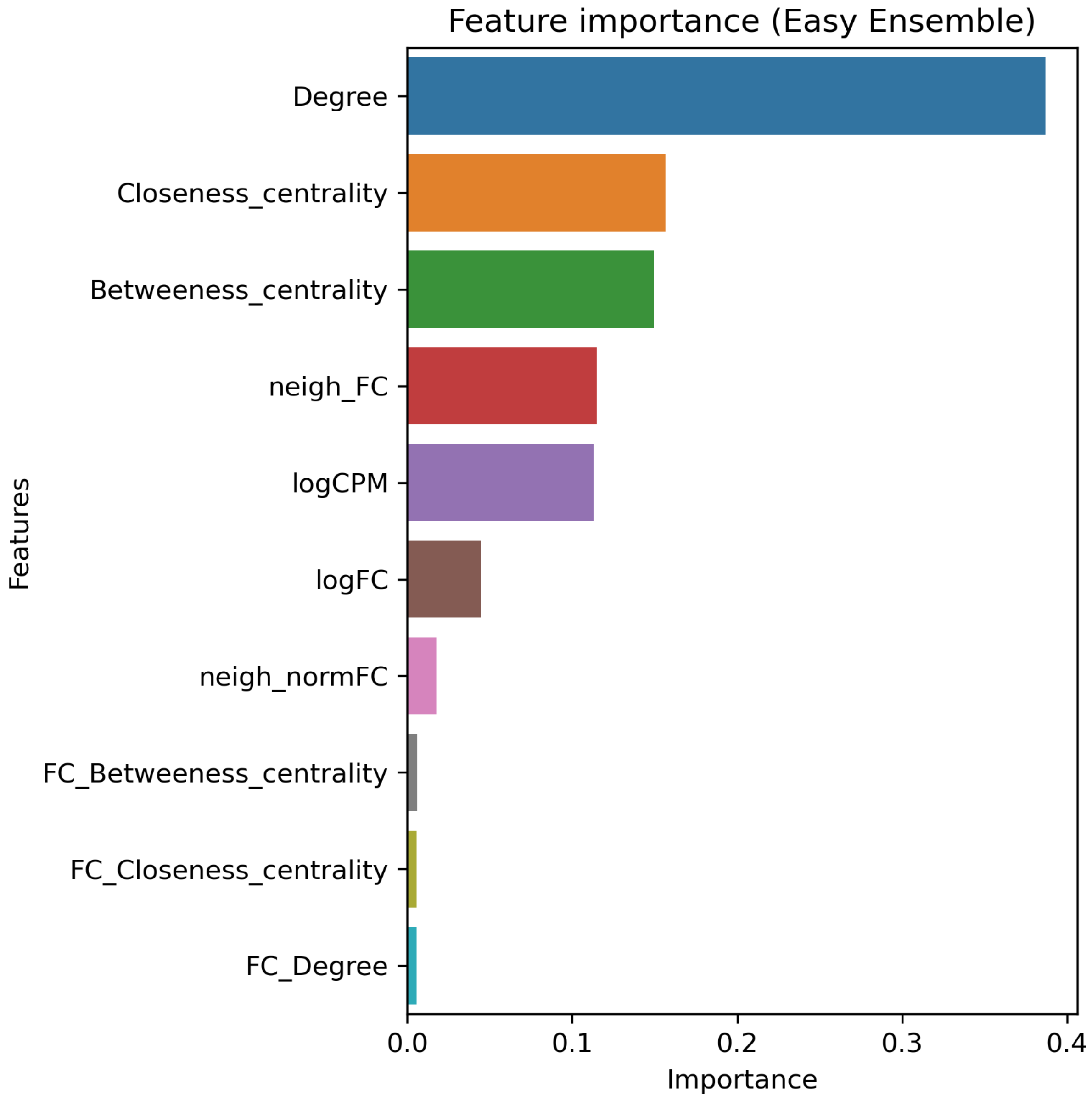

Figure S22A. Top 20 features for BRCA using RNA data with Marellotto *et al.* labels built using Easy Ensemble.

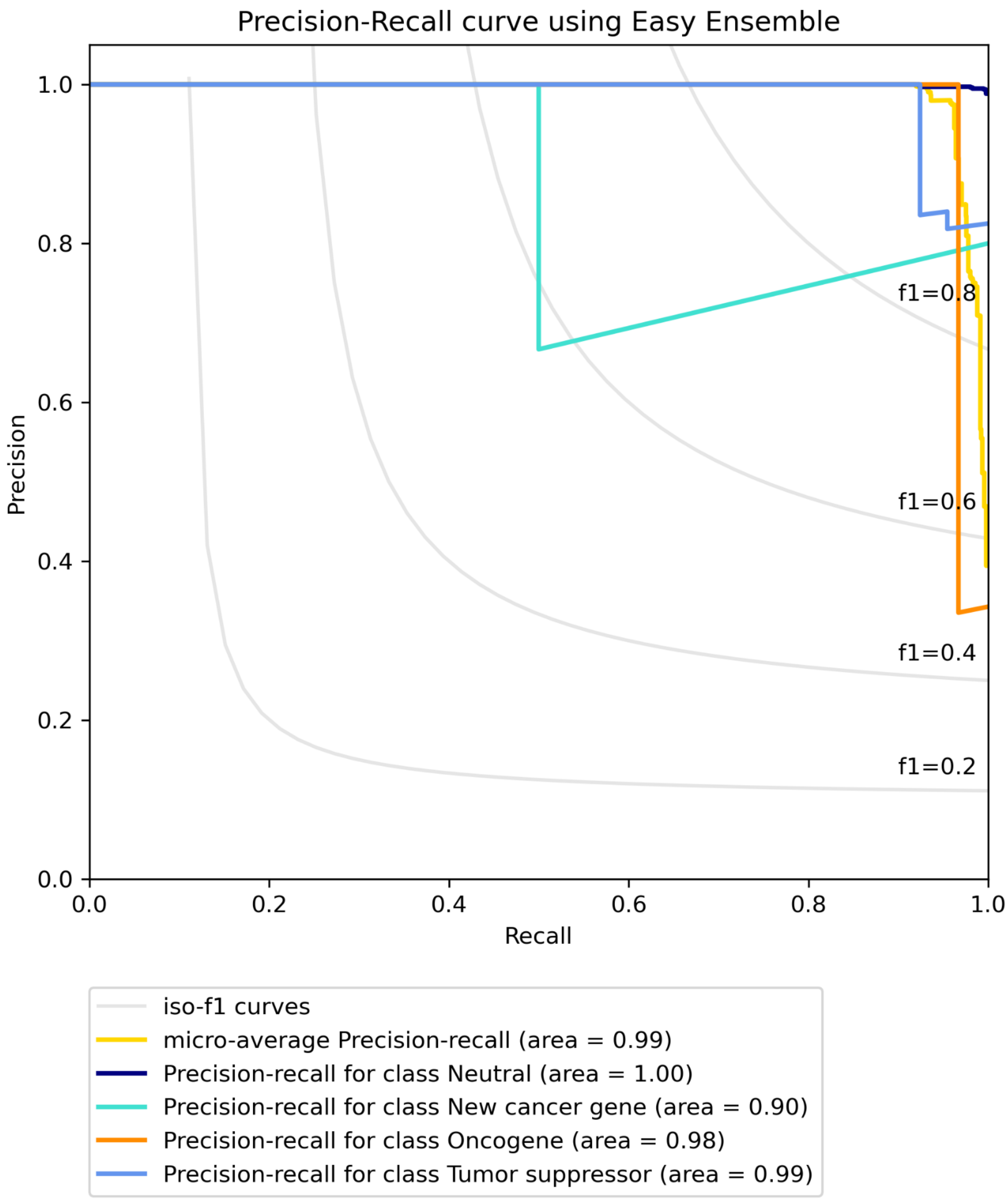

Figure S22B. Precision-recall curve for BRCA using RNA data with Marellotto *et al.* labels built using Easy Ensemble.

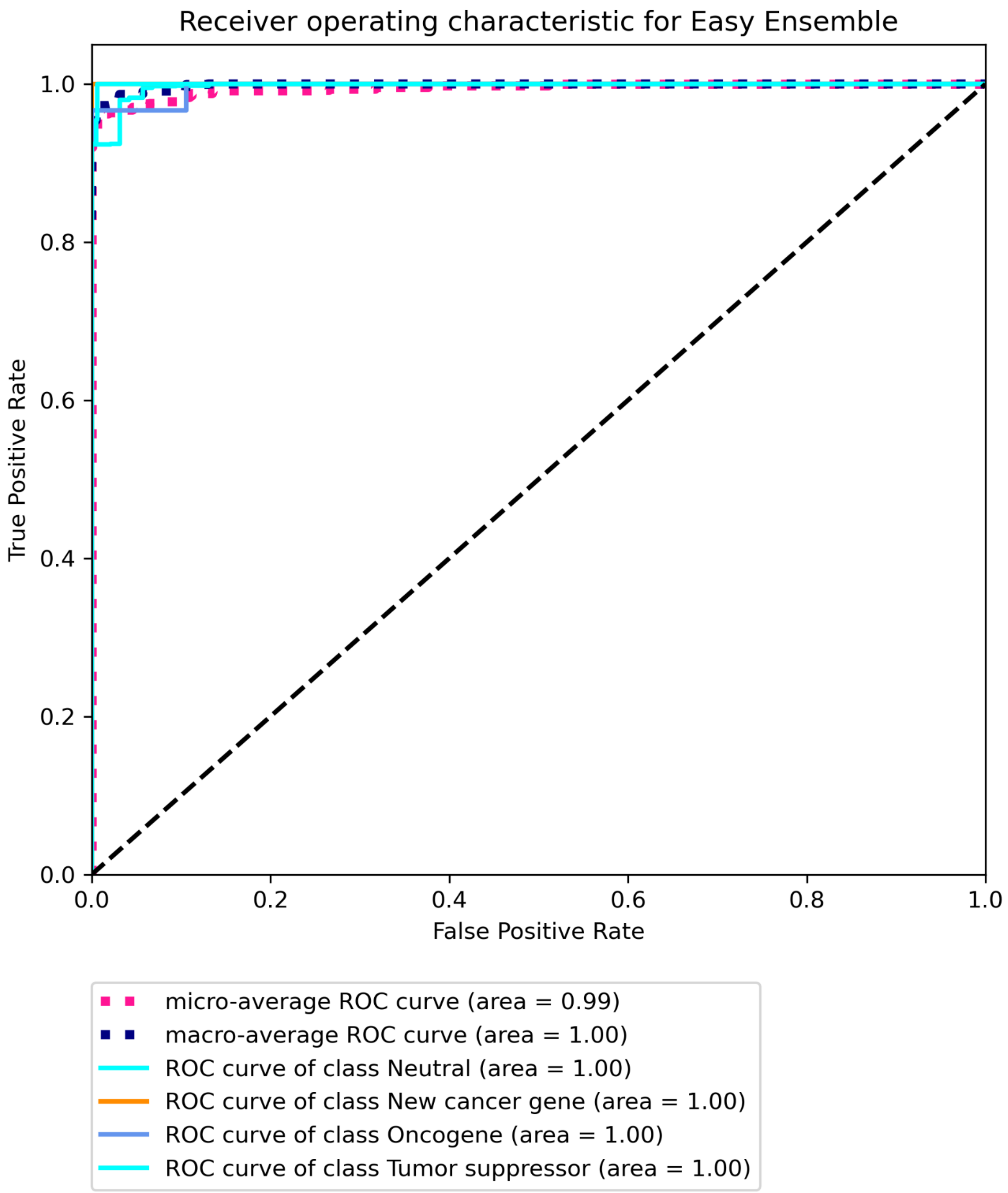

Figure S22C Receiver operating characteristic for BRCA using RNA data with Marellotto *et al.* labels built using Easy Ensemble.

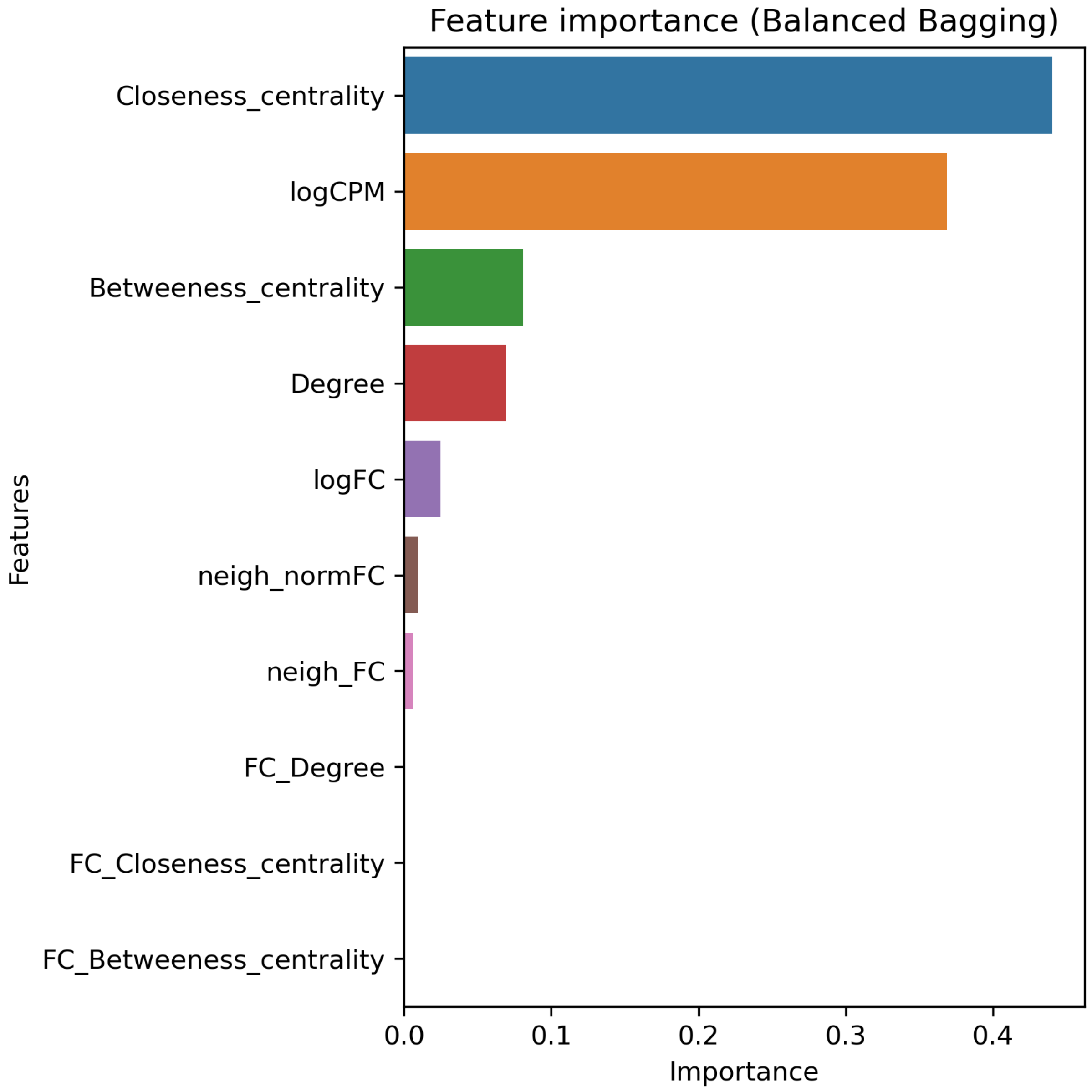

Figure S23A. Top 20 features for COAD using RNA data with Bailey *et al.* labels built using Balanced bagging.

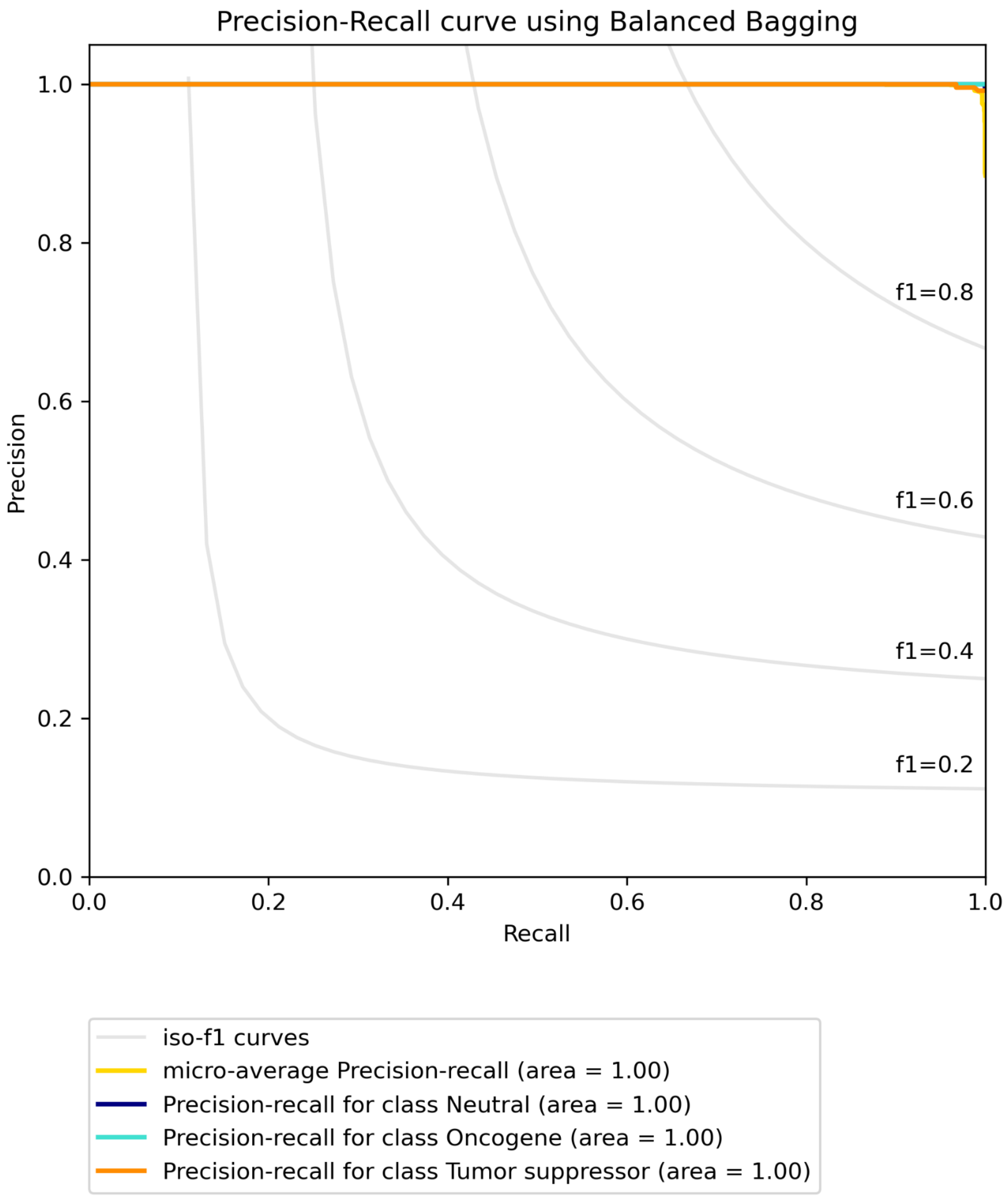

Figure S23B. Precision-recall curve for COAD using RNA data with Bailey *et al.* labels built using Balanced bagging.

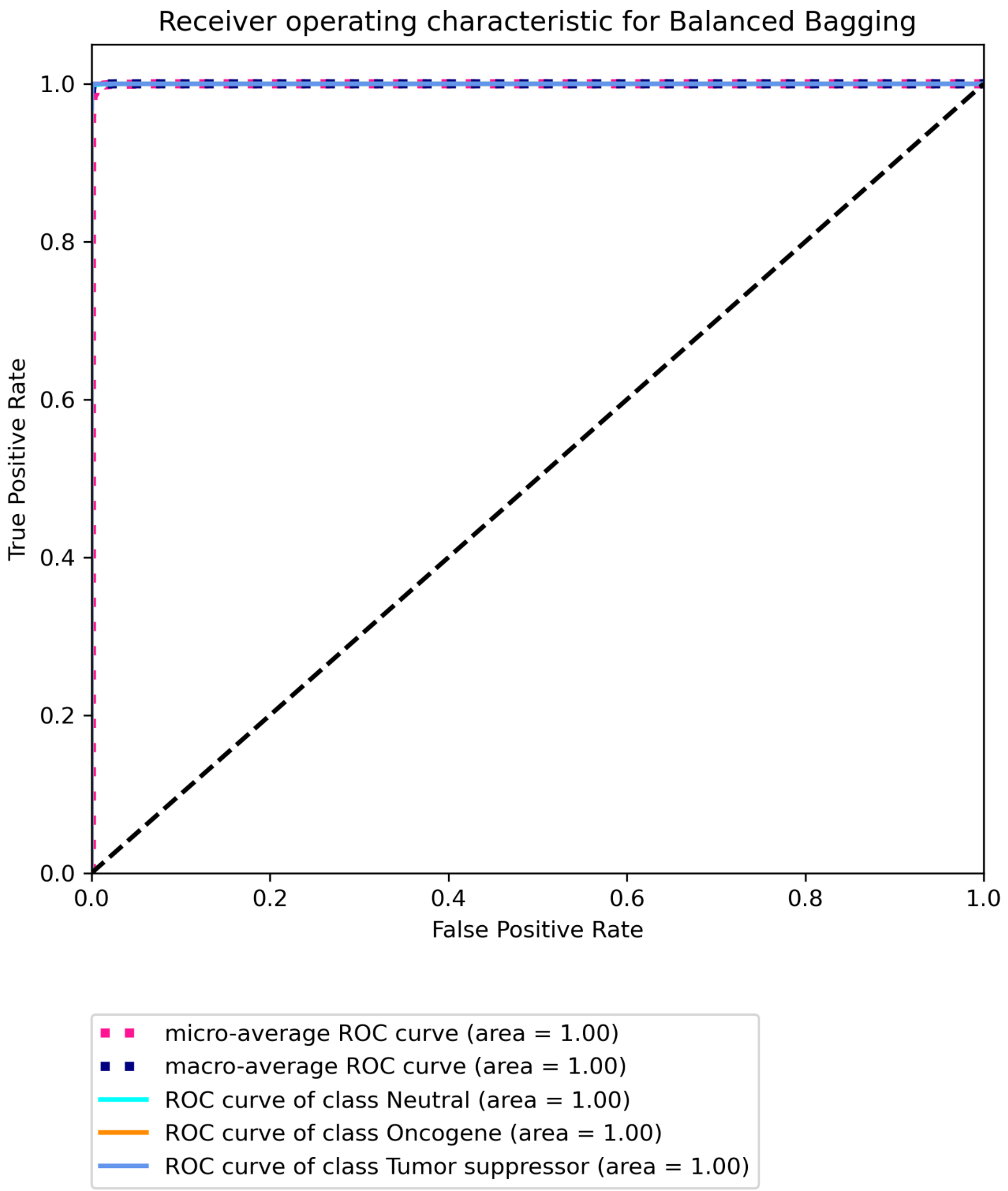

Figure S23C Receiver operating characteristic for COAD using RNA data with Bailey *et al.* labels built using Balanced bagging.

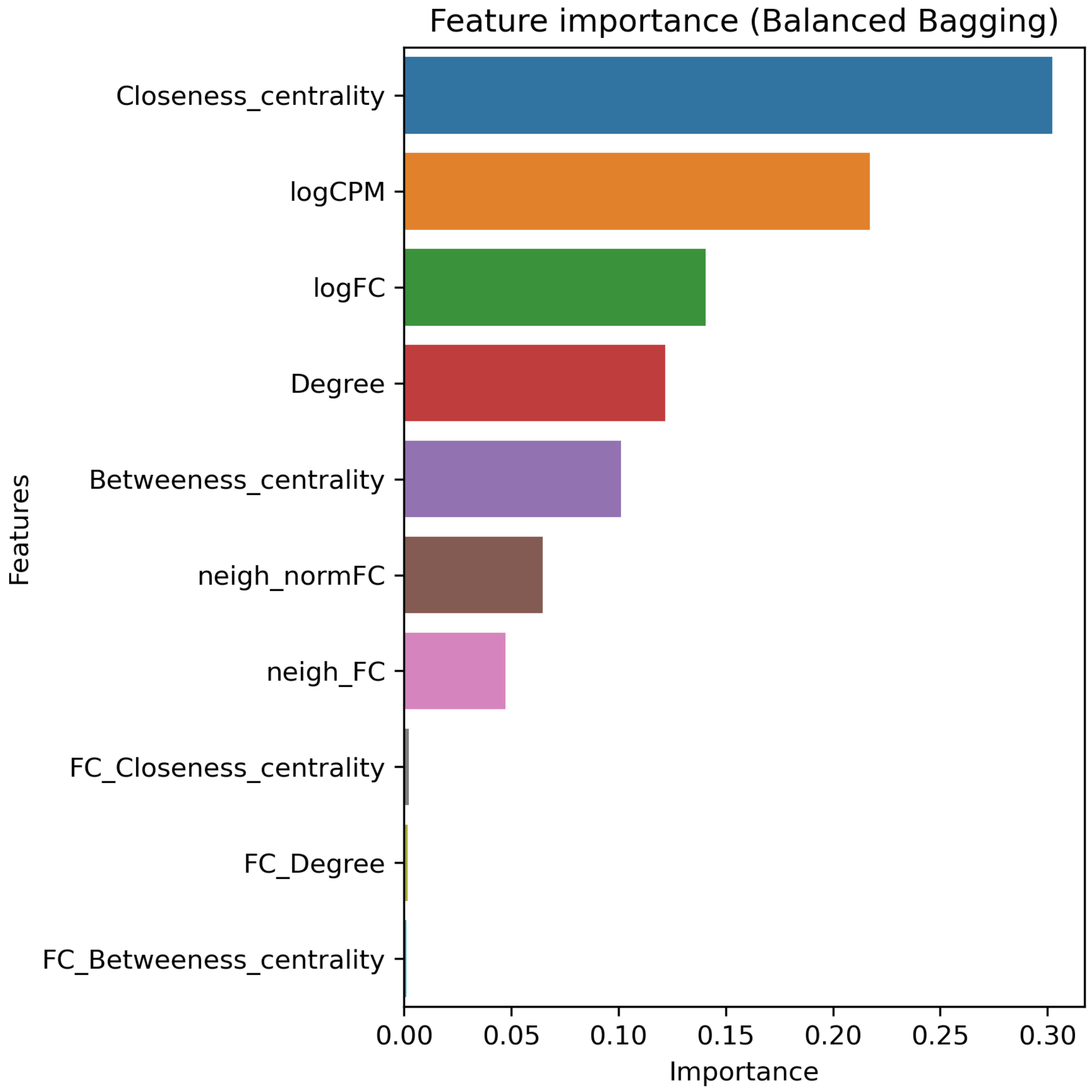

Figure S24A. Top 20 features for COAD using RNA data with CGC labels built using Balanced bagging.

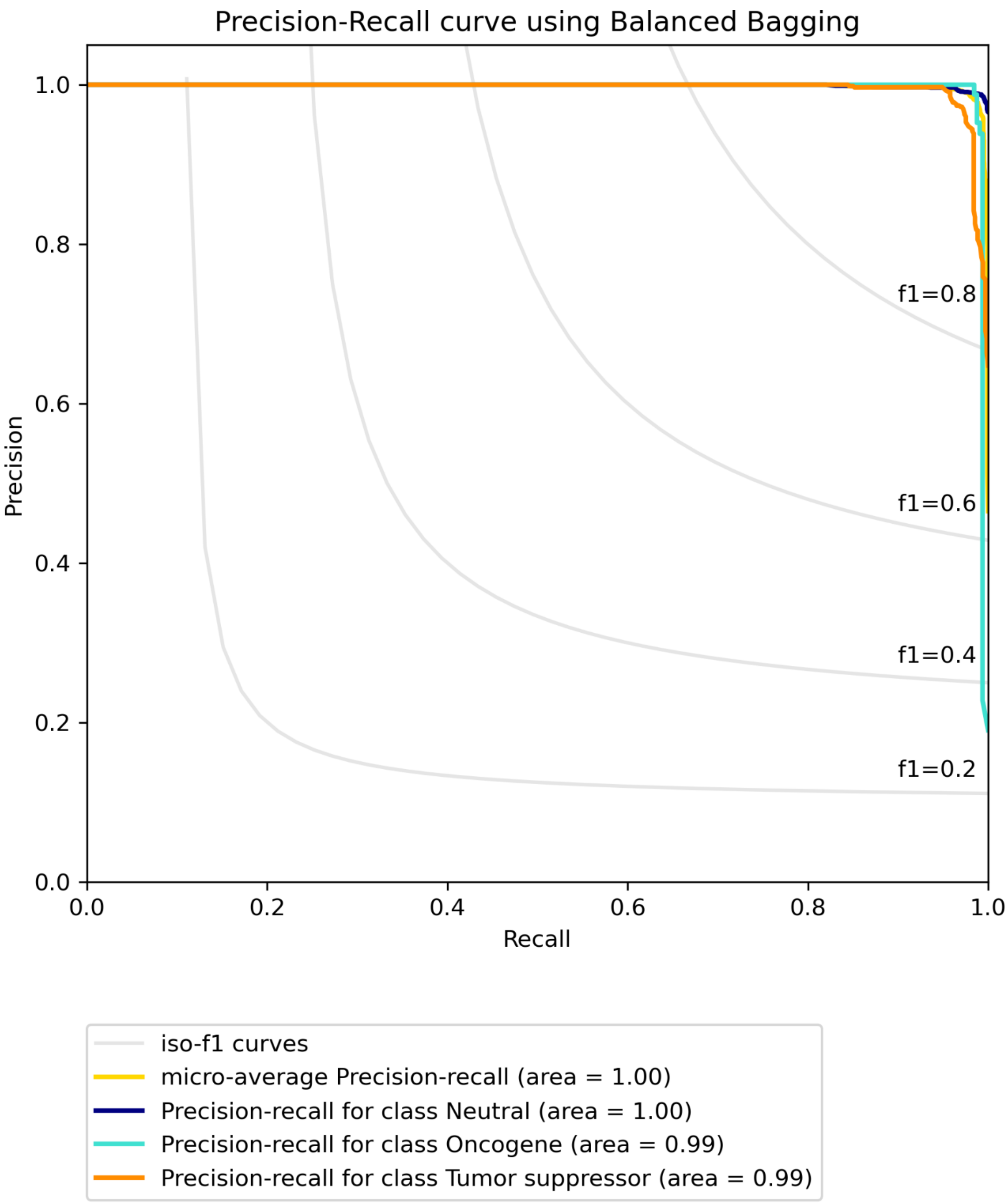

Figure S24B. Precision-recall curve for COAD using RNA data with CGC labels built using Balanced bagging.

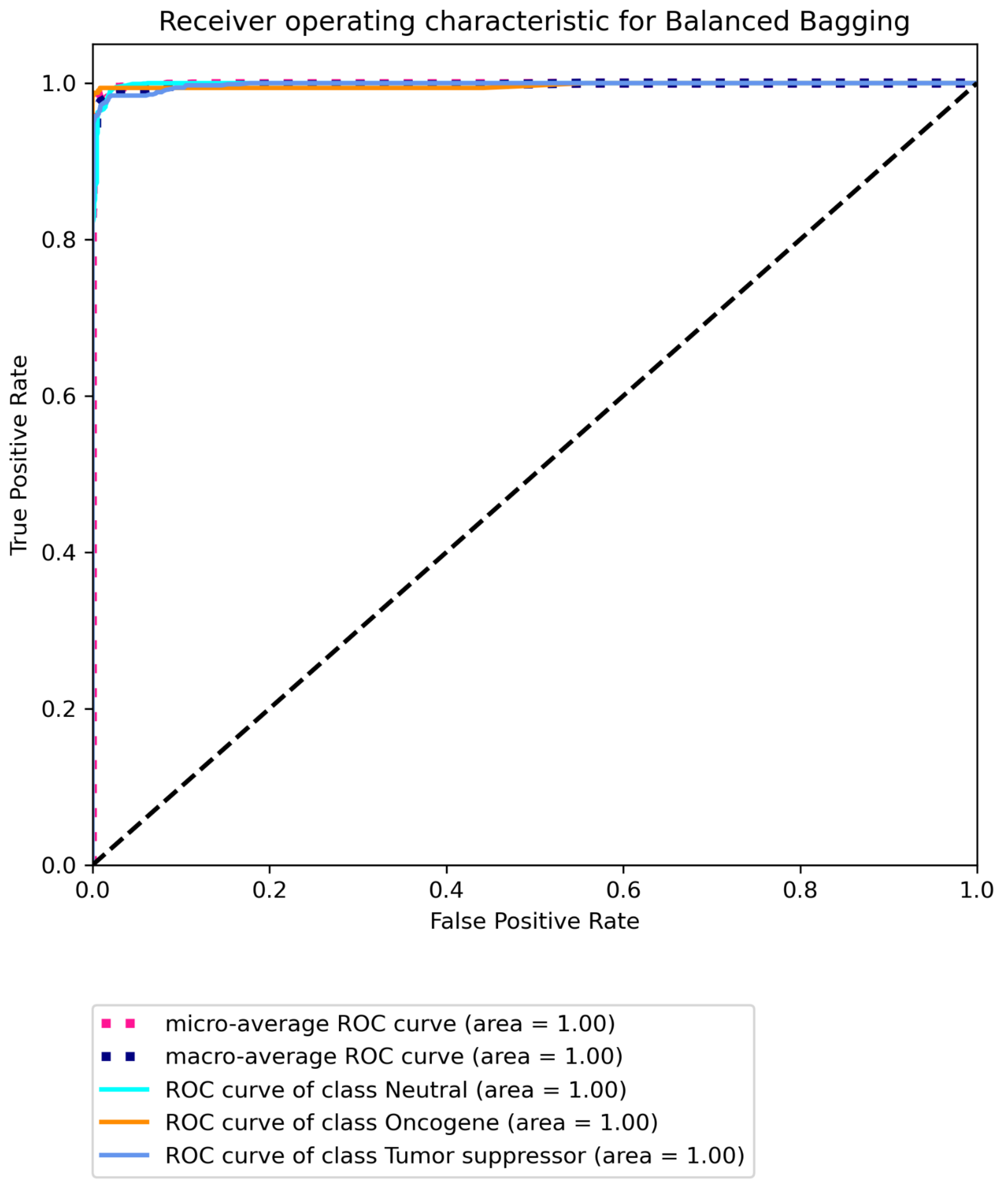

Figure S24C Receiver operating characteristic for COAD using RNA data with CGC labels built using Balanced bagging.

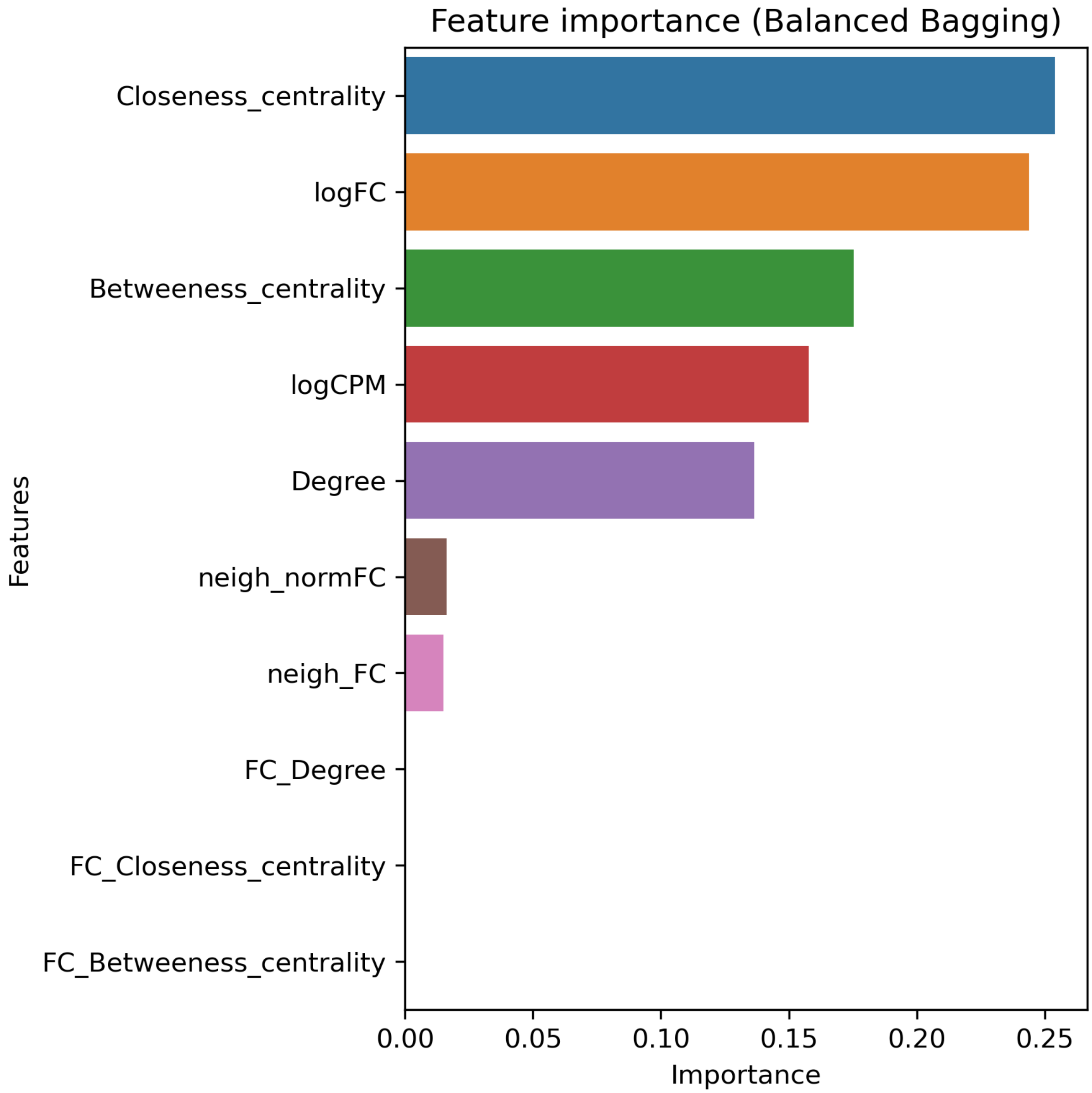

Figure S25A. Top 20 features for LUAD using RNA data with Bailey *et al.* labels built using Balanced bagging.

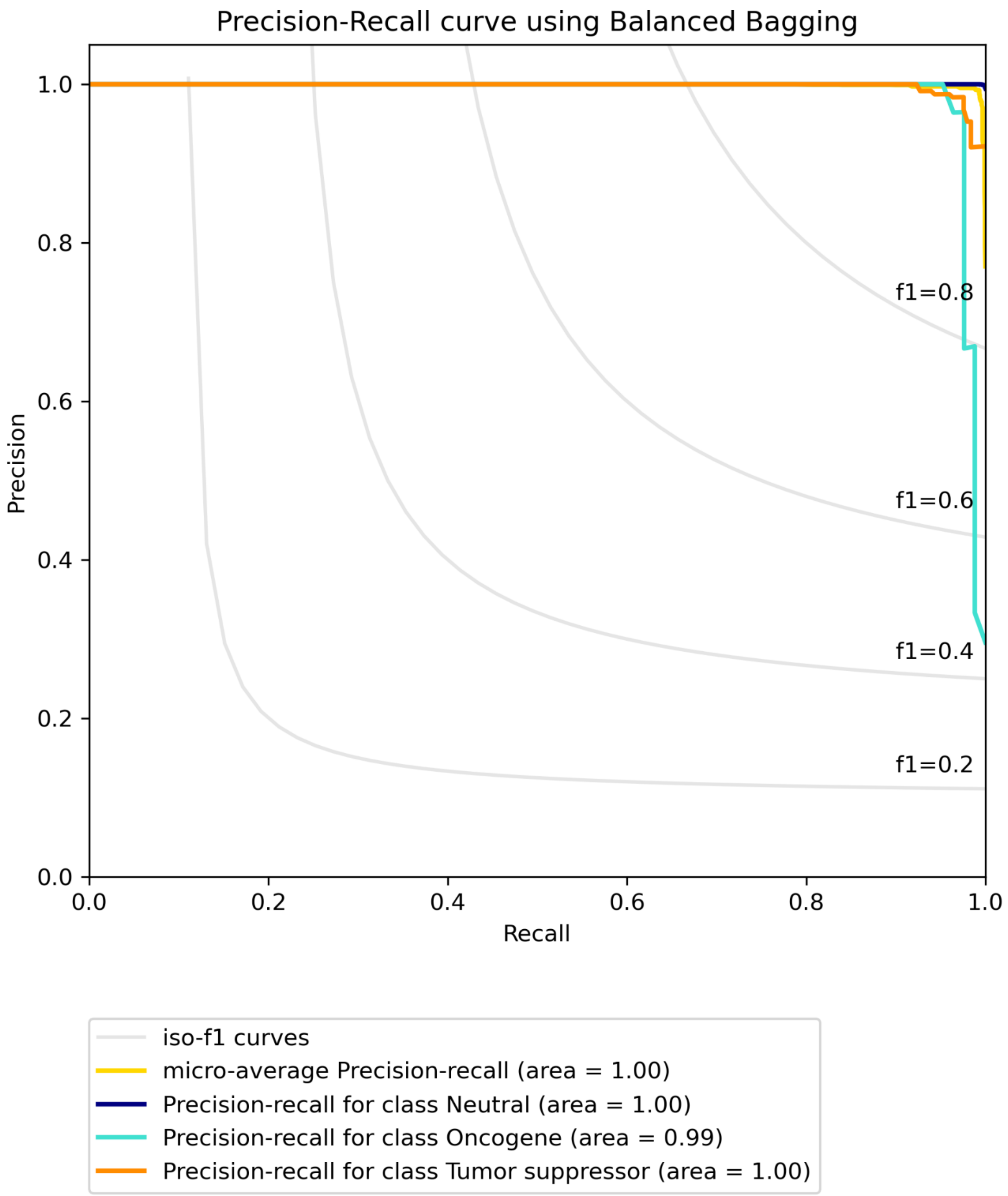

Figure S25B. Precision-recall curve for LUAD using RNA data with Bailey *et al.* labels built using Balanced bagging.

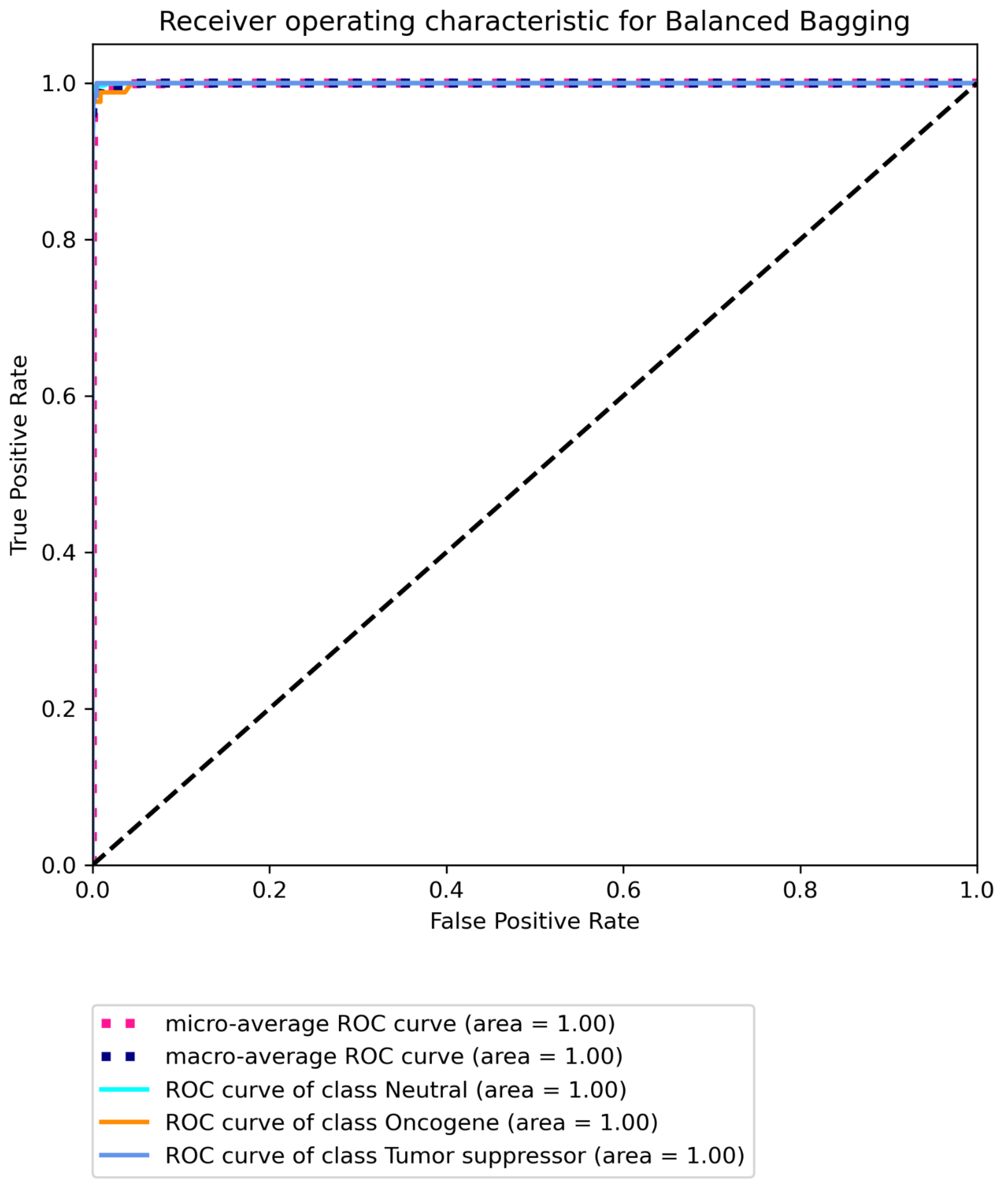

Figure S25C Receiver operating characteristic for LUAD using RNA data with Bailey *et al.* labels built using Balanced bagging.

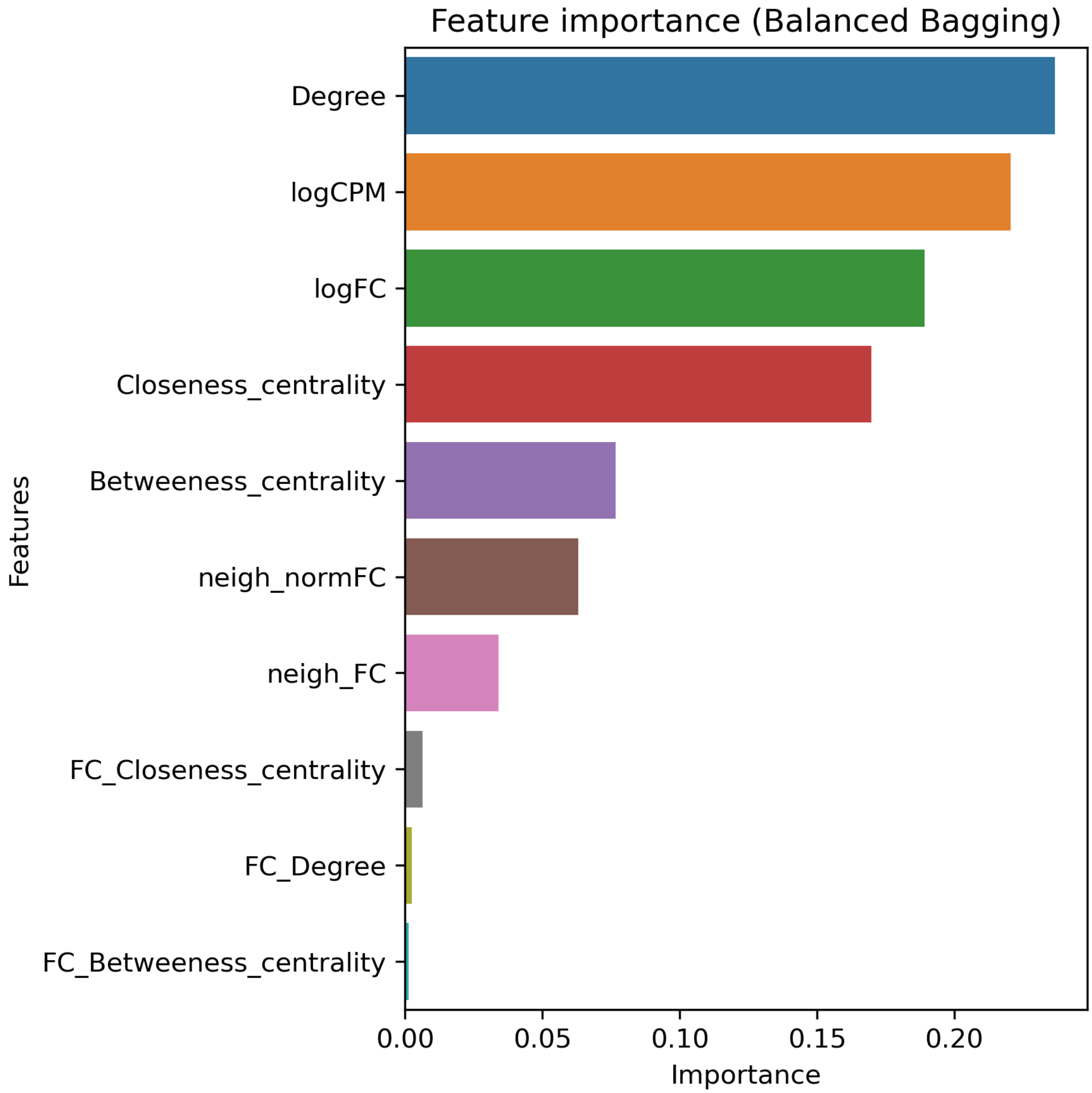

Figure S26A. Top 20 features for LUAD using RNA data with CGC labels built using Balanced bagging.

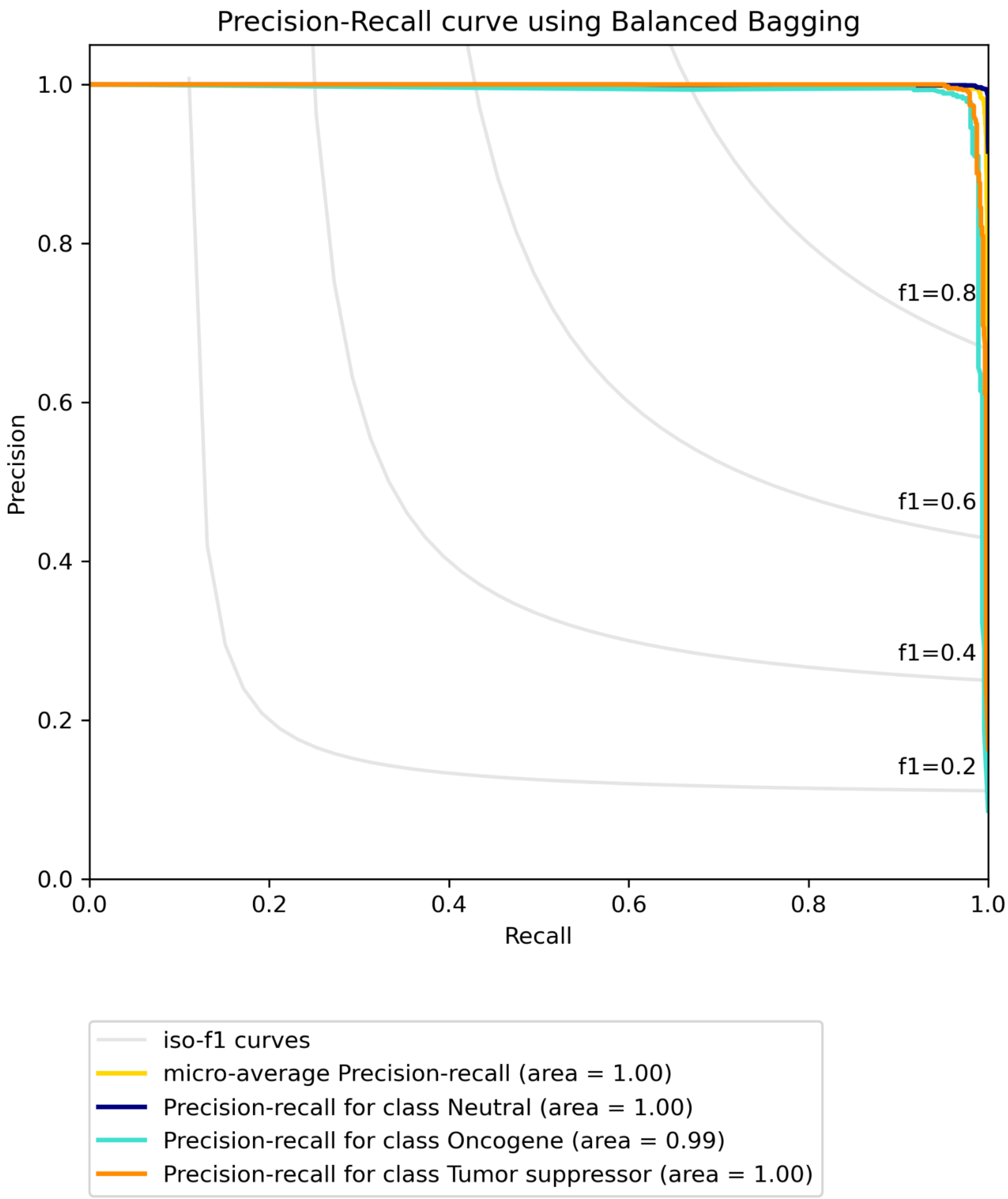

Figure S26B. Precision-recall curve for LUAD using RNA data with CGC labels built using Balanced bagging.

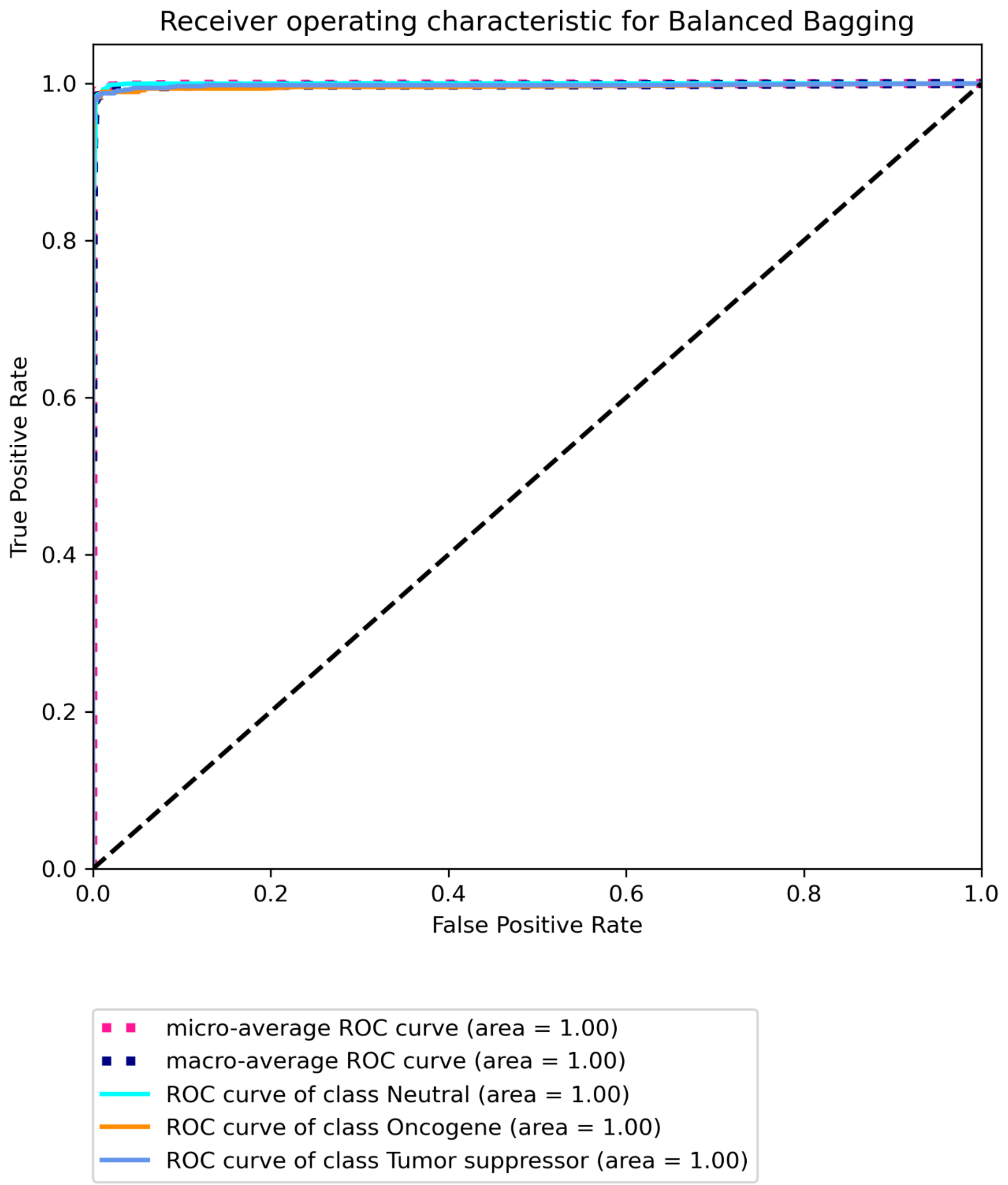

Figure S26C Receiver operating characteristic for LUAD using RNA data with CGC labels built using Balanced bagging.

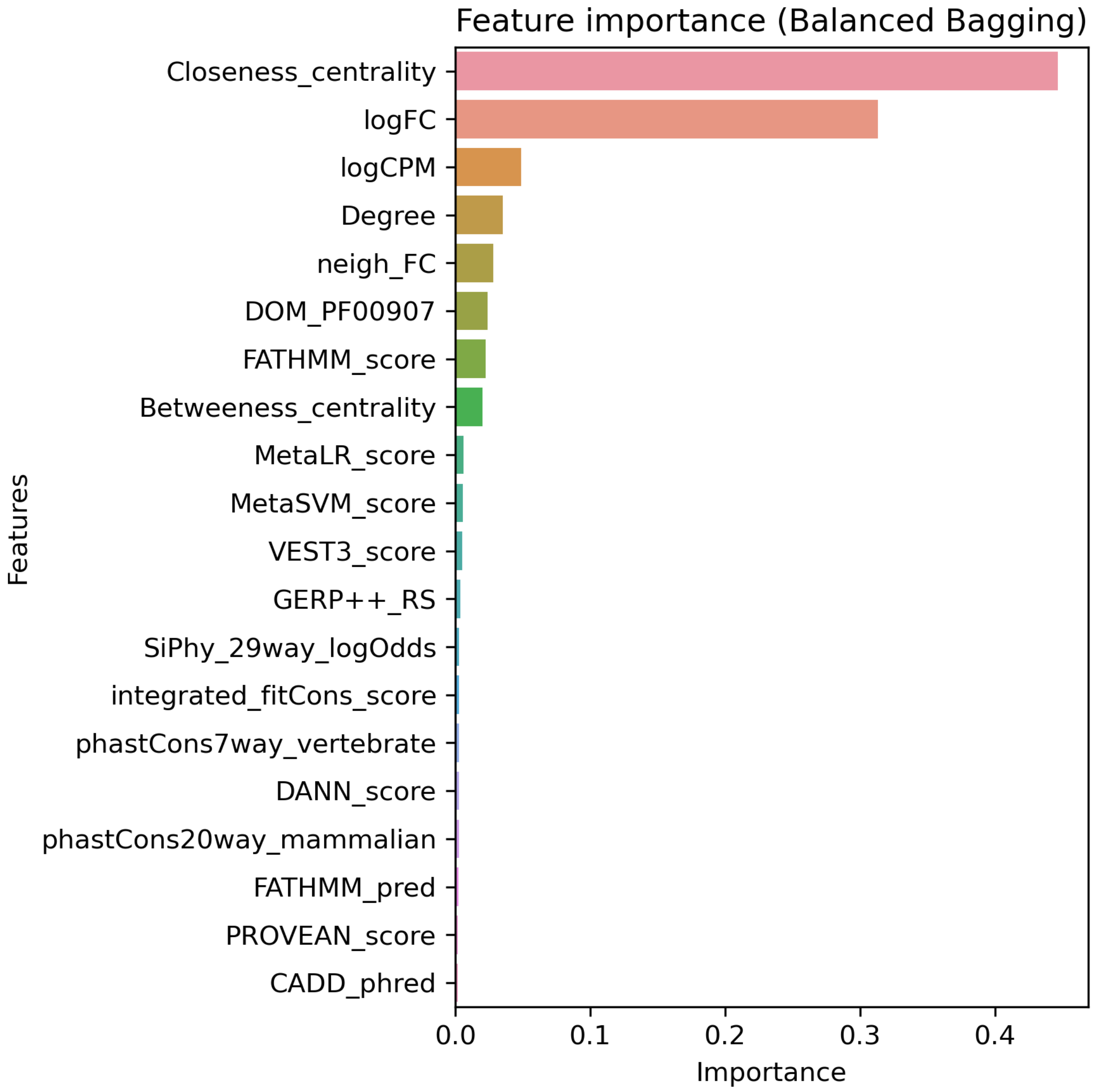

Figure S27A. Top 20 features for BRCA using multi-omic data with Bailey *et al.* labels built on "all" features using Balanced bagging.

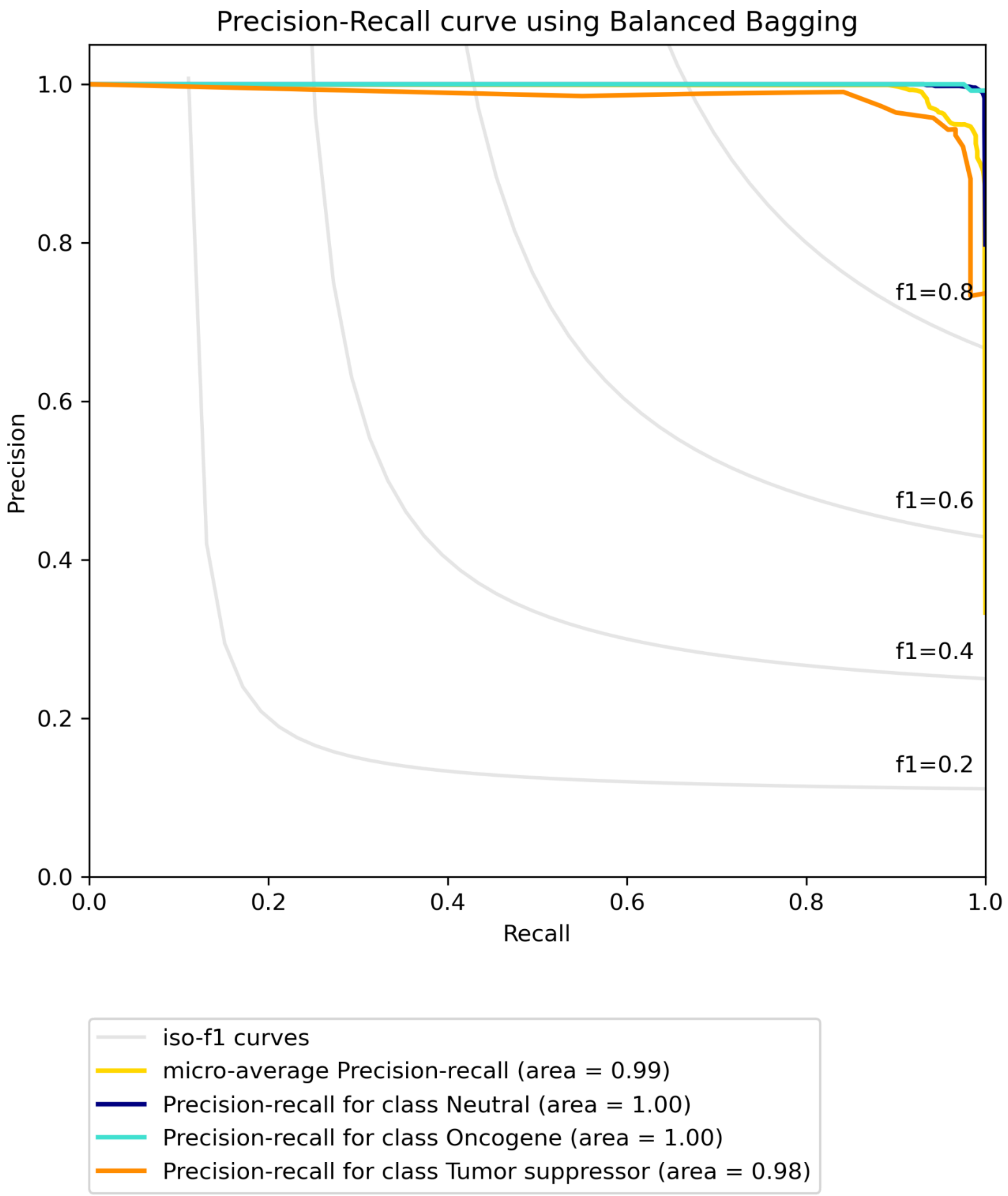

Figure S27B. Precision-recall curve for BRCA using multi-omic data with Bailey *et al.* labels built on "all" features using Balanced bagging.

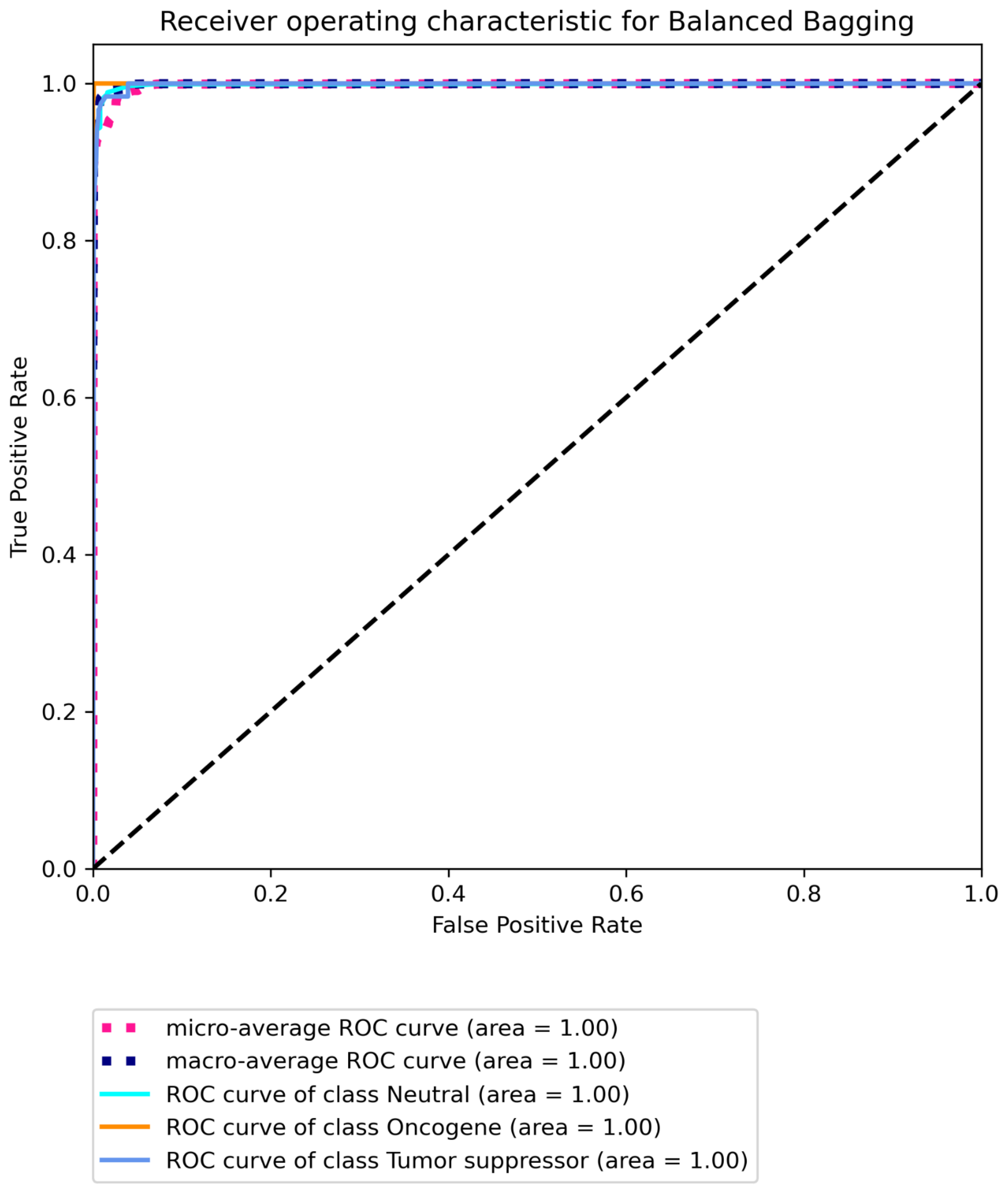

Figure S27C Receiver operating characteristic for BRCA using multi-omic data with Bailey *et al.* labels built on "all" features using Balanced bagging.

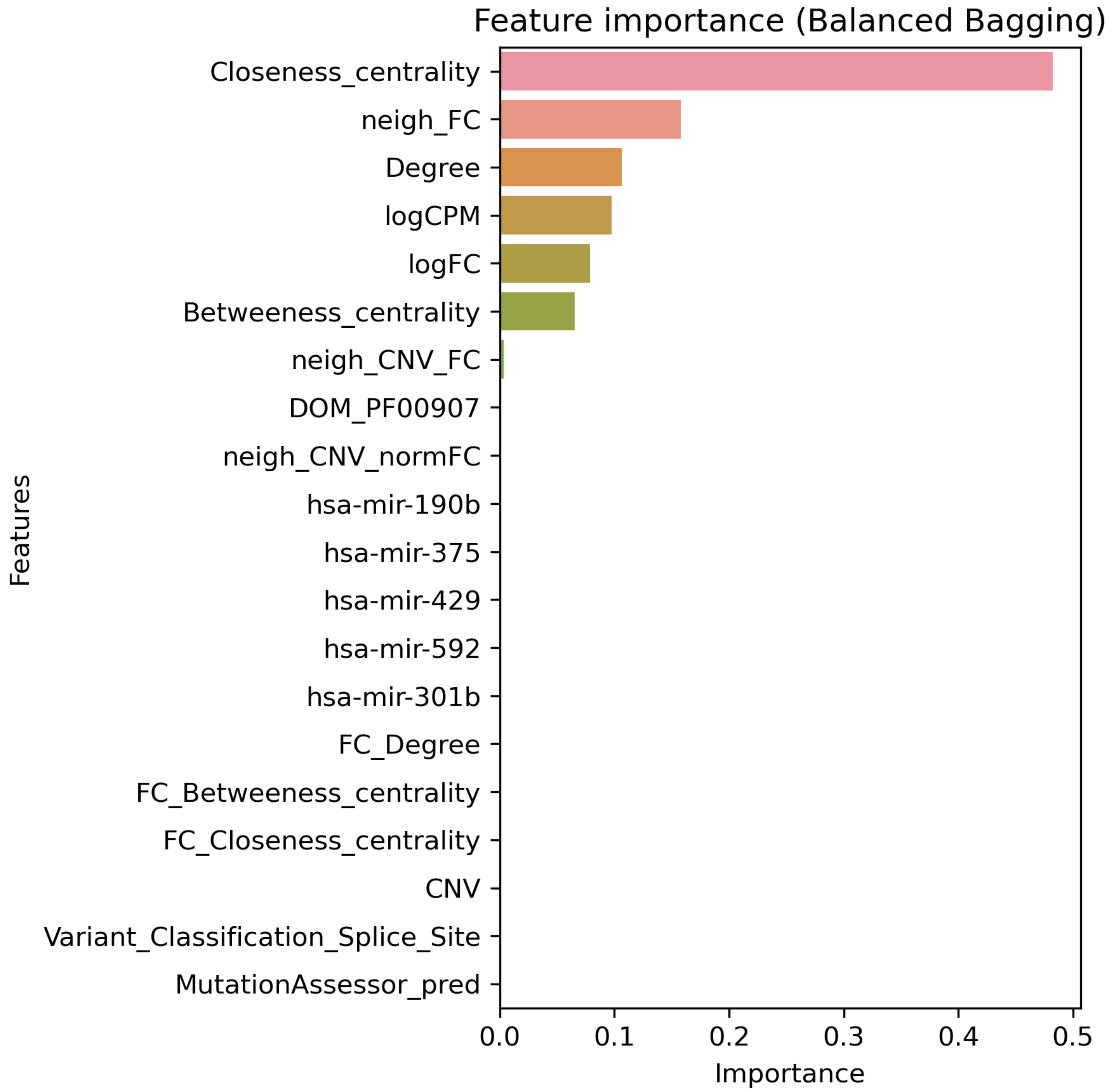

Figure S28A. Top 20 features for BRCA using multi-omic data with Bailey *et al.* labels built on "some" features using Balanced bagging.

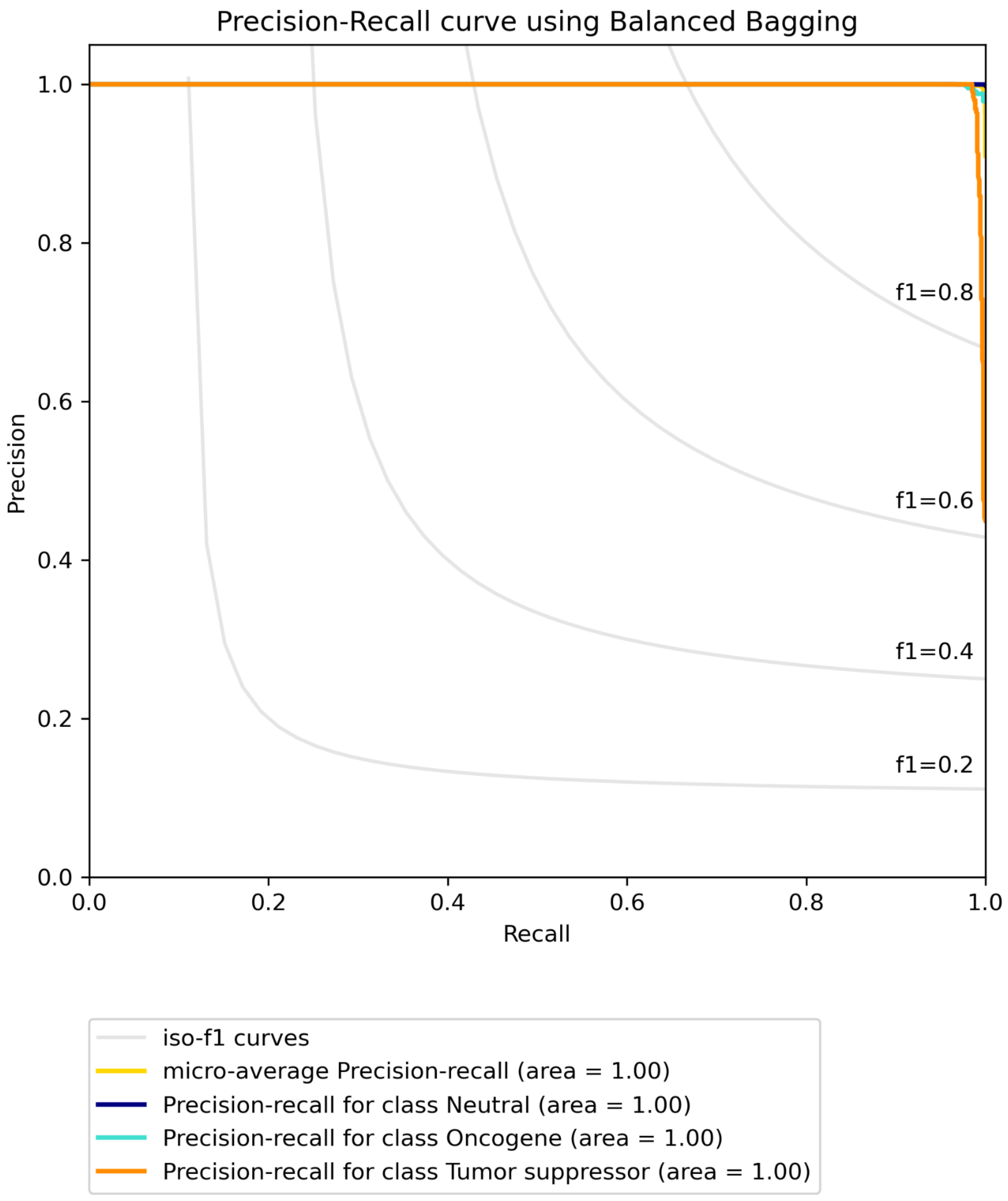

Figure S28B. Precision-recall curve for BRCA using multi-omic data with Bailey *et al.* labels built on "some" features using Balanced bagging.

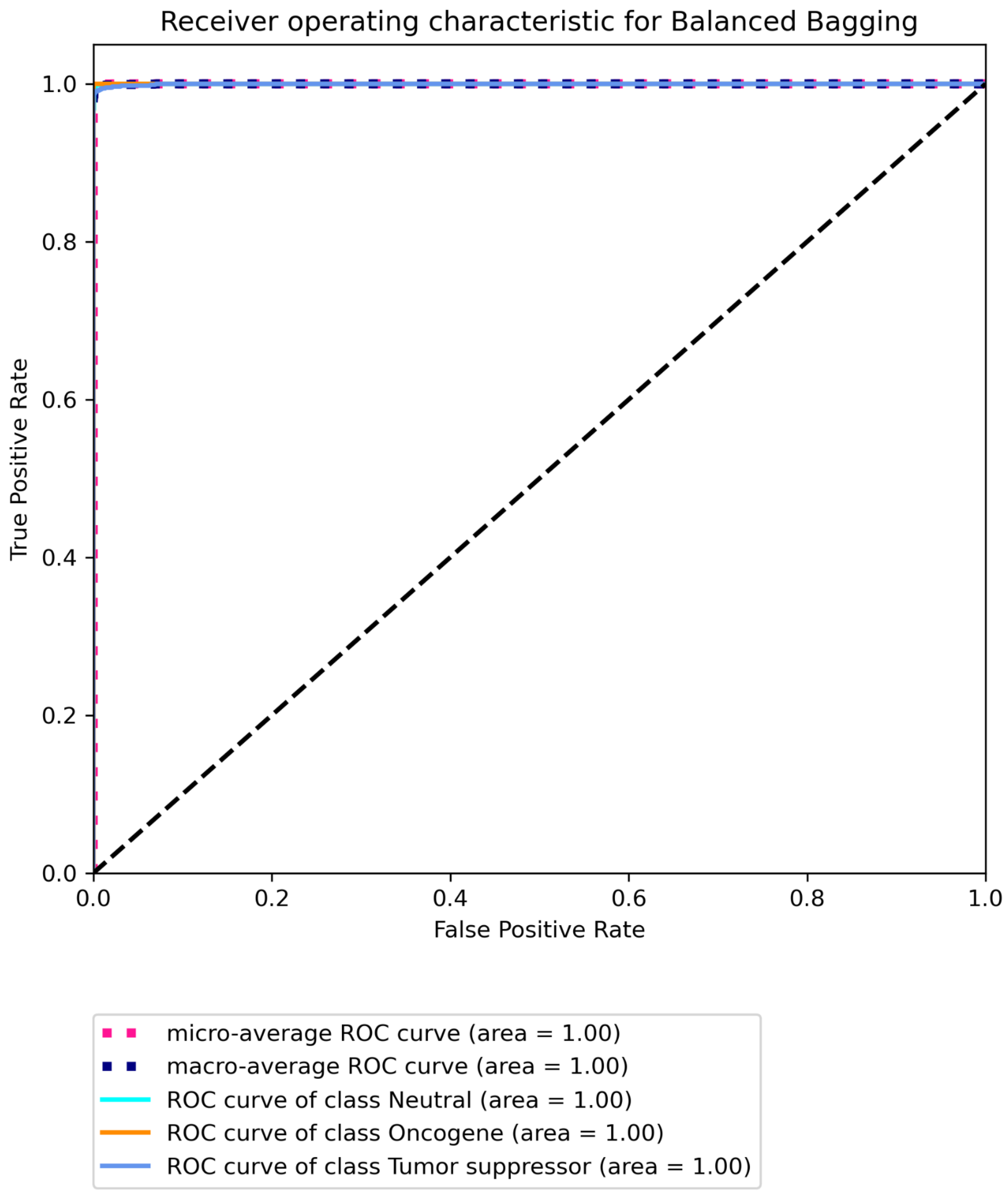

Figure S28C Receiver operating characteristic for BRCA using multi-omic data with Bailey *et al.* labels built on "some" features using Balanced bagging.

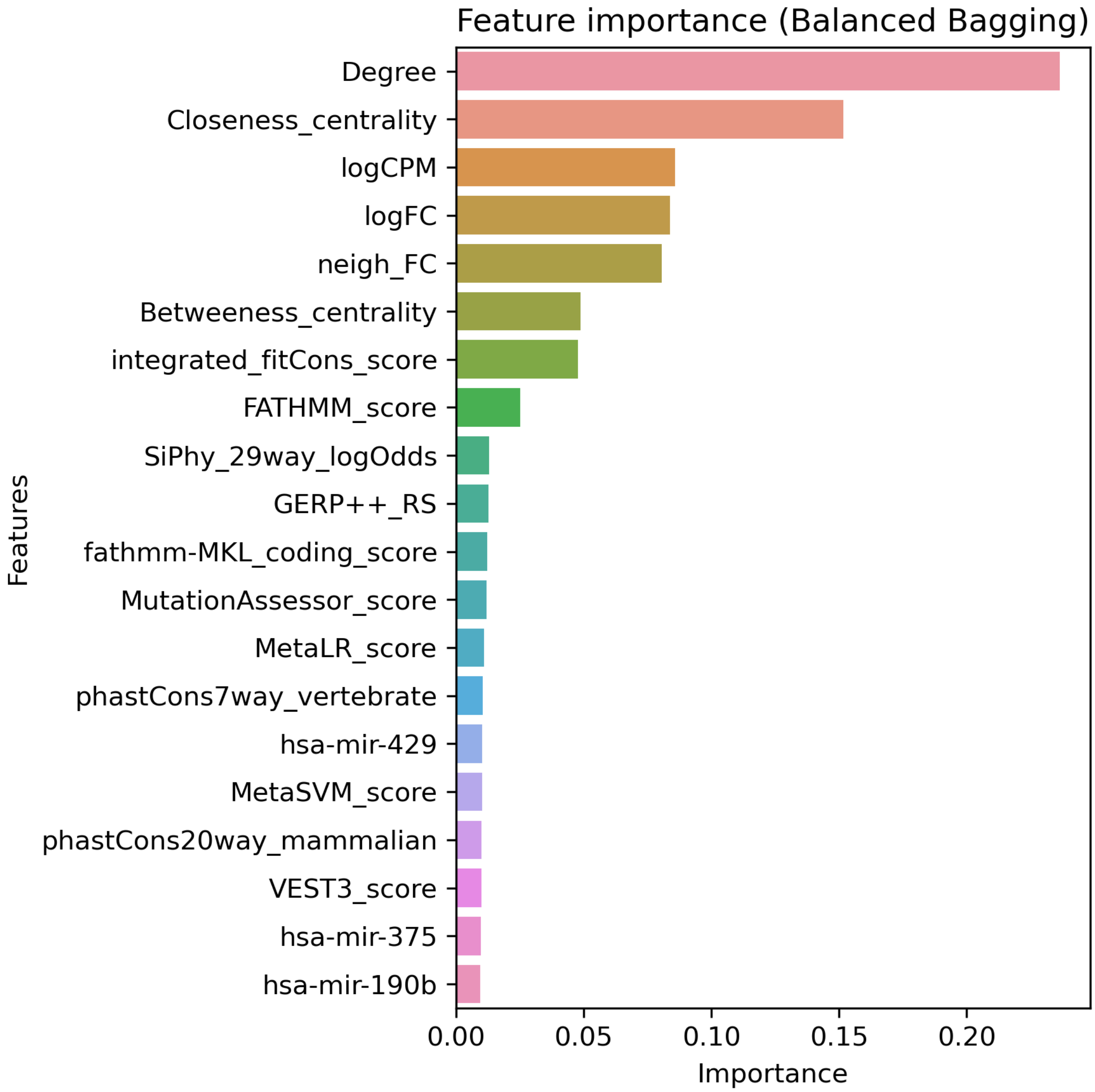

Figure S29A. Top 20 features for BRCA using multi-omic data with CGC labels built on "all" features using Balanced bagging.

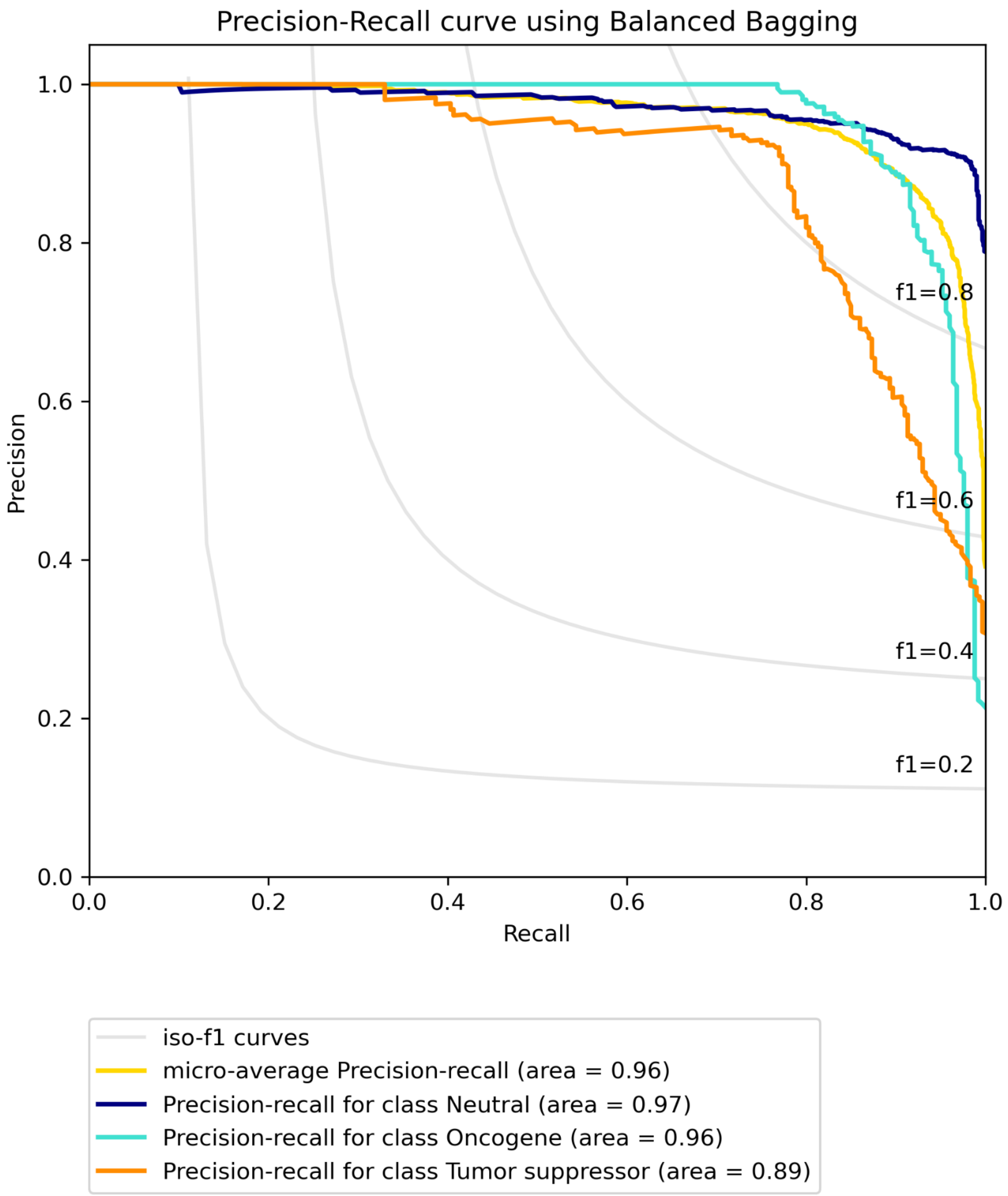

Figure S29B. Precision-recall curve for BRCA using multi-omic data with CGC labels built on “all” features using Balanced bagging.

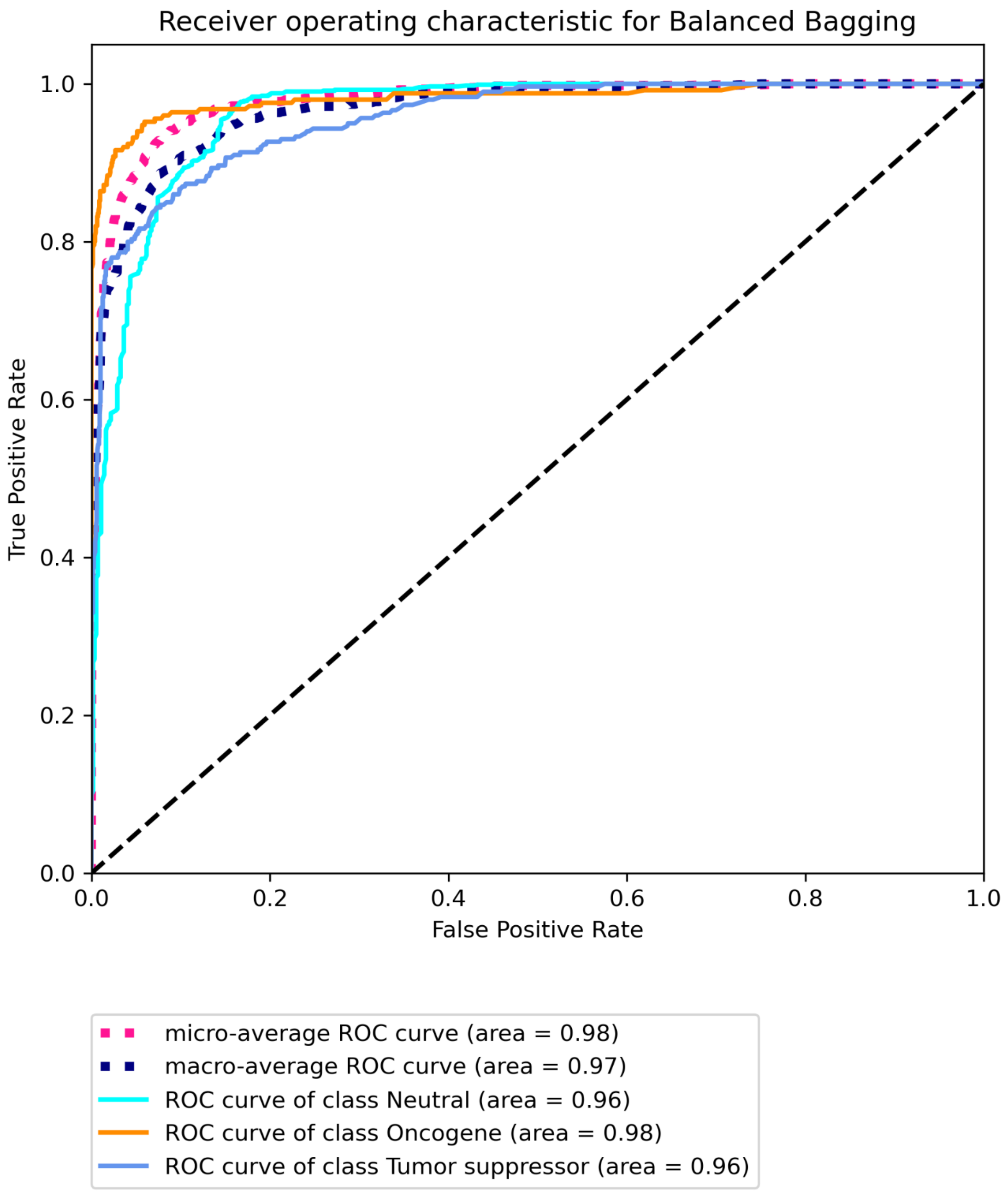

Figure S29C. Receiver operating characteristic for BRCA using multi-omic data with CGC labels built on "all" features using Balanced Bagging.

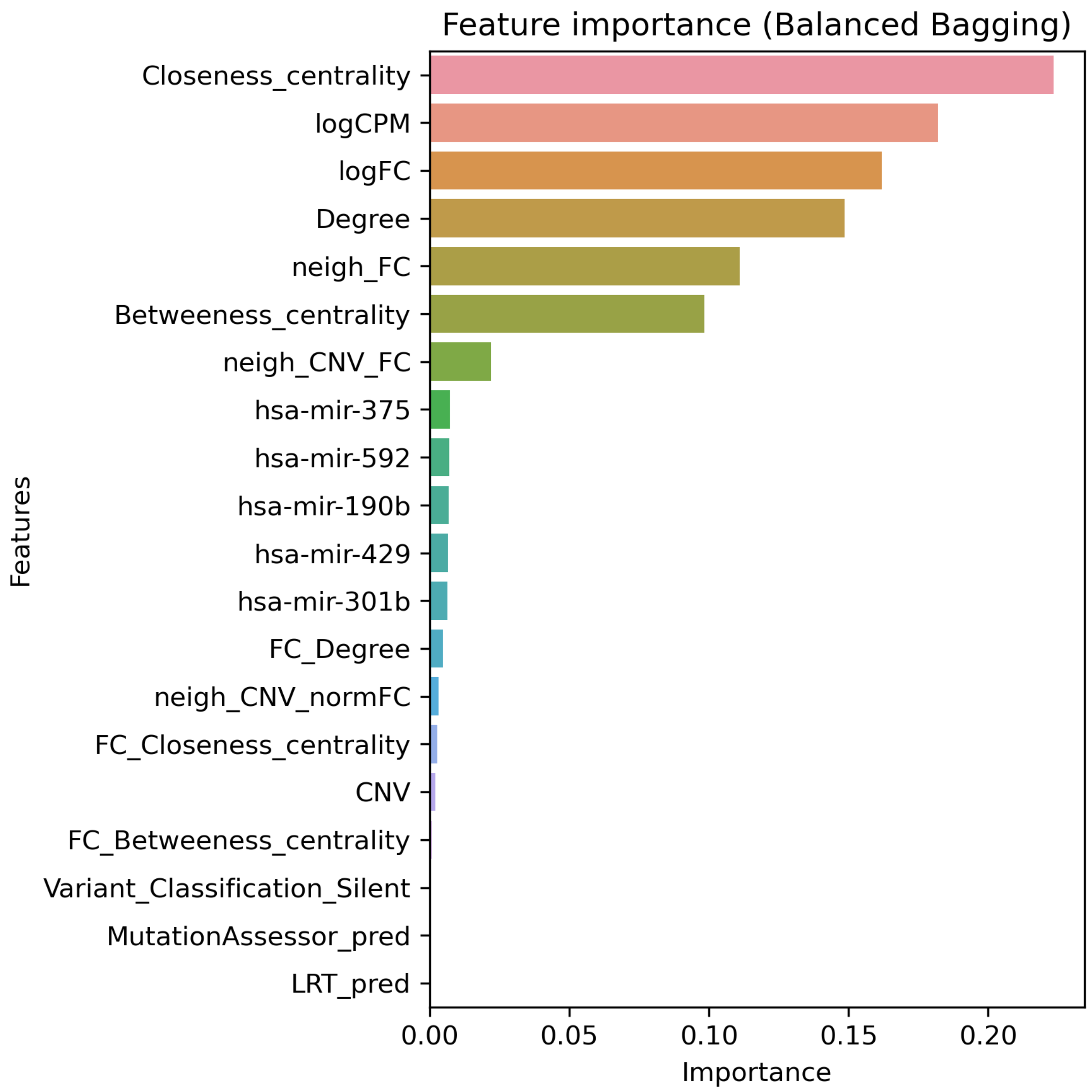

Figure S30A. Top 20 features for BRCA using multi-omic data with CGC labels built on "some" features using Balanced Bagging.

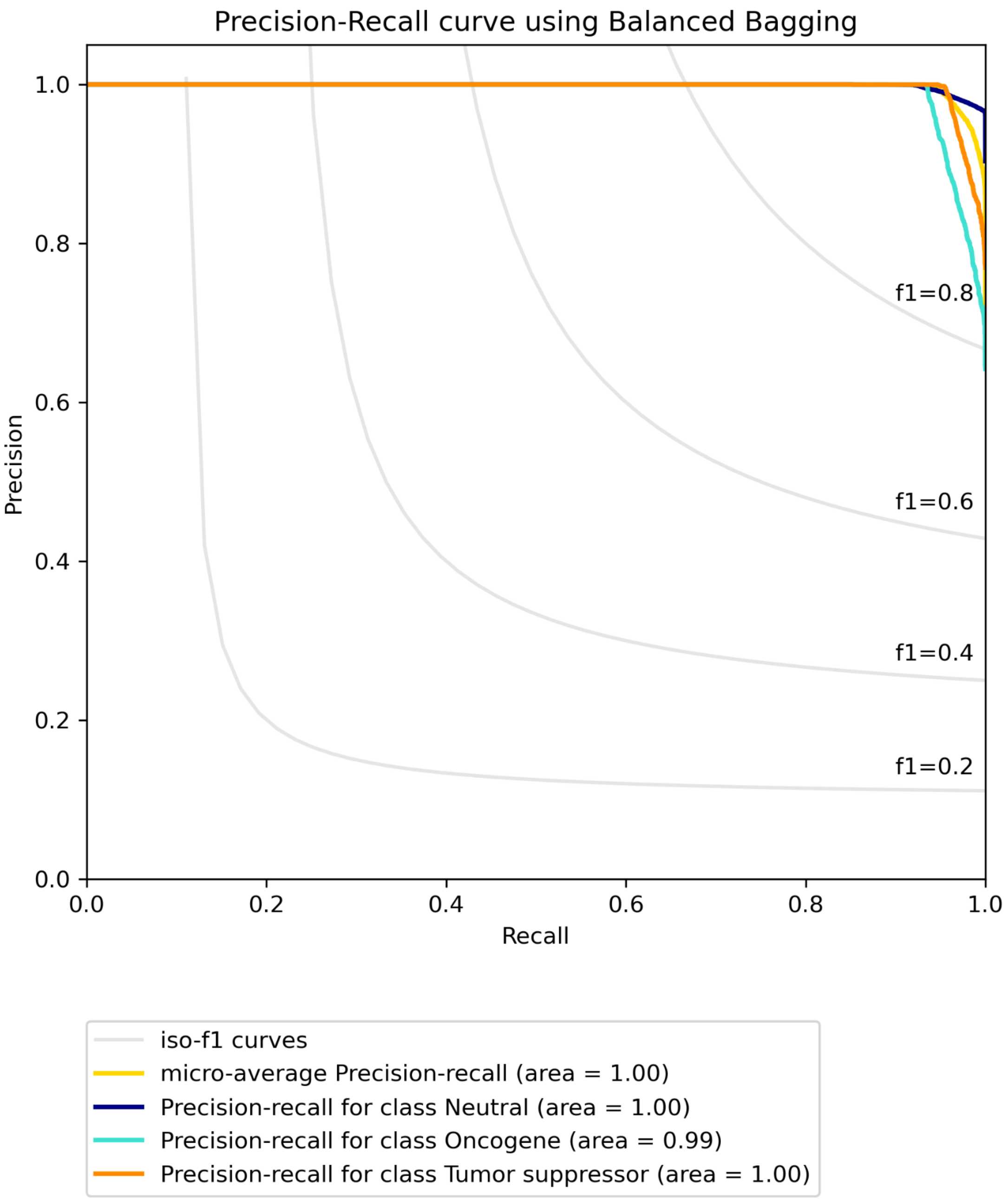

Figure S30B. Precision-recall curve for BRCA using multi-omic data with CGC labels built on “some” features using Balanced Bagging.

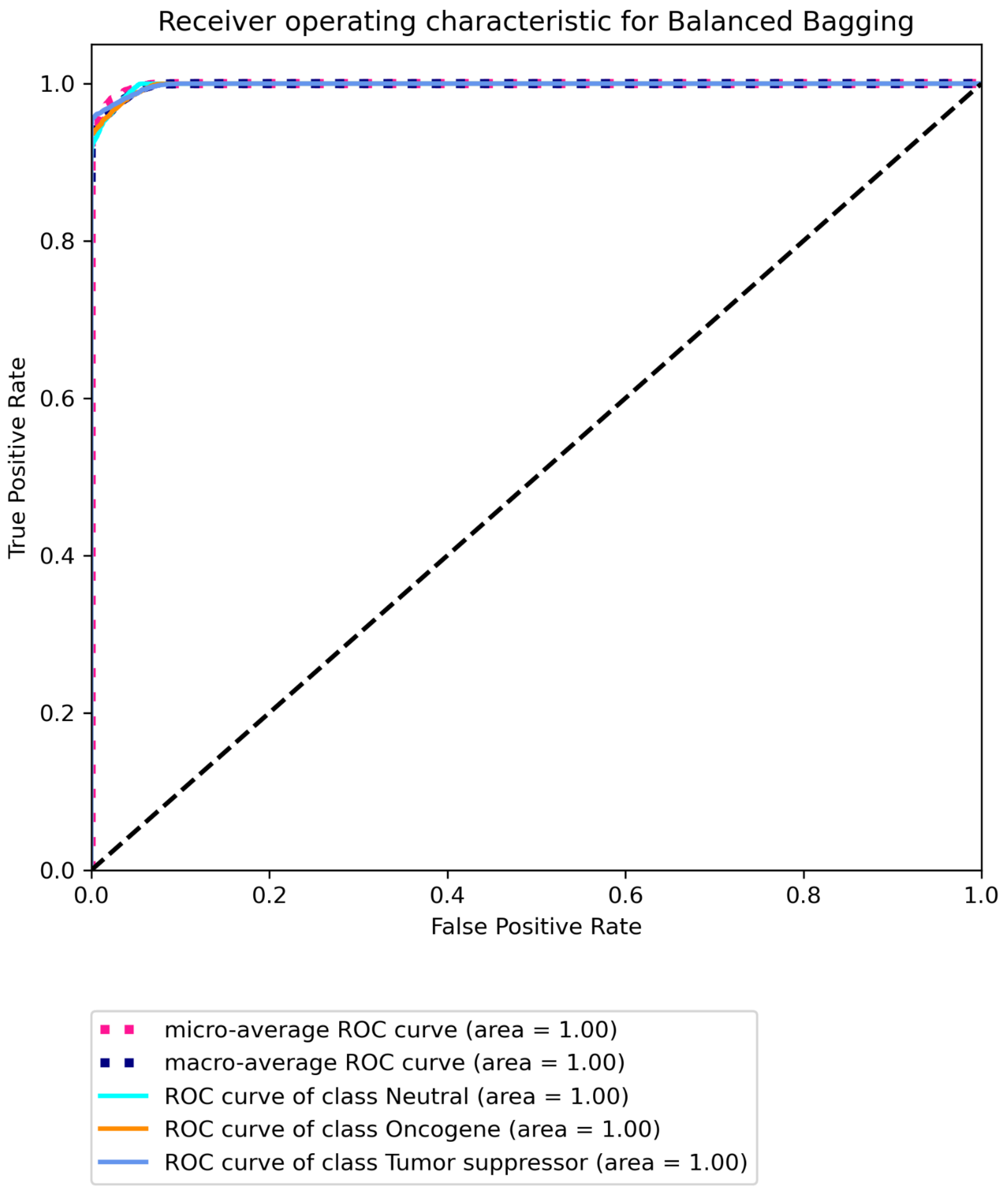

Figure S30C. Receiver operating characteristic for BRCA using multi-omic data with CGC labels built on "some" features using Balanced Bagging.

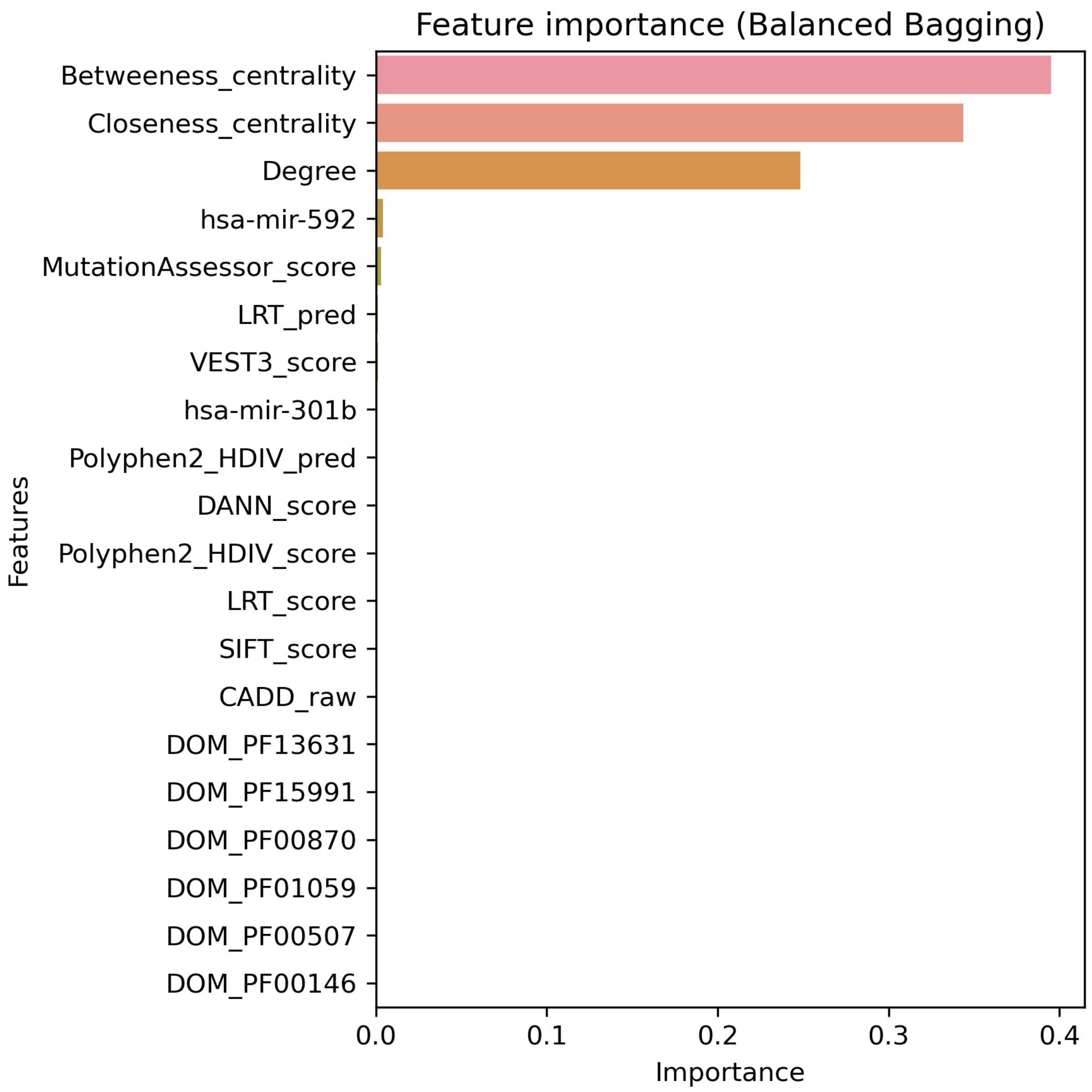

Figure S31A. Top 20 features for BRCA using multi-omic data with CIViC labels built on "all" features using Balanced Bagging.

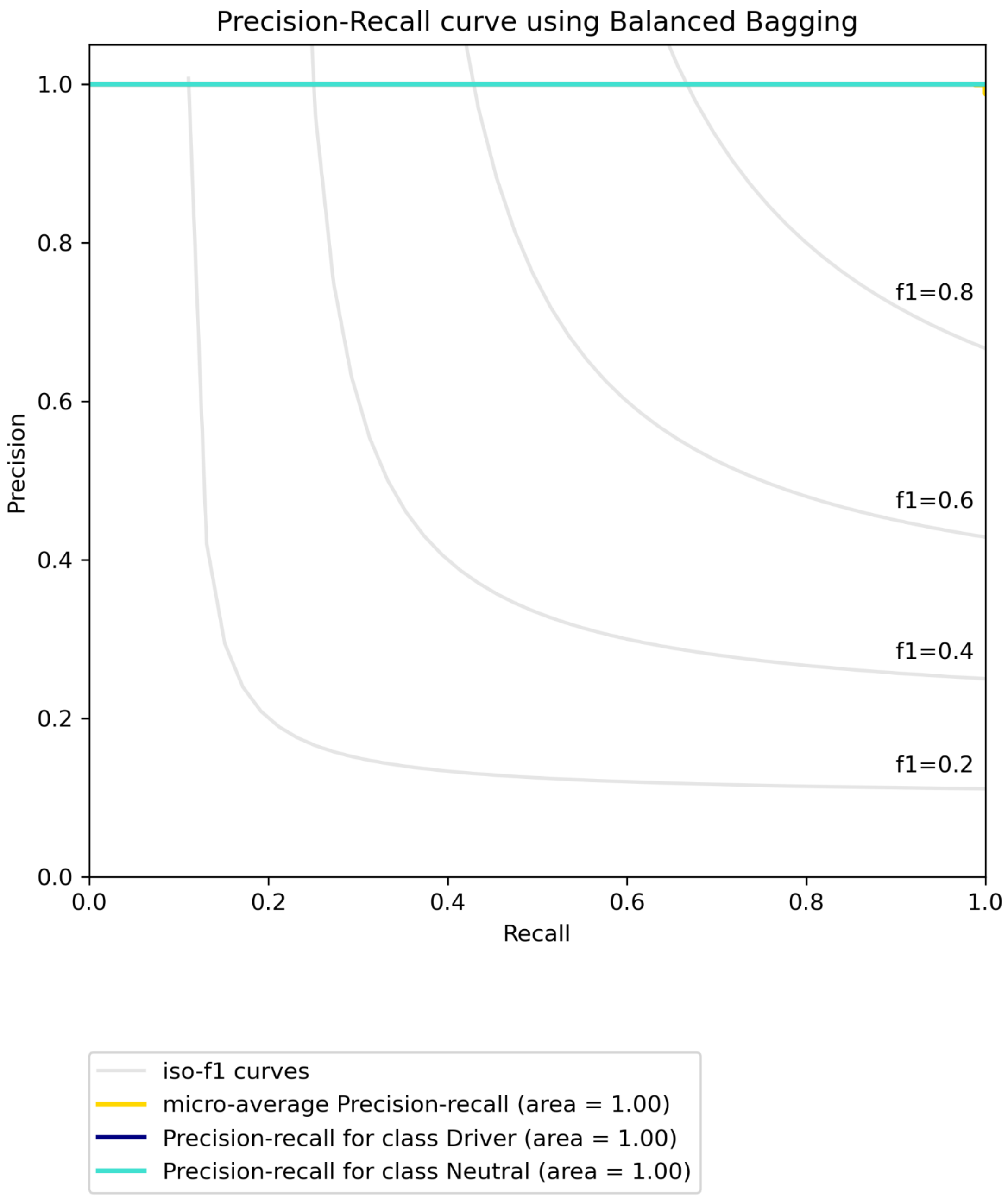

Figure S31B. Precision-recall curve for BRCA using multi-omic data with CIViC labels built on “all” features using Balanced Bagging.

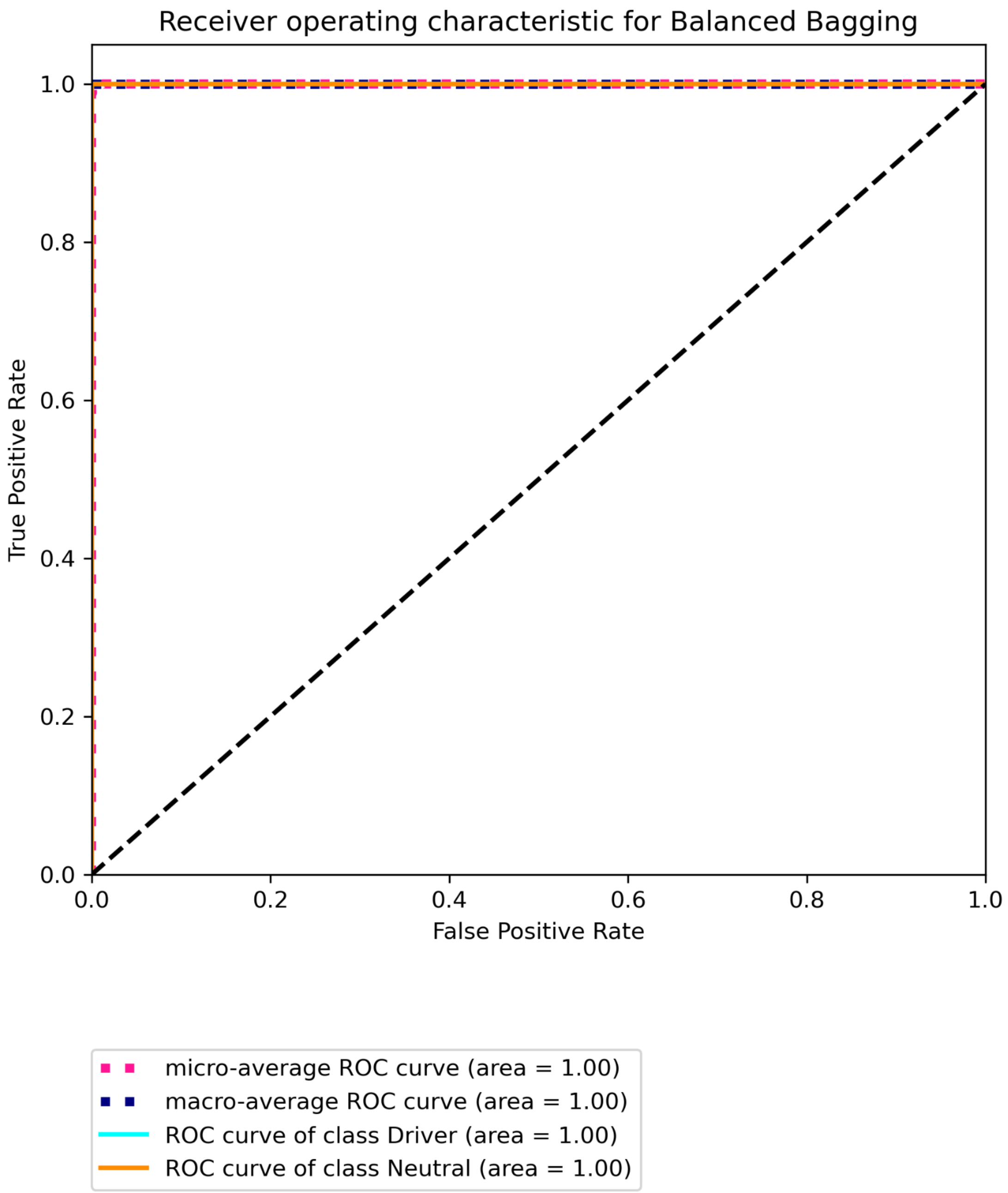

Figure S31C. Receiver operating characteristic for BRCA using multi-omic data with CIViC labels built on "all" features using Balanced Bagging.

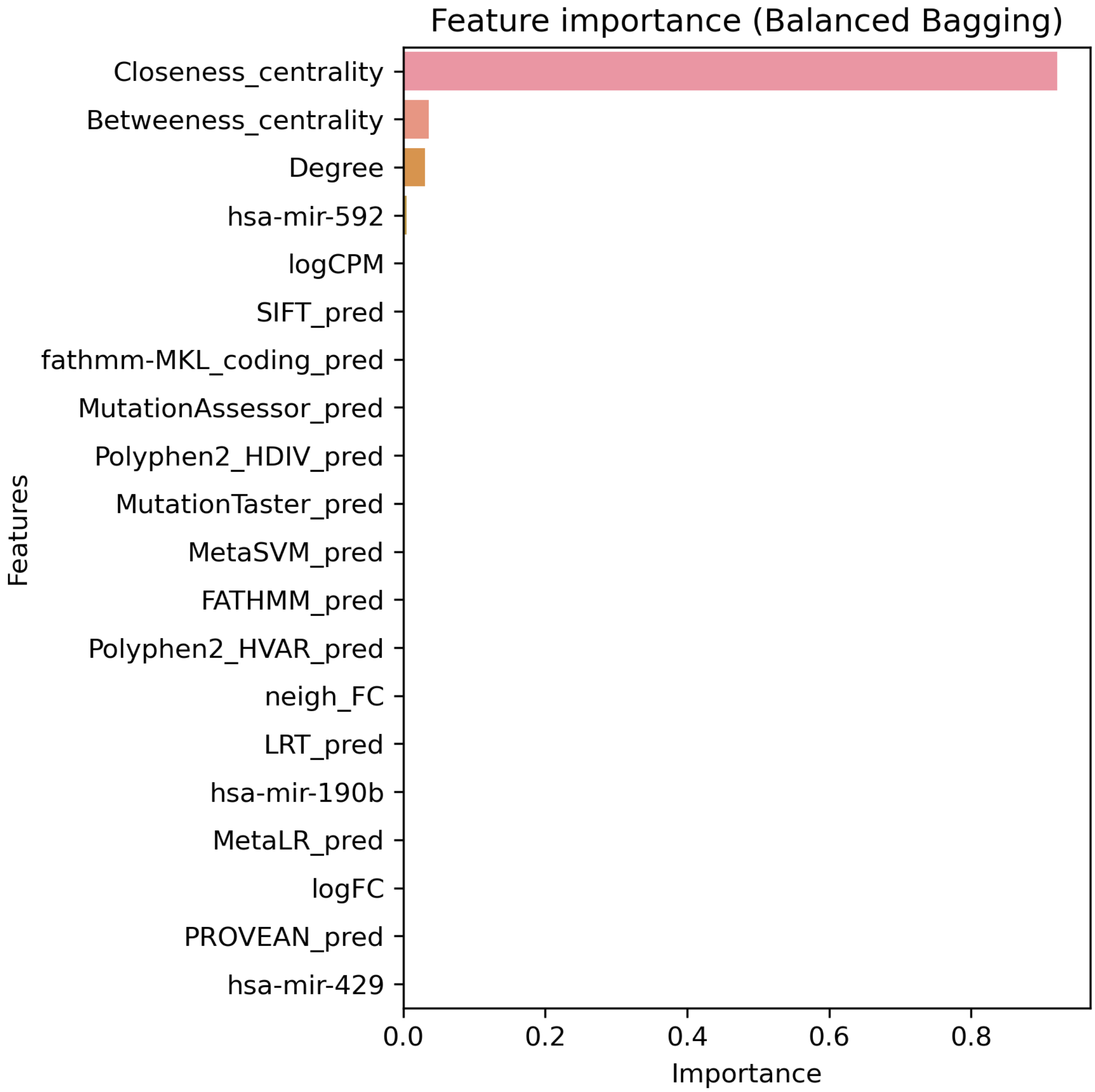

Figure S32A. Top 20 features for BRCA using multi-omic data with CIViC labels built on “some” features using Balanced Bagging.

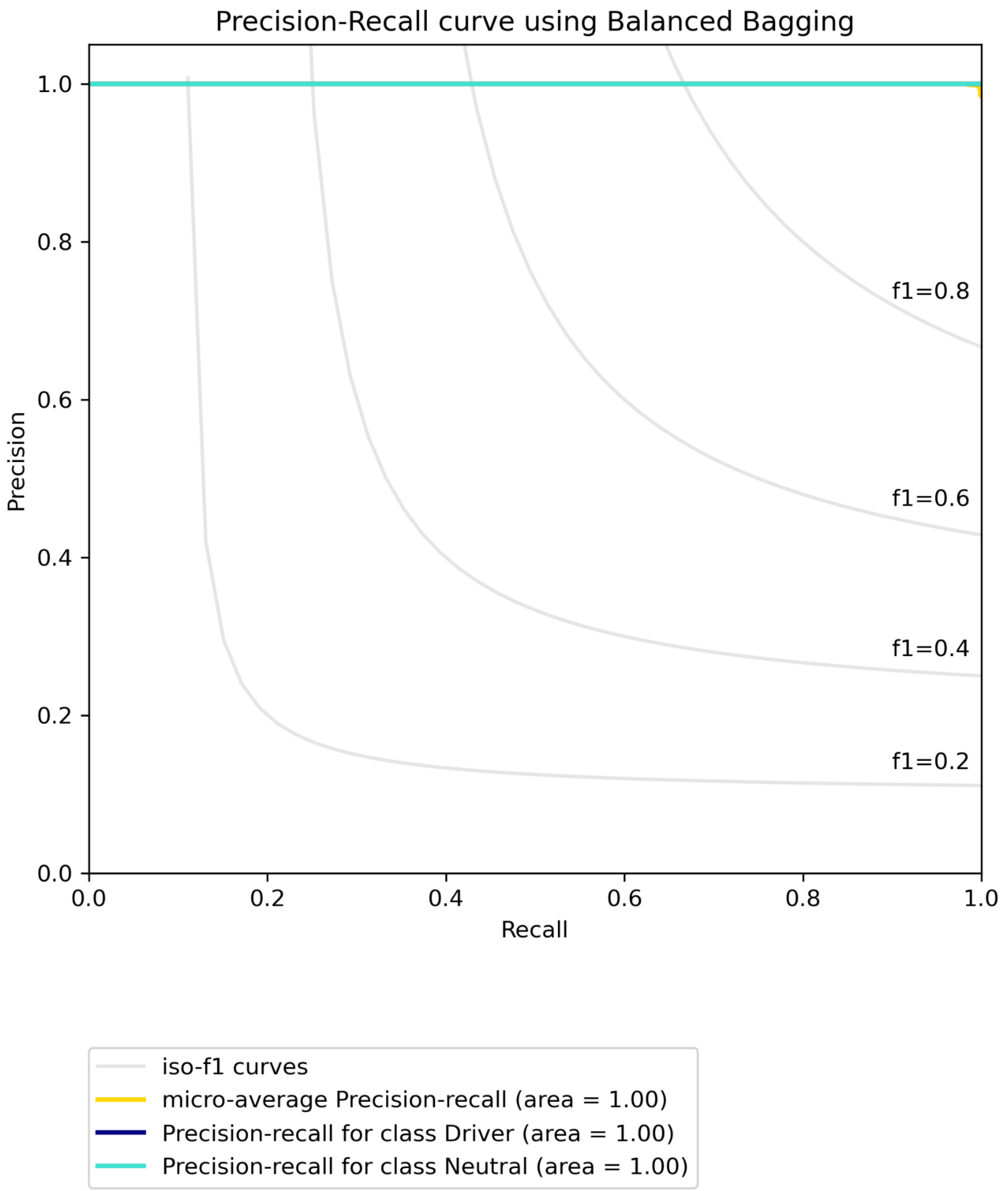

Figure S32B. Precision-recall curve for BRCA using multi-omic data with CIViC labels built on “some” features using Balanced Bagging.

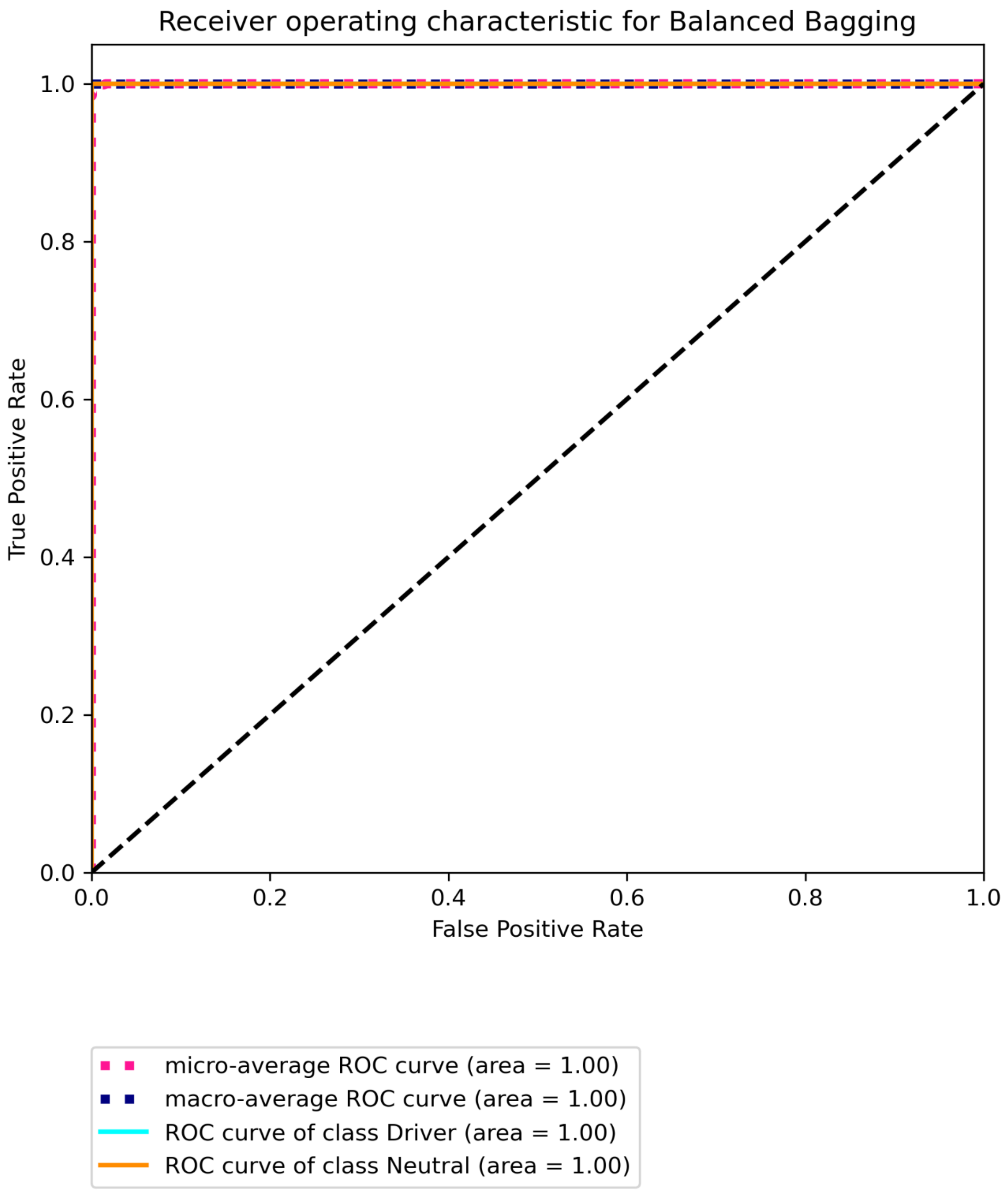

Figure S32C. Receiver operating characteristic for BRCA using multi-omic data with CIViC labels built on "some" features using Balanced Bagging.

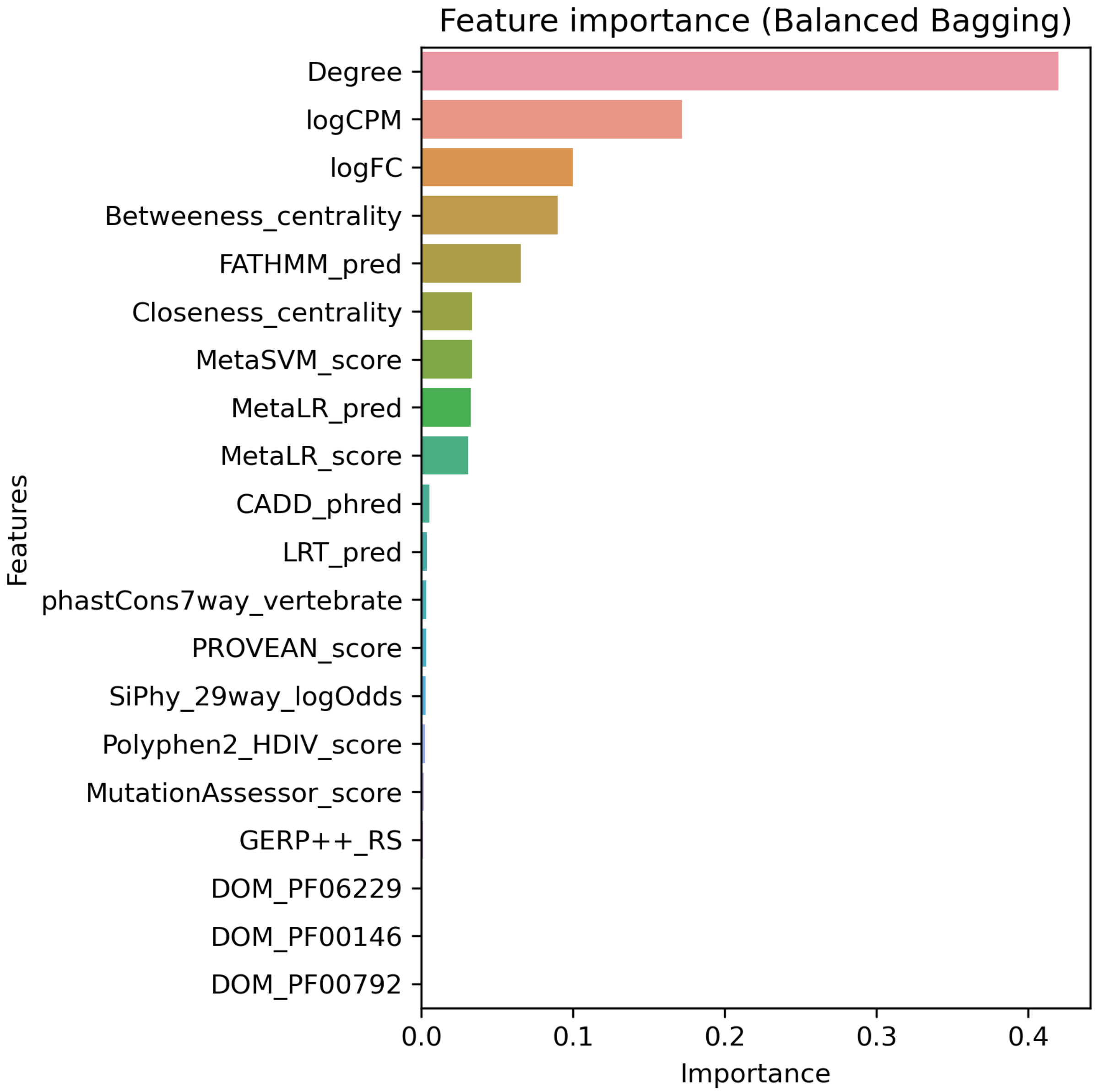

Figure S33A. Top 20 features for BRCA using multi-omic data with Marellotto *et al.* labels built on "all" features using Balanced Bagging.

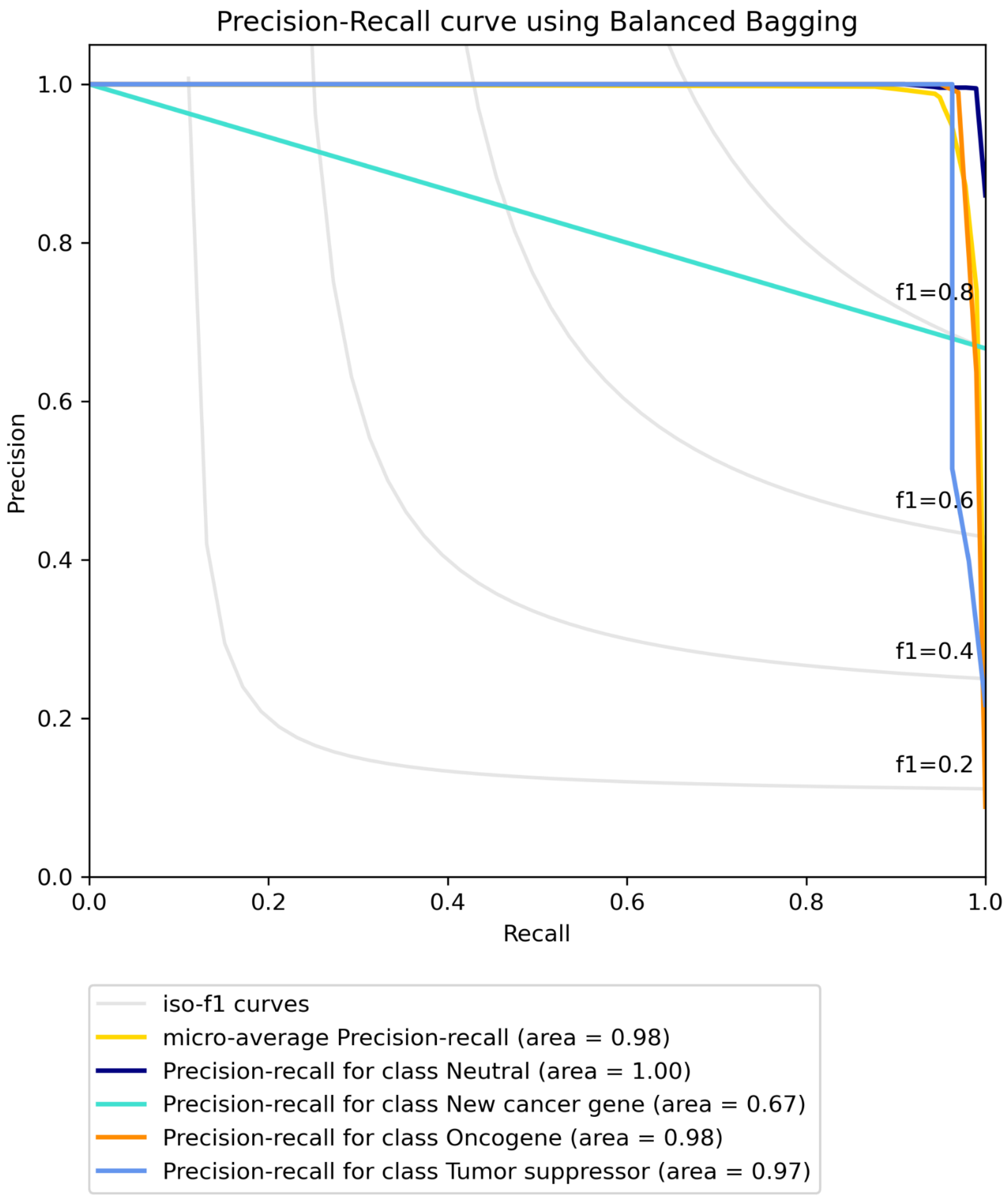

Figure S33B. Precision-recall curve for BRCA using multi-omic data with Marellotto *et al.* labels built on "all" features using Balanced Bagging.

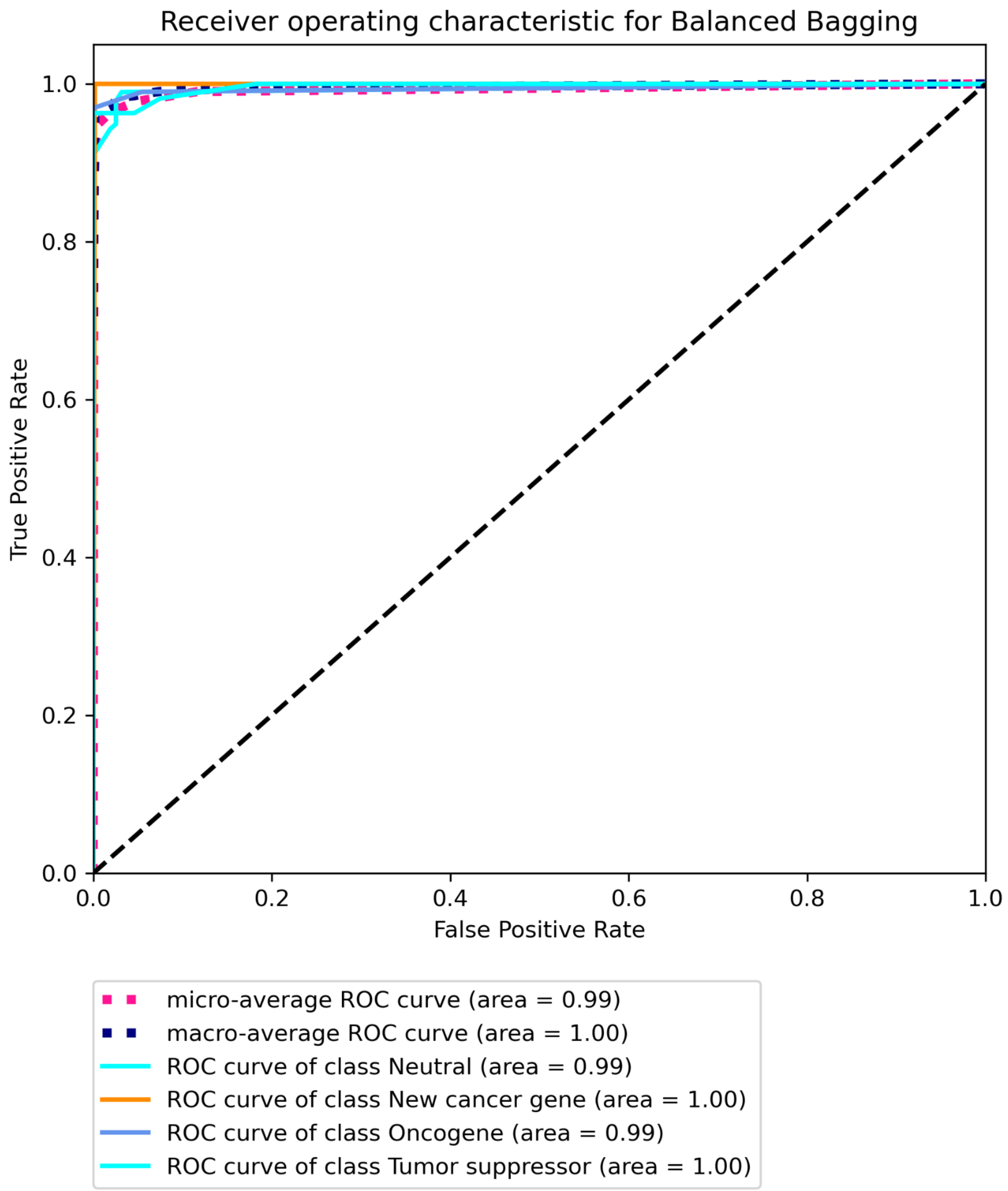

Figure S33C. Receiver operating characteristic for BRCA using multi-omic data with Marellotto *et al.* labels built on "all" features using Balanced Bagging.

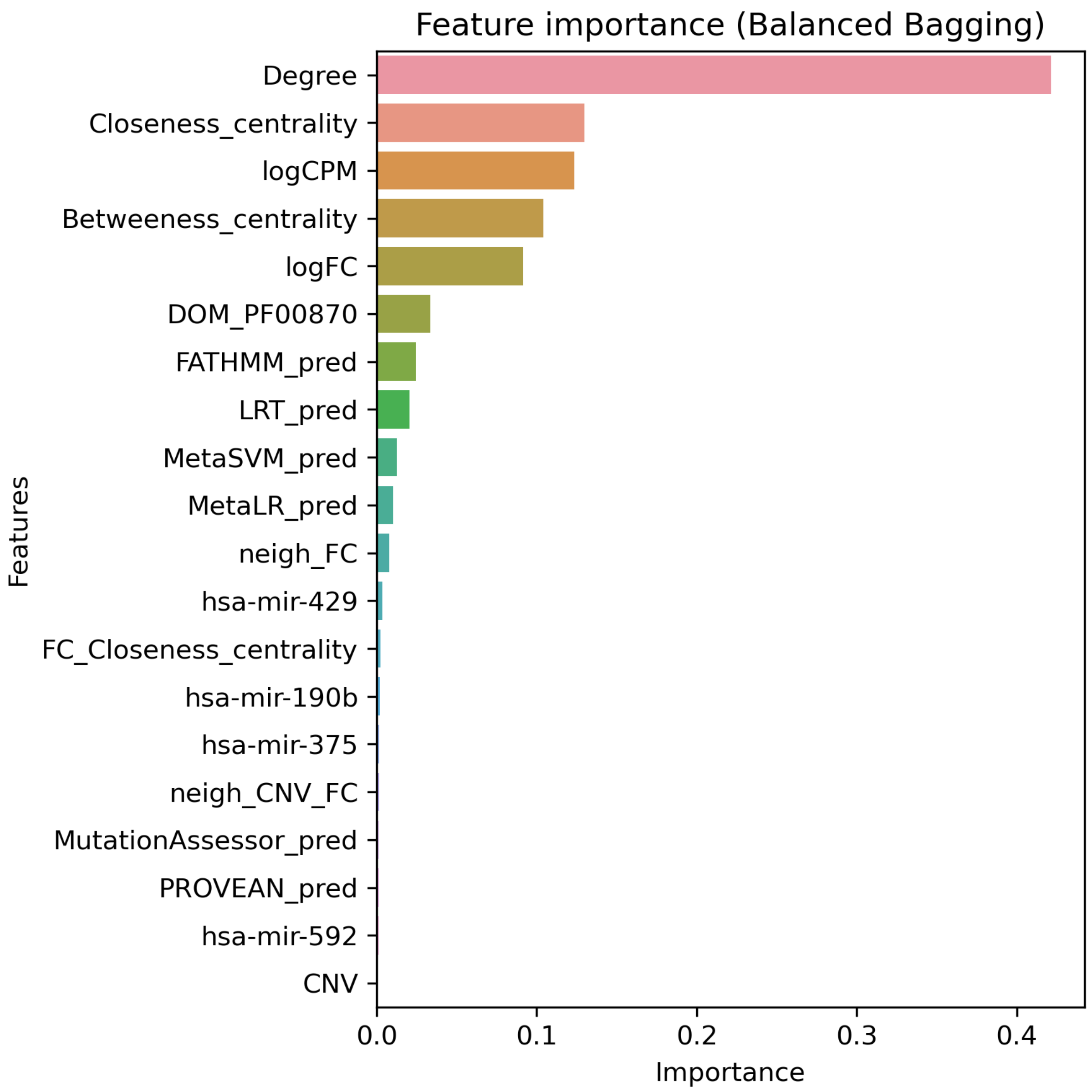

Figure S34A. Top 20 features for BRCA using multi-omic data with Marellotto *et al.* labels built on "some" features using Balanced Bagging.

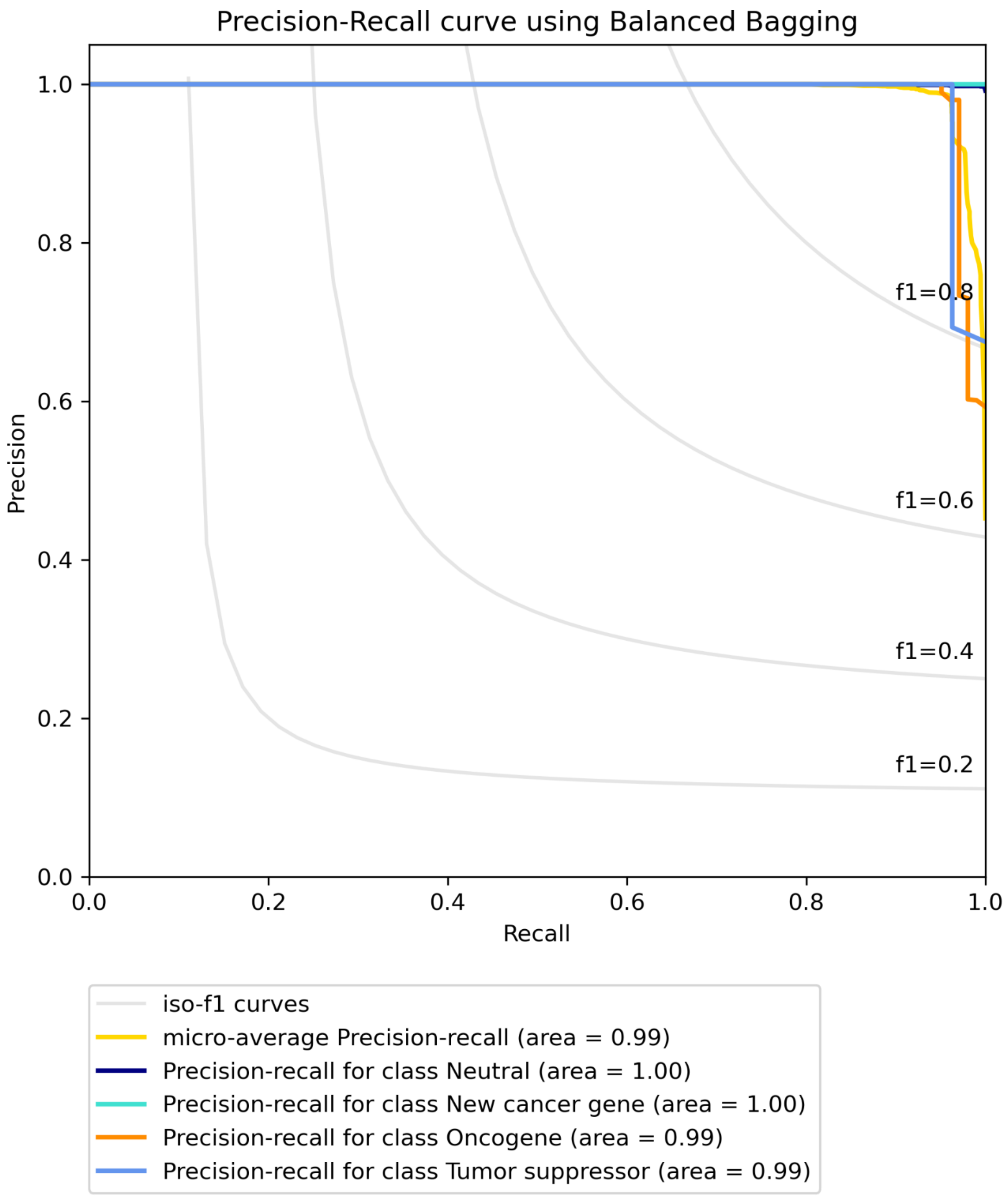

Figure S34B. Precision-recall curve for BRCA using multi-omic data with Marellotto *et al.* labels built on "some" features using Balanced Bagging.

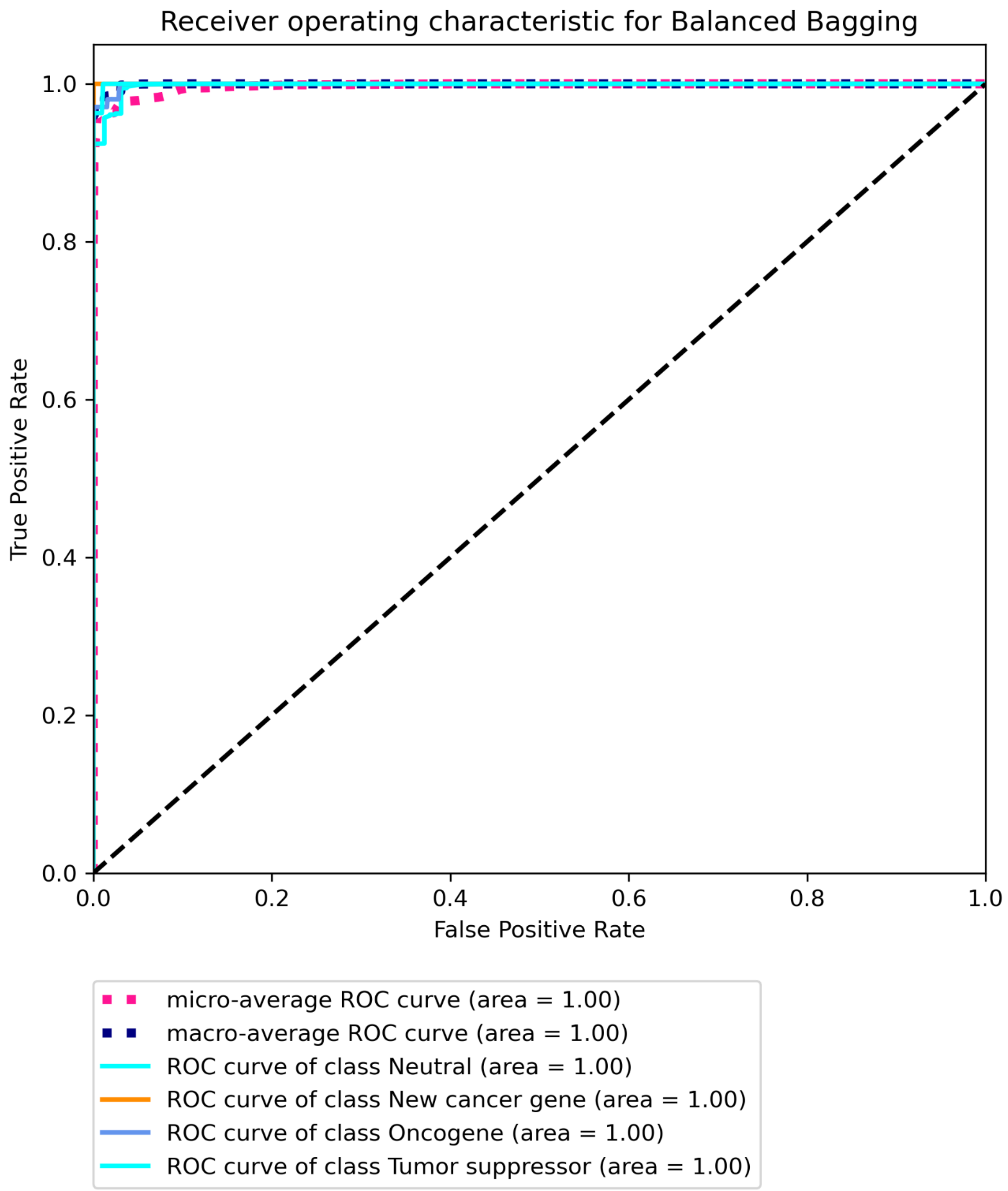

Figure S34C. Receiver operating characteristic for BRCA using multi-omic data with Marellotto *et al.* labels built on "some" features using Balanced Bagging.

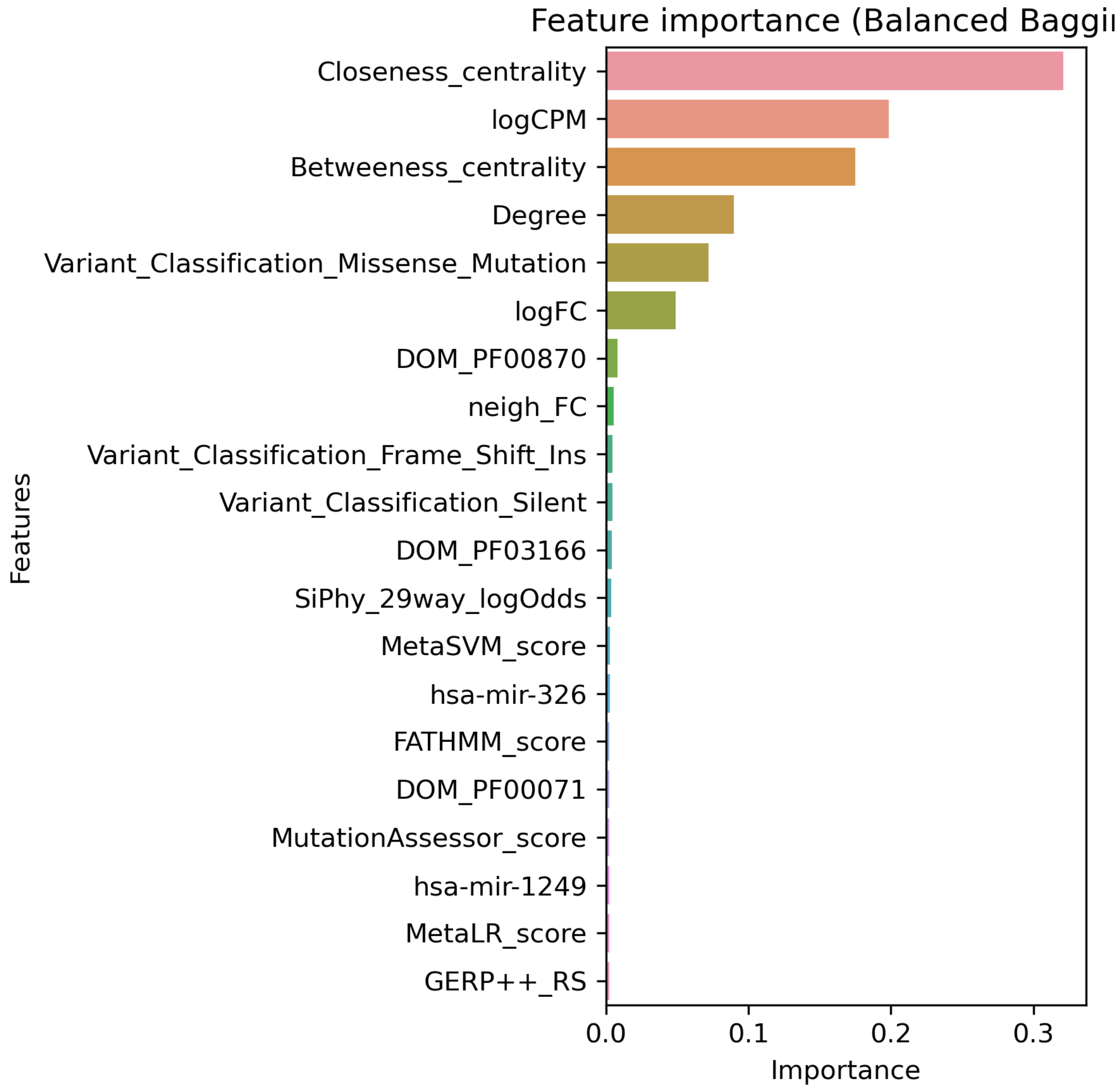

Figure S35A. Top 20 features for COAD using multi-omic data with Bailey *et al.* labels built on "all" features using Balanced bagging.

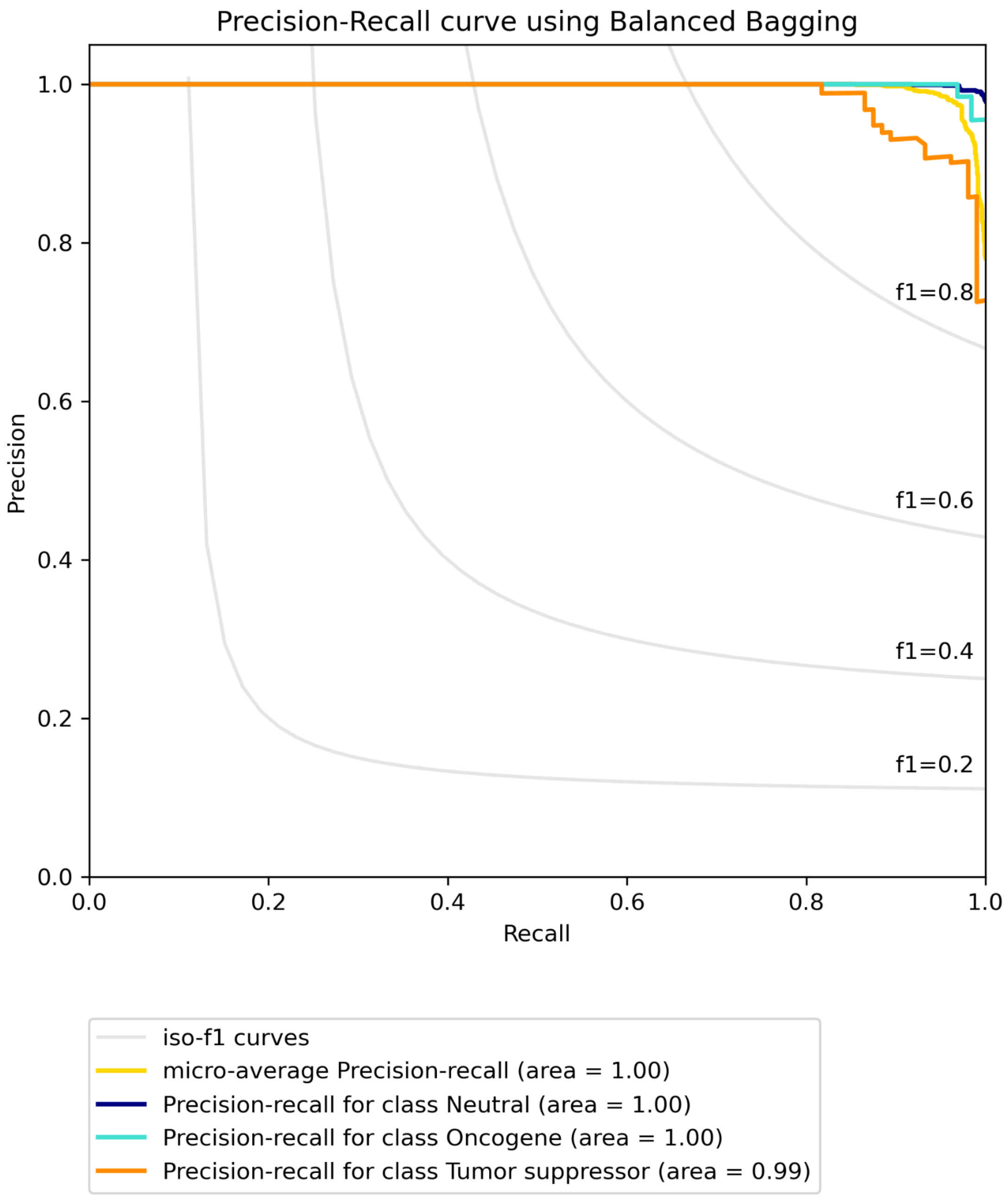

Figure S35B. Precision-recall curve for COAD using multi-omic data with Bailey *et al.* labels built on “all” features using Balanced bagging.

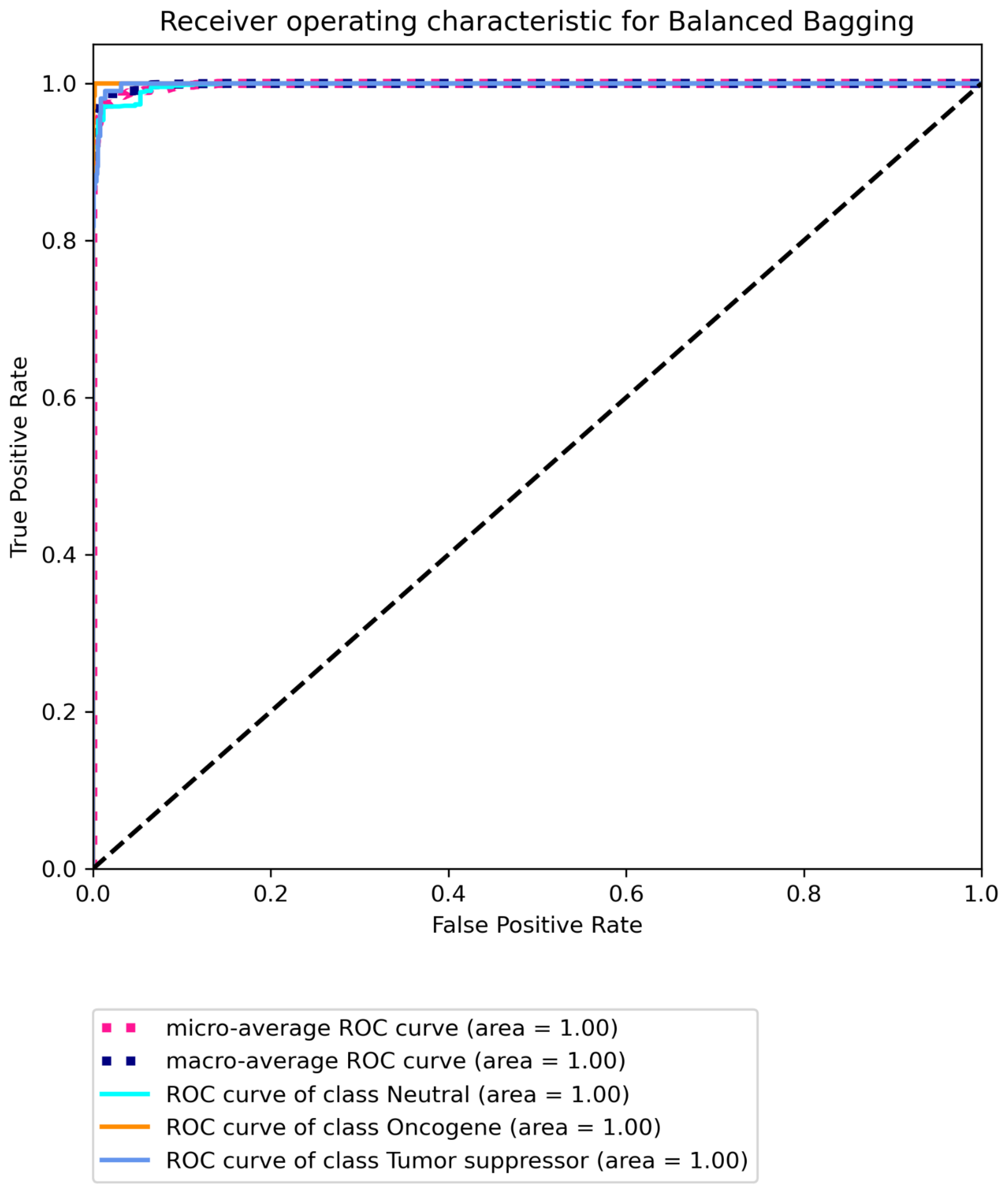

Figure S35C Receiver operating characteristic for COAD using multi-omic data with Bailey *et al.* labels built on "all" features using Balanced bagging.

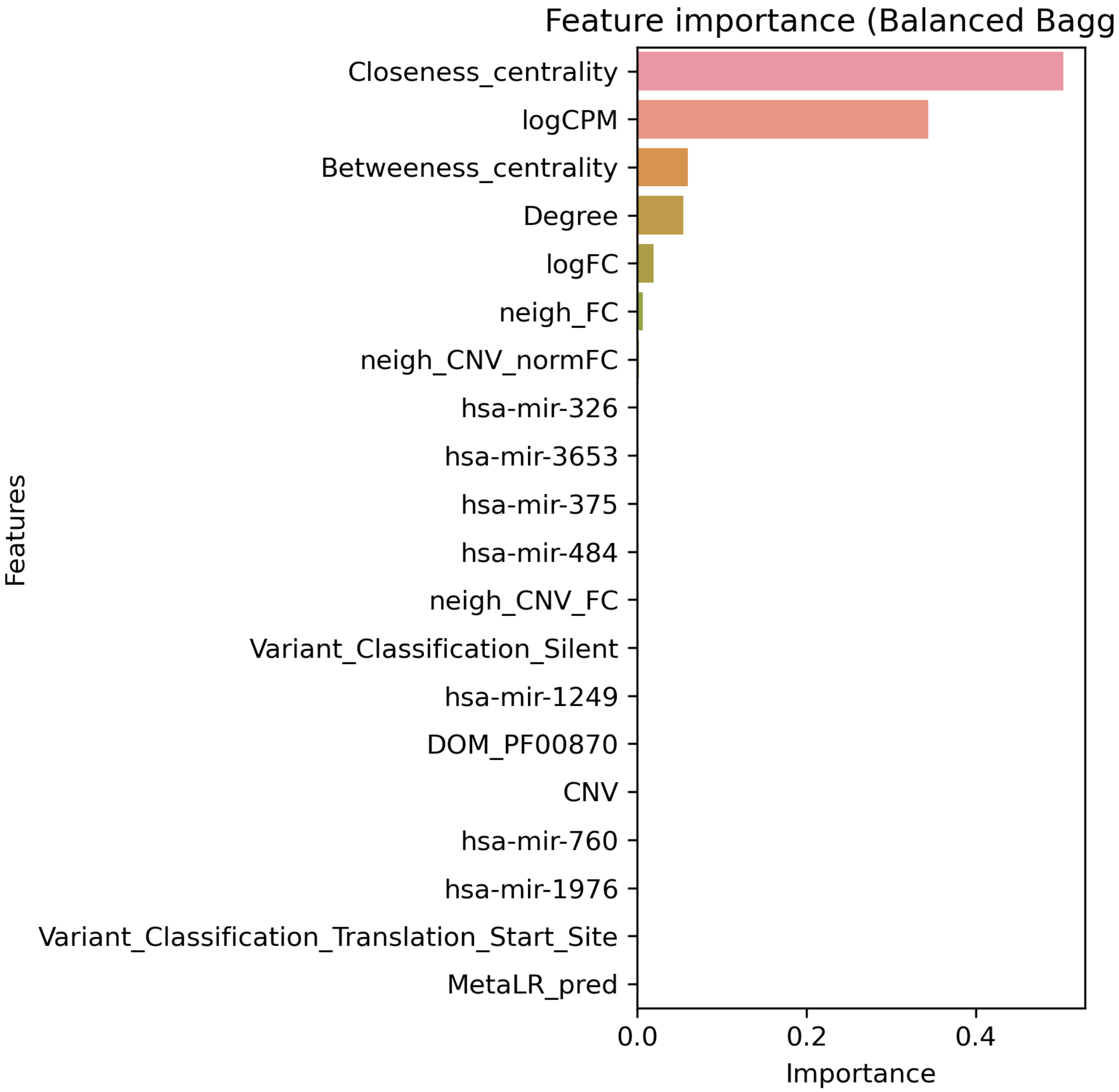

Figure S36A. Top 20 features for COAD using multi-omic data with Bailey *et al.* labels built on "some" features using Balanced bagging.

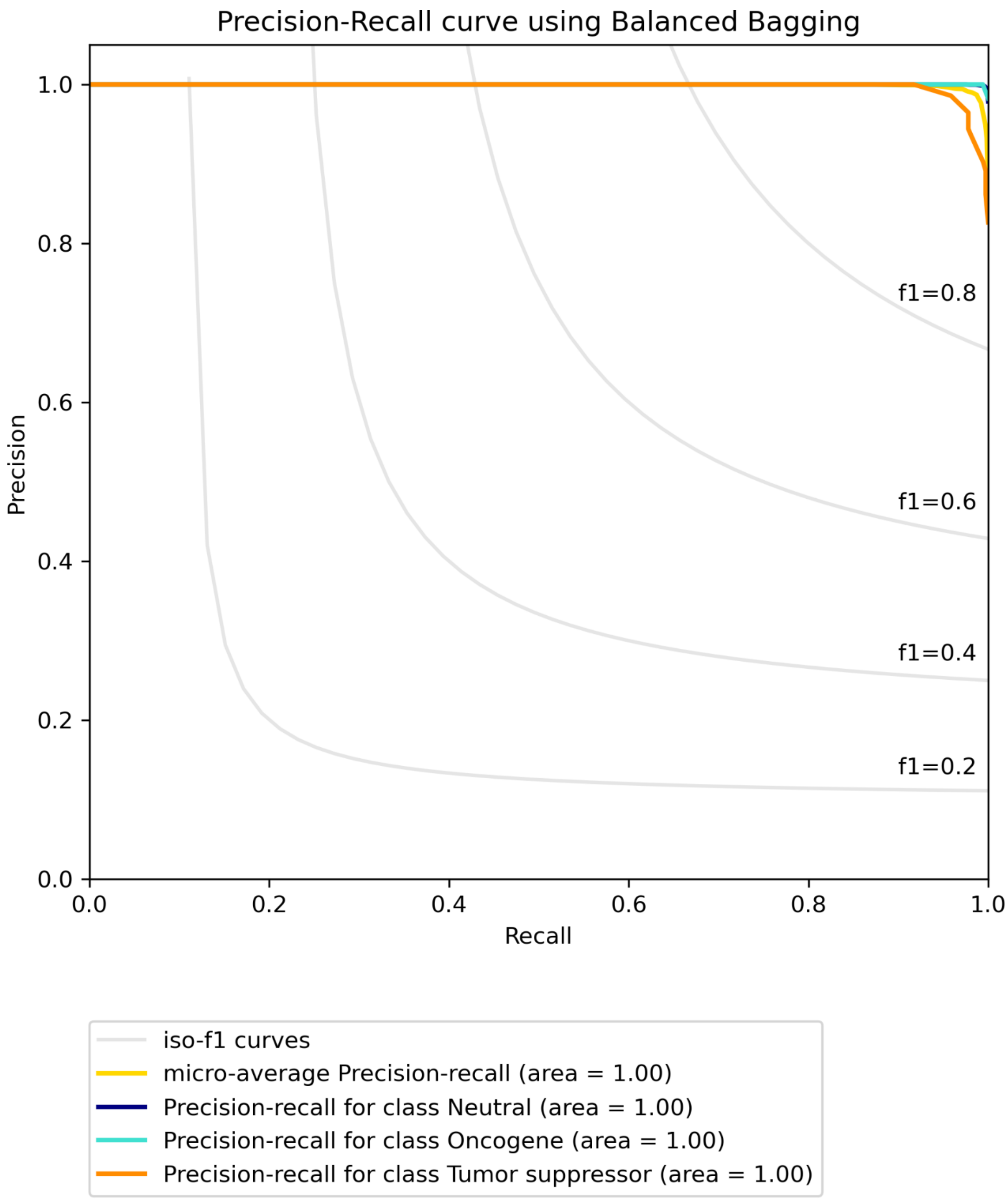

Figure S36B. Precision-recall curve for COAD using multi-omic data with Bailey *et al.* labels built on "some" features using Balanced bagging.

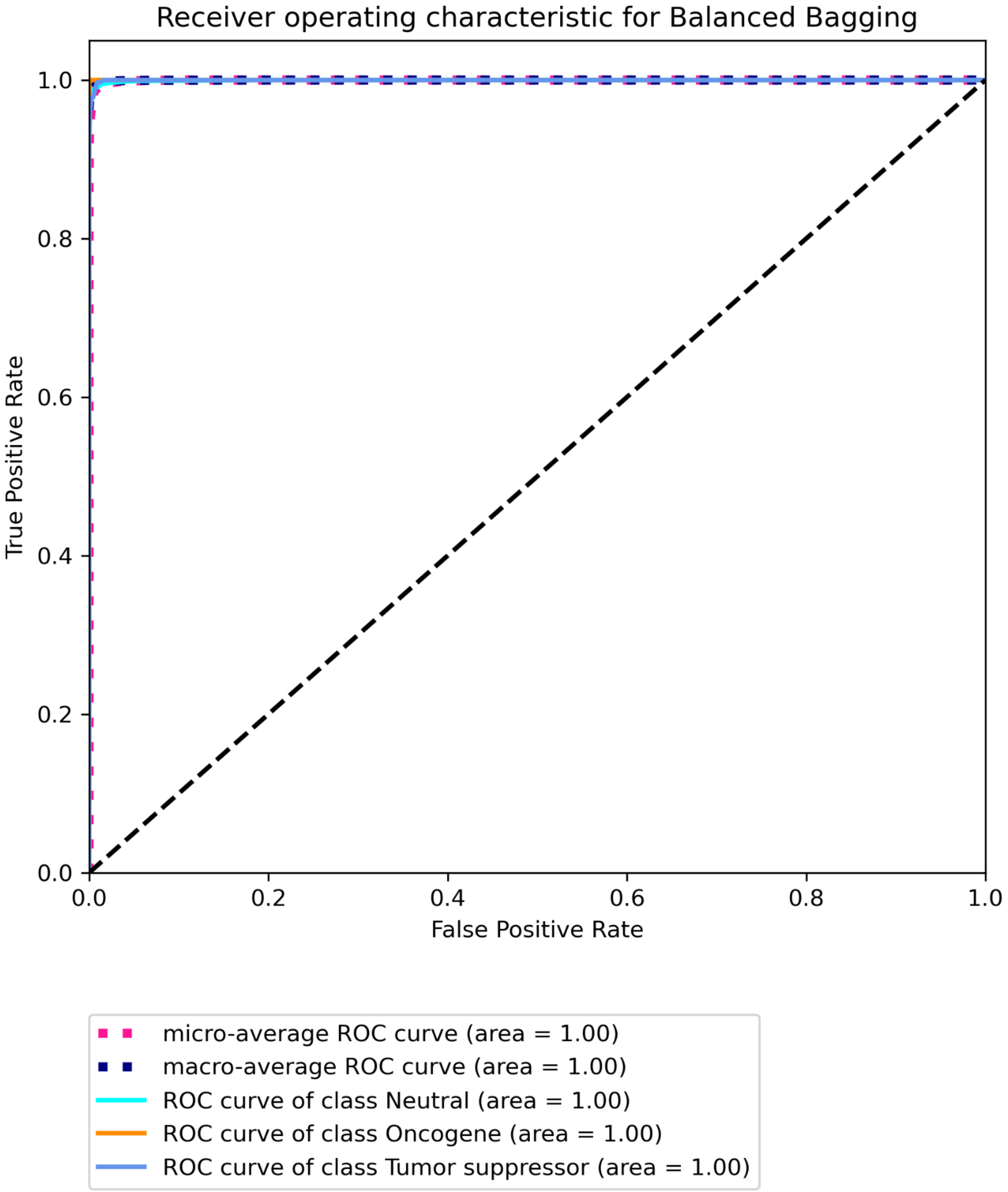

Figure S36C Receiver operating characteristic for COAD using multi-omic data with Bailey *et al.* labels built on “some” features using Balanced bagging.

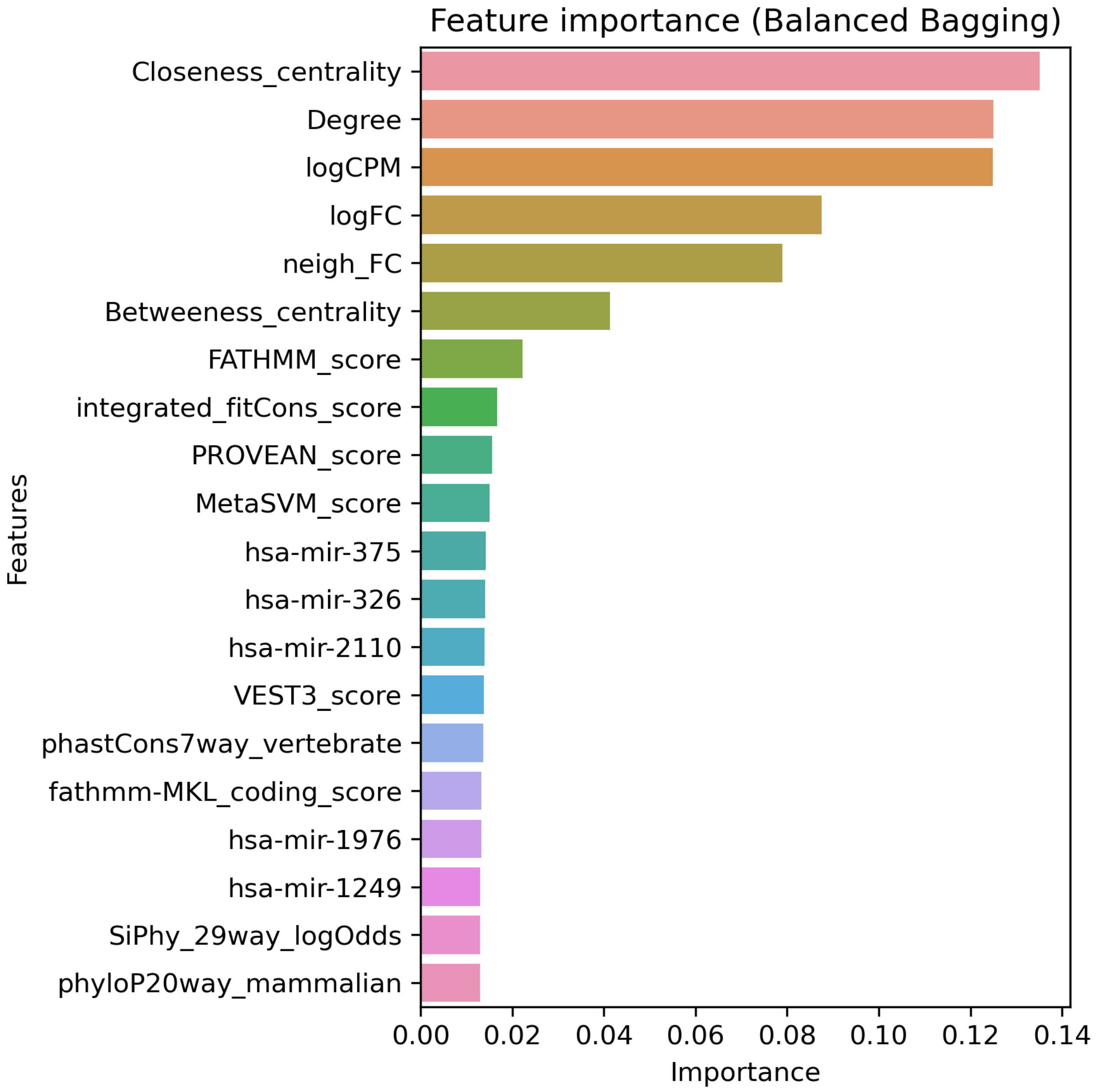

Figure S37A. Top 20 features for COAD using multi-omic data with CGC labels built on "all" features using Balanced bagging.

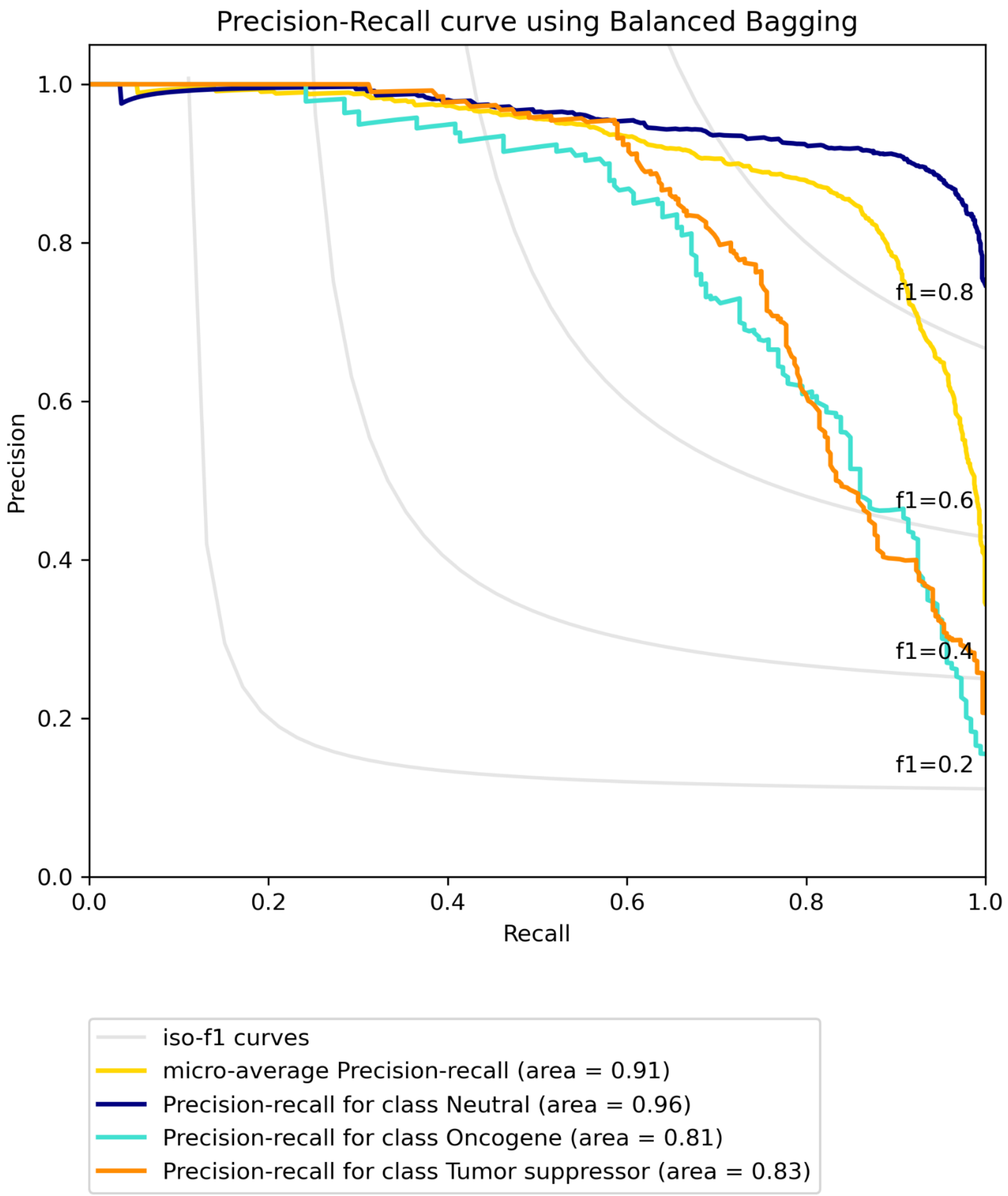

Figure S37B. Precision-recall curve for COAD using multi-omic data with CGC labels built on "all" features using Balanced bagging.

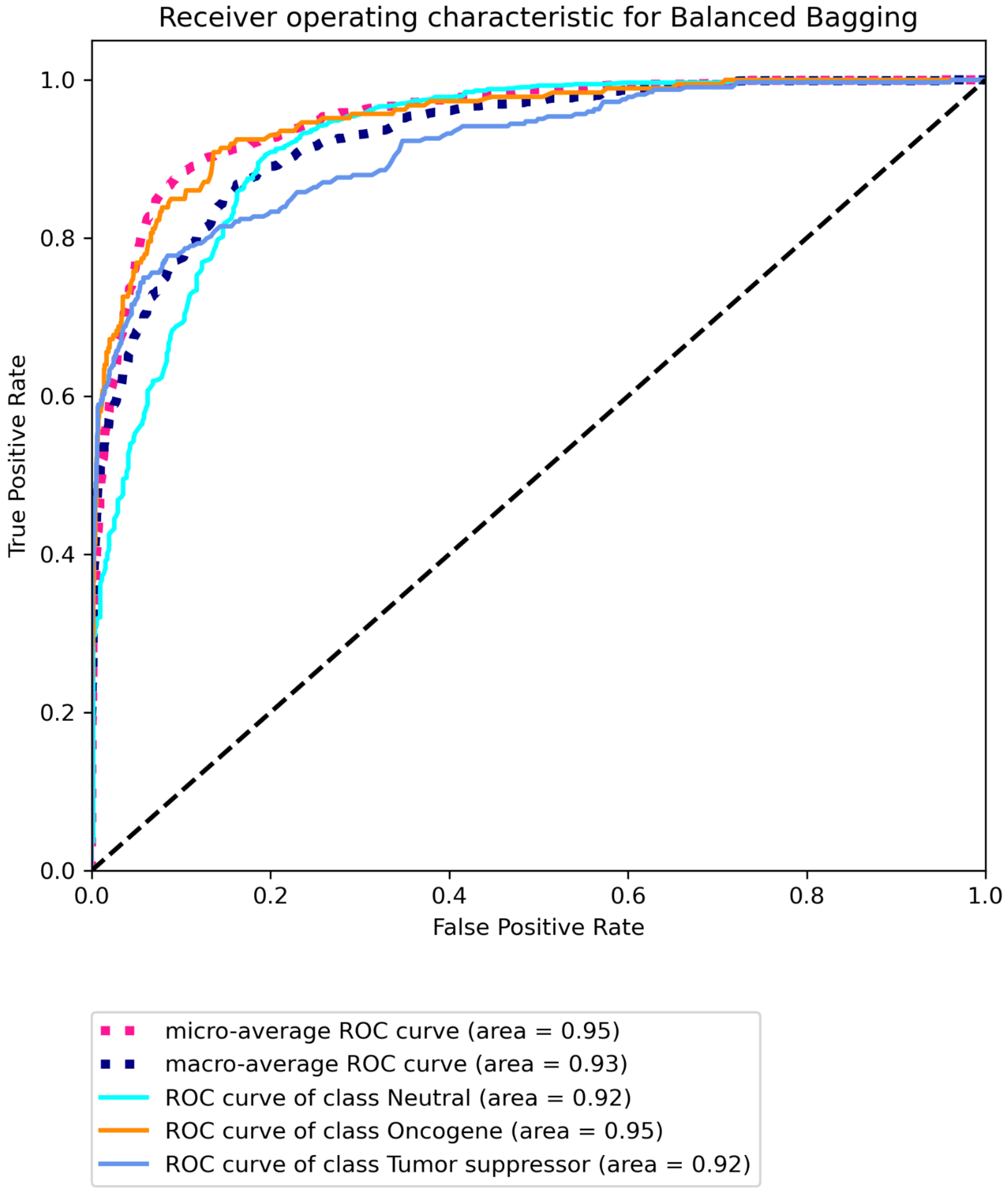

Figure S37C. Receiver operating characteristic for COAD using multi-omic data with CGC labels built on "all" features using Balanced Bagging.

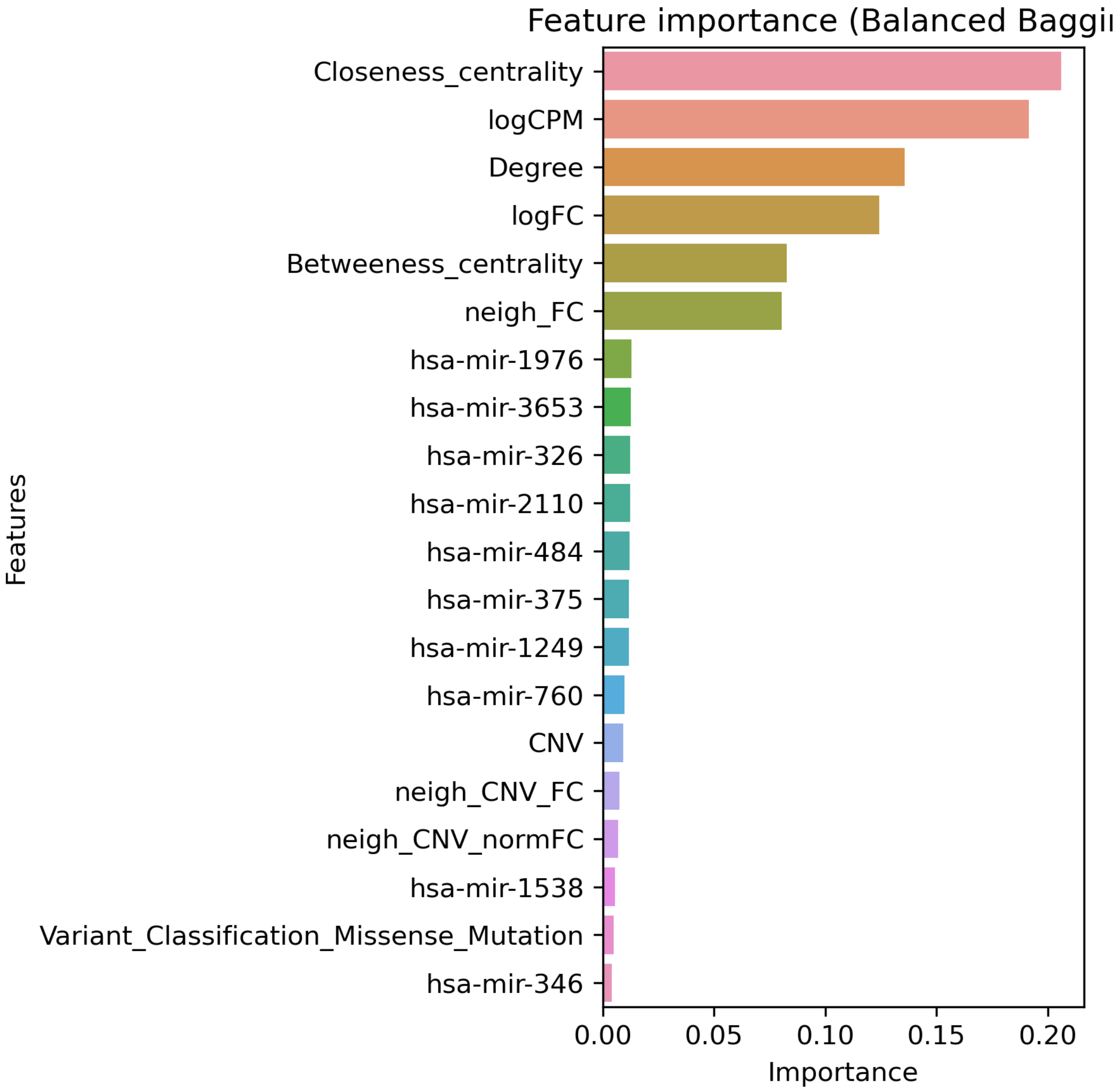

Figure S38A. Top 20 features for COAD using multi-omic data with CGC labels built on "some" features using Balanced Bagging.

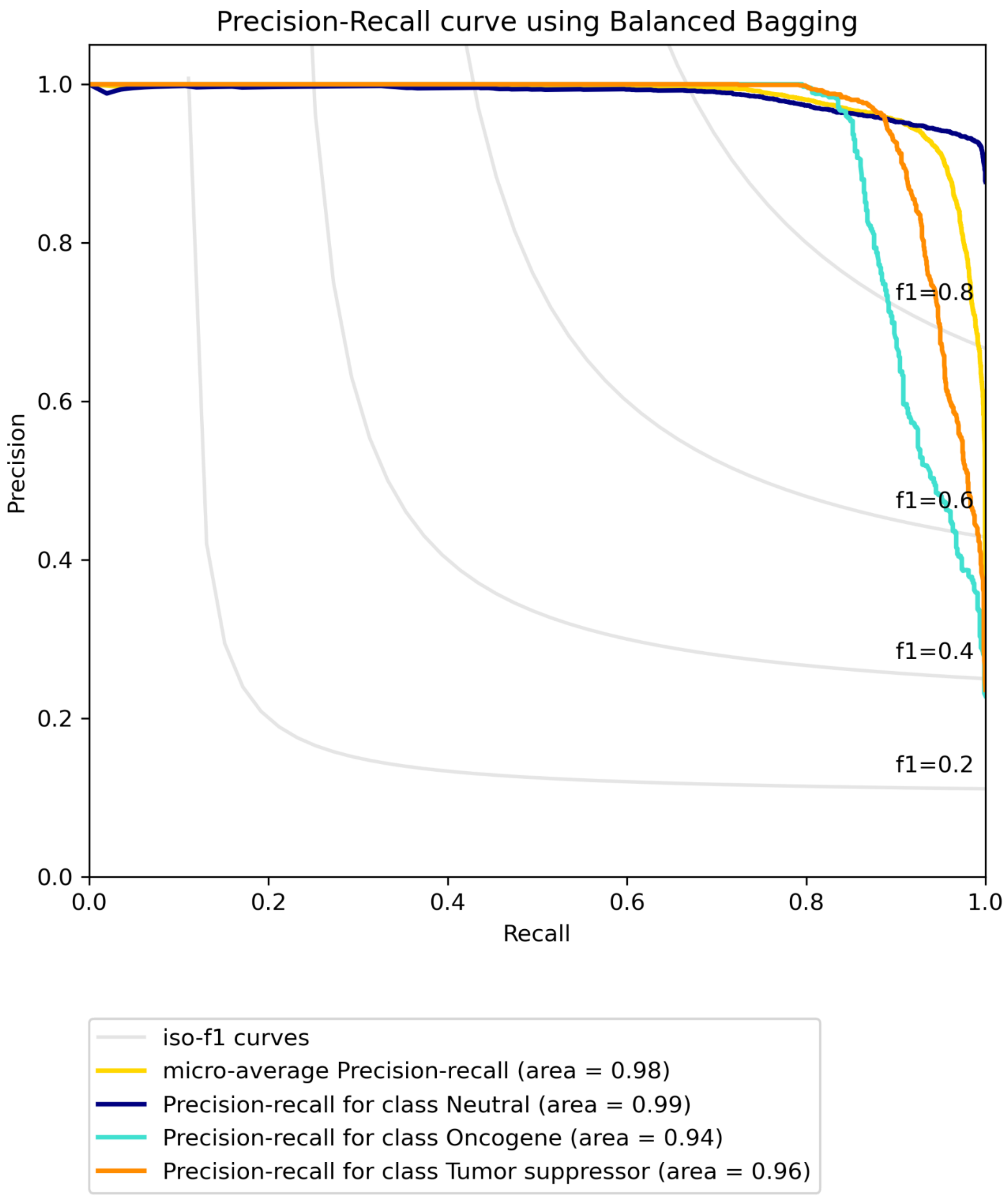

Figure S38B. Precision-recall curve for COAD using multi-omic data with CGC labels built on "some" features using Balanced Bagging.

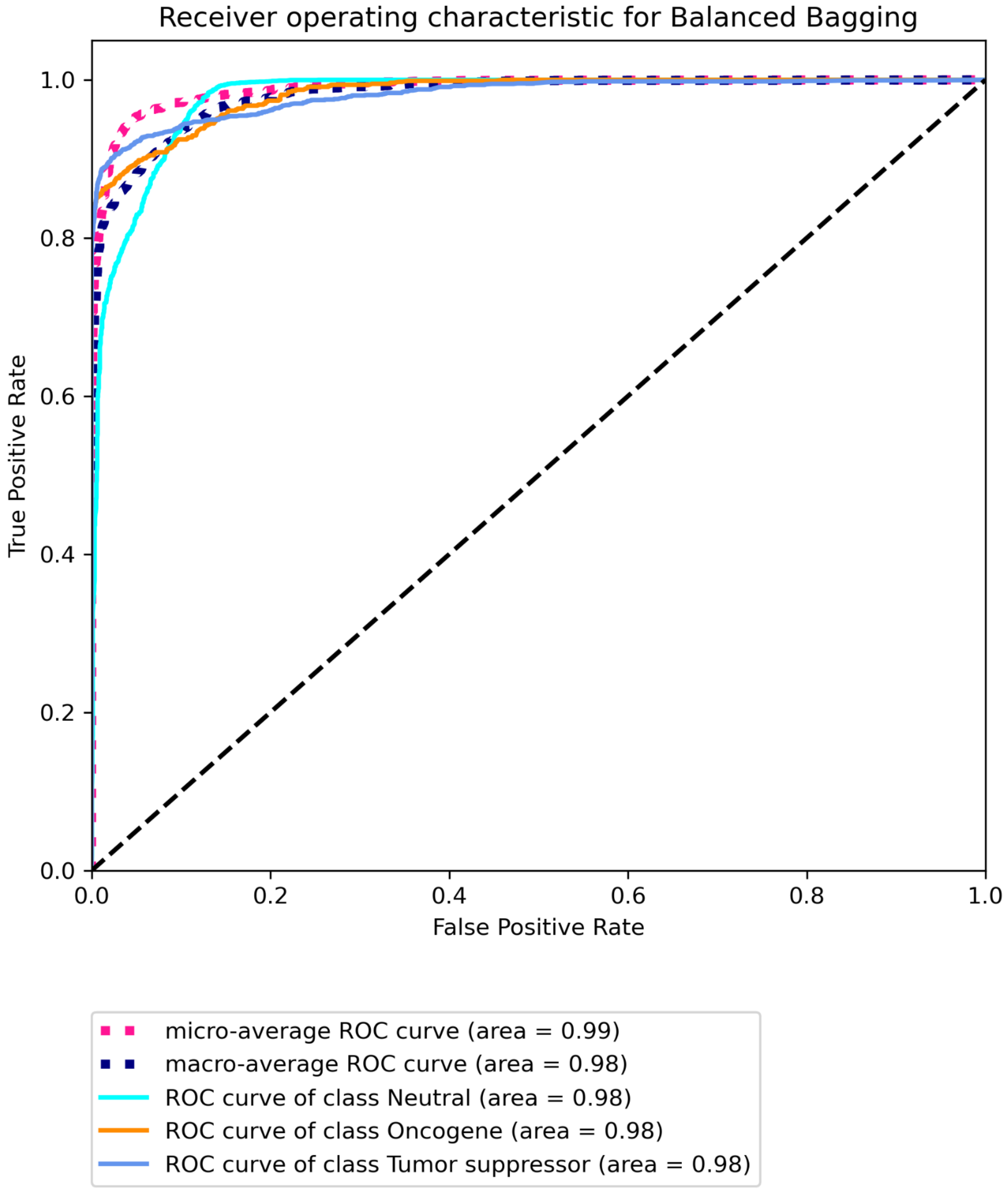

Figure S38C. Receiver operating characteristic for COAD using multi-omic data with CGC labels built on “some” features using Balanced Bagging.

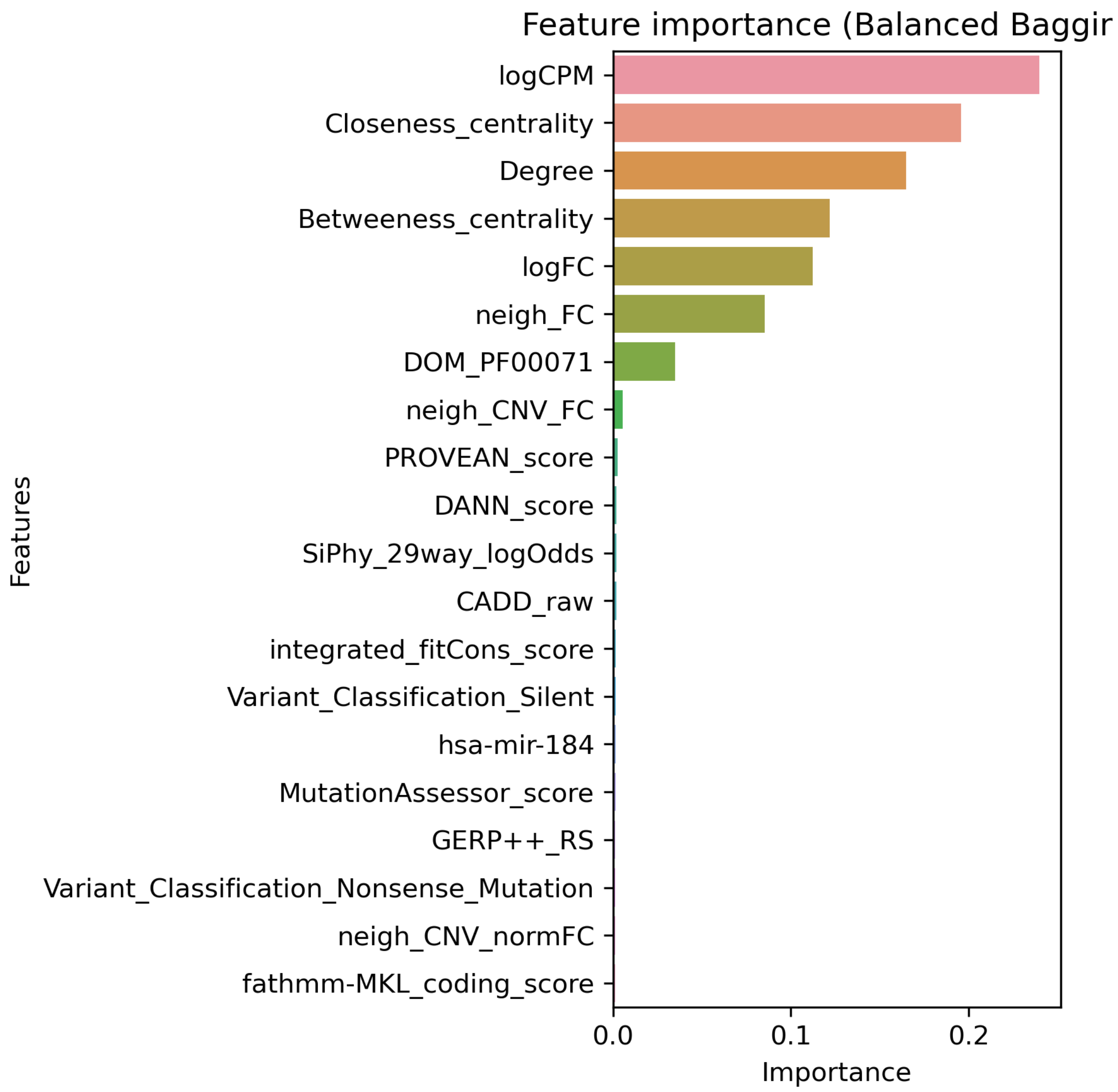

Figure S39A. Top 20 features for LUAD using multi-omic data with Bailey *et al.* labels built on "all" features using Balanced bagging.

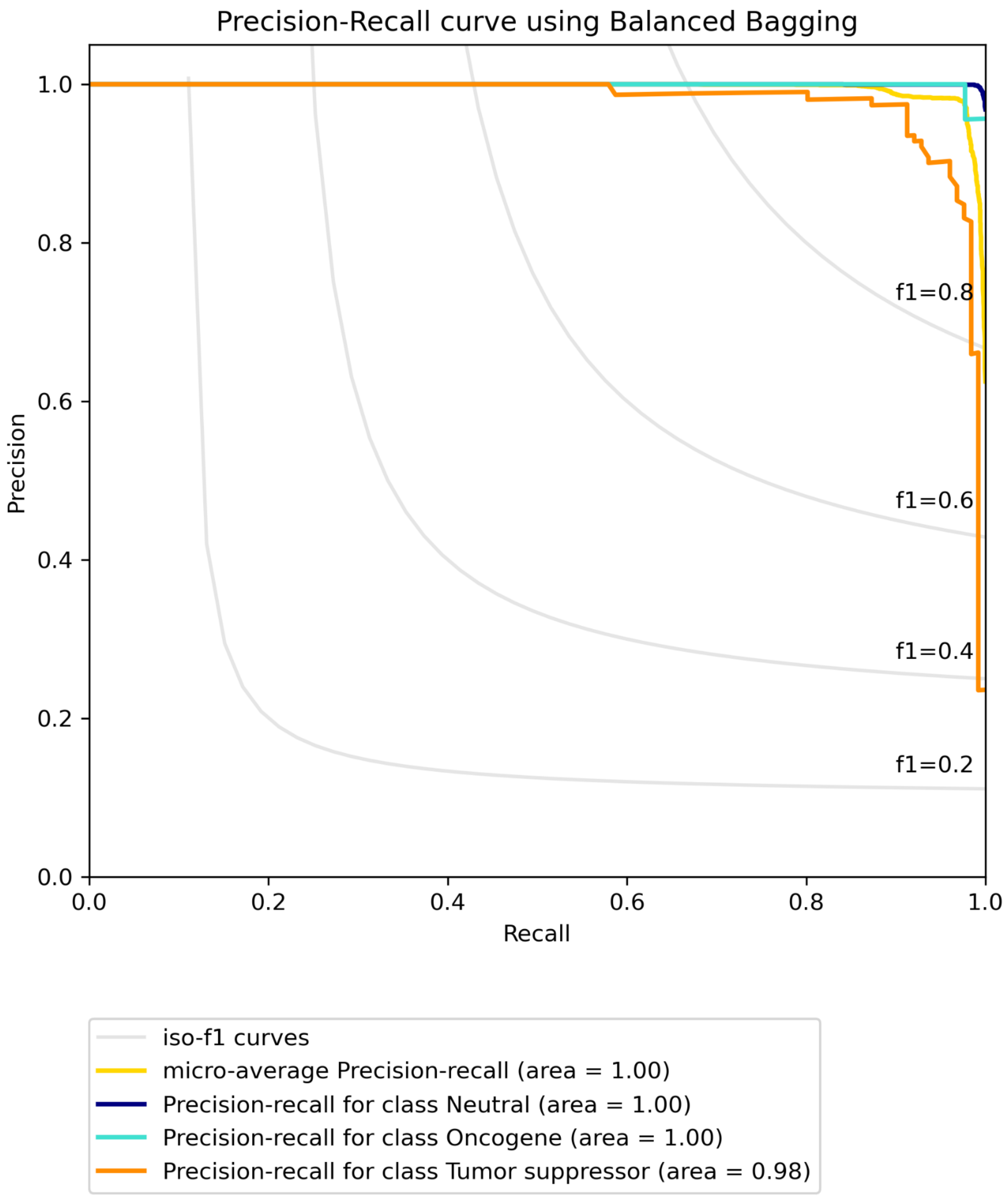

Figure S39B. Precision-recall curve for LUAD using multi-omic data with Bailey *et al.* labels built on "all" features using Balanced bagging.

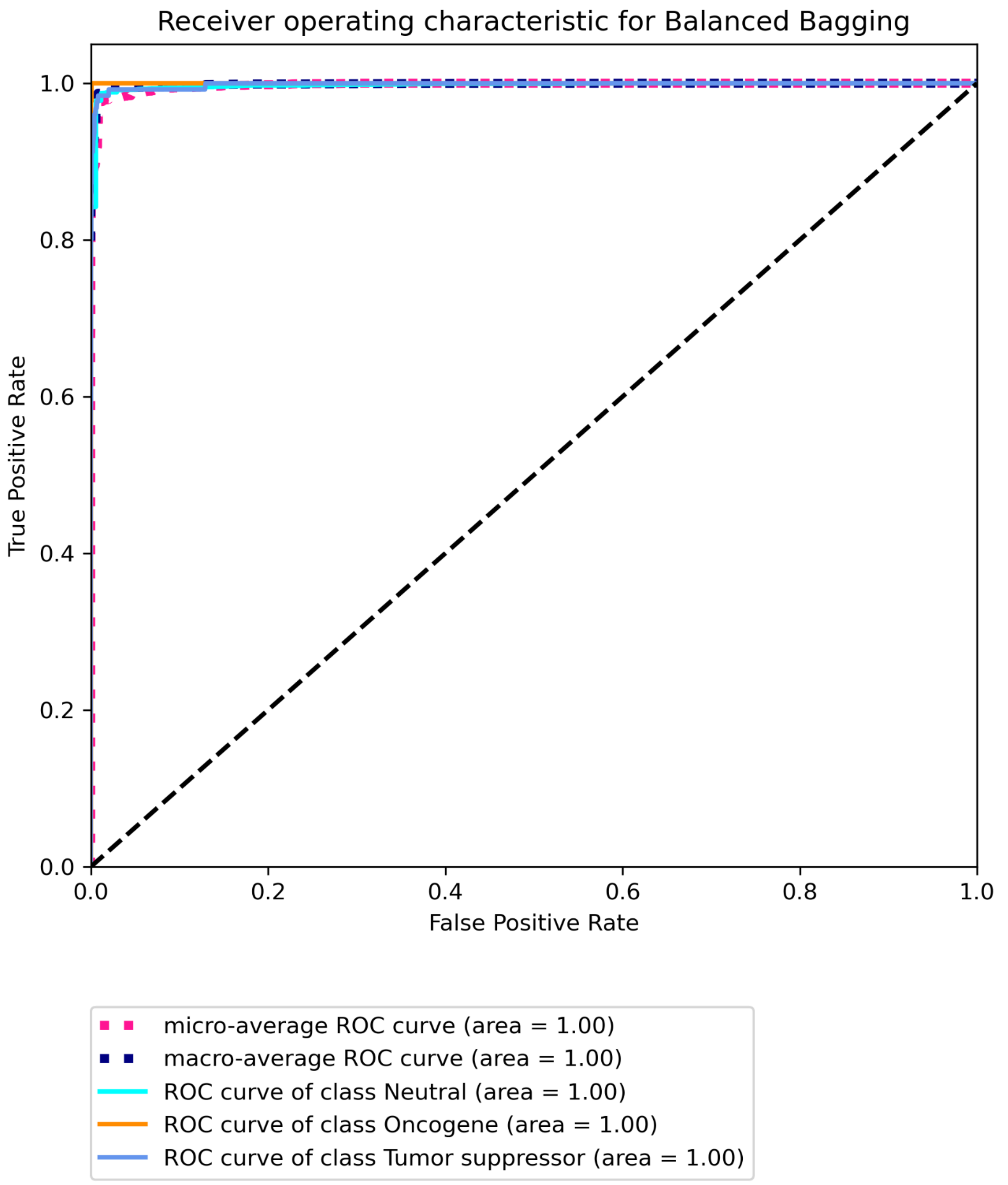

Figure S39C Receiver operating characteristic for LUAD using multi-omic data with Bailey *et al.* labels built on "all" features using Balanced bagging.

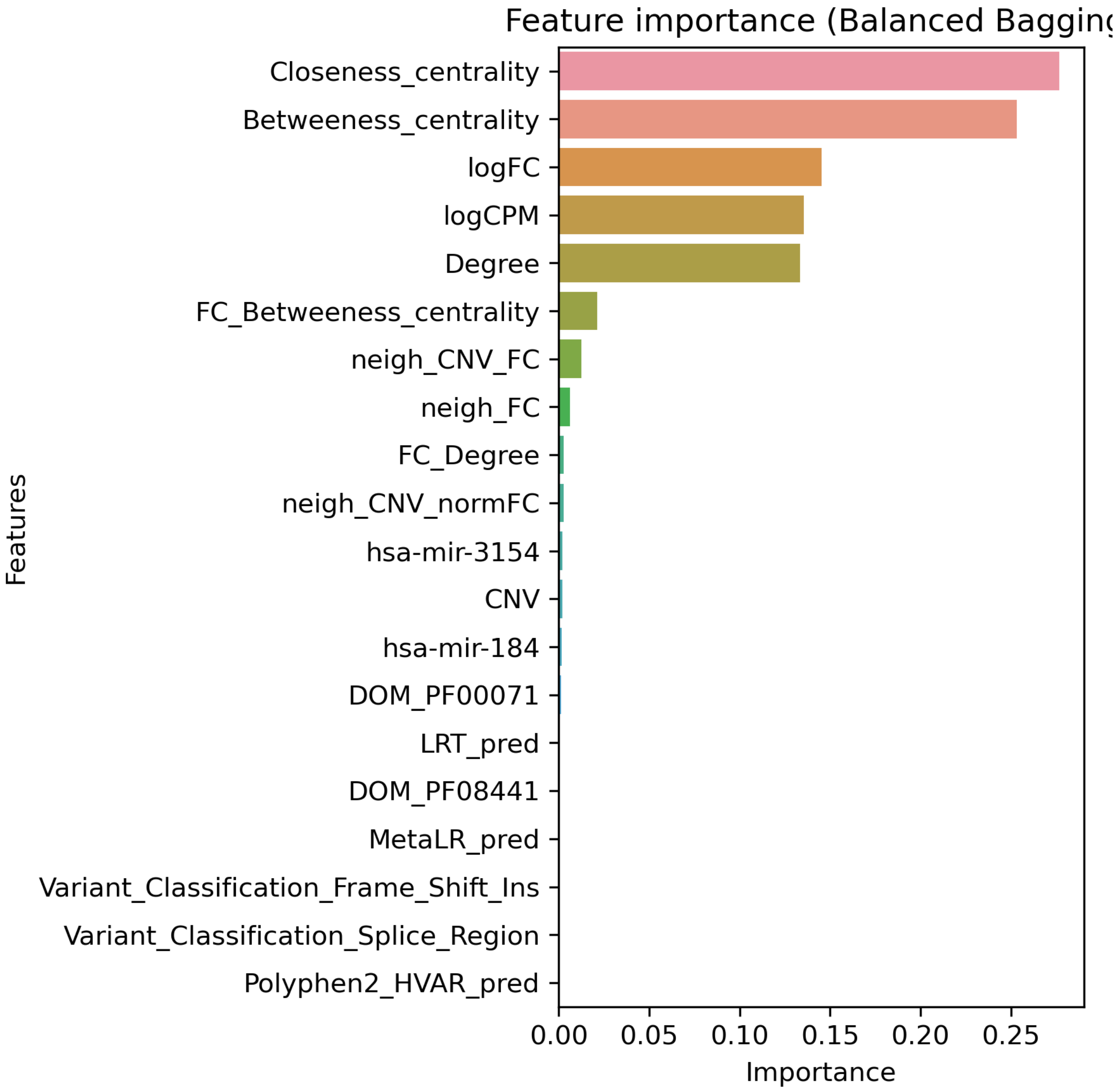

Figure S40A. Top 20 features for LUAD using multi-omic data with Bailey *et al.* labels built on “some” features using Balanced bagging.

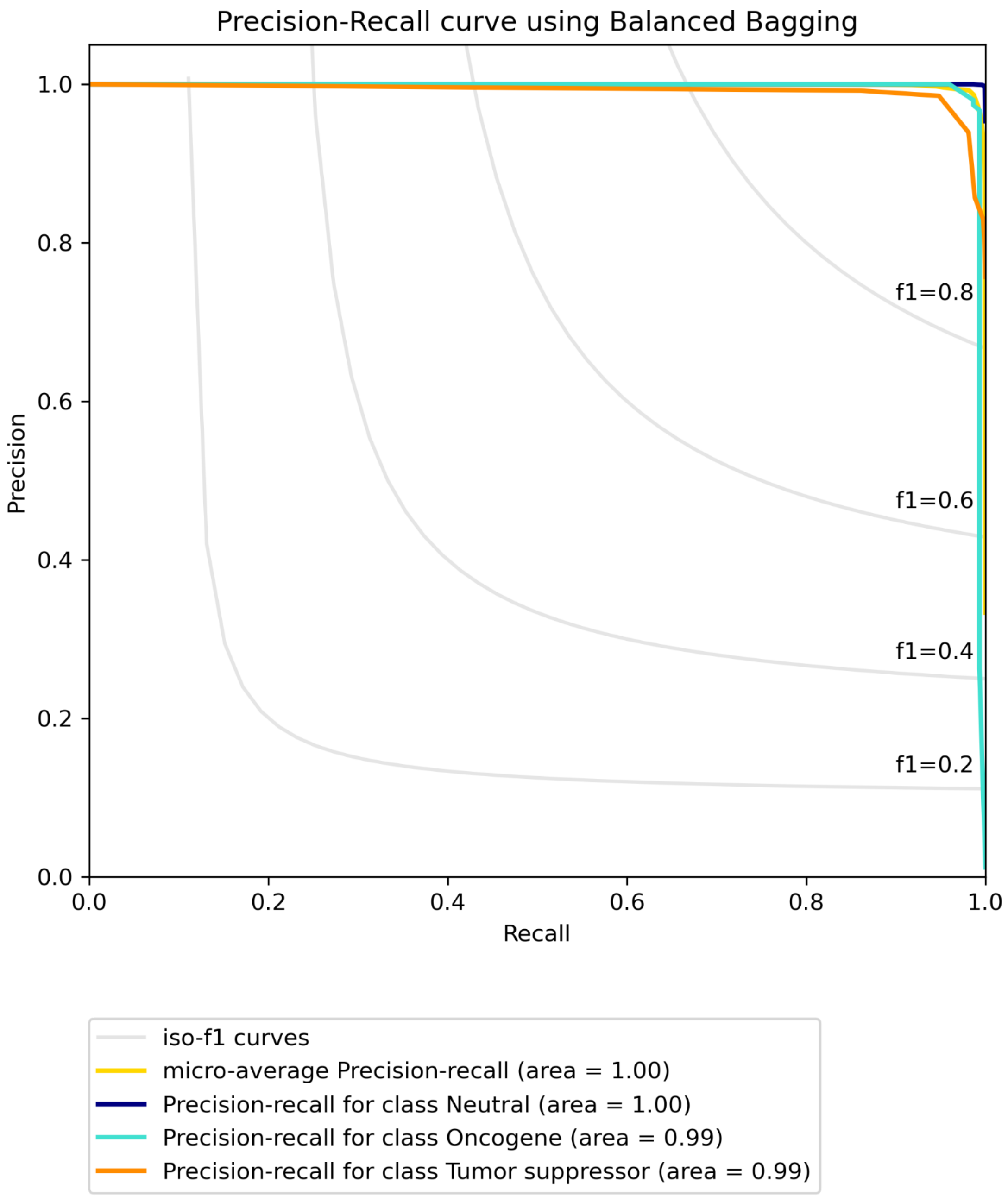

Figure S40B. Precision-recall curve for LUAD using multi-omic data with Bailey *et al.* labels built on "some" features using Balanced bagging.

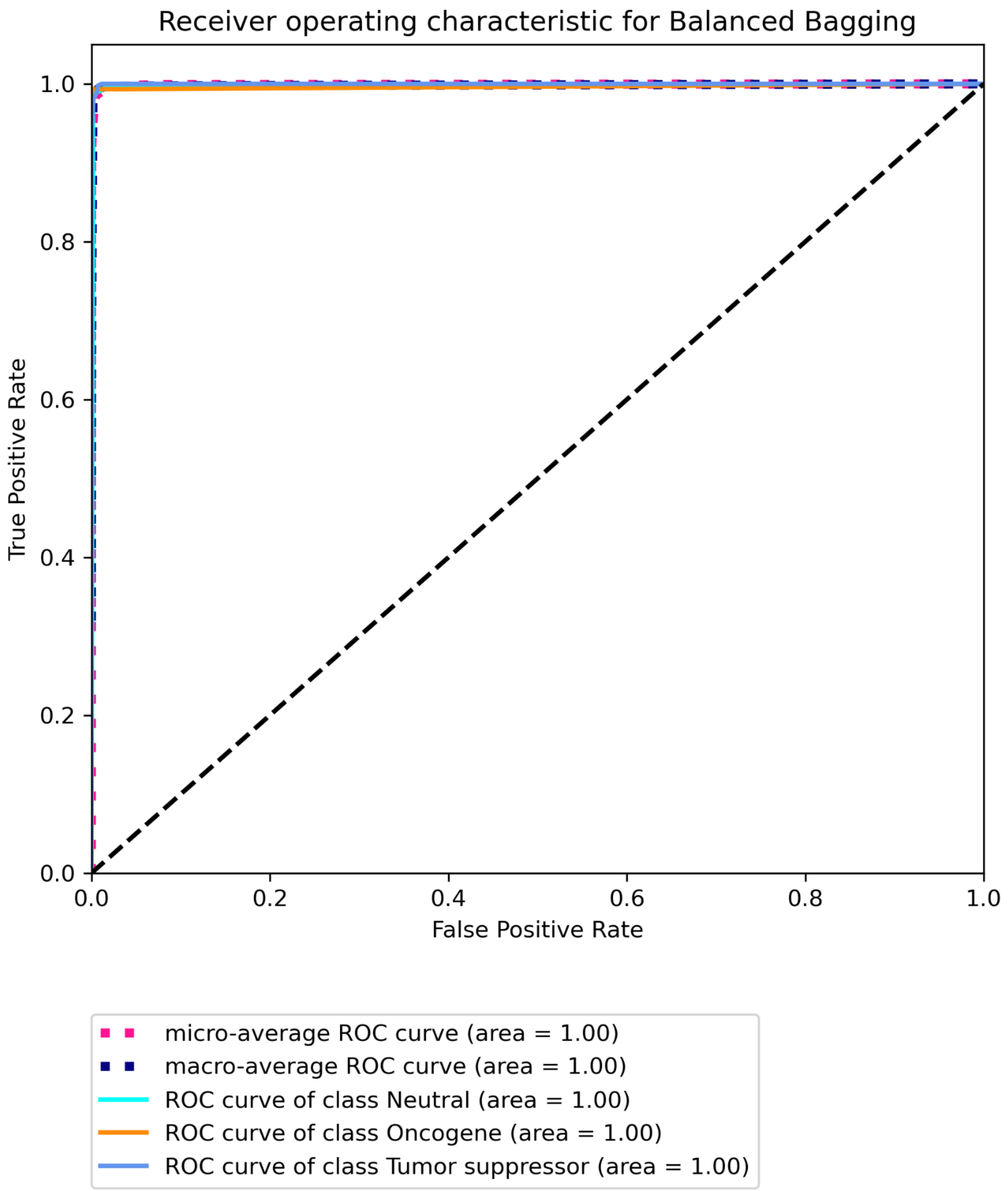

Figure S40C Receiver operating characteristic for LUAD using multi-omic data with Bailey *et al.* labels built on "some" features using Balanced bagging.

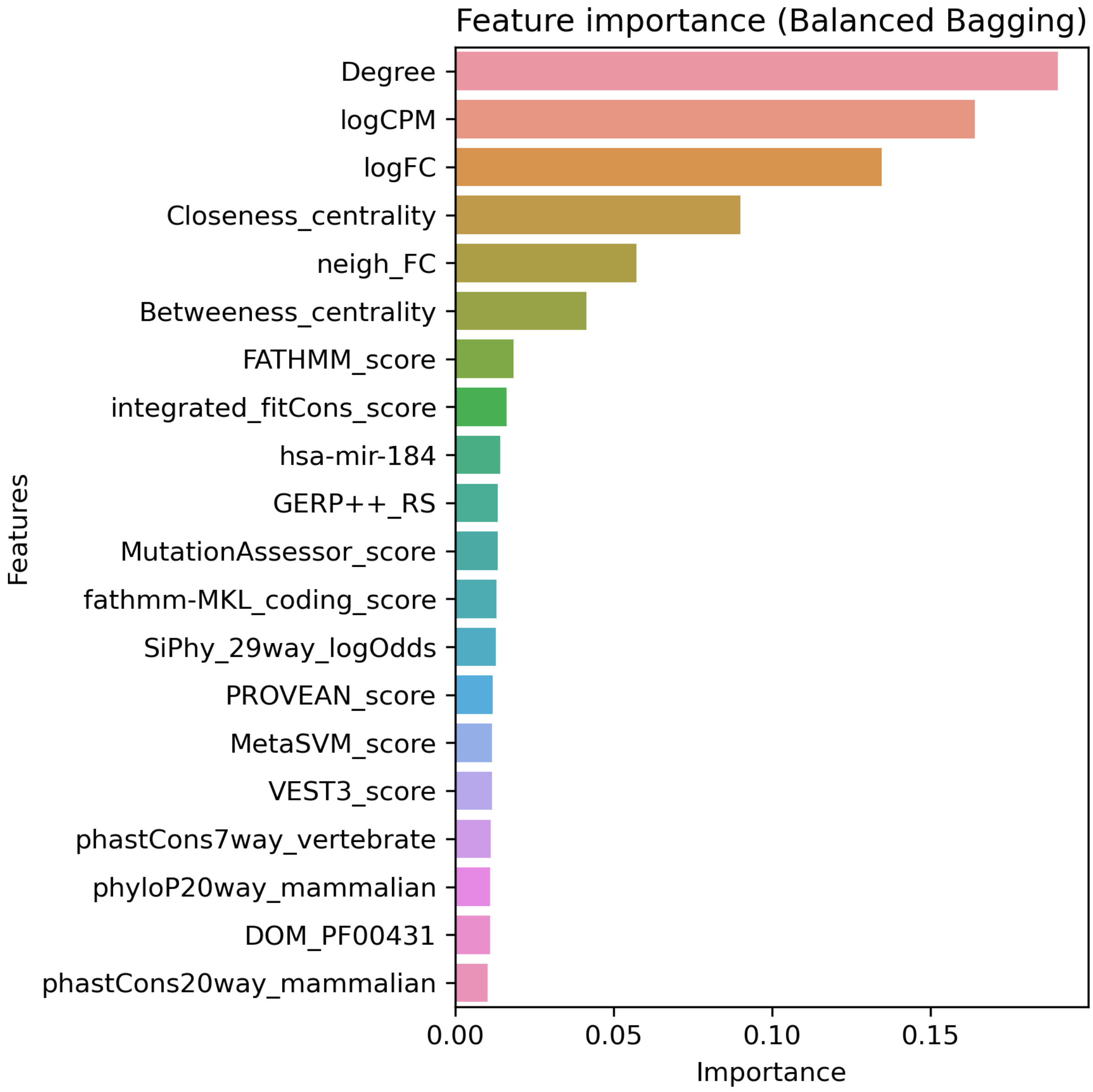

Figure S41A. Top 20 features for LUAD using multi-omic data with CGC labels built on "all" features using Balanced bagging.

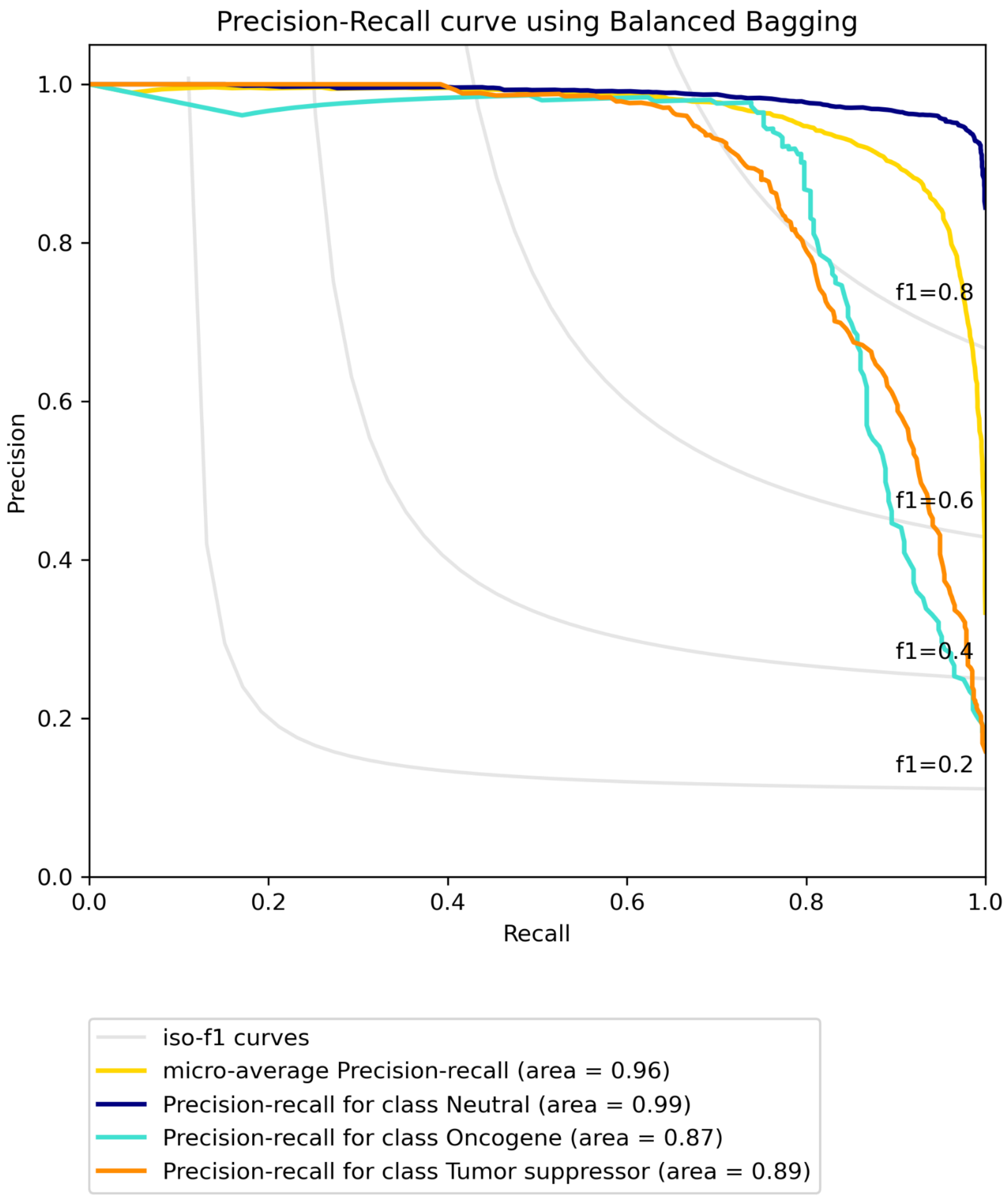

Figure S41B. Precision-recall curve for LUAD using multi-omic data with CGC labels built on “all” features using Balanced bagging.

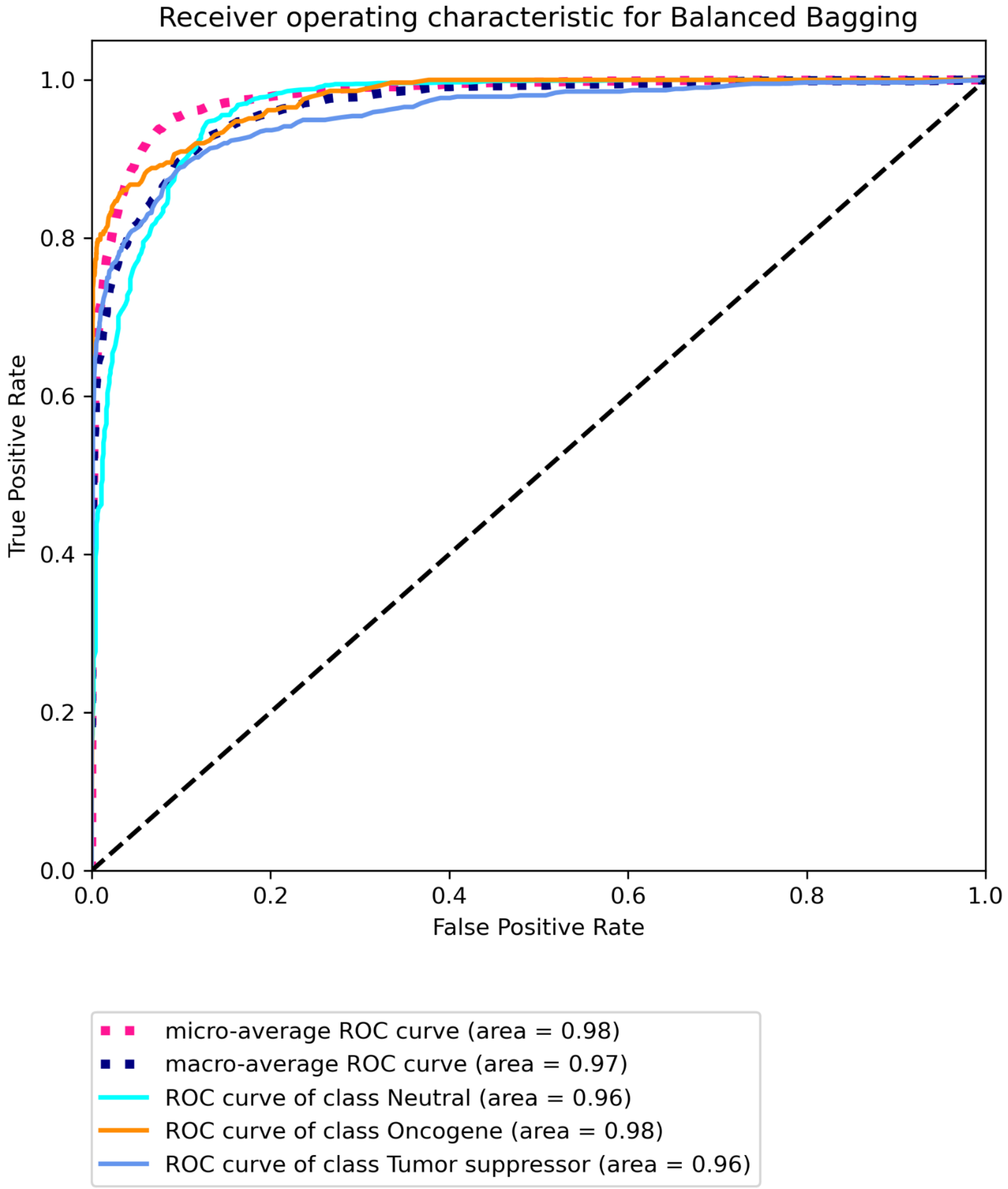

Figure S41C. Receiver operating characteristic for LUAD using multi-omic data with CGC labels built on “all” features using Balanced Bagging.

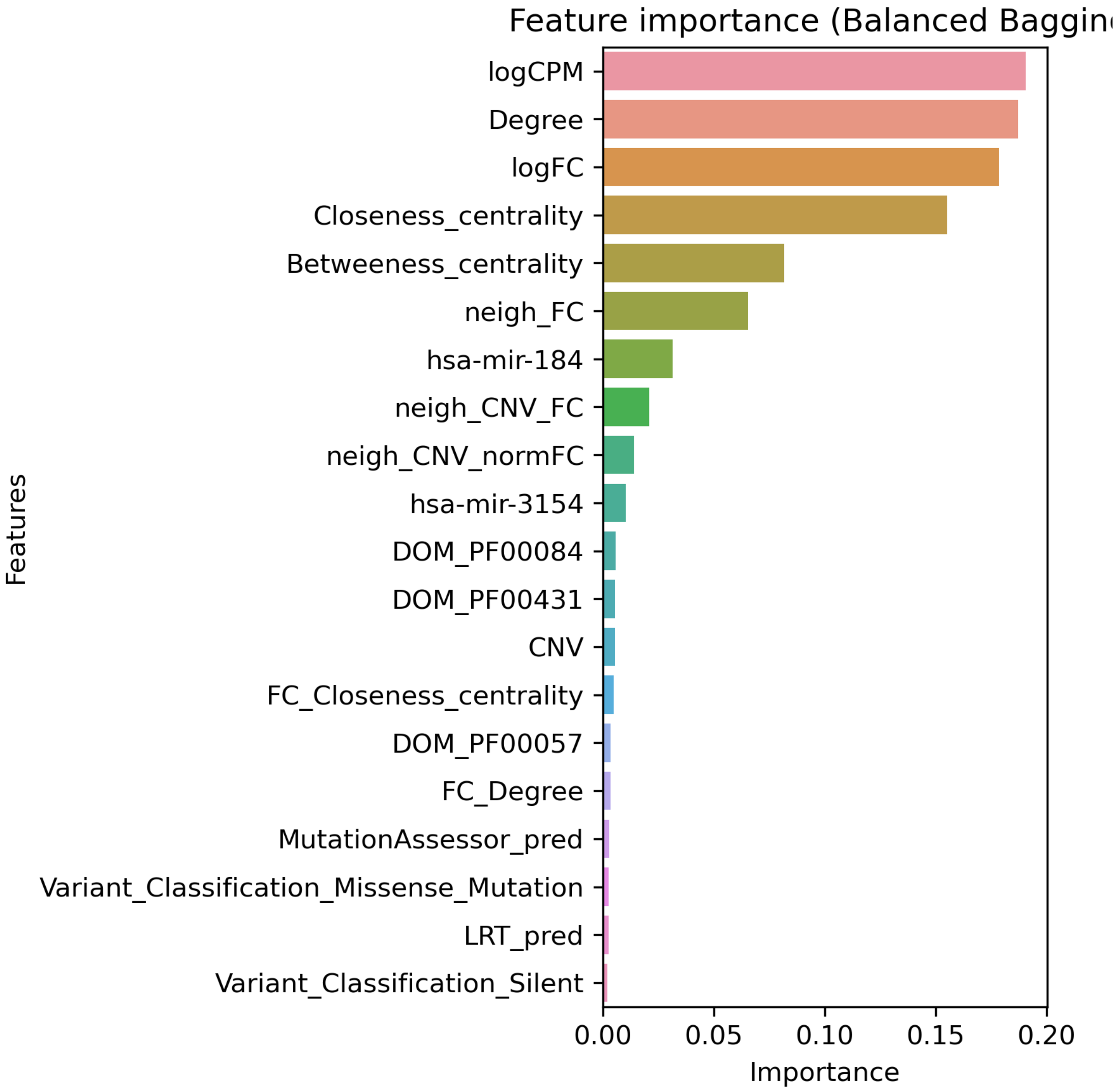

Figure S42A. Top 20 features for LUAD using multi-omic data with CGC labels built on "some" features using Balanced Bagging.

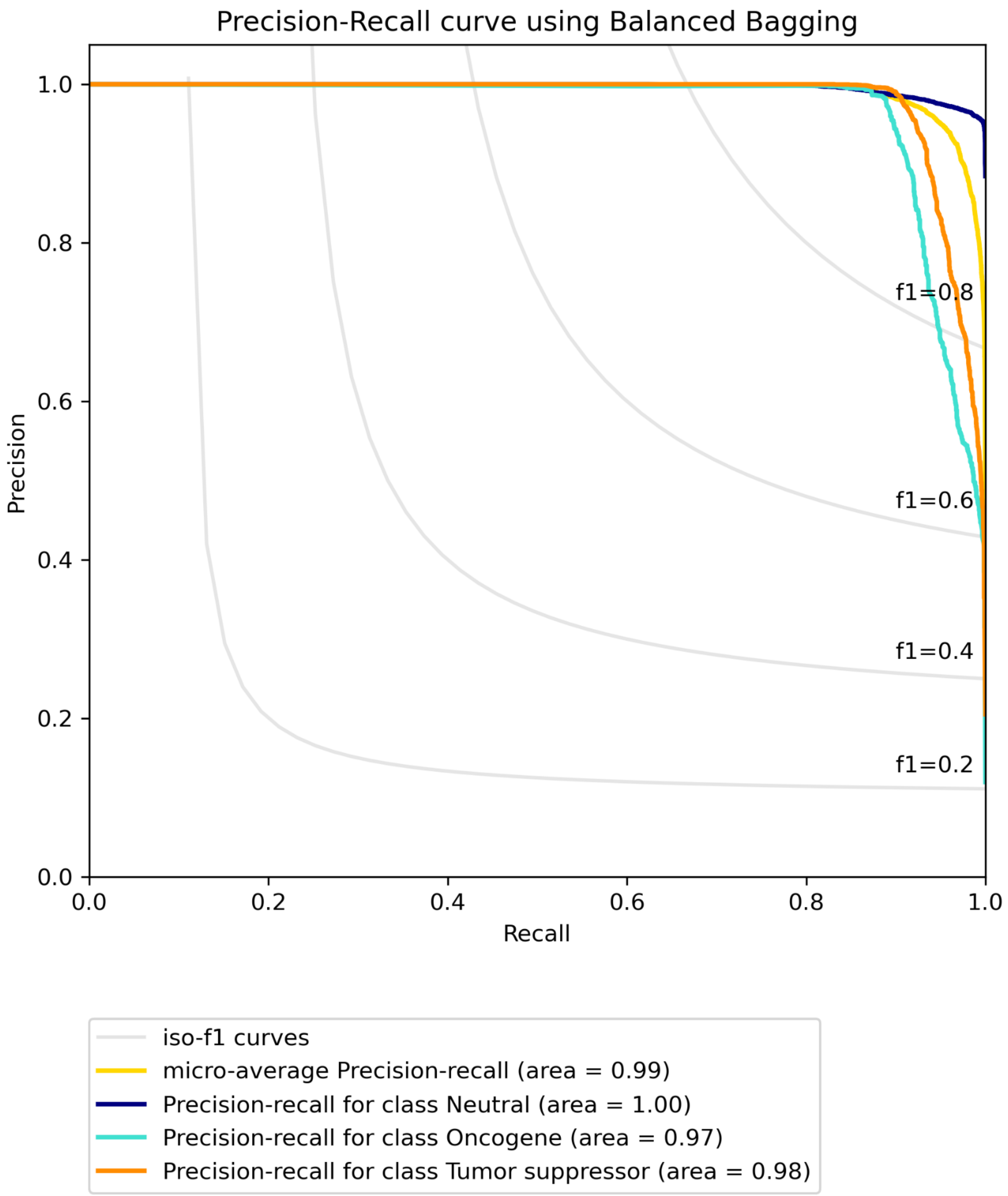

Figure S42B. Precision-recall curve for LUAD using multi-omic data with CGC labels built on “some” features using Balanced Bagging.

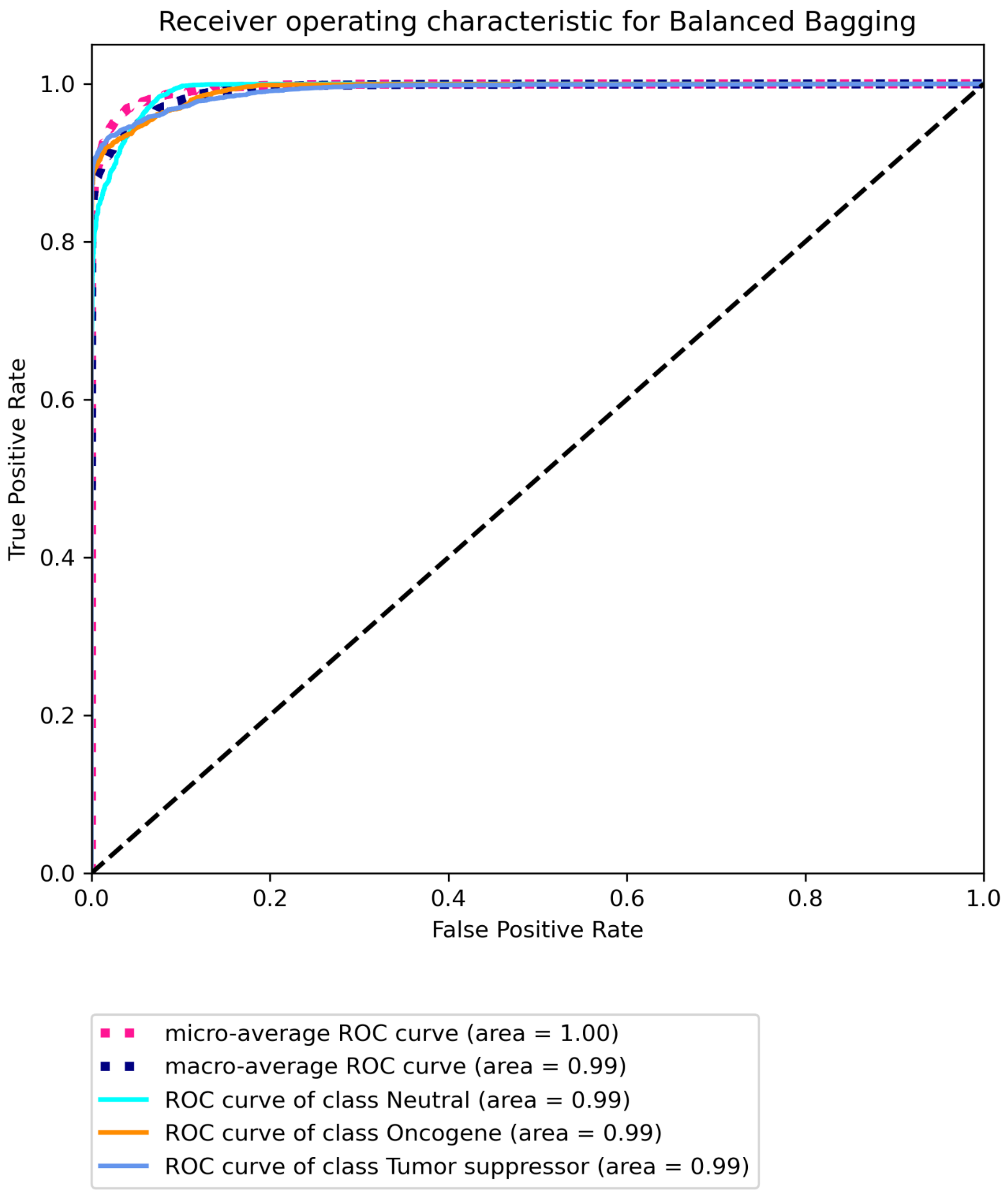

Figure S42C. Receiver operating characteristic for LUAD using multi-omic data with CGC labels built on "some" features using Balanced Bagging.

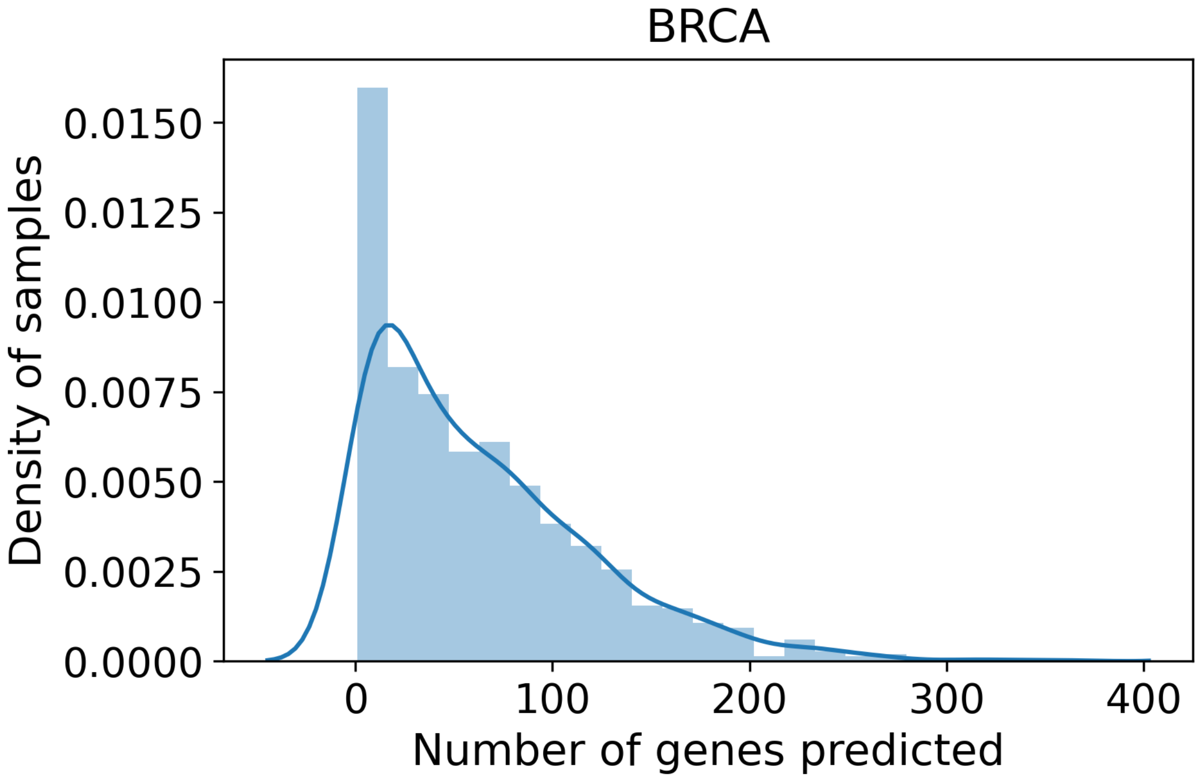

Figure S43. Distribution of genes predicted for samples in BRCA.

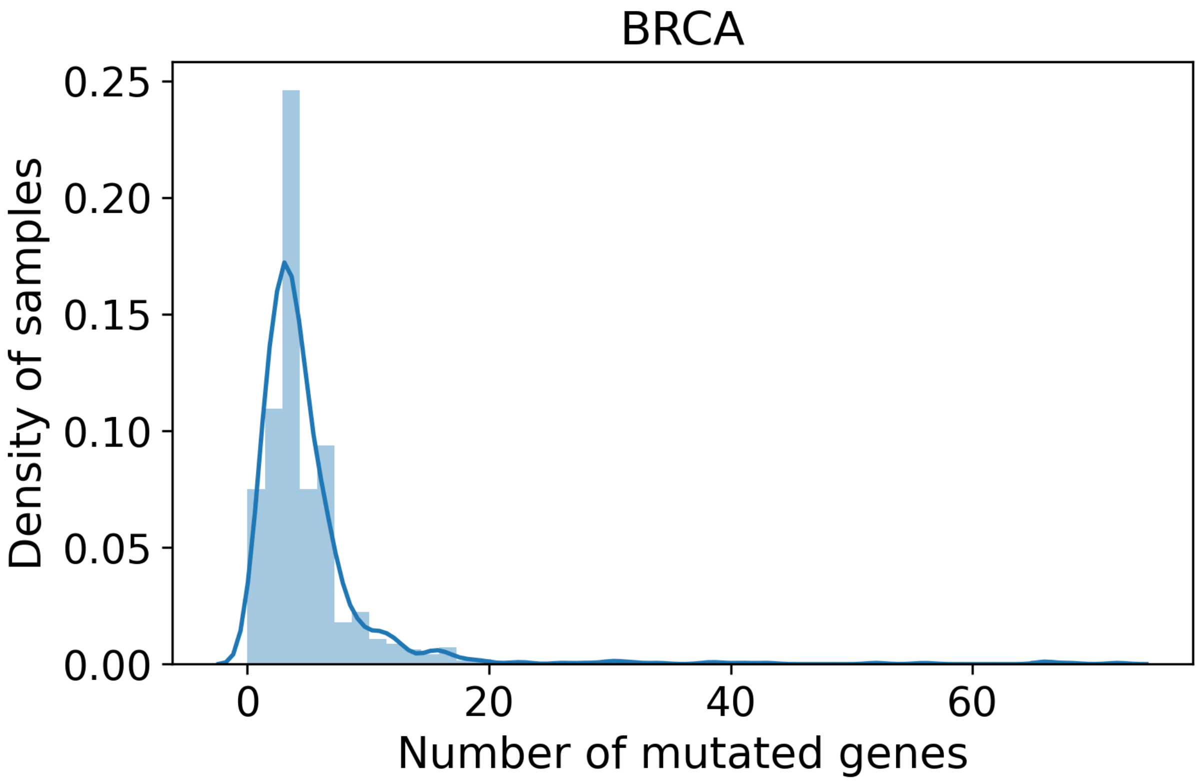

Figure S44. Distribution of mutated genes predicted for samples in BRCA.

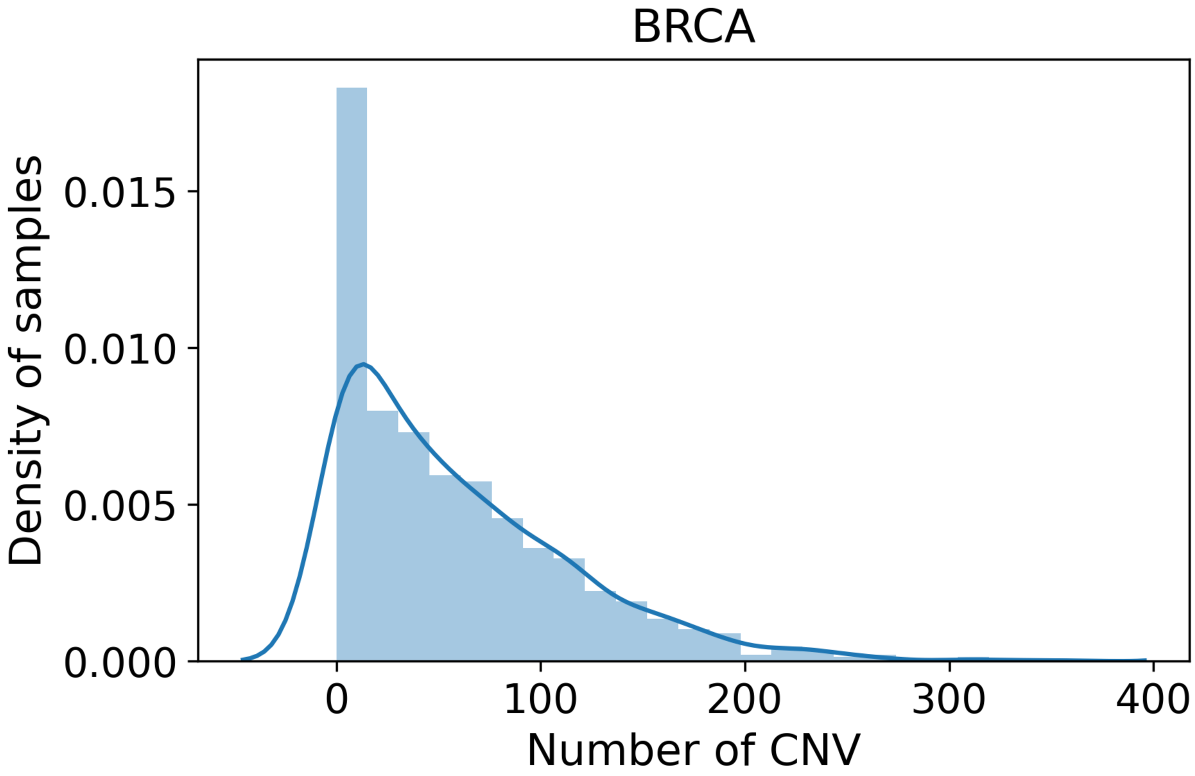

Figure S45. Distribution of CNV altered genes predicted for samples in BRCA.

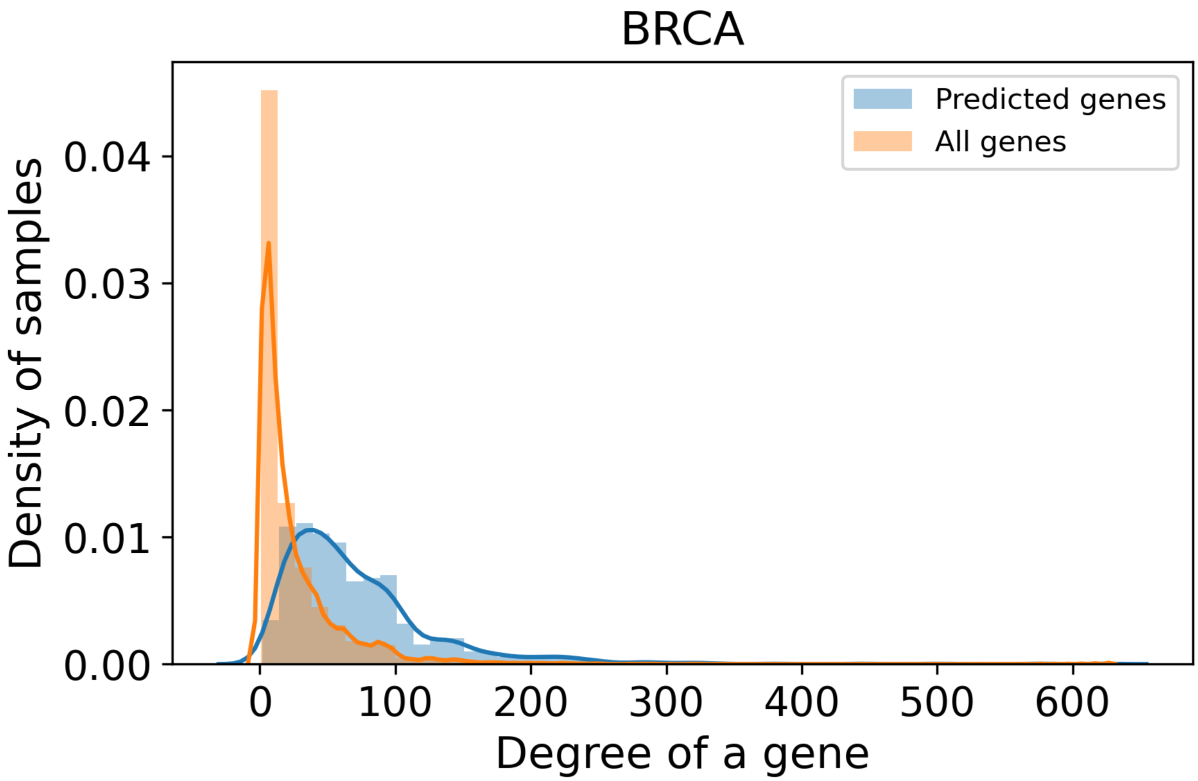

Figure S46. Distribution of degree of genes predicted for samples in BRCA.

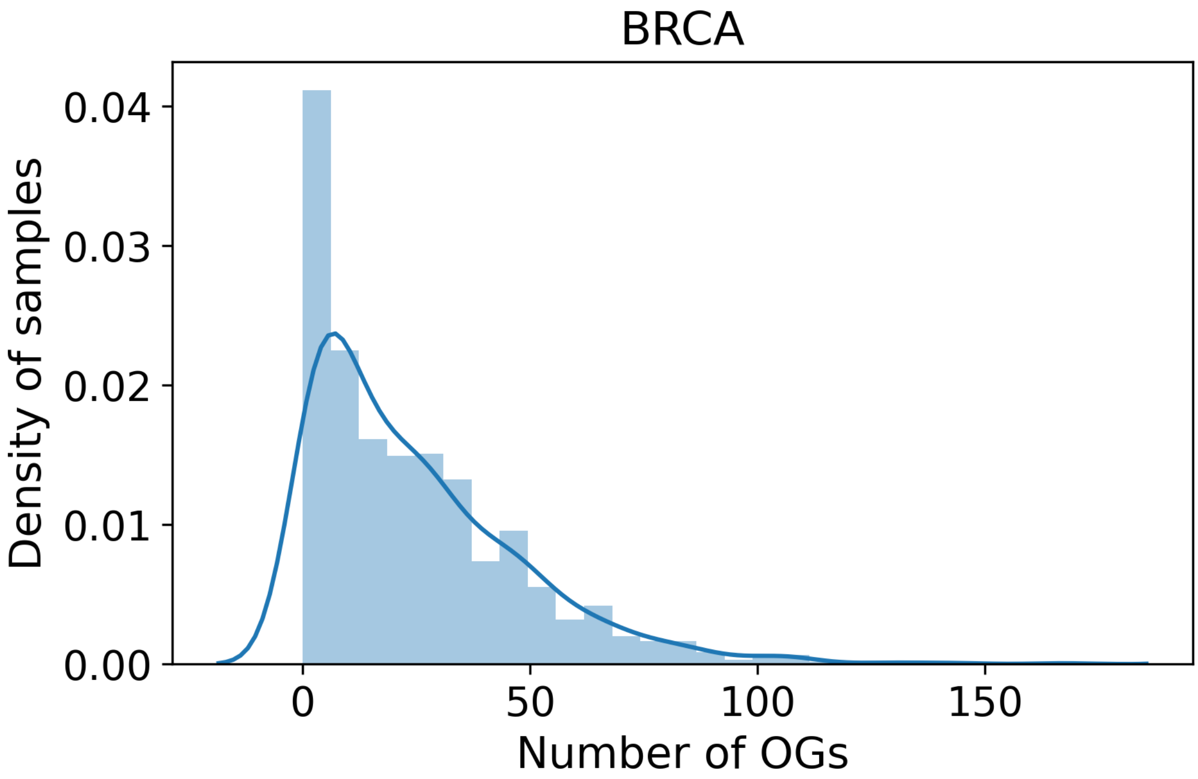

Figure S47. Distribution of predicted oncogenes for samples in BRCA.

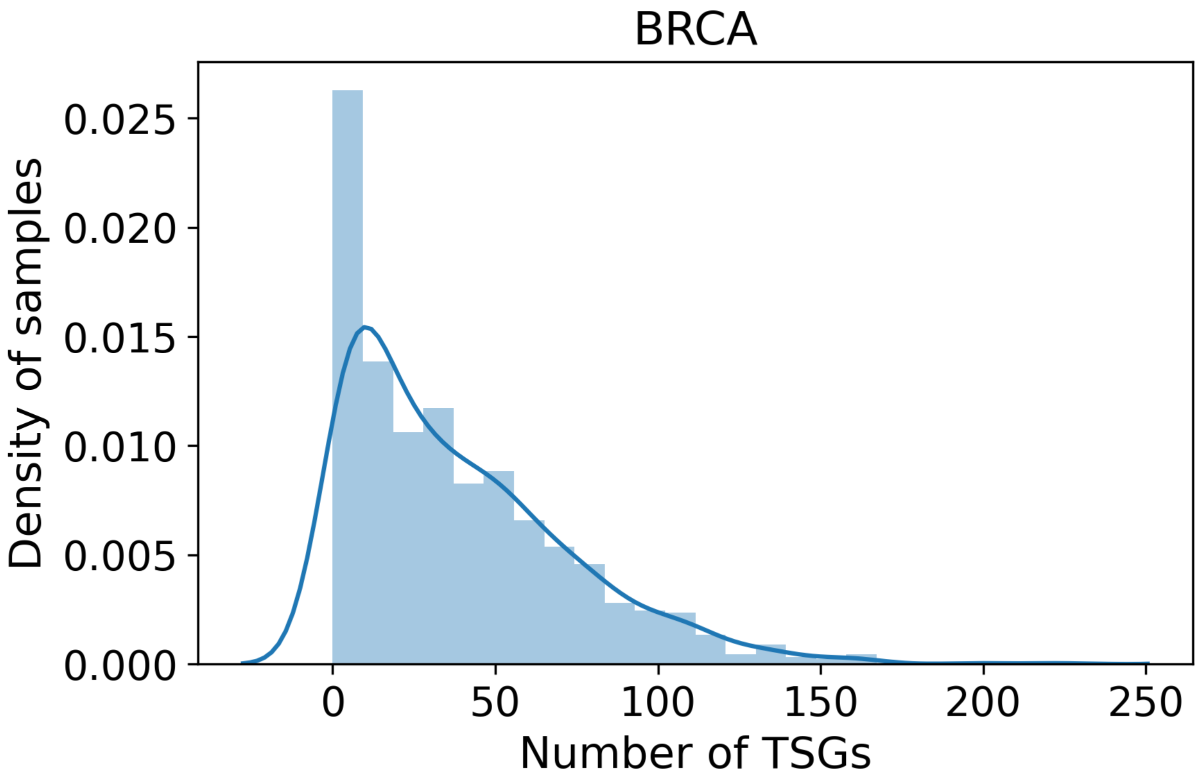

Figure S48. Distribution of predicted tumour suppressor genes in BRCA.

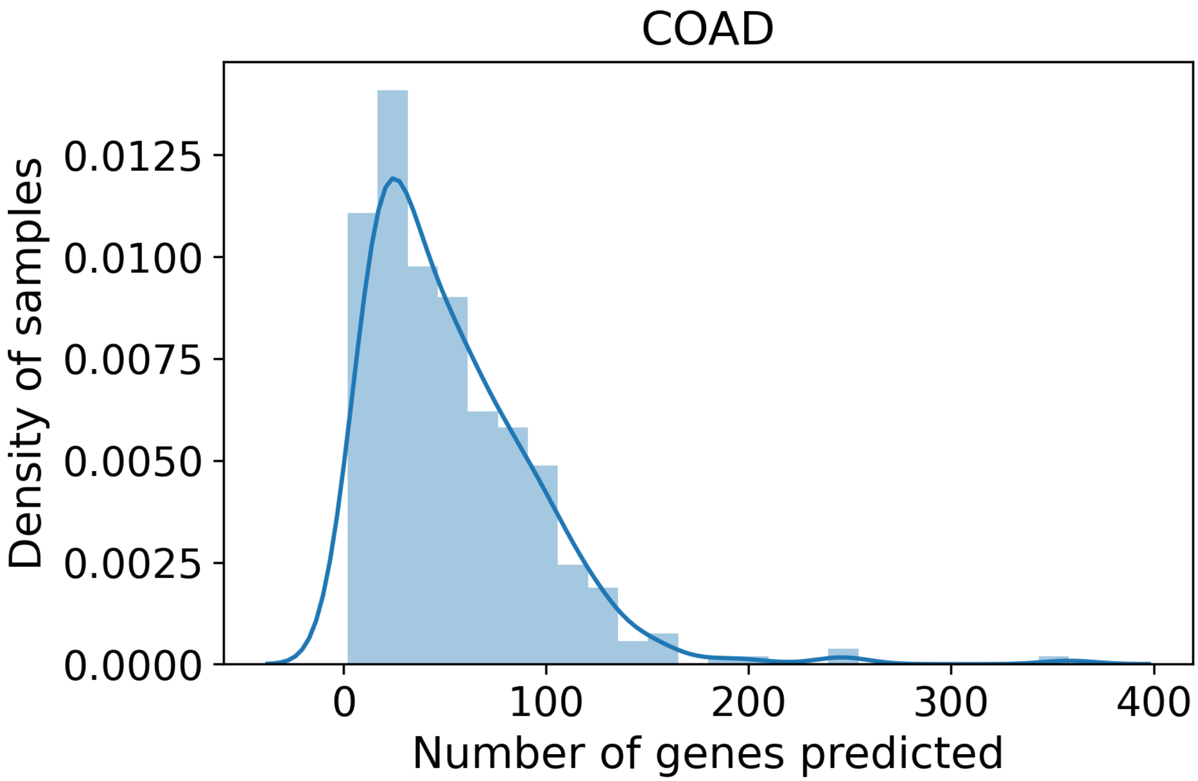

Figure S49. Distribution of genes predicted for samples in COAD.

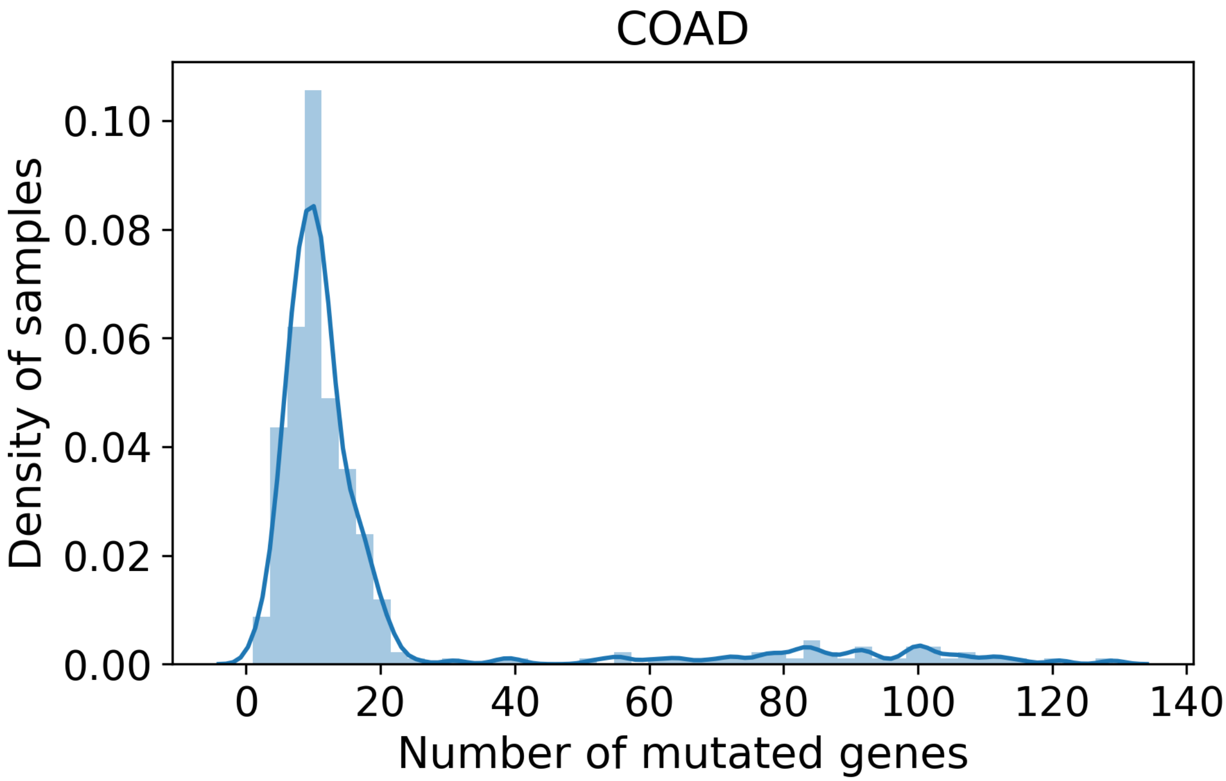

Figure S50. Distribution of mutated genes predicted for samples in COAD.

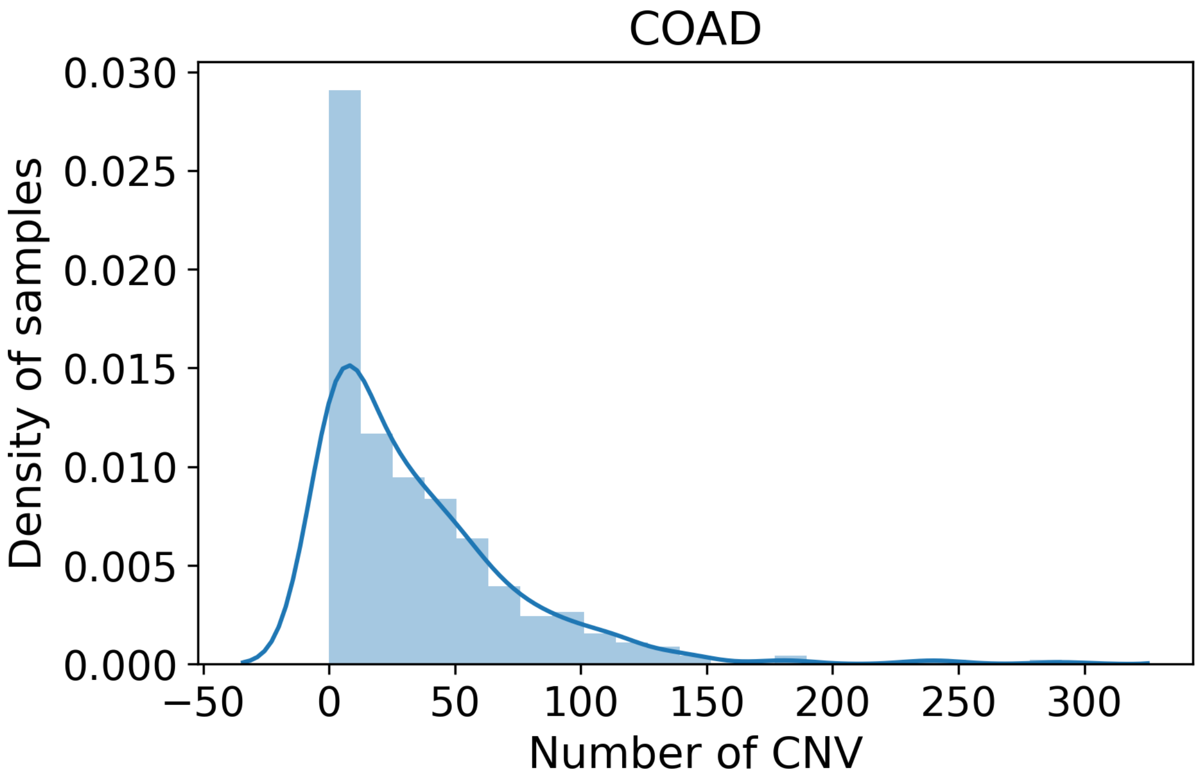

Figure S51. Distribution of CNV altered genes predicted for samples in COAD.

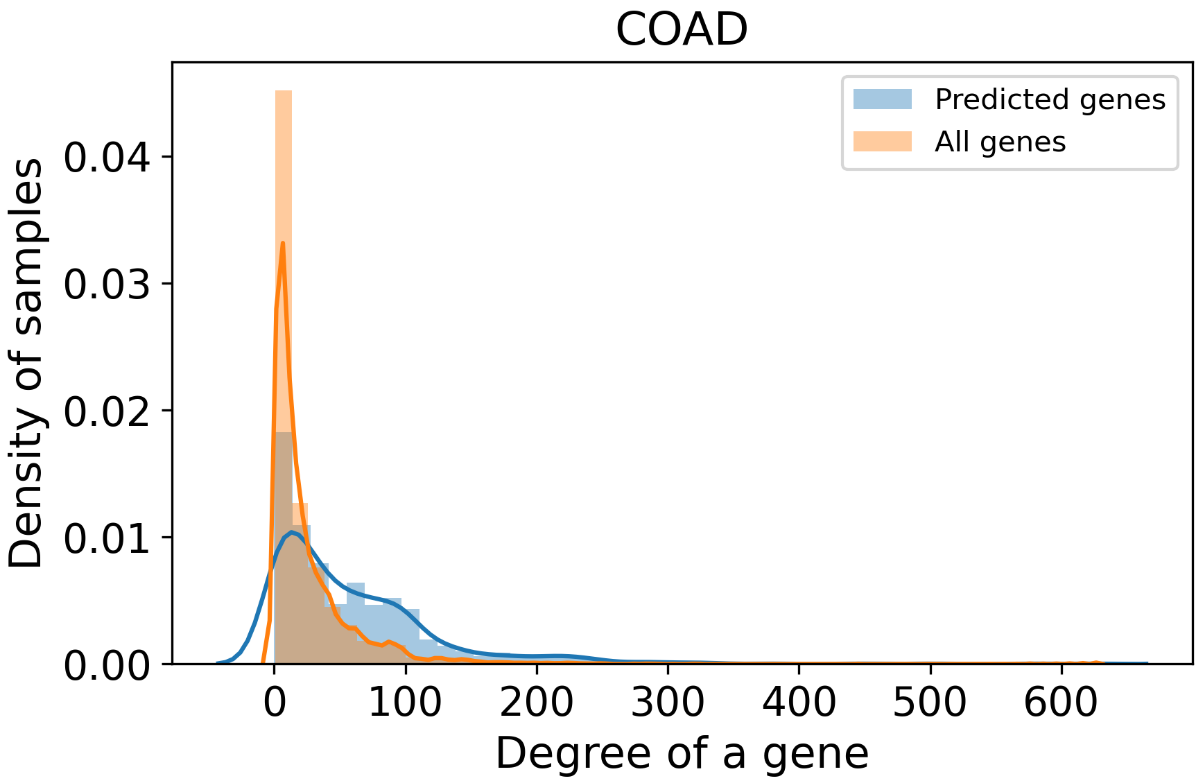

Figure S52. Distribution of degree of genes predicted for samples in COAD.

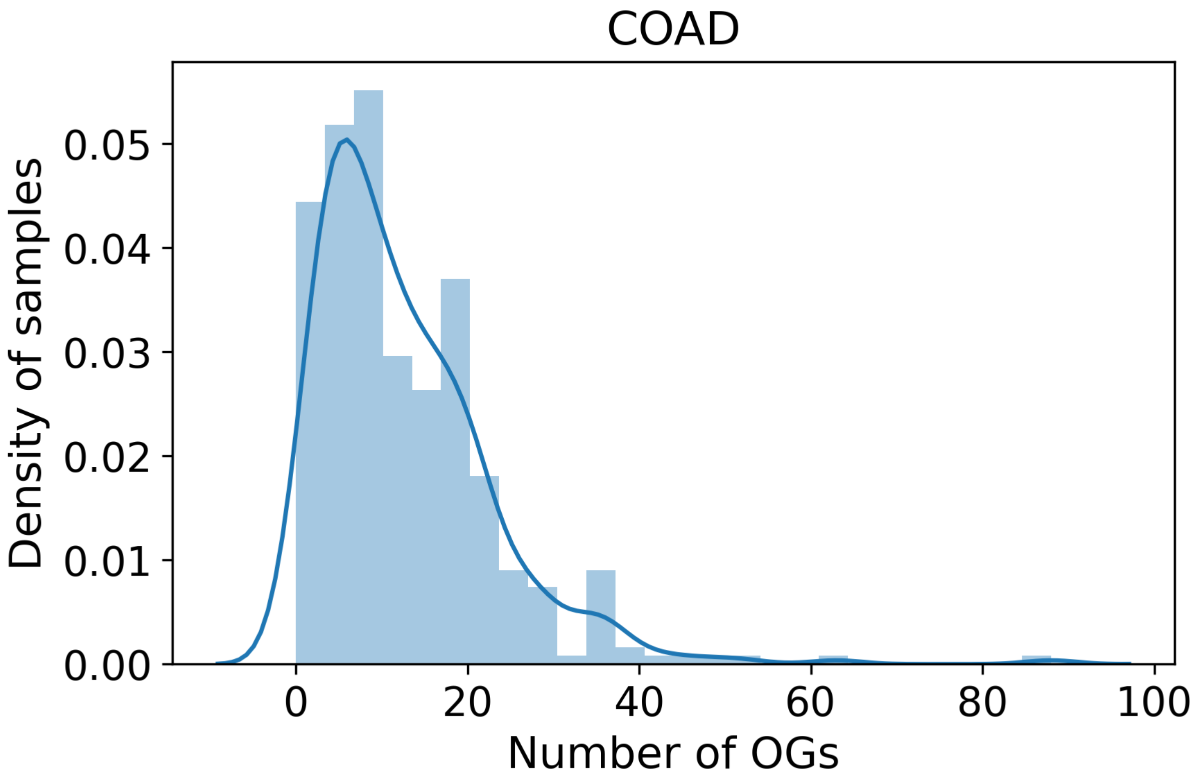

Figure S53. Distribution of predicted oncogenes for samples in COAD.

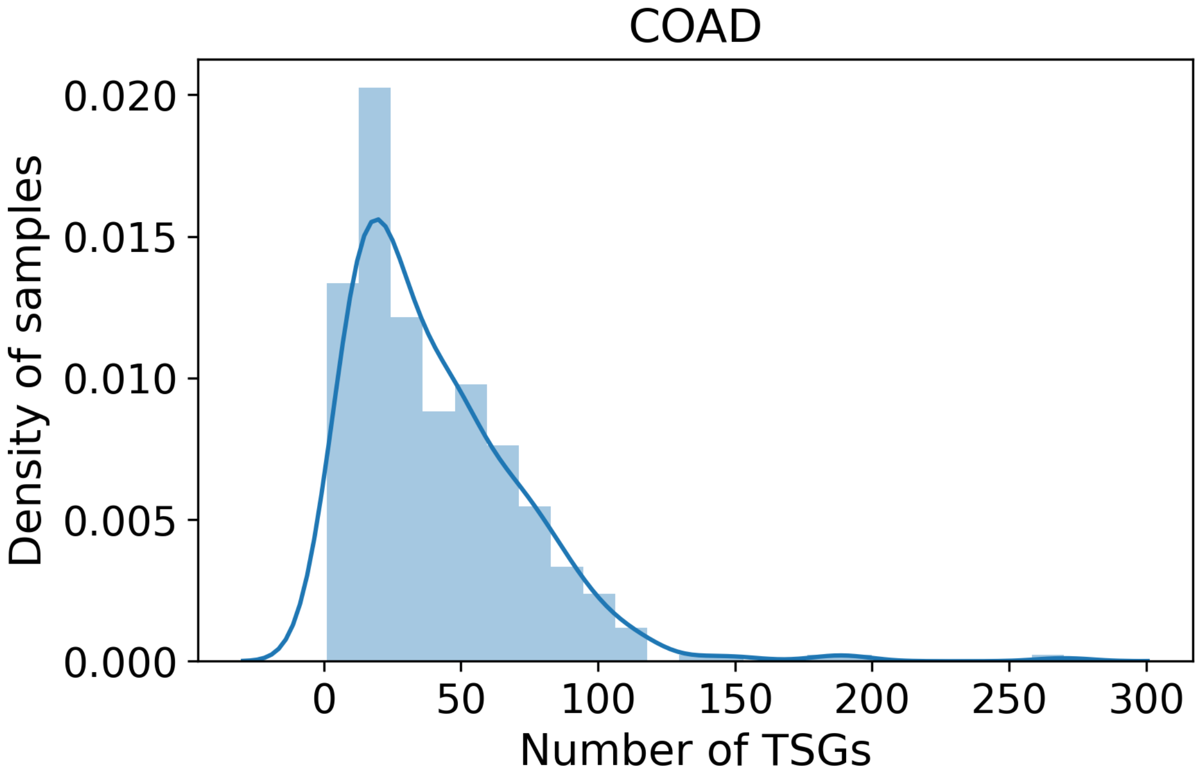

Figure S54. Distribution of predicted tumour suppressor genes in COAD.

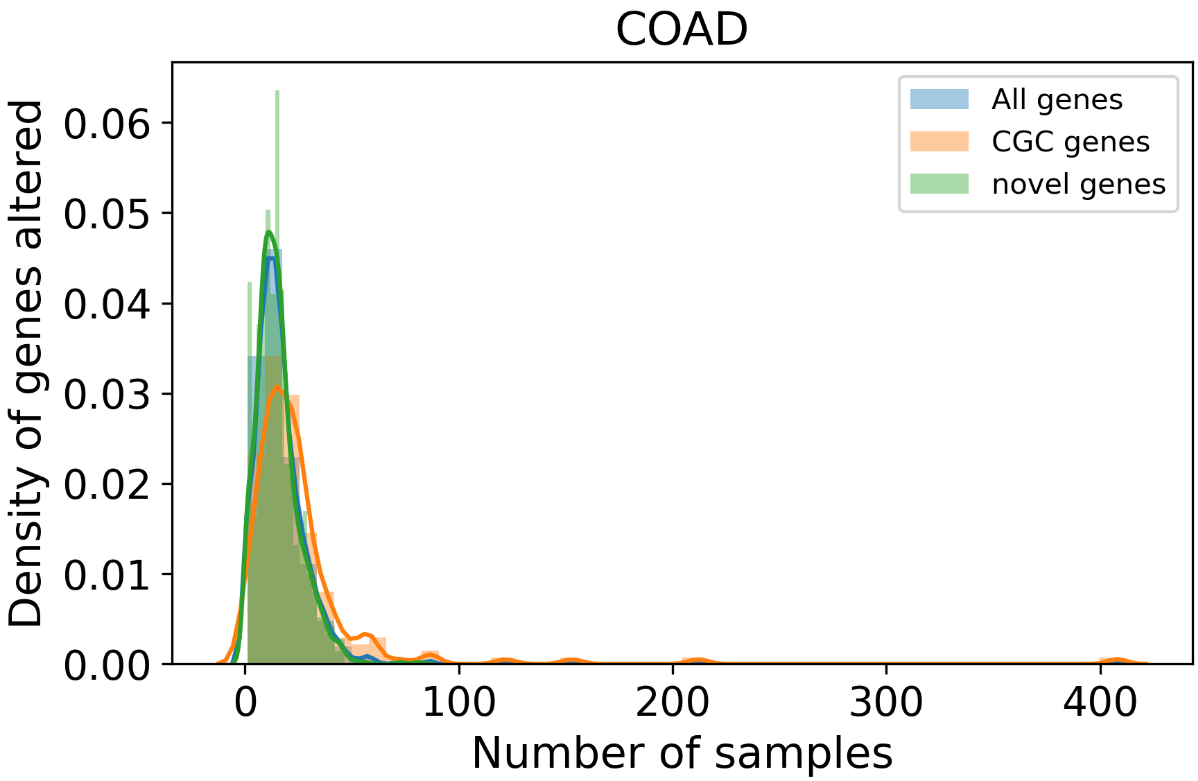

Figure S55. Distribution of samples for genes predicted as driver in COAD.

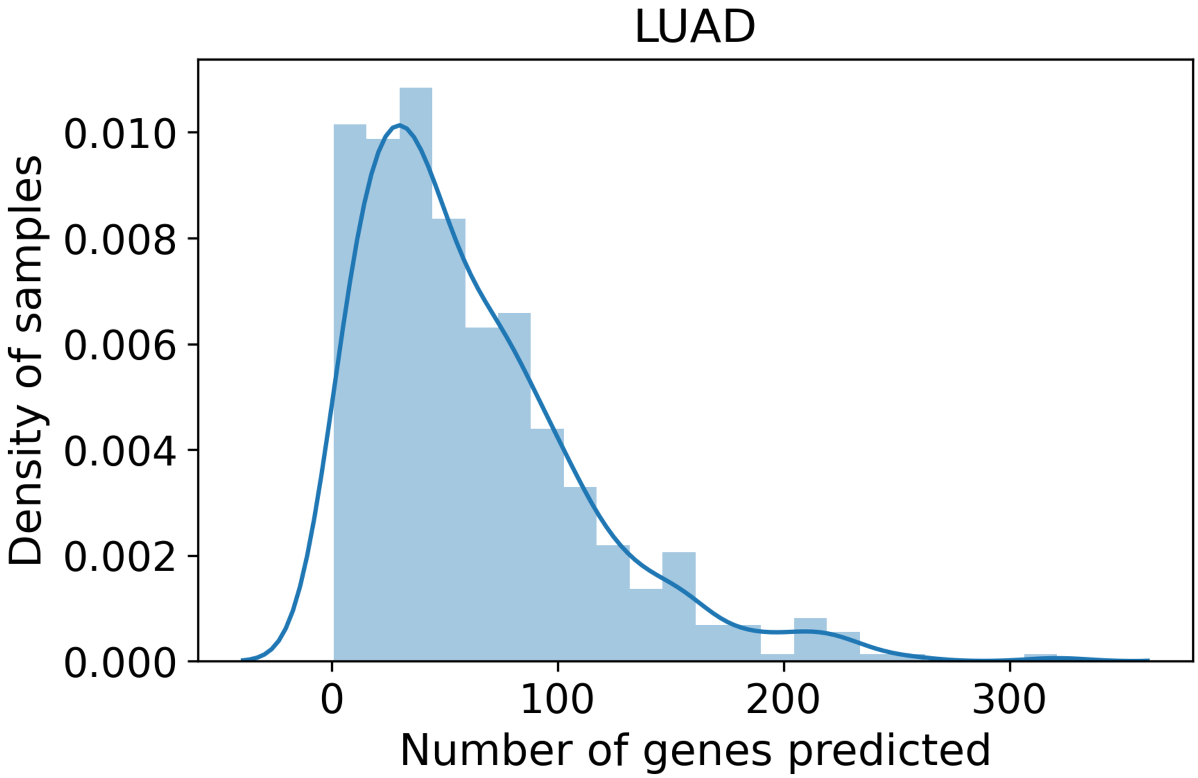

Figure S56. Distribution of genes predicted for samples in LUAD.

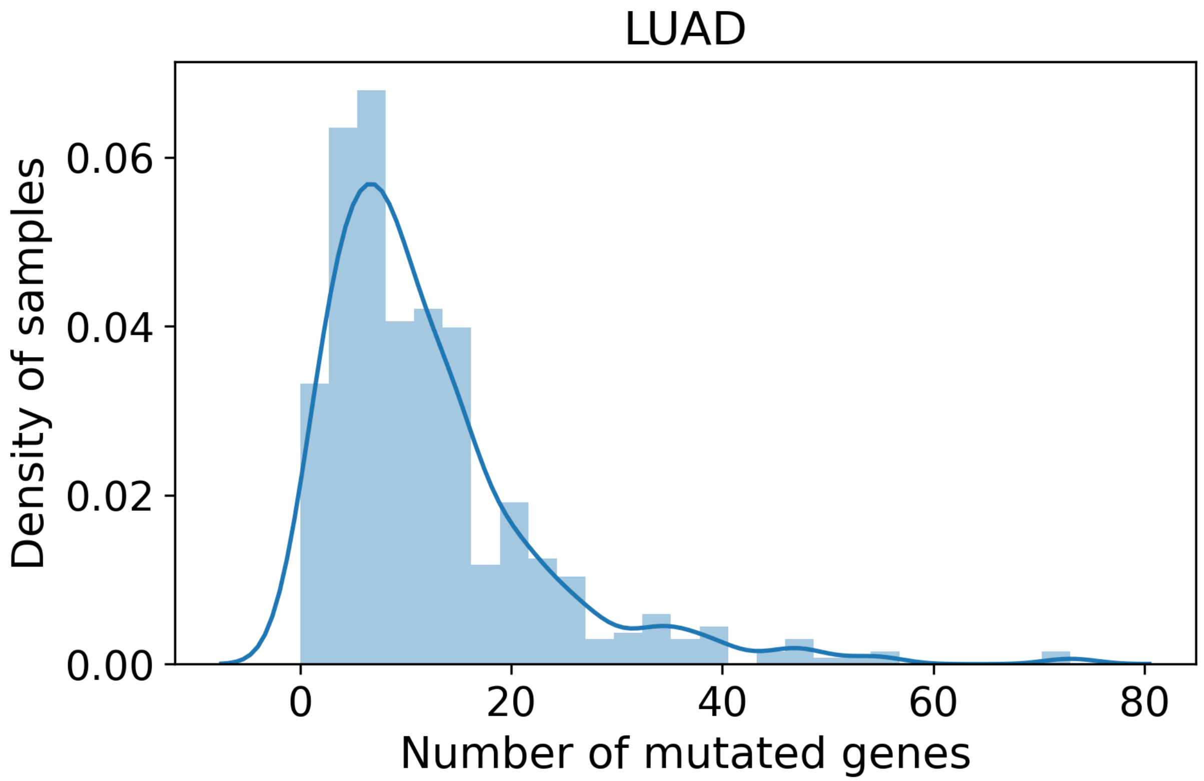

Figure S57. Distribution of mutated genes predicted for samples in LUAD.

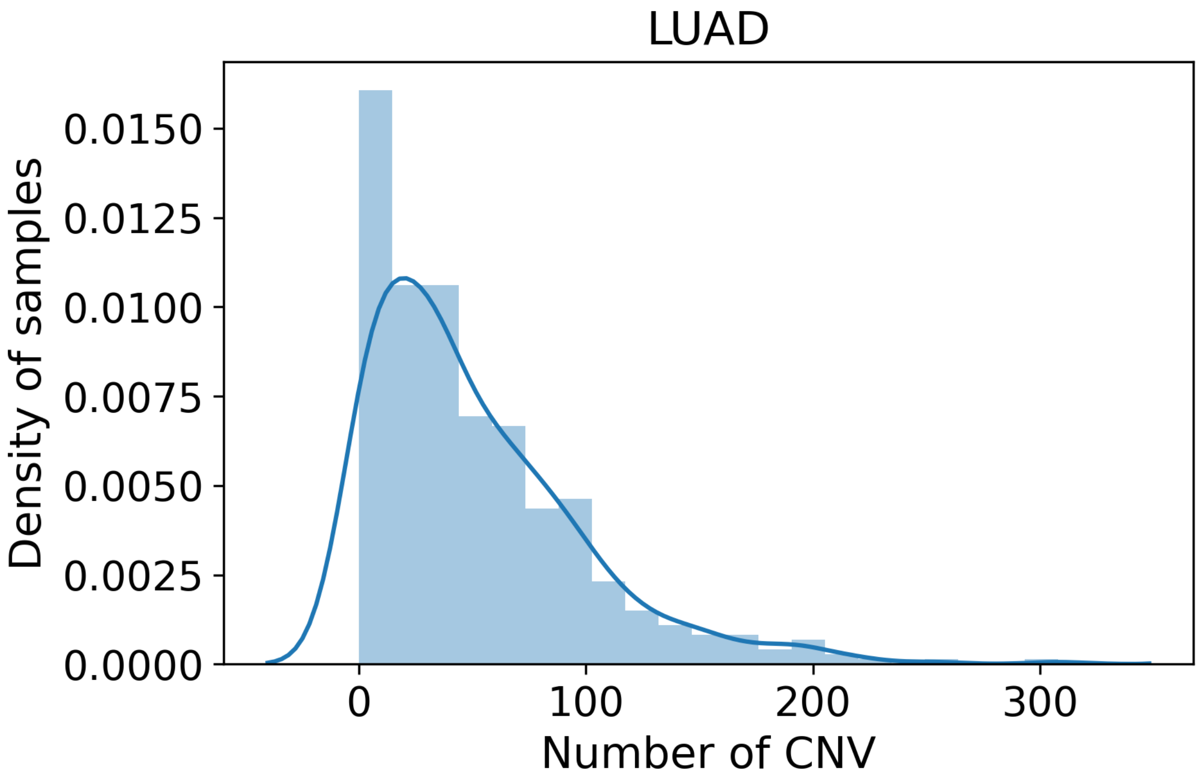

Figure S58. Distribution of CNV altered genes predicted for samples in LUAD.

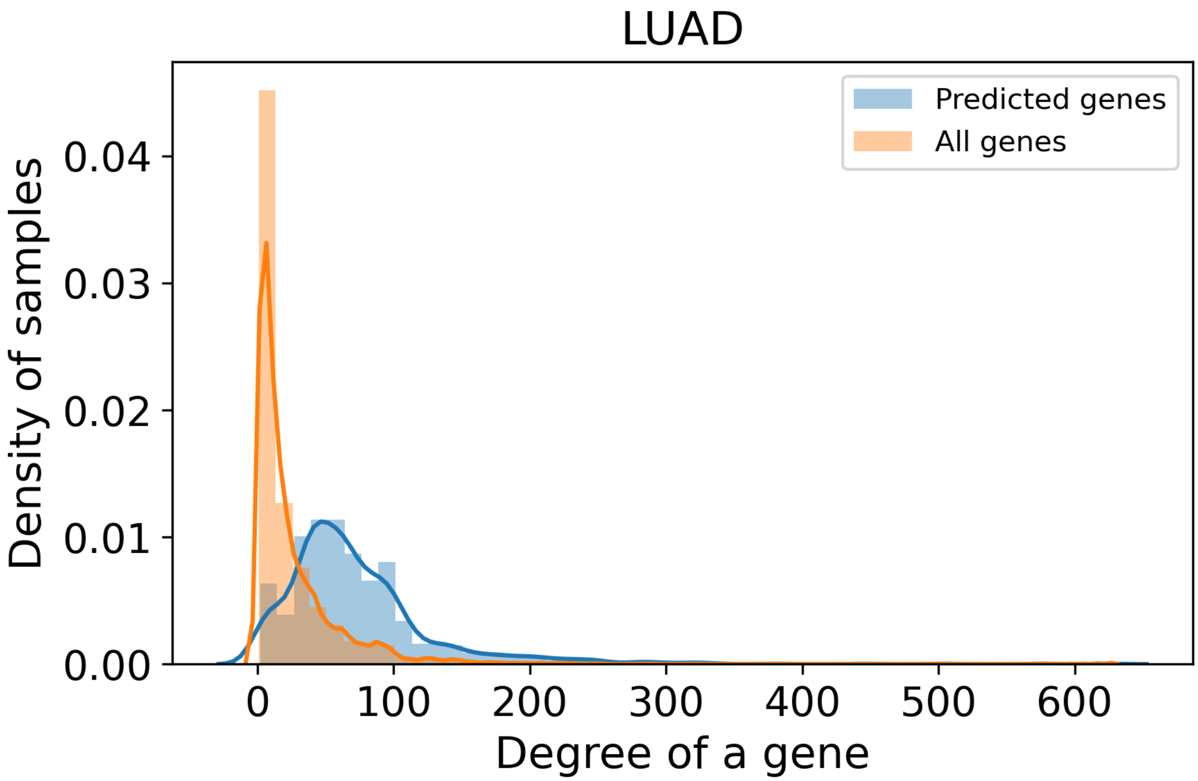

Figure S59. Distribution of degree of genes predicted for samples in LUAD.

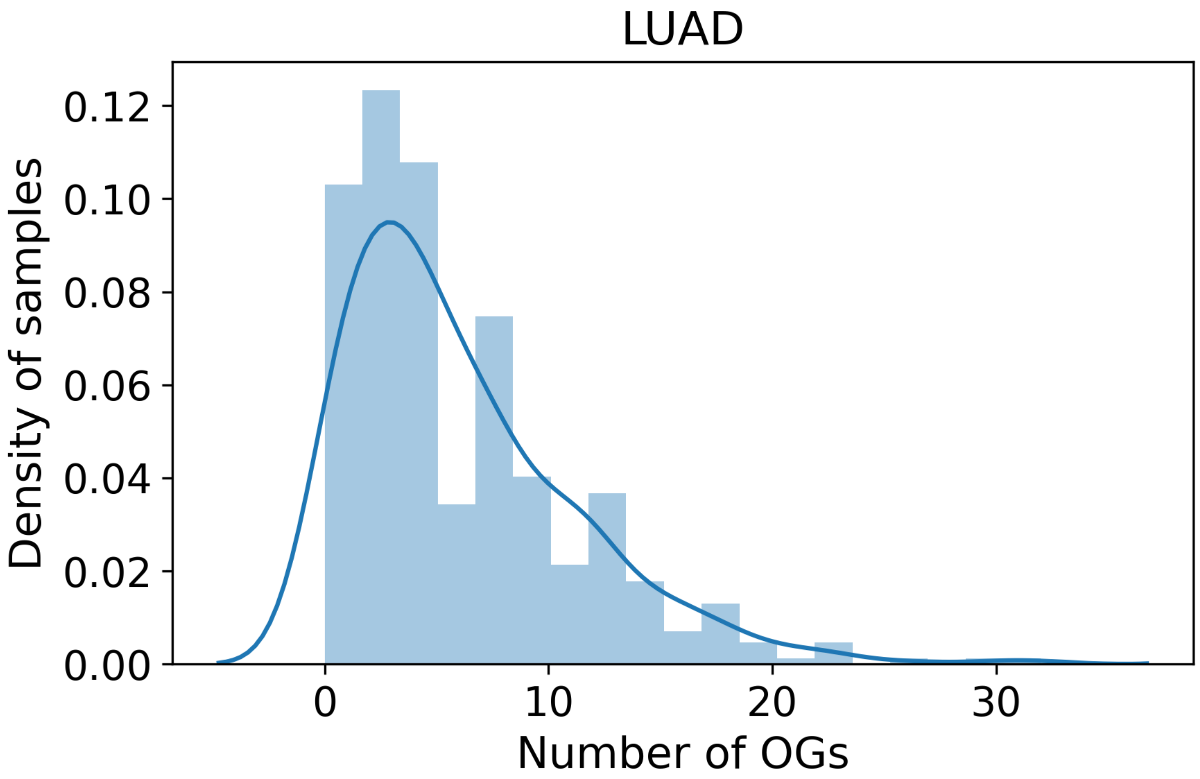

Figure S60. Distribution of predicted oncogenes for samples in LUAD.

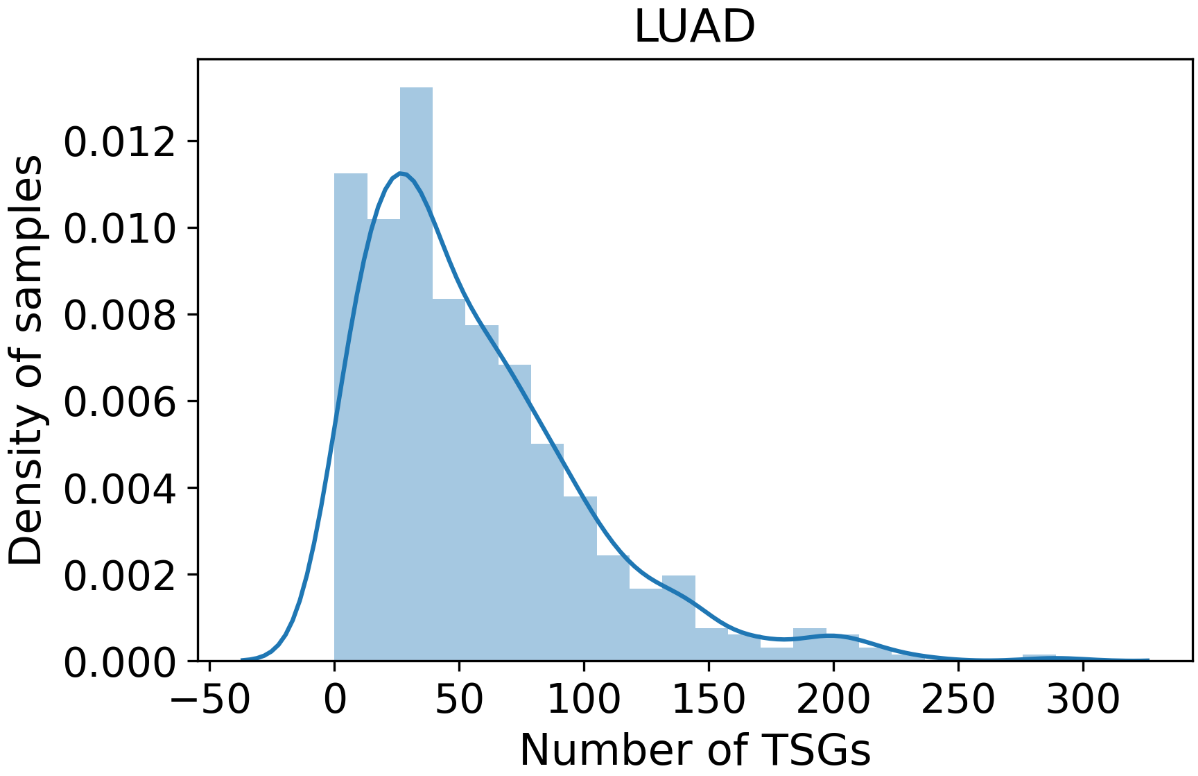

Figure S61. Distribution of predicted tumour suppressor genes in LUAD.

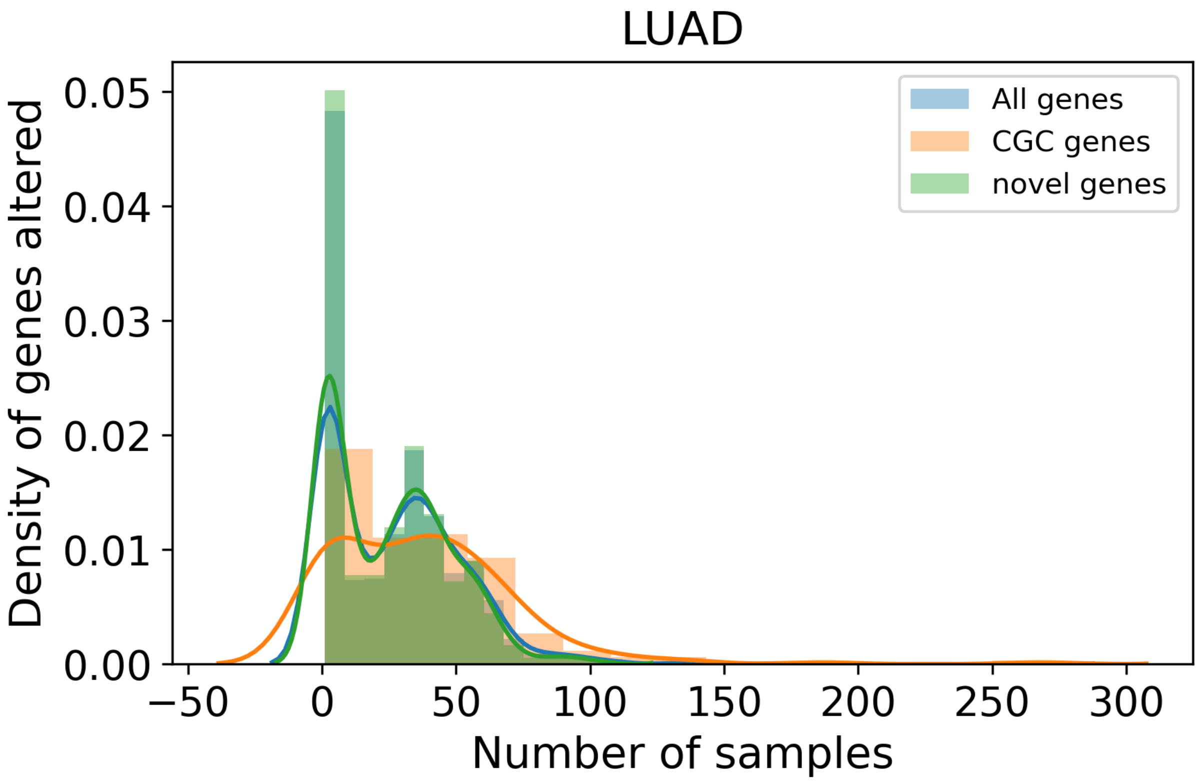

Figure S62. Distribution of samples for genes predicted as driver in LUAD.
